# The diagnostic trajectory of infants and children with clinical features of genetic disease

**DOI:** 10.1038/s41525-021-00260-2

**Published:** 2021-11-22

**Authors:** Brock E. Schroeder, Nina Gonzaludo, Katie Everson, Kyi-Sin Than, Jeff Sullivan, Ryan J. Taft, John W. Belmont

**Affiliations:** 1 Illumina, Inc., San Diego, CA USA; 2 PRECISIONheor, Los Angeles, CA USA

**Keywords:** Diseases, Genetics, Genetic testing

## Abstract

We characterized US pediatric patients with clinical indicators of genetic diseases, focusing on the burden of disease, utilization of genetic testing, and cost of care. Curated lists of diagnosis, procedure, and billing codes were used to identify patients with clinical indicators of genetic disease in healthcare claims from Optum’s de-identified Clinformatics® Database (13,076,038 unique patients). Distinct cohorts were defined to represent permissive and conservative estimates of the number of patients. Clinical phenotypes suggestive of genetic diseases were observed in up to 9.4% of pediatric patients and up to 44.7% of critically-ill infants. Compared with controls, patients with indicators of genetic diseases had higher utilization of services (e.g., mean NICU length of stay of 31.6d in a cohort defined by multiple congenital anomalies or neurological presentations compared with 10.1d for patients in the control population (*P* < 0.001)) and higher overall costs. Very few patients received any genetic testing (4.2–8.4% depending on cohort criteria). These results highlight the substantial proportion of the population with clinical features associated with genetic disorders and underutilization of genetic testing in these populations.

## Introduction

Rare diseases represent a significant clinical and economic burden, with >25 million Americans estimated to be living with a rare disease^1^. Our understanding of the genetic etiologies of many rare diseases has grown substantially in recent years^1,2^. Although most diseases have complex genetic determinants, many rare diseases are caused by pathogenic variants in single genes or by rare DNA copy number abnormalities. For example, in two recent analyses of the Orphanet database, 70–80% of rare diseases were found to be genetic or have genetic subtypes^2,3^. The Online Mendelian Inheritance in Man (OMIM) compendium lists over 6500 clinical phenotypes for which a molecular basis is known and 4200 genes with phenotype-causing pathogenic variants^4^. These numbers have continued to grow as information on genetic variation and gene–phenotype relationships emerge from various research projects including those utilizing comprehensive genomic sequencing approaches^5–10^. Furthermore, as our understanding of the genetic etiology of diseases has grown, our ability to treat many of these conditions has improved, including with targeted pharmaceuticals, vitamin supplementation or dietary restrictions, protein and enzyme replacement therapies, stem cell and organ transplantation, and most recently novel precision medicine approaches such as gene replacement therapy^5,11–17^.

The American College of Medical Genetics and Genomics (ACMG) has highlighted that there is “great clinical value in arriving at a precise medical diagnosis, enabling, among other things, identification of a disorder’s cause and prognosis, as well as frequently informing preventive and treatment modalities.”^18^. A key challenge, however, is that many genetic diseases are difficult to diagnose owing to nonspecific clinical presentations and physician unfamiliarity—leading to delays in diagnosis and initiation of precision care. These challenges are compounded by traditional diagnostic approaches, which focused on subjective assessment, non-genetic tests and imaging, and iterative application of single-gene tests or panels with limited diagnostic yield. In recent years, genome-wide approaches using whole-exome and whole-genome sequencing have demonstrated substantial improvement in diagnostic yield compared with traditional approaches^5,12,14,15,19–23^. These improvements have led to policy changes related to care delivery. For example, the National Health Service (NHS) in England has announced the commission of genome sequencing as part of routine care for patients with undiagnosed rare diseases. In the US, the majority of pediatric lives are covered for exome sequencing (ES) by commercial and public health insurance programs, with some also covering genome sequencing (Data on file from https://www.policyreporter.com/).

Although the pace of research and understanding of genetic diseases has accelerated, there has been little real-world evidence published on population-based epidemiology and the care of patients with clinical indicators of genetic diseases. In a recent study of pediatric inpatients in the US using the 2012 version of the Healthcare Cost and Utilization Project (HCUP) Kid’s Inpatient Database (KID), we estimated that up to 14% of pediatric inpatient stays had indications of genetic disease^24^. Given the burgeoning opportunity to improve the care of patients with genetic diseases, understanding their clinical and diagnostic assessment will be important to improving care delivery. Data are needed to inform our understanding of the potential impact of new interventions such as whole-genome sequencing. The clinical features that guide such testing, which are individually uncommon, are often present in patients with rare and ultrarare genetic diseases and are utilized by specialists to guide genetic testing. However, testing is not uniformly applied to owe to differences in physician practice, delays in access to specialist consultation, complex and non-standardized laboratory platforms, regional and urban/rural disparities in access, and non-uniform health insurance coverage policies. The objective of this study was to characterize the population of patients with clinical features of genetic diseases in a large US commercial healthcare claims database, with a focus on the burden of disease, utilization of genetic testing, and cost of care. Separate analyses were performed for critically-ill infants and the general pediatric population.

## Results

### Cohort definitions

A total of 13,076,038 unique patients were included in the source data set selected. Of these, 11,036,263 subjects were identified and retained as a control for the pediatric population, and 1,148,695 subjects that met all birth and enrollment criteria were retained as controls for the newborn population (Table 1). Within each population (pediatric and critically-ill newborn), analysis cohorts were identified through a combination of diagnosis codes, procedure codes, and revenue codes, with combinations of these codes used to define three different cohorts with different levels of stringency: (1) a Broad cohort with clinical indicators suggestive of a genetic disease; (2) a more specific subset of clinical presentations that are broadly accepted indications for genetic testing [multiple congenital anomalies (MCA), moderate or severe intellectual disability (ID), developmental delay (DD), or epilepsy/seizures (E)]; and (3) a conservative cohort that had indicators of undiagnosed genetic disease as well as ≥1 genetic test (see Methods for additional details on cohort definitions and selection). Details of the sample selection and attrition are shown in Supplementary Table 1.

**Table 1:** Population demographics.

	Critically-ill newborn cohort				Pediatric cohort			
	Control	Broad	MCA/ID/DD/E	Conservative	Control	Broad	MCA/ID/DD/E	Conservative
Sample size	1,148,695	29,348	8723	1924	11,036,263	1,139,035	94,714	28,348
*Patient characteristics*								
Age at index, mean years	0.0	0.0	0.0	0.0	7.5 (5.5)	7.3 (6.1)*	5.0 (5.8)*	6.4 (6.0)*
Length of enrollment, mean days (SD)	602.5 (747.8)	649.0 (756.0)*	823.6 (819.7)*	733.2 (788.5)*	735.3 (794.5)	1494.0 (1096.0)*	1470.0 (1064.0)*	1734.0 (1188.0)*
Age at enrollment, mean days (SD)	4.7 (3.3)	0.7 (3.3)	1 (2.9)	0.6 (3.1)	2903.0 (2048.0)	2180.0 (2045.0)*	1523.0 (1914.0)*	1823.0 (1927.0)*
Male, % (SD)	49.7 (50.0)	57.7(49.4)*	60.6 (48.9)*	57.7 (49.4)*	50.9 (50.0)	53.7 (50.0)*	58.7 (40.2)*	53.3 (50.0)*
White, % (SD)	64.3 (47.9)	64.0 (48.0)	65.7 (47.5)*	65.4 (47.6)	60.6 (48.9)	64.3 (47.9)*	64.5 (47.9)*	62.9 (48.3)*
Hispanic, %	13.0	11.9	10.6	14.6	12.9	10.3	10.4	11.9
Black, %	7.7	9.0	8.7	7.4	9.1	6.7	7.2	5.6
Asian, %	7.2	7.2	6.3	5.6	5.1	5.3	5.6	5.2
Race Unknown, %	7.8	7.9	8.6	7.0	12.4	13.5	12.3	14.5
Below 400% poverty line, % (SD)	0.3 (5.8)	0.4 (6.0)*	0.4 (6.2)	0.2 (4.7)	0.6 (7.8)	0.4 (6.3)*	0.5 (6.7)*	0.4 (6.5)*
*Categorization of index event (%)*								
Possible	N/A	83.5	94.2	71.8	N/A	79.1	69.6	62.8
Probable	N/A	10.8	5.1	22.5	N/A	10.3	30.1	20.4
Definite	N/A	6.9	0.8	0.0	N/A	3.8	0.3	0.0
Genetic test	N/A	0.2	0.0	7.9	N/A	0.9	0.0	21.1
Other CPT/HCPCS	N/A	0.1	0.0	0.0	N/A	9.1	0.0	0.0
*Clinical presentation (ICD-9/10 category) (%)*								
Skin/subcutaneous system	N/A	0.0	0.0	0.0	N/A	0.8	0.0	0.3
Mental/behavioral/neurodevelopmental	N/A	0.0	0.0	0.0	N/A	3.7	7.8	11.8
Endocrine/nutritional/metabolic	N/A	5.8	0.0	10.0	N/A	9.9	0.0	15.2
Circulatory system	N/A	0.9	0.0	0.7	N/A	0.9	0.0	0.8
Congenital anomalies	N/A	62.7	86.4	51.6	N/A	35.5	52.8	26.0
Respiratory system	N/A	0.1	0.0	0.1	N/A	0.0	0.0	0.0
Musculoskeletal system	N/A	0.1	0.0	0.1	N/A	1.2	0.0	0.4
Perinatal period	N/A	11.9	0.0	10.6	N/A	0.7	0.0	1.7
Sense organs	N/A	0.1	0.0	0.2	N/A	1.0	0.0	0.9
Blood disorders	N/A	2.6	0.0	1.5	N/A	1.3	0.0	2.8
Nervous system	N/A	0.6	2.2	0.9	N/A	2.4	32.9	4.2
Digestive system	N/A	0.0	0.0	0.1	N/A	0.1	0.0	0.1
Genitourinary system	N/A	0.0	0.0	0.0	N/A	0.0	0.0	0.0
Symptoms/signs/not otherwise classified	N/A	0.0	0.0	0.0	N/A	0.0	0.0	0.0
Other diagnoses	N/A	0.4	0.0	2.3	N/A	1.1	0.0	3.1

*CPT/HCPCS* Current Procedural terminology/Healthcare common Procedure Coding System (HCPCS), *ICD* International Classification of Diseases, MCA/ID/DD/E: *MCA* multiple congenital anomalies, *ID* intellectual disability, *DD* developmental delay, *E* epilepsy/seizures.

*P < 0.01 vs control.

In the pediatric population, a total of 1,139,035 pediatric patients met the Broad cohort criteria as having one or more clinical features suggestive of an undiagnosed genetic disease, representing 9.4% of the total eligible population (*n* = 12,175,298). A total of 94,714 (0.8% of eligible patients) had ICD-9/10 codes for MCA/ID/DD/E. Finally, 28,348 (0.2% of eligible patients) met the Conservative cohort definition which required at least one genetic test (Table 1).

In the newborn population, 29,348 critically-ill newborns met the Broad cohort criteria. This represented 2.3% of the total eligible newborn population (*n* = 1,303,762) and 44.7% of the total eligible population with a NICU stay (65,695 in the Control and Broad populations). A total of 8723 critically-ill newborns (0.7% of all newborns and 13.3% of all newborns with a NICU stay) had ICD-9/10 codes for MCA/ID/DD/E (primarily MCA and E in this cohort). Finally, 1,924 critically-ill newborns (0.1% of all newborns, and 2.9% of all newborns with a NICU stay) met the Conservative cohort definition which required at least one genetic test (Table 1). There was a sizable number of newborns with a code from our curated list but did not have a qualifying NICU stay (*n* = 125,719 for the Broad cohort) and were therefore excluded from this population and incorporated into the pediatric population.

### Population demographics

Patient demographics are detailed in Table 1. In the pediatric population, the Broad cohort was generally similar in age (7.3 years) to the control group (7.5 years), while the MCA/ID/DD/E cohort (5.0 years) and the Conservative cohort (6.4 years) were somewhat younger at their index date. The proportion of male patients was 53.7% in the Broad population, 58.7% in the MCA/ID/DD/E cohort, and 53.3% in the Conservative cohort, compared with 50.9% in the Control cohort (*P* < 0.01 for all comparisons to the Control cohort). Patients in all three cohorts with the suspected genetic disease generally had higher rates of comorbidities, including respiratory failure (up to three times higher), asphyxia (up to 13 times higher), feeding problems (up to 10 times higher), and pneumonia (up to 2.5 times higher) (Supplementary Table 2). In the Broad cohort, the most common index clinical presentation was congenital anomalies representing 35.5% of patients (Table 1). Other common presentations were: endocrine, nutritional, metabolic (9.9%); mental/behavioral/neurodevelopmental (3.7%); and nervous system (2.4%). In the MCA/ID/DD/E-specific cohort, 52.8% had MCA, 32.9% nervous system diagnoses, and 7.8% with mental, behavioral, or neurodevelopmental disorders. Finally, in the Conservative cohort (in which patients were required to have an ICD-9/10 code indicative of a potential genetic disorder and must have received at least 1 genetic test), the most common clinical presentations were: congenital anomalies (21.0%), endocrine, nutritional, metabolic (15.2%), and mental, behavioral, and neurodevelopmental (11.8%).

In the critically-ill newborn population, the proportion of male patients was 57.7%, 60.6%, and 57.7% in the Broad, MCA/ID/DD/E, and Conservative populations, respectively, compared with 49.7% in the control cohort (*P* < 0.01 for all comparisons; Table 1). As in the pediatric population, critically-ill newborns generally had higher rates of comorbidities, including respiratory failure (up to five times higher), feeding problems (up to six times higher), lung contusion (up to 54 times higher), asphyxia (up to 17 times higher), pneumonia (up to four times higher), septicemia (up to 13 times higher), and shock (up to 96 times higher) (Supplemental Table 2). In the Broad cohort, Congenital anomalies were the most common clinical presentation, representing 62.7% of patients (Table 1). Other common presentations were: conditions presenting in the perinatal period (e.g., abnormal neonatal screening, some cardiac conditions, hypotonia, and others; 11.9%); and endocrine/metabolic (5.8%). In the subset of the population with MCA/ID/DD/E, the vast majority (86.4%) were MCA. The absence of ID/DD was expected, although the proportion with neurological presentations such as hypotonia, epilepsy, or seizure disorders was low (2.2%). Finally, in the Conservative population, the most common clinical presentations were congenital anomalies (51.6%), conditions presenting in the perinatal period (10.6%), and endocrine, nutritional, and metabolic conditions (10.0%). As above, these represent a population-based distribution of what types of patients have a suspicious ICD-9 or 10 code and received genetic testing.

### Intensive care utilization

In the pediatric populations, we observed higher rates of both neonatal intensive care unit (NICU) and pediatric intensive care unit (PICU) utilization in all three cohorts with indicators of suspected genetic disease compared with the control population (Table 2). For example, PICU utilization was ~16-fold higher in the Broad cohort and ~50-fold higher in the MCA/ID/DD/E and Conservative cohorts.

**Table 2:** Utilization.

	Critically-ill newborn population				Pediatric population			
	Control	Broad	MCA/ID/DD/E	Conservative	Control	Broad	MCA/ID/DD/E	Conservative
Sample size	1,148,695	29,348	8723	1924	11,036,263	1,139,035	94,714	28,348
*Genetic testing post index*								
Fraction with genetic test, %	0	7.6	8.4	100	0	4.22	5.4	100
Mean days to genetic test, 1+	N/A	144	247	163	N/A	281	473	340
Number of genetic tests, 1+								
Mean	N/A	1.7	2	1.8	N/A	2.1	2.6	2.3
SD	N/A	1.6	1.8	1.6	N/A	1.7	2.0	1.9
Median	N/A	1.0	1.0	1.0	N/A	2.0	2.0	2.0
Min	N/A	1.0	1.0	1.0	N/A	1.0	1.0	1.0
Max	N/A	23.0	22.0	23.0	N/A	25.0	18.0	26.0
*Intensive care stays*								
Fraction with NICU stay during eligibility, %	3.1	100	100	100	0.3	1.0	1.9	1.1
Fraction with PICU stay during eligibility, %	0.3	19.4	32.3	30.6	0.1	1.6	5.7	6.1
*NICU days among those with stay*								
Mean	10.1	24.6^a^	31.6^a^	26.0^a^	10.1	24.7	30.5^a^	24.9^a^
SD	12.1	33.1	38.8	34.3	12.1	36.3	41.0	40.1
Median	6.0	13.0	17.0	14.0	6.0	12.0	17.0	12.0
Min	1.0	1.0	1.0	1.0	1.0	1.0	1.0	1.0
Max	383.0	807.0	458.0	401.0	383.0	372.0	372.0	218.0

MCA/ID/DD/E: *MCA* multiple congenital anomalies, *ID* intellectual disability, *DD* developmental delay, *E* epilepsy/seizures, *NICU* neonatal intensive care unit, *PICU* pediatric intensive care unit.

^a^*P* < 0.001 vs control.

In the critically-ill newborn populations, mean NICU length of stay (LOS) was 24.6d, 31.6d, and 26.0d in the Broad, MCA/ID/DD/E, and Conservative populations, respectively, compared with 10.1d for patients in the control population that had at least one NICU stay (*P* < 0.01; Table 2; Supplementary Fig. 1). Mean NICU LOS was relatively consistent across different sub-populations of the Broad cohort based on ICD-9/10 code (Supplementary Table 3). In addition, we observed high rates of PICU utilization during the continuous enrollment period—19.4%, 32.3%, and 30.6% in the Broad, MCA/ID/DD/E, and Conservative populations, respectively—compared with 0.3% in the overall newborn control population (Table 2).

### Genetic test utilization

**Fig. 1:**
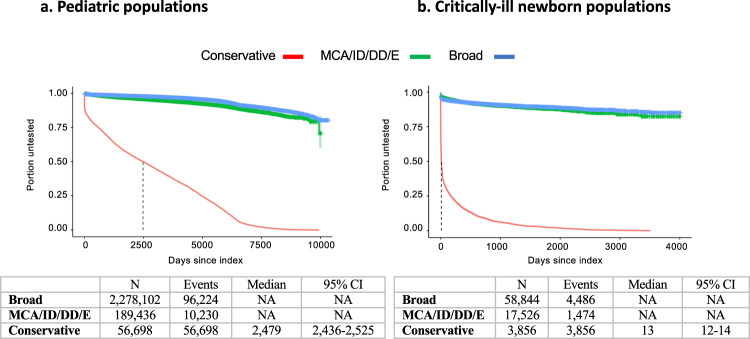
Time to first genetic test. **a** Pediatric populations; **b** critically-ill newborn populations. MCA/ID/DD/E: *MCA* multiple congenital anomalies, *ID* intellectual disability, *DD* developmental delay, *E* epilepsy/seizures.

In the pediatric populations, only 4.2% of the Broad cohort and 5.4% of the MCA/ID/DD/E cohort received at least one genetic test. In patients with at least one genetic test, the mean number of tests was 2.1, 2.6, and 2.3 in the Broad, MCA/ID/DD/E, and conservative cohorts, respectively (Table 2; see also Supplementary Fig. 2 for the distribution of a number of genetic tests). The mean time to the first test of 281 days and 473 days, respectively. Notably, in the subset of patients with moderate or severe ID, only 102 patients (~2%) had microarray testing and 9 patients had whole-exome sequencing. In the Conservative cohort, all of whom had at least one genetic test by definition, the mean time to the first test was 340 days and the median was ~7 years (2479 days) (Table 2**;** Fig. 1A).

We further interrogated genetic test utilization in the four most prevalent sub-populations of the pediatric Broad cohort (Supplementary Table 3). Genetic test utilization was low (<8%) across all sub-populations: 7.1% in patients with ICD-9/10 codes representing mental, behavioral, and neurodevelopmental disorders, 3.9% of patients with endocrine/nutritional/metabolic disorders, 3.5% with nervous system disorders, and 1.7% of patients with congenital anomalies.

Among critically-ill newborns, in the Broad cohort, only 7.6% had at least one genetic test. In patients with at least one genetic test, the mean number of tests was 1.7, 2.0, and 1.8 in the Broad, MCA/ID/DD/E, and conservative cohorts, respectively (Table 2; see also Supplementary Fig. 2 for the distribution of a number of genetic tests). The mean time to first test was 144 days. In the MCA/ID/DD/E cohort (predominantly MCA, as noted above), 8.4% had at least one genetic test with a mean time to the first genetic test of 247 days (Table 2). In the Conservative cohort, all of whom had at least one genetic test by definition, the mean time to the first test was 163 days (Table 2); however, more than half of these patients were tested in the first 2 weeks, whereas the remainder received tests over the remaining follow-up time (Fig. 1B).

In the analysis of the four most prevalent sub-populations of the critically-ill newborn broad cohort, genetic test utilization was highest in patients with blood disorders (24.2%), followed by endocrine/nutritional/metabolic disorders (14.7%), congenital anomalies (5.1%), and disorders presenting in the perinatal period (3.4%).

### Diagnostic transitions

Not all genetic diseases have specific ICD-9/10 codes; thus, a definitive assessment of genetic disease diagnoses in patients with initial clinical presentations that are indicative of a suspected genetic disease is not possible from an administrative claims-based analysis. However, assessing the proportion of patients transitioning from an initial index diagnosis code in the *possible* or *probable* genetic disease categories to a primary diagnosis code in the *definite* genetic disease category is indicative of a conservative estimate of definitive genetic disease diagnoses. These transitions may have resulted from additional clinical investigations including specialist assessments, the results of imaging or other non-invasive procedures, or, in an extreme minority of cases per the genetic test utilization results above, molecular testing. The number of patients who moved into more definite categories of diagnosis is reported in Table 3.

**Table 3:** Diagnostic transitions.

	Critically-ill newborn population			Pediatric population		
	Broad	MCA/ID/DD/E	Conservative	Broad	MCA/ID/DD/E	Conservative
Sample size	29,348	8,723	1,924	1,139,035	94,714	28,348
Fraction possible to probable	9.2	17.7	25.9	3.2	7.7	16.6
Fraction possible to definite	4.6	6.1	29	0.9	2.0	12.4
Fraction probable to definite	0.7	0.4	12.6	0.2	0.6	3.4
Fraction genetic test to definite	0.0	0.0	3.3	0.1	0.0	3.3
Fraction CPT/HCPCS to definite	0.0	0.0	0.0	0.2	0.0	0.0

The first diagnostic category represents the category of code used for the index event. The second diagnostic category represents a change from a less-specific category to a more specific category during follow-up.

*CPT/HCPCS* Current Procedural terminology/Healthcare common Procedure Coding System (HCPCS), MCA/ID/DD/E: *MCA* multiple congenital anomalies, *ID* intellectual disability, *DD* developmental delay, *E* epilepsy/seizures.

In the Broad and MCA/ID/DD/E cohorts of the pediatric population, 1.1% and 2.6% of patients, respectively, transitioned from *possible* or *probable* to *definite* (Table 3). We observed a higher transition rate in the Conservative cohort—which had at least one genetic test by definition—where 15.8% of patients transitioned from a *possible* or *probable* diagnosis to a *definite* diagnosis within the follow-up period (Table 3).

In the Broad and MCA/ID/DD/E cohorts of the critically-ill newborn population, 5.3% and 6.5% of patients, respectively, transitioned from *possible* or *probable* to *definite* (Table 3). As in the pediatric population, the Conservative cohort had a much higher rate of conversion to *definite* compared to the other two cohorts: 41.6% of patients transitioned from a *possible* or *probable* index diagnosis to a definite genetic disease diagnosis during the follow-up period.

### Costs

**Fig. 2:**
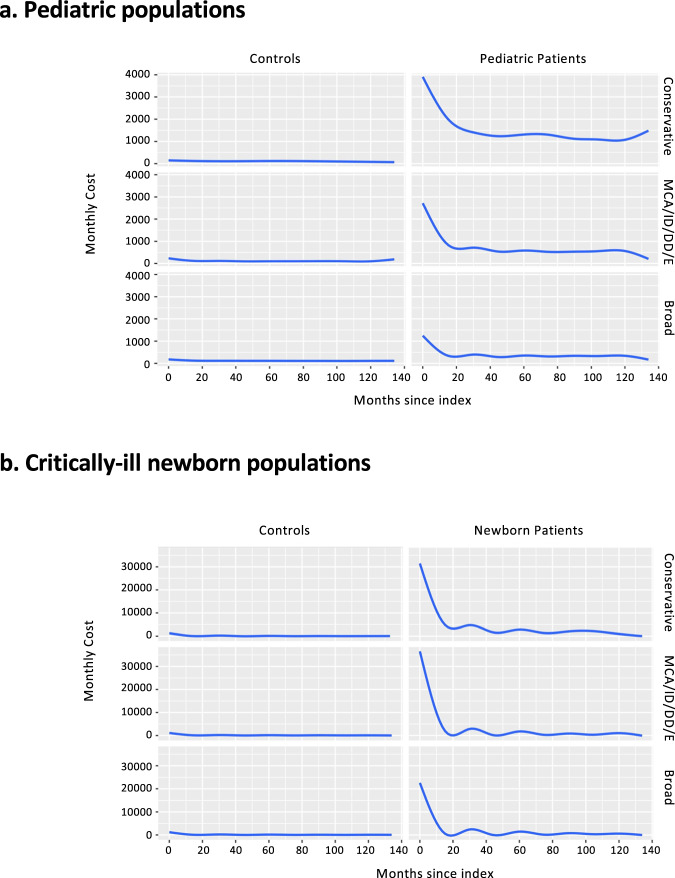
Monthly costs from index date. For both the **a** pediatric populations and **b** critically-ill newborn populations, control populations are shown on the left and patient cohorts on the right. MCA/ID/DD/E: *MCA* multiple congenital anomalies, *ID* intellectual disability, *DD* developmental delay, *E* epilepsy/seizures.

Analyses of total healthcare costs in the pediatric populations are shown in Fig. 2A and Supplementary Table 4. In all three pediatric cohorts, patients had substantially higher healthcare costs early in the observation compared with the matched control populations. For example, mean Year 1 costs were highest in the conservative cohort ($23,514), followed by the MCA/ID/DD/E cohort ($14,339) and the Broad cohort ($6256), whereas mean Year 1 costs in the matched control groups were $1736, $2261, and $1941, respectively (Supplementary Table 4). Costs declined over time in all three cohorts, reaching a steady-state after ~12–24 months post index.

A similar pattern emerged in the critically-ill newborn population (Fig. 2B and Supplementary Table 4), with higher costs in the three cohorts of patients with indicators of suspected genetic disease compared with the control population. Mean Year 1 costs were highest in the MCA/ID/DD/E cohort ($137,590), followed by the Conservative cohort ($131,392) and the Broad cohort ($80,757), whereas mean Year 1 costs in the matched control groups were $6753, $7268, and $7205, respectively (Supplementary Table 4). In all three cohorts, monthly costs declined over time, reaching a steady-state after ~12 months post index.

## Discussion

Our study aimed to identify and characterize patients with clinical indicators of genetic disease in a large US payer population. There are several key findings from our study. First, patients with clinical indicators of genetic diseases represent a substantial cohort of patients collectively. Our Broad classification—which was intended to be broad and err on the side of over-identification—represented 9.4% of the total eligible pediatric patient population—similar to the 14% of patients identified in the previously reported study using the HCUP KID hospitalization database^24^. However, even the subset of patients with a select group of clinical presentations—MCA, moderate or severe intellectual disability, developmental disorders, and epilepsy—collectively represented 0.8% of pediatric patients. While definitive epidemiological estimates are challenging to develop in the context of rare genetic diseases, our estimates are complementary to published estimates of the prevalence of rare diseases more broadly in the range of 2–6.2%^2,25–27^. In the critically-ill newborn population, our broad classification represented 44.7% of patients with a NICU stay and the subset with MCA or epilepsy/seizures represented 13.3% of critically-ill newborn patients—approximately one of seven patients. Our estimate that approximately half of the NICU population in this large real-world data set has indications of a genetic disease is similar to at least one other estimate based on recruitment for clinical study participation^28^. Thus, critically-ill infants represent an enriched population where the concern for an underlying genetic disease is very high for a substantial fraction of patients.

A second major takeaway from our study was the significant underutilization of genetic testing and associated delays in genetic testing. In the Broad population of pediatric patients, only 4.2% of patients had even one genetic test, and the first test was performed an average of 281 days from the first clinical presentation. Although the broad population was intended to be broad and we would not expect all patients to require genetic testing, even in the specific cohort of patients with MCA/ID/DD/E—clinical presentations that should indicate for genetic testing—only 1 in 20 patients had even one genetic test and those tests were delayed from the first clinical presentation by an average of 15 months. Utilization of specific genetic tests such as chromosomal microarrays and ES, which are recommended and increasingly covered by insurance, were also extremely rare—even with the caveat that the timeframe of analysis (Jan 2007 to Dec 2017) extends to a time period prior to recommendations and broad coverage. Similar to the pediatric population, only a small proportion of critically-ill newborns with MCA or epilepsy/seizures—~8%—received even one genetic test in the entire follow-up period. Although not directly addressed in our study, the consequences of delaying diagnosis of genetic diseases among newborns and children can be devastating, resulting in missed opportunities for effective treatment, unnecessary procedures, excessive costs, in addition to patient and family anxiety^12,15,21,22,29–31^. Future research should evaluate the reasons underlying the low genetic test utilization rates—which likely include clinician and patient/family education, socioeconomic barriers, and insurance hurdles^32–41^, the potential impact on patient outcomes, and novel or innovative methods to address barriers and inequities in access.

A third major takeaway was that critically-ill newborns with indicators of suspected genetic diseases had long NICU stays compared with a control group of newborns without evidence of a suspected genetic condition. In addition, both the critically-ill newborn and pediatric populations had high rates of PICU utilization. Several studies have demonstrated that rapid exome or genome approaches may lead to decreases in NICU LOS in a subset of patients^12,22,42^. Although our study design does not allow for an assessment of whether application of earlier diagnostic testing or more rapid diagnosis would have resulted in a decrease in the average LOS in the NICU, identifying the underlying clinical diagnosis would accelerate the ability to institute appropriate clinical management in this acutely ill population^3,12,22,43,44^.

Finally, patients with clinical indicators of genetic disease in all of the cohorts evaluated had substantially higher costs compared with controls–particularly in the first 12–24 months post index. These data build on a previous study focused on costs associated with pediatric hospitalizations^24^. This area of research deserves further exploration. Notably, from a health economic perspective, several published studies demonstrate that earlier incorporation of genome-wide approaches such as exome or genome sequencing is cost-effective by preventing long expensive diagnostic odysseys^21,22,45,46^. A recent study in the Netherlands found that medical costs decreased substantially following ES—regardless of whether there was a diagnosis—a finding they termed an “end of trajectory” effect^47^. Future studies should interrogate the services contributing to costs and how the mix of services changes over time.

Our study has strengths and limitations. Strengths include the size of the population evaluated—which included over 13 million unique patients from a large commercial health plan population, representative of commercially-insured patients more generally. Our methodology, which included a detailed curation of indicators of genetic disease, allowed us to evaluate several different populations of patients based on different levels of specificity. Finally, our administrative claims-based approach allowed for a comprehensive assessment of direct healthcare-related utilization and costs. Several limitations should be considered in the interpretation of our results. First, as our methodology utilized insurance claims data as opposed to electronic health records, we are limited in our ability to interpret both diagnostic thinking and clinical decision-making. For example, while we observe substantial delays in genetic testing in many patients, the reasons for these delays are unclear and may be clinically warranted based on the unique circumstances of individual patients. Similarly, ICD-9/10 codes do not capture the granularity required to distinguish diagnosed genetic conditions. Human Phenotype Ontology (HPO) provides a deep ontology that relates detailed clinical features to both diseases and genes. Availability of HPO terms in a large data set like this would have improved our specificity in defining populations and HPO coding (or improvements in ICD coding informed by HPO) could contribute to future studies of genetic disease in populations. In addition, while some genetic diseases have specific ICD-9/10 codes, many do not; thus, we are unable to definitively determine the end of a diagnostic odyssey for many individual patients. Second, our definitions of possible, probable and definite genetic disease are based on expert opinion and manual curation. Not all patients with these features would have had a genetic diagnosis even if optimal testing had been performed. This is likely due to additional undescribed disease-causing genes, oligogenic and complex inheritance, and environmental phenocopies. How ICD-9/10 codes are used for insurance billing purposes may not be aligned with the diagnostic thinking of clinicians. Coding systems, including diagnosis and procedure codes, are used for billing purposes and are likely to fail to reflect the variability observed in clinical practice. Furthermore, a level of miscoding is presumed to occur as is inherent in claims database studies (e.g., a coding specialist may select the first code on a list of possible codes as opposed to the most appropriate code). Third, given the substantial health conditions of patients with genetic diseases, patients may transition to public insurance programs, switch between private insurance plans, or for other reasons stopped being observed in the database. Although our continuous enrollment requirements help control for some of this variability, there are inherent limitations to the comprehensiveness of the follow-up, which could also be exacerbated by the severity of the disease. Finally, in our selection of patients, we found a large number of newborns with codes indicating a possible genetic condition but no stay in a NICU. Because we required a stay in a NICU, these patients were excluded from analysis in the critically-ill infant population and were instead included in the pediatric population.

The results of our study highlight the substantial patient population with clinical features that may lead a physician to suspect genetic disease, and notably, that there is significant underutilization of genetic testing. Earlier and more comprehensive genomic testing has the potential to reduce the burden on children, families, and the healthcare system. As the availability of new therapies and approaches expands, accurate and rapid diagnosis becomes even more critical. Integrating novel diagnostic approaches early in the life of children with clinical symptoms will improve the long-term prospects of these children and reduce unnecessary costs to the healthcare system.

## Methods

### Data source

We performed a retrospective administrative claims analysis using Optum’s de-identified Clinformatics® Data Mart Database 7.1 database which contains linked eligibility, medical claims, and pharmacy claims. Data for all patients 0–18 years of age were used to identify patients with clinical features that may lead a physician to suspect genetic disease based on the date of birth between 1 January 2007 through 31 December 2017. Age was determined by the patient’s earliest eligibility effective date. To protect patient privacy, actual dates of service were not provided in the data; instead, the database contained “derived dates”, which indicated the days since birth that an event occurred. For example, if a date was 0 this would be the day the patient was born. If the date was 365, this would indicate that the event was 1-year post birth. Data were provided in de-identified format and met all requirements for patient privacy (i.e., names, exact birth dates, health plan identifiers, and zip codes were not provided as part of the data set).

### Ethics

Institutional review board approval was not required as the Optum database has been evaluated and approved in compliance with HIPAA standards for data privacy.

### Study population

Our methodology to define the study populations was developed with the goal of identifying pediatric (<18 years of age) patients with indicators of genetic diseases. Diagnostic codes typically do not classify by underlying disease mechanism, and we therefore manually curated lists of International Statistical Classification of Diseases and Related Health Problems (ICD)-9 and ICD-10 diagnosis codes, Current Procedural Technology (CPT), and Healthcare Common Procedure Coding System (HCPCS) codes, and other relevant billing codes (e.g., neonatal intensive care). A detailed list of all codes used in this study can be found in the Supplementary Information. We then used combinations of these codes (see below) to define patient subsets for analysis. These code sets were initially curated by an expert medical geneticist and were reviewed by claims coding experts at Optum. A similar curation methodology for ICD-9 codes has been previously described^24^, though the current study the code sets were expanded to allow for more detailed classification of patient cohorts and utilization of genetic and non-genetic testing and procedures (by including CPT/HCPCS codes) and to allow for additional timeframe analyses (by adding ICD-10 codes). The code curation process resulted in identification of 1269 ICD-9 codes, 2155 ICD-10 codes, and 781 CPT/HCPCS codes. These codes were further curated with respect to the age of presentation as it is well known that some clinical diagnoses are highly suggestive of a genetic disorder within certain age groups but not others (e.g., isolated scoliosis in a newborn may indicate a genetic disorder, but the same feature in an adolescent is more likely to be multifactorial).

By definition, ICD-9/10 codes are intended to classify diseases or conditions; thus, some ICD-9/10 codes definitively identify specific genetic diseases whereas others indicate clinical presentations that may be an accepted indication to test for a genetic disease. As such, as described previously^24^, ICD-9/10 codes were first categorized into three sets: (1) *definite* genetic disease; (2) *probable* genetic disease; and (3) *possible* genetic disease. The definite category was defined by ICD-9/10 codes that identify specific genetic diseases (e.g., cystic fibrosis, Down syndrome, specific inborn errors of metabolism). Probable genetic diseases were defined as ICD-9/10 codes that describe clinical presentations wherein a genetic etiology is likely to be achieved with appropriate genetic testing in >30% of patients with these codes (e.g., neonatal intractable epilepsy). Finally, possible genetic diseases were defined as ICD-9/10 codes that describe clinical presentations wherein a genetic etiology is likely to be achieved with appropriate testing in ~10–30% of patients (e.g., most severe birth defects, such as congenital central nervous system, and cardiac malformations). We further identified ICD-9/10 codes where there are established and specific clinical and/or laboratory diagnostic criteria and availability of established and commonly administered targeted diagnostic tests (e.g., cystic fibrosis, sickle cell disorders, neurofibromatosis), and therefore patients with these codes would be less likely to present clinically as an undiagnosed genetic disease. CPT and HCPCS codes were categorized into two categories: (1) genetic tests (e.g., specific single-gene tests, molecular pathology, chromosomal microarray (CMA), ES); and (2) other procedures that are commonly used in the workup of patients with suspected genetic diseases (e.g., electrophysiologic tests, imaging, skin or muscle biopsies, etc), though we acknowledge this second category includes codes that are not specific to the workup of patients with suspected genetic diseases.

We used combinations of this curated code sets to group patients into three non-mutually exclusive cohorts for analysis [a complete list of codes and the algorithms for identifying each cohort (Supplementary Information) and an attrition table (Supplementary Table 1) can be found in the Supplementary Information].

#### Broad

The Broad cohort was intended to be the broadest cohort of patients. We defined this cohort as patients with a *possible*, *probable*, or *definite* ICD-9/10 code (excluding codes considered easy to diagnose, per the description above) or ≥1 curated genetic or non-genetic CPT/HCPCS code.

#### Conservative

The Conservative cohort was intended to be a specific cohort of patients with indicators of undiagnosed disease and evidence of genetic testing. We defined this cohort as patients with a *possible* or *probable* ICD-9/10 diagnosis code as the index code and ≥1 genetic test CPT/HCPCS code at any time. Thus, patients with *definite* ICD-9/10 codes as the index code were excluded.

#### Subset of clinical indications broadly accepted for genetic testing (MCA/ID/DD/E)

This cohort was intended to identify a specific subset of clinical presentations that are broadly accepted indications for genetic testing, as suggested by clinical guidelines^48–54^. We defined this cohort as patients with ICD-9/10 diagnosis codes for MCA, moderate or severe ID, DD, or E. For patients with MCA, patients were required to have either a code specific for multiple anomalies or codes for single anomalies in two or more organ systems.

An index date was set on the service date of the first appearing qualifying code for a diagnosis for the Conservative and MCA/ID/DD/E cohorts, or on the first appearing diagnosis or test date for the Broad cohort. Identification of the index date included all ICD codes, regardless of whether the code was principal or secondary, with a hierarchy of definite>probable>possible for the index code for claims with more than one associated diagnosis code. Patients were required to be continuously enrolled for 6 months prior to the index diagnosis, with any child <6 months of age having a reduced continuous enrollment requirement equal to their age on index.

**Fig. 3:**
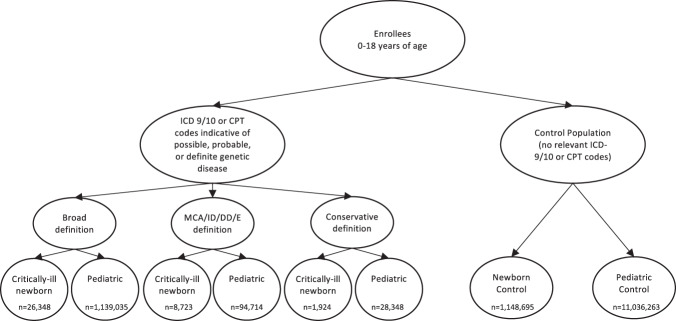
Population selection process. Description of cohort identification process. Note that groups are not mutually exclusive (e.g., MCA/ID/DD/E and Conservative are subsets of Broad). MCA/ID/DD/E: *MCA* multiple congenital anomalies, *ID* intellectual disability, *DD* developmental delay, *E* epilepsy/seizures. *CPT/HCPCS* Current Procedural terminology/ Healthcare common Procedure Coding System (HCPCS).

Finally, we divided the sample into two non-overlapping populations based on age and patient acuity: pediatric patients and critically-ill infants. Pediatric patients were either >28 days of age and ≤18 years on the index date, or were ≤28 days of age on the index date but had no observed NICU stay at any time during the observation period. Critically-ill newborns were ≤28 days of age on the index date and had a stay in a NICU at any time during the observation period. Thus our final analyses were completed on six groups: within both critically-ill infants and within pediatric patients we evaluated Broad, Conservative, and MCA/ID/DD/E cohorts. (Fig. 3)

### Control population

We defined control populations with none of the *possible, definite*, or *probable* ICD-9/10 diagnosis or CPT codes used to select the study populations. The first continuous enrollment period for control was identified and the index date was set as of the start date of enrollment. For descriptive analyses, all subjects were included. Each of the six cohorts (the three definition categories for each of the two patient age groups) were matched to a control population generated by the use of propensity score technique matching on ethnicity/race, gender, Census Division, age at index (pediatric only), year of birth, and a number of children in subscriber’s household. A NICU stay was not required for inclusion in the newborn control cohort. One way of measuring the balance is with a Multivariate Imbalance Measure. In this context, an L1 of 1.0 means completely unmatched, and an L1 of 0.0 means perfectly matched. We then used three procedures for matching to determine the best methodological approach to achieve balanced cohorts. For the evaluation of survival, controls were matched using the Coarsened Exact Matching (CEM) method^55^ with k-to-k (k2k)—i.e., where the matching procedure reduces the samples to a 1 to 1 match rather than many to 1 match. The CEM with k2k is almost as good as CEM without k2k, but has the computational benefit of reducing the number of records in the control population, so these results were used for all matching procedures.

### Variable definitions

Variables assessed included clinical presentation, demographics (including age, ethnicity/race, gender, geography, poverty status, and comorbidities), genetic test utilization, time in the NICU and PICU, and overall costs. Clinical presentations and comorbidities were identified based on ICD-9/10 diagnosis codes as described above and in the Supplementary Information. NICU and PICU stays were identified through revenue codes for billing of neonatal intensive care room and board billing. Genetic test utilization was identified based on CPT/HCPCS codes. Costs were measured during the continuous enrollment period in 30-day increments starting with the index date according to the methodology described by Lin et al.^56^.

All medical and pharmacy claims were used in the calculation of costs using the Optum database standard allowable charges field. Costs were calculated as total costs (inclusive of medical and pharmacy costs), inpatient costs (identified from the inpatient confinement claims), and outpatient costs (from the medical claims). More information on variable definitions and data limitations can be found in Supplementary Notes1 and 2.

### Analytic approach

Statistical comparisons to the control group were conducted for demographic variables, including age at index, length of enrollment, age at enrollment, % male, and % white using independent samples, two-sided, *t* test. Other primary analyses performed were descriptive—to avoid introducing the possibility of error based on multiple hypothesis testing.

### Reporting summary

Further information on research design is available in the Nature Research Reporting Summary linked to this article.

## Supplementary information

Supplementary Information

Reporting Summary

## Data Availability

The data evaluated in this study was obtained from OptumInsight (https://www.optum.com/) under a data license agreement. Due to the expiration of the data set license, the data sets generated during and/or analyzed during the current study may not be available in full, but tables of output may be available on reasonable request.

## Code availability

The analyses performed were descriptive and did not use any custom code or mathematical algorithm that is deemed central to the conclusions. A detailed list of all curated codes used in this study to group patients for descriptive analyses is provided in the Supplementary Information.
