# Supplementary material for: The diagnostic trajectory of infants and children with clinical features of genetic disease
Source: NPJ Genom Med. 2021 Nov 22;6:98. doi: 10.1038/s41525-021-00260-2 (PMC8609026; doi:10.1038/s41525-021-00260-2)
Supplement: Supplementary file 1 — Supplementary Information [file 41525_2021_260_MOESM1_ESM.pdf]

The Diagnostic Trajectory of Infants and Children with Clinical Features of Genetic Disease

Supplementary Information

Supplementary Note 1: Definitions and Variable Creation

The following details apply to the study variable definitions.

NICU stays

We were unable to determine specific admission and discharge dates for NICU stays. Therefore, NICU use during any time of an inpatient stay was identified based on revenue and procedure codes. Length of stay was calculated for the entire inpatient stay with NICU use.

Genetic testing

Genetic testing was identified based on the presence of procedure codes during the period of continuous enrollment. The following measures were calculated:

- Date of first appearing genetic test
- Date of last appearing genetic test
- Number of unique genetic tests
- Number of distinct days in which a genetic test was given

Comorbidities

Comorbidities were identified from both primary and secondary diagnosis codes on medical claims. A table of ICD9 codes was constructed based on prior research[1] and mapped to current ICD10 codes using the CMS General Equivalence Mappings (GEMs). In the original source paper several codes were incorrectly listed. ICD-9 codes were correct as follows:

- Pyrexia: 672
- Septicemia: 041.11; 041.8; 041.0; 041.4; 038.9; 038.1; 041.7; 041.3; 041.5; 038.0; 996.6
- Gastroenteritis: 008.6; 558; 558.0
- head injury: 800.1; 801.1; 852.0; 348.8; 854.0; 853.0; 851.0; 800.3; 348.4; 910.0

Supplementary Note 2: Data limitations

In addition to those limitations discussed in the manuscript, an additional limitation related to the actual data. First, for a very small number of patients, enrollment dates exceeded the database observation period. Second, three codes overlapped in the transition status algorithms.

H47311	COLOBOMA OF OPTIC DISC RIGHT EYE
H47312	COLOBOMA OF OPTIC DISC LEFT EYE
H47313	COLOBOMA OF OPTIC DISC BILATERAL

[1] Tai D, Dick P, To T, Wright J. Development of pediatric comorbidity prediction model. Archives of Pediatrics & Adolescent Medicine. 2006;160: 293-9.

Supplementary Table 1 Attrition Table showing the details of the sample selection process and attrition of patients from the final sample based on the application of the study criteria

	Broad Definition	MCA/ID/DD/E Definition	Conservative Definition	Unmatched Controls
# patients in enrollment table	13,076,212	13,076,212	13,076,212	13,076,212
# patients defined by population	1,838,617	164,615	50,465	11,174,373
# patients enrolled at index	1,838,592	164,615	50,465	11,174,261
# patients enrolled >180d pre index or patient <180d old at index	1,384,992	111,673	36,964	
# patients age >= 0 or age <= 6570 at index	1,168,383	103,437	30,272	11,036,263
Critically ill newborn				
# patients age 0 >= or age <= 28	155,067	28,853	7,250	1,148,695
# patients age 0 >= or age <= 28 & 1 NICU code in CE period	29,348	8,723	1,924	
Pediatric				
# patients > 28d and <= 18 years and 180d enrollment pre index; or patients <= 28 & no NICU code in CE period	1,139,035	94,714	28,348	11,036,263

Supplementary Table 2 Distribution of newborn and pediatric comorbidities

	Critically Ill Newborn Population				Pediatric Population			
	**Control**	**Broad**	**MCA/ID/DD/E**	**Conservative**	**Control**	**Wide**	**MCA/ID/DD/E**	**Conservative**
Sample Size	1,148,695	29,348	8,723	1,924	11,036,263	1,139,035	94,714	28,348
Brain Cancer	0.0044%	0.18%	0.37%	0.36%	0.016%	0.21%	0.76%	1.21%
Asphyxia	1.30%	12.76%	20.61%	21.57%	0.74%	2.96%	9.51%	9.27%
Shock	0.12%	8.04%	11.57%	11.28%	0.076%	0.48%	1.30%	2.39%
Leukemia	0.0026%	0.12%	0.16%	0.94%	0.021%	0.18%	0.24%	2.69%
Feeding Problems	6.58%	25.96%	39.52%	40.12%	1.13%	3.53%	10.20%	11.64%
Pneumonitis	0.029%	1.71%	3.54%	3.95%	0.017%	0.15%	0.75%	0.74%
Candidiasis	0.80%	1.14%	1.55%	1.46%	0.28%	0.53%	0.93%	1.25%
Head Injury	1.16%	5.24%	9.96%	9.36%	1.24%	2.56%	5.82%	6.04%
Acidosis	0.15%	4.52%	7.52%	8.68%	0.074%	0.42%	1.34%	2.12%
Hypertension	0.056%	3.17%	5.88%	7.28%	0.33%	1.17%	1.89%	3.87%
Respiratory Failure	11.04%	36.19%	51.20%	45.69%	7.32%	12.11%	21.50%	22.02%
Lung Contusion	0.41%	11.74%	21.09%	21.62%	0.38%	1.28%	3.71%	4.62%
Septicemia	1.03%	8.65%	13.88%	12.84%	0.85%	2.00%	3.50%	5.68%
Pyrexia	0.0015%	0.0068%	0.00%	0.00%	0.0025%	0.0068%	0.0042%	0.025%
Pneumonia	4.39%	11.15%	17.17%	15.54%	3.69%	4.95%	8.97%	9.01%
Femur Fracture	0.11%	0.29%	0.50%	0.62%	0.34%	0.42%	0.52%	0.58%

Supplementary Table 3 Utilization in Subsets of Broad Population Based on ICD-9/10 Category

	Critically Ill Newborn Population				Pediatric Population			
	Congenital anomalies	Perinatal period	Endocrine/ Nutritional/ Metabolic	Blood disorders	Congenital anomalies	Endocrine/ Nutritional/ Metabolic	Mental/ Behavioral/ Neuro-developmental	Nervous system
Sample Size	18,414	3,497	1,705	756	404,034	113,145	42233	26,878
% of Broad Category	62.7	11.9	5.8	2.6	35.5	9.9	3.7	2.4
Genetic testing post-index								
Fraction with genetic test, %	5.1	3.4	14.7	24.2	1.7	3.9	7.1	3.5
Number of genetic tests., 1+								
Mean	2.1	1.4	1.5	1.4	2.4	1.8	2.7	2.8
SD	1.9	1.2	1.6	0.9	1.9	1.6	1.8	2.4
Median	1.0	1.0	1.0	1.0	2.0	1.0	2.0	2.0
Min	1.0	1.0	1.0	1.0	1.0	1.0	1.0	1.0
Max	23.0	11.0	22.0	7.0	21.0	21.0	23.0	17.0
Intensive care stays								
Fraction with NICU stay during eligibility, %	100	100	100	100	1.6	0.3	0.5	0.7
Fraction with PICU stay during eligibility, %	21.3	13.9	13.6	19.8	1.9	1.2	0.7	5.3
Mean NICU days among those with a stay								
Mean	25.8	22.2	23.0	27.2	24.1	23.8	68.5	29.8
SD	34.3	30.4	29.1	36.7	34.9	24.2	118.4	41.1
Median	13.0	11.0	13.0	14.0	13.0	15.0	16.0	12.0
Min	1.0	1.0	1.0	1.0	1.0	1.0	4.0	1.0
Max	807.0	415.0	400.0	315.0	372.0	114.0	246.0	165.0

Supplementary Table 4 Total cost by year (from index date)

	Critically Ill Newborn Population					
	Broad	Broad Control	MCA/ID/DD/E	MCA/ID/DD/E Control	Conservative	Conservative Control
Year 1	$80,757	$7,205	$137,590	$6,753	$131,392	$7,268
Year 2	$12,596	$2,394	$19,832	$2,491	$40,061	$2,333
Year 3	$7,779	$1,323	$10,135	$1,442	$26,558	$1,722
Year 4	$6,133	$1,049	$8,329	$1,066	$17,537	$739
Year 5	$6,145	$1,268	$8,130	$1,321	$19,121	$927
Year 6	$5,219	$1,124	$6,847	$1,047	$15,691	$779
Year 7	$4,038	$881	$5,605	$830	$15,395	$752
Year 8	$3,429	$887	$4,280	$1,320	$13,692	$417
Year 9	$4,330	$705	$4,349	$732	$23,061	$467
Year 10	$3,674	$754	$6,345	$947	$14,501	$505
	Pediatric Population					
	Broad	Broad Control	MCA/ID/DD/E	MCA/ID/DD/E Control	Conservative	Conservative Control
Year 1	$6,256	$1,941	$14,339	$2,261	$23,514	$1,736
Year 2	$3,311	$1,465	$6,766	$1,493	$14,084	$1,407
Year 3	$3,017	$1,363	$5,602	$1,297	$12,394	$1,291
Year 4	$2,946	$1,365	$5,305	$1,233	$11,265	$1,282
Year 5	$3,017	$1,363	$5,046	$1,192	$11,823	$1,400
Year 6	$3,061	$1,318	$5,211	$1,267	$13,312	$1,684
Year 7	$3,104	$1,360	$4,967	$1,153	$12,519	$1,198
Year 8	$3,067	$1,271	$5,006	$1,300	$10,877	$1,296
Year 9	$3,246	$1,299	$5,431	$1,306	$12,295	$1,112
Year 10	$3,084	$1,216	$5,242	$865	$8,431	$922

Supplementary Figure 1 Total days in NICU for critically ill newborns

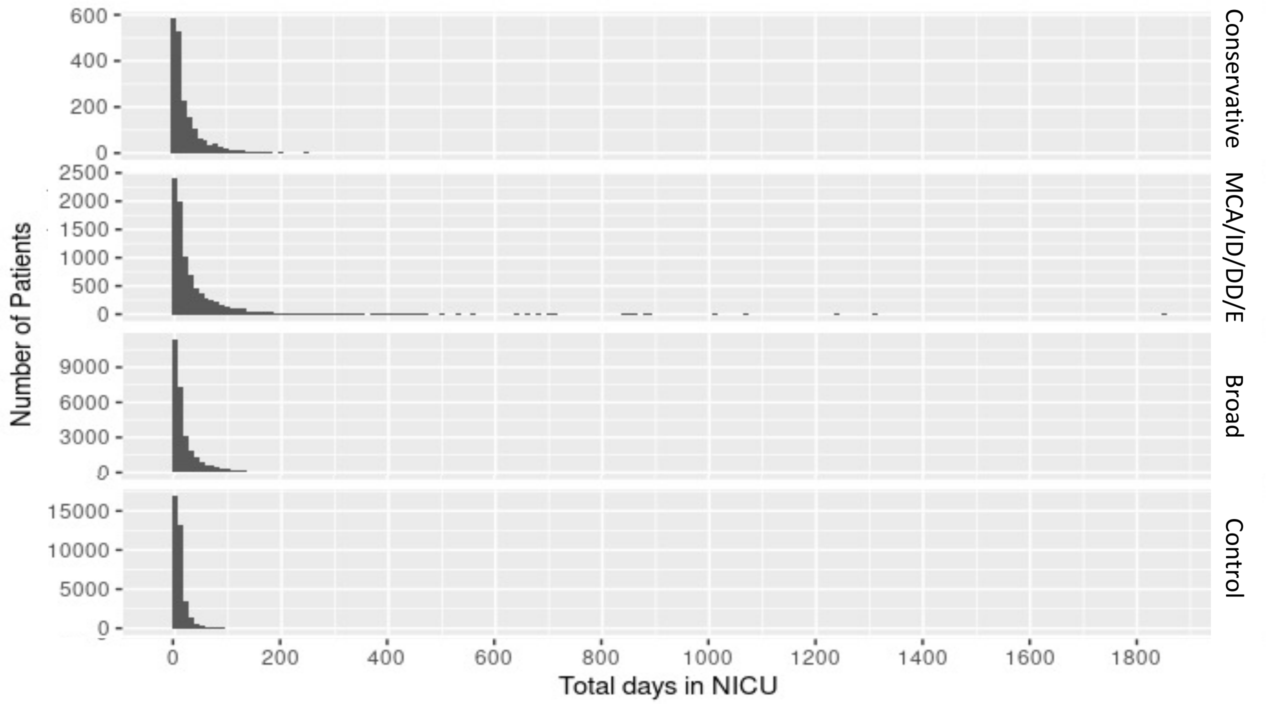

Supplementary Figure 2 Genetic test utilization in patients with at least 1 test

A. Pediatric populations

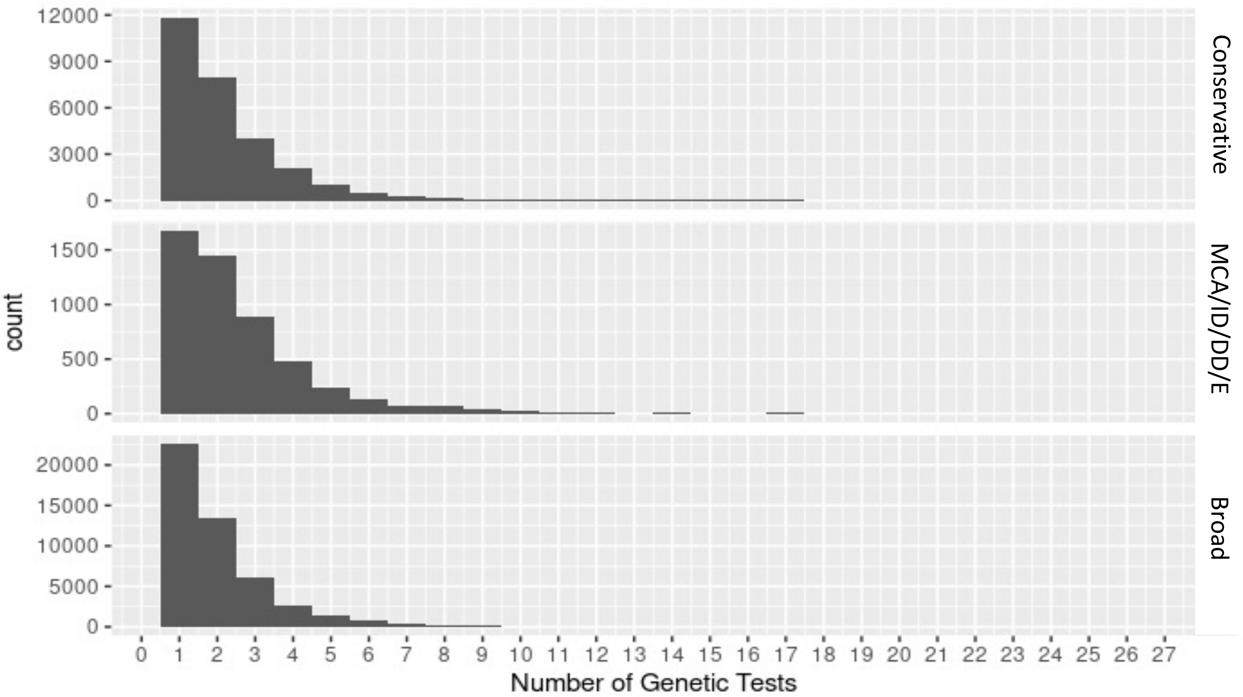

B. Critically-ill newborn populations

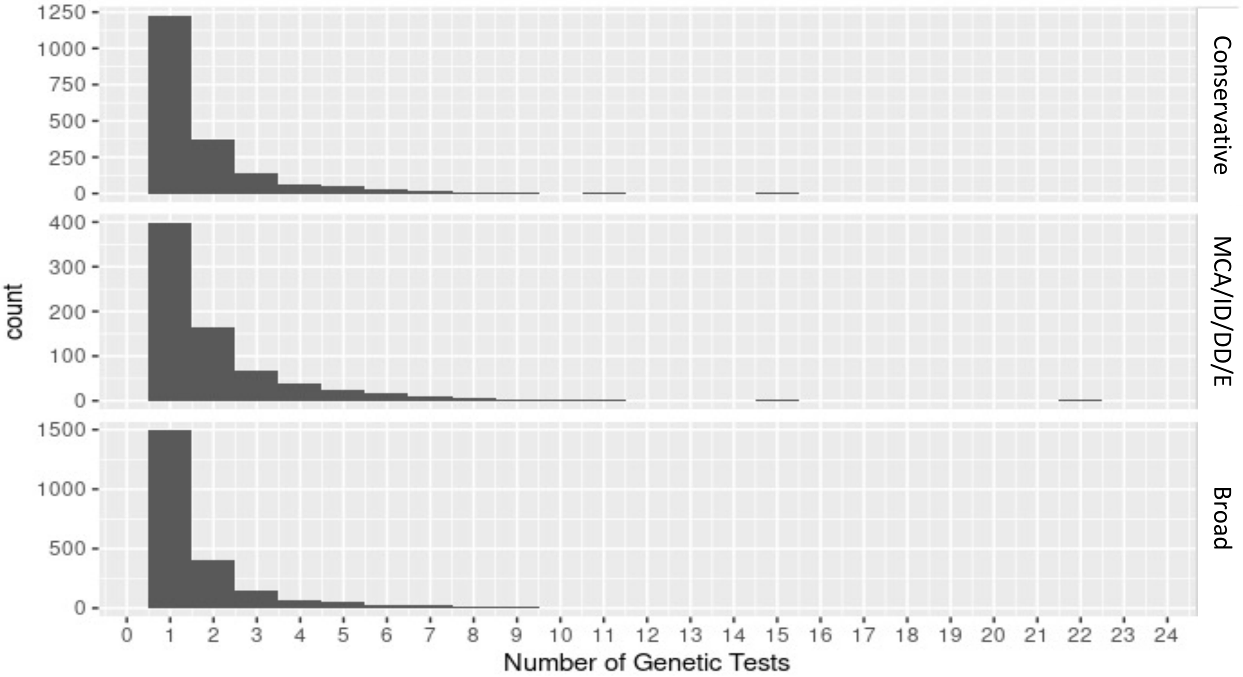

List of ICD-9, ICD-10, CPT, and HCPCS codes

ICD-9 CODE	Category Number	Category Name	Multiple Congenital Anomalies Category	Description	Targeted Testing	Newborn_Definite	Newborn_Probable	Newborn_Possible	Ped_Definite	Ped_Probable	Ped_Possible	MCA, ID,DD, Epilepsy
04611	001-139	Infectious and parasitic diseases	NA	Variant Creutzfeldt-Jakob disease	1	0	0	0	0	1	0	
04619	001-139	Infectious and parasitic diseases	NA	Other and unspecified Creutzfeldt-Jakob disease	1	0	0	0	0	1	0	
0463	001-139	Infectious and parasitic diseases	NA	Progressive multifocal leukoencephalopathy	0	0	0	0	0	1	0	
04671	001-139	Infectious and parasitic diseases	NA	Gerstmann-Straussler-Scheinker syndrome	1	0	0	0	0	1	0	
04672	001-139	Infectious and parasitic diseases	NA	Fatal familial insomnia	1	0	0	0	0	1	0	
20230	140–239	Neoplasms	NA	Malignant histiocytosis, unspecified site, extranodal a	0	0	1	0	0	0	1	
20238	140–239	Neoplasms	NA	Malignant histiocytosis, lymph nodes of multiple sites	0	0	1	0	0	1	0	
23770	140–239	Neoplasms	NA	Neurofibromatosis, unspecified	1	1	0	0	1	0	0	
23771	140–239	Neoplasms	NA	Neurofibromatosis, type 1 [von recklinghausen's dise	1	1	0	0	1	0	0	
23772	140–239	Neoplasms	NA	Neurofibromatosis, type 2 [acoustic neurofibromatosi	1	1	0	0	1	0	0	
23773	140–239	Neoplasms	NA	Schwannomatosis	1	0	0	0	0	0	1	
23779	140–239	Neoplasms	NA	Other neurofibromatosis	1	1	0	0	1	0	0	
243	240–279	Endocrine, nutritional, and metabolic di	NA	Congenital hypothyroidism	0	0	0	1	0	0	1	
2461	240–279	Endocrine, nutritional, and metabolic di	NA	Dyshormonogenic goiter	0	1	0	0	1	0	0	
250	240–279	Endocrine, nutritional, and metabolic di	NA	DIABETES MELLITUS	0	0	0	1	0	0	1	
2510	240–279	Endocrine, nutritional, and metabolic di	NA	Hypoglycemic coma	0	0	0	1	0	0	1	
2521	240–279	Endocrine, nutritional, and metabolic di	NA	Hypoparathyroidism	0	0	1	0	0	0	1	
2532	240–279	Endocrine, nutritional, and metabolic di	NA	Panhypopituitarism	0	0	0	1	0	0	1	
2533	240–279	Endocrine, nutritional, and metabolic di	NA	Pituitary dwarfism	0	0	1	0	0	1	0	
2535	240–279	Endocrine, nutritional, and metabolic di	NA	Diabetes insipidus	0	0	0	1	0	0	1	
25513	240–279	Endocrine, nutritional, and metabolic di	NA	Bartter's syndrome	0	1	0	0	1	0	0	
2552	240–279	Endocrine, nutritional, and metabolic di	NA	Adrenogenital disorders	0	1	0	0	1	0	0	
25541	240–279	Endocrine, nutritional, and metabolic di	NA	Glucocorticoid deficiency	0	0	1	0	0	0	1	
25542	240–279	Endocrine, nutritional, and metabolic di	NA	Mineralocorticoid deficiency	0	0	1	0	0	0	1	
25801	240–279	Endocrine, nutritional, and metabolic di	NA	Multiple endocrine neoplasia [MEN] type I	1	1	0	0	1	0	0	
25802	240–279	Endocrine, nutritional, and metabolic di	NA	Multiple endocrine neoplasia [MEN] type IIA	1	1	0	0	1	0	0	
25803	240–279	Endocrine, nutritional, and metabolic di	NA	Multiple endocrine neoplasia [MEN] type IIB	1	1	0	0	1	0	0	
2588	240–279	Endocrine, nutritional, and metabolic di	NA	Other specified polyglandular dysfunction	0	0	0	1	0	0	1	
2590	240–279	Endocrine, nutritional, and metabolic di	NA	Delay in sexual development and puberty, not elsewh	0	0	0	0	0	1	0	
2591	240–279	Endocrine, nutritional, and metabolic di	NA	PRECOCIOUS SEXUAL DVLP&PUBERTY NEC	0	0	0	1	0	0	1	
2594	240–279	Endocrine, nutritional, and metabolic di	NA	Dwarfism, not elsewhere classified	0	0	1	0	0	1	0	
2595	240–279	Endocrine, nutritional, and metabolic di	NA	ANDROGEN INSENSITIVITY SYNDROME	1	1	0	0	1	0	0	
25950	240–279	Endocrine, nutritional, and metabolic di	NA	Androgen insensitivity, unspecified	1	1	0	0	1	0	0	
25951	240–279	Endocrine, nutritional, and metabolic di	NA	Androgen insensitivity syndrome	1	1	0	0	1	0	0	
25952	240–279	Endocrine, nutritional, and metabolic di	NA	Partial androgen insensitivity	1	1	0	0	1	0	0	
2700	240–279	Endocrine, nutritional, and metabolic di	NA	Disturbances of amino-acid transport	0	1	0	0	1	0	0	
2701	240–279	Endocrine, nutritional, and metabolic di	NA	Phenylketonuria [PKU]	1	1	0	0	1	0	0	
2702	240–279	Endocrine, nutritional, and metabolic di	NA	Other disturbances of aromatic amino-acid metabolis	0	1	0	0	1	0	0	
2703	240–279	Endocrine, nutritional, and metabolic di	NA	Disturbances of branched-chain amino-acid metabolis	0	1	0	0	1	0	0	
2704	240–279	Endocrine, nutritional, and metabolic di	NA	Disturbances of sulphur-bearing amino-acid metaboli	0	1	0	0	1	0	0	
2705	240–279	Endocrine, nutritional, and metabolic di	NA	Disturbances of histidine metabolism	0	1	0	0	1	0	0	
2706	240–279	Endocrine, nutritional, and metabolic di	NA	Disorders of urea cycle metabolism	0	1	0	0	1	0	0	
2707	240–279	Endocrine, nutritional, and metabolic di	NA	Other disturbances of straight-chain amino-acid meta	0	1	0	0	1	0	0	
2708	240–279	Endocrine, nutritional, and metabolic di	NA	Other specified disorders of amino-acid metabolism	0	1	0	0	1	0	0	
2709	240–279	Endocrine, nutritional, and metabolic di	NA	Unspecified disorder of amino-acid metabolism	0	1	0	0	1	0	0	
271	240–279	Endocrine, nutritional, and metabolic di	NA	D/O CARBOHYDRATE TRANSPORT&METAB	0	1	0	0	1	0	0	
2710	240–279	Endocrine, nutritional, and metabolic di	NA	Glycogenosis	0	1	0	0	1	0	0	
2711	240–279	Endocrine, nutritional, and metabolic di	NA	Galactosemia	1	1	0	0	1	0	0	
2712	240–279	Endocrine, nutritional, and metabolic di	NA	Hereditary fructose intolerance	1	1	0	0	1	0	0	
2713	240–279	Endocrine, nutritional, and metabolic di	NA	Intestinal disaccharidase deficiencies and disaccharid	1	0	0	1	0	0	1	
2714	240–279	Endocrine, nutritional, and metabolic di	NA	Renal glycosuria	0	0	0	1	0	0	1	
2718	240–279	Endocrine, nutritional, and metabolic di	NA	Other specified disorders of carbohydrate transport a	0	1	0	0	1	0	0	
2719	240–279	Endocrine, nutritional, and metabolic di	NA	Unspecified disorder of carbohydrate transport and m	0	1	0	0	1	0	0	
272	240–279	Endocrine, nutritional, and metabolic di	NA	DISORDERS OF LIPOID METABOLISM	1	1	0	0	1	0	0	
2720	240–279	Endocrine, nutritional, and metabolic di	NA	PURE HYPERCHOLESTEROLEMIA	0	0	0	1	0	0	1	
2721	240–279	Endocrine, nutritional, and metabolic di	NA	PURE HYPERGLYCERIDEMIA	0	0	0	1	0	0	1	
2722	240–279	Endocrine, nutritional, and metabolic di	NA	Mixed hyperlipidemia	0	0	0	0	0	0	1	
2723	240–279	Endocrine, nutritional, and metabolic di	NA	Hyperchylomicronemia	0	1	0	0	0	0	1	
2725	240–279	Endocrine, nutritional, and metabolic di	NA	Lipoprotein deficiencies	0	1	0	0	1	0	0	
2726	240–279	Endocrine, nutritional, and metabolic di	NA	Lipodystrophy	0	1	0	0	1	0	0	
2727	240–279	Endocrine, nutritional, and metabolic di	NA	Lipidoses	0	1	0	0	1	0	0	
2728	240–279	Endocrine, nutritional, and metabolic di	NA	Other disorders of lipoid metabolism	0	1	0	0	1	0	0	
2729	240–279	Endocrine, nutritional, and metabolic di	NA	Unspecified disorder of lipoid metabolism	0	0	1	0	0	1	0	
273	240–279	Endocrine, nutritional, and metabolic di	NA	DISORDERS PLASMA PROTEIN METABOLISM	1	1	0	0	1	0	0	
2734	240–279	Endocrine, nutritional, and metabolic di	NA	Alpha-1-antitrypsin deficiency	1	1	0	0	1	0	0	
2739	240–279	Endocrine, nutritional, and metabolic di	NA	UNSPEC DISORDER PLASMA PROT METAB	0	1	0	0	1	0	0	
274	240–279	Endocrine, nutritional, and metabolic di	NA	GOUT	0	0	0	1	0	0	1	
2740	240–279	Endocrine, nutritional, and metabolic di	NA	GOUTY ARTHROPATHY	0	0	0	1	0	0	1	
27400	240–279	Endocrine, nutritional, and metabolic di	NA	Gouty arthropathy, unspecified	0	0	0	0	0	1	0	
27401	240–279	Endocrine, nutritional, and metabolic di	NA	Acute gouty arthropathy	0	0	0	0	0	1	0	
27402	240–279	Endocrine, nutritional, and metabolic di	NA	Chronic gouty arthropathy without mention of tophus	0	0	0	0	0	1	0	
27403	240–279	Endocrine, nutritional, and metabolic di	NA	Chronic gouty arthropathy with tophus (tophi)	0	0	0	0	0	1	0	
2741	240–279	Endocrine, nutritional, and metabolic di	NA	GOUTY NEPHROPATHY	0	0	0	1	0	0	1	
27410	240–279	Endocrine, nutritional, and metabolic di	NA	Gouty nephropathy, unspecified	0	0	0	0	0	1	0	
27411	240–279	Endocrine, nutritional, and metabolic di	NA	Uric acid nephrolithiasis	0	0	0	0	0	1	0	
27419	240–279	Endocrine, nutritional, and metabolic di	NA	Other gouty nephropathy	0	0	0	0	0	1	0	
2749	240–279	Endocrine, nutritional, and metabolic di	NA	Gout, unspecified	0	0	0	0	0	0	1	
275	240–279	Endocrine, nutritional, and metabolic di	NA	DISORDERS OF MINERAL METABOLISM	0	0	0	1	0	0	1	
2750	240–279	Endocrine, nutritional, and metabolic di	NA	DISORDERS OF IRON METABOLISM	0	1	0	0	1	0	0	
27501	240–279	Endocrine, nutritional, and metabolic di	NA	Hereditary hemochromatosis	1	1	0	0	1	0	0	
27503	240–279	Endocrine, nutritional, and metabolic di	NA	Other hemochromatosis	0	1	0	0	0	1	0	
27509	240–279	Endocrine, nutritional, and metabolic di	NA	Other disorders of iron metabolism	0	1	0	0	1	0	0	
2751	240–279	Endocrine, nutritional, and metabolic di	NA	Disorders of copper metabolism	0	1	0	0	1	0	0	
2752	240–279	Endocrine, nutritional, and metabolic di	NA	Disorders of magnesium metabolism	0	0	1	0	0	1	0	
2753	240–279	Endocrine, nutritional, and metabolic di	NA	DISORDERS OF PHOSPHORUS METABOLISM	0	1	0	0	1	0	0	
2754	240–279	Endocrine, nutritional, and metabolic di	NA	DISORDERS OF CALCIUM METABOLISM	0	0	0	1	0	0	1	
2758	240–279	Endocrine, nutritional, and metabolic di	NA	OTH SPEC D/O MINERAL METABOLISM	0	0	0	1	0	0	1	
2759	240–279	Endocrine, nutritional, and metabolic di	NA	UNSPEC DISORDER MINERAL METABOLISM	0	0	0	1	0	0	1	
2770	240–279	Endocrine, nutritional, and metabolic di	NA	CYSTIC FIBROSIS	1	1	0	0	1	0	0	
27700	240–279	Endocrine, nutritional, and metabolic di	NA	Cystic fibrosis without mention of meconium ileus	1	1	0	0	1	0	0	
27701	240–279	Endocrine, nutritional, and metabolic di	NA	Cystic fibrosis with meconium ileus	1	1	0	0	1	0	0	
27702	240–279	Endocrine, nutritional, and metabolic di	NA	Cystic fibrosis with pulmonary manifestations	1	1	0	0	1	0	0	
27703	240–279	Endocrine, nutritional, and metabolic di	NA	Cystic fibrosis with gastrointestinal manifestations	1	1	0	0	1	0	0	
27709	240–279	Endocrine, nutritional, and metabolic di	NA	Cystic fibrosis with other manifestations	1	1	0	0	1	0	0	
2771	240–279	Endocrine, nutritional, and metabolic di	NA	Disorders of porphyrin metabolism	0	1	0	0	1	0	0	
2772	240–279	Endocrine, nutritional, and metabolic di	NA	Other disorders of purine and pyrimidine metabolism	0	1	0	0	1	0	0	
2773	240–279	Endocrine, nutritional, and metabolic di	NA	AMYLOIDOSIS	0	0	0	1	0	0	1	
27730	240–279	Endocrine, nutritional, and metabolic di	NA	Amyloidosis, unspecified	0	0	0	0	0	1	0	
27731	240–279	Endocrine, nutritional, and metabolic di	NA	Familial Mediterranean fever	1	1	0	0	1	0	0	
27739	240–279	Endocrine, nutritional, and metabolic di	NA	Other amyloidosis	0	0	0	0	0	1	0	
2774	240–279	Endocrine, nutritional, and metabolic di	NA	Disorders of bilirubin excretion	0	1	0	0	1	0	0	
2775	240–279	Endocrine, nutritional, and metabolic di	NA	Mucopolysaccharidosis	0	1	0	0	1	0	0	
2776	240–279	Endocrine, nutritional, and metabolic di	NA	Other deficiencies of circulating enzymes	0	1	0	0	1	0	0	
2777	240–279	Endocrine, nutritional, and metabolic di	NA	DYSMETABOLIC SYNDROME X	0	0	0	0	0	0	1	
2778	240–279	Endocrine, nutritional, and metabolic di	NA	OTHER SPEC DISORDERS METABOLISM	0	0	0	1	0	0	1	
27781	240–279	Endocrine, nutritional, and metabolic di	NA	Primary carnitine deficiency	0	1	0	0	1	0	0	
27782	240–279	Endocrine, nutritional, and metabolic di	NA	Carnitine deficiency due to inborn errors of metabolisı	0	1	0	0	1	0	0	
27784	240–279	Endocrine, nutritional, and metabolic di	NA	Other secondary carnitine deficiency	0	0	1	0	0	0	1	
27785	240–279	Endocrine, nutritional, and metabolic di	NA	Disorders of fatty acid oxidation	0	1	0	0	1	0	0	
27786	240–279	Endocrine, nutritional, and metabolic di	NA	Peroxisomal disorders	0	1	0	0	1	0	0	
27787	240–279	Endocrine, nutritional, and metabolic di	NA	Disorders of mitochondrial metabolism	0	0	1	0	0	1	0	
27789	240–279	Endocrine, nutritional, and metabolic di	NA	Other specified disorders of metabolism	0	0	1	0	0	0	1	
2779	240–279	Endocrine, nutritional, and metabolic di	NA	Unspecified disorder of metabolism	0	0	1	0	0	1	0	
27803	240–279	Endocrine, nutritional, and metabolic di	NA	Obesity hypoventilation syndrome	0	0	0	0	0	0	1	
279	240–279	Endocrine, nutritional, and metabolic di	NA	D/O INVOLVING IMMUNE MECHANISM	0	0	0	1	0	0	1	
2790	240–279	Endocrine, nutritional, and metabolic di	NA	Unspecified hypogammaglobulinemia	0	0	0	0	1	0	0	
27900	240–279	Endocrine, nutritional, and metabolic di	NA	Hypogammaglobulinemia, unspecified	0	0	0	0	0	1	0	
27901	240–279	Endocrine, nutritional, and metabolic di	NA	Selective IgA immunodeficiency	0	0	0	0	0	1	0	
27902	240–279	Endocrine, nutritional, and metabolic di	NA	Selective IgM immunodeficiency	0	0	0	0	0	1	0	
27903	240–279	Endocrine, nutritional, and metabolic di	NA	Other selective immunoglobulin deficiencies	0	0	0	0	0	1	0	
27904	240–279	Endocrine, nutritional, and metabolic di	NA	Congenital hypogammaglobulinemia	0	0	1	0	0	1	0	
27905	240–279	Endocrine, nutritional, and metabolic di	NA	Immunodeficiency with increased IgM	0	1	0	0	1	0	0	
27906	240–279	Endocrine, nutritional, and metabolic di	NA	Common variable immunodeficiency	0	0	0	0	1	0	0	
27909	240–279	Endocrine, nutritional, and metabolic di	NA	Other deficiency of humoral immunity	0	0	1	0	0	1	0	
2791	240–279	Endocrine, nutritional, and metabolic di	NA	DEFICIENCY CELL-MEDIATED IMMUNITY	0	0	1	0	0	1	0	

27910	240–279	Endocrine, nutritional, and metabolic di	NA	Immunodeficiency with predominant T-cell defect, ur	0	1	0	0	1	0	0	
27911	240–279	Endocrine, nutritional, and metabolic di	NA	Digeorge's syndrome	1	1	0	0	1	0	0	
27912	240–279	Endocrine, nutritional, and metabolic di	NA	Wiskott-aldrich syndrome	0	1	0	0	1	0	0	
27913	240–279	Endocrine, nutritional, and metabolic di	NA	Nezelof's syndrome	0	1	0	0	1	0	0	
27919	240–279	Endocrine, nutritional, and metabolic di	NA	Other deficiency of cell-mediated immunity	0	1	0	0	1	0	0	
2792	240–279	Endocrine, nutritional, and metabolic di	NA	Combined immunity deficiency	0	1	0	0	1	0	0	
2793	240–279	Endocrine, nutritional, and metabolic di	NA	UNSPECIFIED IMMUNITY DEFICIENCY	0	0	0	1	0	0	1	
2794	240–279	Endocrine, nutritional, and metabolic di	NA	AUTOIMMUNE DISEASE NEC	0	0	0	1	0	0	1	
27941	240–279	Endocrine, nutritional, and metabolic di	NA	Autoimmune lymphoproliferative syndrome	0	1	0	0	1	0	0	
2798	240–279	Endocrine, nutritional, and metabolic di	NA	Other specified disorders involving the immune mech	0	0	1	0	0	1	0	
2799	240–279	Endocrine, nutritional, and metabolic di	NA	Unspecified disorder of immune mechanism	0	0	0	1	0	0	1	
282	280–289	Diseases of the blood	NA	HEREDITARY HEMOLYTIC ANEMIAS	0	1	0	0	1	0	0	
2820	280–289	Diseases of the blood	NA	Hereditary spherocytosis	0	1	0	0	1	0	0	
2821	280–289	Diseases of the blood	NA	Hereditary elliptocytosis	0	1	0	0	1	0	0	
2822	280–289	Diseases of the blood	NA	Anemias due to disorders of glutathione metabolism	0	1	0	0	1	0	0	
2823	280–289	Diseases of the blood	NA	Other hemolytic anemias due to enzyme deficiency	0	1	0	0	1	0	0	
2824	280–289	Diseases of the blood	NA	THALASSEMIAS	1	1	0	0	1	0	0	
28240	280–289	Diseases of the blood	NA	Thalassemia, unspecified	1	1	0	0	1	0	0	
28241	280–289	Diseases of the blood	NA	Sickle-cell thalassemia without crisis	1	1	0	0	1	0	0	
28242	280–289	Diseases of the blood	NA	Sickle-cell thalassemia with crisis	1	1	0	0	1	0	0	
28243	280–289	Diseases of the blood	NA	Alpha thalassemia	1	1	0	0	1	0	0	
28244	280–289	Diseases of the blood	NA	Beta thalassemia	1	1	0	0	1	0	0	
28245	280–289	Diseases of the blood	NA	Delta-beta thalassemia	1	1	0	0	1	0	0	
28246	280–289	Diseases of the blood	NA	Thalassemia minor	1	1	0	0	1	0	0	
28247	280–289	Diseases of the blood	NA	Hemoglobin E-beta thalassemia	1	1	0	0	1	0	0	
28249	280–289	Diseases of the blood	NA	Other thalassemia	1	1	0	0	1	0	0	
2825	280–289	Diseases of the blood	NA	Sickle-cell trait	1	1	0	0	1	0	0	
2826	280–289	Diseases of the blood	NA	SICKLE-CELL DISEASE	1	1	0	0	1	0	0	
28260	280–289	Diseases of the blood	NA	Sickle-cell disease, unspecified	1	1	0	0	1	0	0	
28261	280–289	Diseases of the blood	NA	Hb-SS disease without crisis	1	1	0	0	1	0	0	
28262	280–289	Diseases of the blood	NA	Hb-SS disease with crisis	1	1	0	0	1	0	0	
28263	280–289	Diseases of the blood	NA	Sickle-cell/Hb-C disease without crisis	1	1	0	0	1	0	0	
28264	280–289	Diseases of the blood	NA	Sickle-cell/Hb-C disease with crisis	1	1	0	0	1	0	0	
28268	280–289	Diseases of the blood	NA	Other sickle-cell disease without crisis	1	1	0	0	1	0	0	
28269	280–289	Diseases of the blood	NA	Other sickle-cell disease with crisis	1	1	0	0	1	0	0	
2827	280–289	Diseases of the blood	NA	Other hemoglobinopathies	0	1	0	0	1	0	0	
2828	280–289	Diseases of the blood	NA	Other specified hereditary hemolytic anemias	0	1	0	0	1	0	0	
2829	280–289	Diseases of the blood	NA	Hereditary hemolytic anemia, unspecified	0	1	0	0	1	0	0	
284	280–289	Diseases of the blood	NA	APLASTIC ANEMIA & OTH BM FAIL SYN	0	0	1	0	0	0	1	
2840	280–289	Diseases of the blood	NA	CONSTITUTIONAL APLASTIC ANEMIA	0	0	0	1	0	0	1	
28401	280–289	Diseases of the blood	NA	Constitutional red blood cell aplasia	0	0	1	0	0	1	0	
28409	280–289	Diseases of the blood	NA	Other constitutional aplastic anemia	0	0	1	0	0	1	0	
2848	280–289	Diseases of the blood	NA	OTHER SPECIFIED APLASTIC ANEMIAS	0	0	0	1	0	0	1	
28489	280–289	Diseases of the blood	NA	Other specified aplastic anemias	0	0	1	0	0	1	0	
2849	280–289	Diseases of the blood	NA	Aplastic anemia, unspecified	0	0	0	1	0	0	1	
2850	280–289	Diseases of the blood	NA	Sideroblastic anemia	0	1	0	0	1	0	0	
286	280–289	Diseases of the blood	NA	COAGULATION DEFECTS	0	0	0	1	0	0	1	
2860	280–289	Diseases of the blood	NA	Congenital factor VIII disorder	1	1	0	0	1	0	0	
2861	280–289	Diseases of the blood	NA	Congenital factor IX disorder	1	1	0	0	1	0	0	
2862	280–289	Diseases of the blood	NA	Congenital factor XI deficiency	0	1	0	0	1	0	0	
2863	280–289	Diseases of the blood	NA	Congenital deficiency of other clotting factors	0	1	0	0	1	0	0	
2864	280–289	Diseases of the blood	NA	Von Willebrand's disease	1	1	0	0	1	0	0	
2869	280–289	Diseases of the blood	NA	OTHER&UNSPEC COAGULATION DEFECTS	0	0	1	0	0	0	1	
2871	280–289	Diseases of the blood	NA	Qualitative platelet defects	0	1	0	0	1	0	0	
2873	280–289	Diseases of the blood	NA	PRIMARY THROMBOCYTOPENIA	0	0	1	0	0	1	0	
28730	280–289	Diseases of the blood	NA	Primary thrombocytopenia,unspecified	0	0	1	0	0	1	0	
28733	280–289	Diseases of the blood	NA	Congenital and hereditary thrombocytopenic purpura	0	1	0	0	1	0	0	
288	280–289	Diseases of the blood	NA	DISEASES OF WHITE BLOOD CELLS	0	0	1	0	0	0	1	
28801	280–289	Diseases of the blood	NA	Congenital neutropenia	0	0	1	0	0	1	0	
28802	280–289	Diseases of the blood	NA	Cyclic neutropenia	0	0	1	0	0	1	0	
2881	280–289	Diseases of the blood	NA	Functional disorders of polymorphonuclear neutrophi	0	1	0	0	1	0	0	
2882	280–289	Diseases of the blood	NA	Genetic anomalies of leukocytes	0	1	0	0	1	0	0	
2884	280–289	Diseases of the blood	NA	Hemophagocytic syndromes	0	1	0	0	1	0	0	
2896	280–289	Diseases of the blood	NA	Familial polycythemia	0	1	0	0	1	0	0	
2897	280–289	Diseases of the blood	NA	Methemoglobinemia	1	1	0	0	1	0	0	
28981	280–289	Diseases of the blood	NA	Primary hypercoagulable state	0	0	1	0	0	1	0	
29381	290-319	Mental disorders	NA	Psychotic disorder with delusions in conditions classifi	0	0	0	0	0	0	1	
29382	290-319	Mental disorders	NA	Psychotic disorder with hallucinations in conditions cla	0	0	0	0	0	0	1	
295	290-319	Mental disorders	NA	SCHIZOPHRENIC DISORDERS	0	0	0	0	0	0	1	
29500	290-319	Mental disorders	NA	Simple type schizophrenia, unspecified	0	0	0	0	0	0	1	
29501	290-319	Mental disorders	NA	Simple type schizophrenia, subchronic	0	0	0	0	0	0	1	
29502	290-319	Mental disorders	NA	Simple type schizophrenia, chronic	0	0	0	0	0	0	1	
29503	290-319	Mental disorders	NA	Simple type schizophrenia, subchronic with acute exa	0	0	0	0	0	0	1	
29504	290-319	Mental disorders	NA	Simple type schizophrenia, chronic with acute exacerl	0	0	0	0	0	0	1	
29505	290-319	Mental disorders	NA	Simple type schizophrenia, in remission	0	0	0	0	0	0	1	
29510	290-319	Mental disorders	NA	Disorganized type schizophrenia, unspecified	0	0	0	0	0	0	1	
29511	290-319	Mental disorders	NA	Disorganized type schizophrenia, subchronic	0	0	0	0	0	0	1	
29512	290-319	Mental disorders	NA	Disorganized type schizophrenia, chronic	0	0	0	0	0	0	1	
29513	290-319	Mental disorders	NA	Disorganized type schizophrenia, subchronic with acu	0	0	0	0	0	0	1	
29514	290-319	Mental disorders	NA	Disorganized type schizophrenia, chronic with acute e	0	0	0	0	0	0	1	
29515	290-319	Mental disorders	NA	Disorganized type schizophrenia, in remission	0	0	0	0	0	0	1	
29520	290-319	Mental disorders	NA	Catatonic type schizophrenia, unspecified	0	0	0	0	0	0	1	
29521	290-319	Mental disorders	NA	Catatonic type schizophrenia, subchronic	0	0	0	0	0	0	1	
29522	290-319	Mental disorders	NA	Catatonic type schizophrenia, chronic	0	0	0	0	0	0	1	
29523	290-319	Mental disorders	NA	Catatonic type schizophrenia, subchronic with acute e	0	0	0	0	0	0	1	
29524	290-319	Mental disorders	NA	Catatonic type schizophrenia, chronic with acute exac	0	0	0	0	0	0	1	
29525	290-319	Mental disorders	NA	Catatonic type schizophrenia, in remission	0	0	0	0	0	0	1	
2959	290-319	Mental disorders	NA	UNSPECIFIED SCHIZOPHRENIA	0	0	0	0	0	0	1	
299	290-319	Mental disorders	NA	PERVASIVE DEVELOPMENTAL DISORDERS	0	0	0	0	0	0	1	
2990	290-319	Mental disorders	NA	AUTISTIC DISORDER	0	0	0	0	0	0	1	
29900	290-319	Mental disorders	NA	Autistic disorder, current or active state	0	0	0	0	0	0	1	
29901	290-319	Mental disorders	NA	Autistic disorder, residual state	0	0	0	0	0	0	1	
2991	290-319	Mental disorders	NA	CHILDHOOD DISINTEGRATIVE DISORDER	0	0	0	0	0	0	1	
29910	290-319	Mental disorders	NA	Childhood disintegrative disorder, current or active sta	0	0	0	0	0	0	1	
29911	290-319	Mental disorders	NA	Childhood disintegrative disorder, residual state	0	0	0	0	0	0	1	
2998	290-319	Mental disorders	NA	OTH SPEC PERVASIVE DVLPMENTL D/O	0	0	0	0	0	0	1	
29980	290-319	Mental disorders	NA	Other specified pervasive developmental disorders, c	0	0	0	0	0	0	1	
29981	290-319	Mental disorders	NA	Other specified pervasive developmental disorders, r	0	0	0	0	0	0	1	
2999	290-319	Mental disorders	NA	UNSPEC PERVASIVE DVLPMENTL DISORDER	0	0	0	0	0	0	1	
29990	290-319	Mental disorders	NA	Unspecified pervasive developmental disorder, curre	0	0	0	0	0	0	1	
29991	290-319	Mental disorders	NA	Unspecified pervasive developmental disorder, resid	0	0	0	0	0	0	1	
3073	290-319	Mental disorders	NA	Stereotypic movement disorder	0	0	0	1	0	0	1	
31534	290-319	Mental disorders	NA	Speech and language developmental delay due to he	0	0	0	0	0	0	1	
3155	290-319	Mental disorders	NA	MIXED DEVELOPMENT DISORDER	0	0	0	0	0	0	1	1
317	290-319	Mental disorders	NA	Mild intellectual disabilities	0	0	0	0	0	0	1	
318	290-319	Mental disorders	NA	OTHER SPECIFIED MENTAL RETARDATION	0	0	0	0	0	0	1	1
3180	290-319	Mental disorders	NA	Moderate intellectual disabilities	0	0	0	0	0	0	1	1
3181	290-319	Mental disorders	NA	Severe intellectual disabilities	0	0	0	0	0	1	0	1
3182	290-319	Mental disorders	NA	Profound intellectual disabilities	0	0	0	0	0	1	0	1
319	290-319	Mental disorders	NA	Unspecified intellectual disabilities	0	0	0	0	0	0	1	1
3190	290-319	Mental disorders	NA	UNSPECIFIED MTL RETARD	0	0	0	1	0	0	1	1
32721	320–359	Diseases of the nervous system	NA	Primary central sleep apnea	0	0	1	0	0	1	0	
32725	320–359	Diseases of the nervous system	NA	Congenital central alveolar hypoventilation syndrome	0	0	1	0	0	1	0	
32727	320–359	Diseases of the nervous system	NA	Central sleep apnea in conditions classified elsewhere	0	0	1	0	0	0	1	
3300	320–359	Diseases of the nervous system	NA	Leukodystrophy	0	0	0	0	0	1	0	
3301	320–359	Diseases of the nervous system	NA	Cerebral lipidoses	0	1	0	0	1	0	0	
3302	320–359	Diseases of the nervous system	NA	Cerebral degeneration in generalized lipidoses	0	1	0	0	1	0	0	
3303	320–359	Diseases of the nervous system	NA	Cerebral degeneration of childhood in other diseases	0	1	0	0	1	0	0	
3308	320–359	Diseases of the nervous system	NA	Other specified cerebral degenerations in childhood	0	1	0	0	1	0	0	
3309	320–359	Diseases of the nervous system	NA	Unspecified cerebral degeneration in childhood	0	0	0	0	0	1	0	
3311	320–359	Diseases of the nervous system	NA	FRONTOTEMPORAL DEMENTIA	0	0	0	0	0	1	0	
33111	320–359	Diseases of the nervous system	NA	Pick's disease	1	0	0	0	0	0	1	
33119	320–359	Diseases of the nervous system	NA	Other frontotemporal dementia	1	0	0	0	0	0	1	
3313	320–359	Diseases of the nervous system	NA	Communicating hydrocephalus	0	0	0	1	0	0	1	
3314	320–359	Diseases of the nervous system	NA	Obstructive hydrocephalus	0	0	0	1	0	0	1	
3315	320–359	Diseases of the nervous system	NA	Idiopathic normal pressure hydrocephalus [INPH]	0	0	0	1	0	0	1	
3316	320–359	Diseases of the nervous system	NA	Corticobasal degeneration	0	0	0	0	1	0	0	
3317	320–359	Diseases of the nervous system	NA	CERBRL DEGEN DISEASES CLASS ELSW	0	0	0	0	0	1	0	
33181	320–359	Diseases of the nervous system	NA	Reye's syndrome	0	0	0	0	0	0	1	

33182	320–359	Diseases of the nervous system	NA	Dementia with Lewy bodies	0	0	0	0	0	0	1	
33189	320–359	Diseases of the nervous system	NA	Other cerebral degeneration	0	0	0	0	0	0	1	
3319	320–359	Diseases of the nervous system	NA	UNSPECIFIED CEREBRAL DEGENERATION	0	0	0	0	0	1	0	
3330	320–359	Diseases of the nervous system	NA	Other degenerative diseases of the basal ganglia	0	0	0	0	1	0	0	
3331	320–359	Diseases of the nervous system	NA	Essential and other specified forms of tremor	0	0	0	0	0	0	1	
3333	320–359	Diseases of the nervous system	NA	Tics of organic origin	0	0	0	0	0	0	1	
3334	320–359	Diseases of the nervous system	NA	Huntington's chorea	1	1	0	0	1	0	0	
3335	320–359	Diseases of the nervous system	NA	Other choreas	0	0	0	0	0	1	0	
3336	320–359	Diseases of the nervous system	NA	Genetic torsion dystonia	0	1	0	0	1	0	0	
33371	320–359	Diseases of the nervous system	NA	Athetoid cerebral palsy	0	0	0	0	0	0	1	
33383	320–359	Diseases of the nervous system	NA	Spasmodic torticollis	0	0	0	1	0	0	1	
33391	320–359	Diseases of the nervous system	NA	Stiff-man syndrome	0	0	0	0	0	0	1	
334	320–359	Diseases of the nervous system	NA	SPINOCEREBELLAR DISEASE	0	0	0	0	0	1	0	
3340	320–359	Diseases of the nervous system	NA	Friedreich's ataxia	1	1	0	0	1	0	0	
3341	320–359	Diseases of the nervous system	NA	Hereditary spastic paraplegia	0	1	0	0	1	0	0	
3342	320–359	Diseases of the nervous system	NA	Primary cerebellar degeneration	0	1	0	0	1	0	0	
3343	320–359	Diseases of the nervous system	NA	Other cerebellar ataxia	0	0	0	0	0	1	0	
3344	320–359	Diseases of the nervous system	NA	Cerebellar ataxia in diseases classified elsewhere	0	0	0	0	0	1	0	
3348	320–359	Diseases of the nervous system	NA	Other spinocerebellar diseases	0	0	0	0	0	1	0	
3349	320–359	Diseases of the nervous system	NA	Spinocerebellar disease, unspecified	0	0	0	0	0	1	0	
335	320–359	Diseases of the nervous system	NA	ANTERIOR HORN CELL DISEASE	0	1	0	0	1	0	0	
3350	320–359	Diseases of the nervous system	NA	Werdnig-Hoffmann disease	1	1	0	0	1	0	0	
3351	320–359	Diseases of the nervous system	NA	SPINAL MUSCULAR ATROPHY	1	1	0	0	1	0	0	
33510	320–359	Diseases of the nervous system	NA	Spinal muscular atrophy, unspecified	1	1	0	0	1	0	0	
33511	320–359	Diseases of the nervous system	NA	Kugelberg-Welander disease	1	1	0	0	1	0	0	
33519	320–359	Diseases of the nervous system	NA	Other spinal muscular atrophy	0	1	0	0	1	0	0	
3352	320–359	Diseases of the nervous system	NA	MOTOR NEURON DISEASE	0	1	0	0	1	0	0	
33520	320–359	Diseases of the nervous system	NA	Amyotrophic lateral sclerosis	0	0	0	0	0	1	0	
33521	320–359	Diseases of the nervous system	NA	Progressive muscular atrophy	0	0	0	0	0	1	0	
33522	320–359	Diseases of the nervous system	NA	Progressive bulbar palsy	0	0	0	0	0	1	0	
33524	320–359	Diseases of the nervous system	NA	Primary lateral sclerosis	0	0	0	0	0	1	0	
33529	320–359	Diseases of the nervous system	NA	Other motor neuron disease	0	0	1	0	0	1	0	
3358	320–359	Diseases of the nervous system	NA	Other anterior horn cell diseases	0	0	0	0	0	1	0	
3359	320–359	Diseases of the nervous system	NA	Anterior horn cell disease, unspecified	0	0	0	0	0	1	0	
3360	320–359	Diseases of the nervous system	NA	Syringomyelia and syringobulbia	0	0	0	1	0	0	1	
3370	320–359	Diseases of the nervous system	NA	IDIOPATH PERIPH AUTONOM NEUROPATHY	0	0	0	0	0	1	0	
3371	320–359	Diseases of the nervous system	NA	PRIPHERL AUTONOMIC NEUROPTHY D/O CE	0	0	0	0	0	1	0	
3380	320–359	Diseases of the nervous system	NA	CENTRAL PAIN SYNDROME	0	0	0	0	0	0	1	
3384	320–359	Diseases of the nervous system	NA	Chronic pain syndrome	0	0	0	0	0	0	1	
3411	320–359	Diseases of the nervous system	NA	Schilder's disease	1	1	0	0	1	0	0	
3418	320–359	Diseases of the nervous system	NA	Other demyelinating diseases of central nervous syst	0	0	0	0	1	0	0	
3430	320–359	Diseases of the nervous system	NA	Diplegic infantile cerebral palsy	0	0	0	0	0	0	1	
3431	320–359	Diseases of the nervous system	NA	Hemiplegic infantile cerebral palsy	0	0	0	0	0	0	1	
3432	320–359	Diseases of the nervous system	NA	Quadriplegic infantile cerebral palsy	0	0	0	0	0	0	1	
3433	320–359	Diseases of the nervous system	NA	Monoplegic infantile cerebral palsy	0	0	0	0	1	0	0	
3438	320–359	Diseases of the nervous system	NA	Other specified infantile cerebral palsy	0	0	0	1	0	0	1	
3439	320–359	Diseases of the nervous system	NA	Infantile cerebral palsy, unspecified	0	0	0	1	0	0	1	
345	320–359	Diseases of the nervous system	NA	EPILEPSY AND RECURRENT SEIZURES	0	0	1	0	0	0	1	1
3450	320–359	Diseases of the nervous system	NA	GENERALIZED NONCONVULSIVE EPILEPSY	0	0	1	0	0	0	1	1
34500	320–359	Diseases of the nervous system	NA	Generalized nonconvulsive epilepsy, without mentior	0	0	1	0	0	0	1	1
34501	320–359	Diseases of the nervous system	NA	Generalized nonconvulsive epilepsy, with intractable	0	0	1	0	0	0	1	1
3451	320–359	Diseases of the nervous system	NA	GENERALIZED CONVULSIVE EPILEPSY	0	0	1	0	0	0	1	1
34510	320–359	Diseases of the nervous system	NA	Generalized convulsive epilepsy, without mention of i	0	0	1	0	0	0	1	1
34511	320–359	Diseases of the nervous system	NA	Generalized convulsive epilepsy, with intractable epil	0	0	1	0	0	0	1	1
3452	320–359	Diseases of the nervous system	NA	Petit mal status	0	0	0	1	0	0	1	1
3453	320–359	Diseases of the nervous system	NA	Grand mal status	0	0	0	1	0	0	1	1
3454	320–359	Diseases of the nervous system	NA	LOC-REL EPILEPSY & ES W/CPS	0	0	1	0	0	0	1	1
34540	320–359	Diseases of the nervous system	NA	Localization-related (focal) (partial) epilepsy and epile	0	0	1	0	0	0	1	1
34541	320–359	Diseases of the nervous system	NA	Localization-related (focal) (partial) epilepsy and epile	0	0	1	0	0	0	1	1
3455	320–359	Diseases of the nervous system	NA	LOC-REL EPILEPSY & ES W/SPS	0	0	1	0	0	0	1	1
34550	320–359	Diseases of the nervous system	NA	Localization-related (focal) (partial) epilepsy and epile	0	0	1	0	0	0	1	1
34551	320–359	Diseases of the nervous system	NA	Localization-related (focal) (partial) epilepsy and epile	0	0	1	0	0	0	1	1
3456	320–359	Diseases of the nervous system	NA	INFANTILE SPASMS	0	0	1	0	0	0	1	1
34560	320–359	Diseases of the nervous system	NA	Infantile spasms, without mention of intractable epile	0	0	1	0	0	1	0	1
34561	320–359	Diseases of the nervous system	NA	Infantile spasms, with intractable epilepsy	0	0	1	0	0	1	0	1
3457	320–359	Diseases of the nervous system	NA	EPILEPSIA PARTIALIS CONTINUA	0	0	1	0	0	0	1	1
34570	320–359	Diseases of the nervous system	NA	Epilepsia partialis continua, without mention of intract	0	0	1	0	0	0	1	1
34571	320–359	Diseases of the nervous system	NA	Epilepsia partialis continua, with intractable epilepsy	0	0	1	0	0	0	1	1
3458	320–359	Diseases of the nervous system	NA	OTH FORMS EPILEPSY&RECUR SEIZURES	0	0	1	0	0	0	1	1
34580	320–359	Diseases of the nervous system	NA	Other forms of epilepsy and recurrent seizures, witho	0	0	1	0	0	0	1	1
34581	320–359	Diseases of the nervous system	NA	Other forms of epilepsy and recurrent seizures, with i	0	0	1	0	0	0	1	1
3459	320–359	Diseases of the nervous system	NA	UNSPECIFIED EPILEPSY	0	0	1	0	0	0	1	1
34590	320–359	Diseases of the nervous system	NA	Epilepsy, unspecified, without mention of intractable (	0	0	1	0	0	0	1	1
34591	320–359	Diseases of the nervous system	NA	Epilepsy, unspecified, with intractable epilepsy	0	0	1	0	0	0	1	1
34831	320–359	Diseases of the nervous system	NA	Metabolic encephalopathy	0	0	1	0	0	1	0	
34839	320–359	Diseases of the nervous system	NA	Other encephalopathy	0	0	1	0	0	0	1	
356	320–359	Diseases of the nervous system	NA	HEREDIT&IDIOPATH PERIPH NEUROPATHY	0	0	0	0	0	1	0	
3560	320–359	Diseases of the nervous system	NA	Hereditary peripheral neuropathy	0	1	0	0	1	0	0	
3561	320–359	Diseases of the nervous system	NA	PERONEAL MUSCULAR ATROPHY	0	0	0	0	0	0	1	
3562	320–359	Diseases of the nervous system	NA	Hereditary sensory neuropathy	0	1	0	0	1	0	0	
3563	320–359	Diseases of the nervous system	NA	Refsum's disease	1	1	0	0	1	0	0	
3564	320–359	Diseases of the nervous system	NA	Idiopathic progressive polyneuropathy	0	0	0	0	0	1	0	
3568	320–359	Diseases of the nervous system	NA	Other specified idiopathic peripheral neuropathy	0	0	0	0	0	1	0	
3569	320–359	Diseases of the nervous system	NA	Unspecified hereditary and idiopathic peripheral neur	0	0	0	0	0	1	0	
3588	320–359	Diseases of the nervous system	NA	Other specified myoneural disorders	0	0	1	0	0	1	0	
359	320–359	Diseases of the nervous system	NA	MUSCULAR DYSTROPHIES&OTH MYOPATHIES	0	0	1	0	0	1	0	
3590	320–359	Diseases of the nervous system	NA	Congenital hereditary muscular dystrophy	0	1	0	0	1	0	0	
3591	320–359	Diseases of the nervous system	NA	Hereditary progressive muscular dystrophy	0	1	0	0	1	0	0	
3592	320–359	Diseases of the nervous system	NA	MYOTONIC DISORDERS	0	1	0	0	1	0	0	
35921	320–359	Diseases of the nervous system	NA	Myotonic muscular dystrophy	1	1	0	0	1	0	0	
35922	320–359	Diseases of the nervous system	NA	Myotonia congenita	0	1	0	0	1	0	0	
35923	320–359	Diseases of the nervous system	NA	Myotonic chondrodystrophy	0	1	0	0	1	0	0	
35929	320–359	Diseases of the nervous system	NA	Other specified myotonic disorder	0	0	0	0	0	0	1	
3593	320–359	Diseases of the nervous system	NA	Periodic paralysis	0	1	0	0	1	0	0	
3598	320–359	Diseases of the nervous system	NA	OTHER MYOPATHIES	0	0	1	0	0	1	0	
35989	320–359	Diseases of the nervous system	NA	Other myopathies	0	0	1	0	0	1	0	
3599	320–359	Diseases of the nervous system	NA	Myopathy, unspecified	0	0	1	0	0	1	0	
36110	360–389	Diseases of the sense organs	NA	Retinoschisis, unspecified	0	1	0	0	1	0	0	
36111	360–389	Diseases of the sense organs	NA	Flat retinoschisis	0	1	0	0	1	0	0	
36119	360–389	Diseases of the sense organs	NA	Other retinoschisis and retinal cysts	0	0	0	0	0	0	1	
36215	360–389	Diseases of the sense organs	NA	Retinal telangiectasia	0	0	0	0	0	0	1	
3626	360–389	Diseases of the sense organs	NA	PERIPHERAL RETINAL DEGENERATIONS	0	0	0	0	0	0	1	
3627	360–389	Diseases of the sense organs	NA	HEREDITARY RETINAL DYSTROPHIES	0	0	0	0	1	0	0	
36270	360–389	Diseases of the sense organs	NA	Hereditary retinal dystrophy, unspecified	0	1	0	0	1	0	0	
36271	360–389	Diseases of the sense organs	NA	RETINAL DYSTROPHY-LIPIDOSES	0	0	0	0	0	1	0	
36272	360–389	Diseases of the sense organs	NA	RETINAL DYSTROPHY OTH SYS D/O&SYNDS	0	0	0	0	0	1	0	
36273	360–389	Diseases of the sense organs	NA	Vitreoretinal dystrophies	0	0	0	0	0	0	1	
36274	360–389	Diseases of the sense organs	NA	Pigmentary retinal dystrophy	0	0	0	0	0	1	0	
36275	360–389	Diseases of the sense organs	NA	Other dystrophies primarily involving the sensory reti	0	1	0	0	1	0	0	
36276	360–389	Diseases of the sense organs	NA	Dystrophies primarily involving the retinal pigment ep	0	1	0	0	1	0	0	
36277	360–389	Diseases of the sense organs	NA	Dystrophies primarily involving Bruch's membrane	0	1	0	0	1	0	0	
3635	360–389	Diseases of the sense organs	NA	HEREDITARY CHOROIDAL DYSTROPHIES	0	0	0	0	1	0	0	
36350	360–389	Diseases of the sense organs	NA	Hereditary choroidal dystrophy or atrophy, unspecifie	0	1	0	0	1	0	0	
36353	360–389	Diseases of the sense organs	NA	Central dystrophy of choroid, partial	0	0	0	0	0	0	1	
36355	360–389	Diseases of the sense organs	NA	Choroideremia	1	1	0	0	1	0	0	
36356	360–389	Diseases of the sense organs	NA	Other diffuse or generalized dystrophy of choroid, par	0	0	0	0	0	0	1	
36357	360–389	Diseases of the sense organs	NA	Other diffuse or generalized dystrophy of choroid, tot	0	0	0	0	0	0	1	
36452	360–389	Diseases of the sense organs	NA	Iridoschisis	0	0	0	0	0	0	1	
365	360–389	Diseases of the sense organs	NA	GLAUCOMA	0	0	1	0	0	1	0	
36511	360–389	Diseases of the sense organs	NA	Primary open angle glaucoma	0	0	1	0	0	0	1	
36514	360–389	Diseases of the sense organs	NA	Glaucoma of childhood	0	0	0	0	0	1	0	
3655	360–389	Diseases of the sense organs	NA	GLAUCOMA ASSOC W/DISORDERS LENS	0	0	1	0	0	1	0	
3658	360–389	Diseases of the sense organs	NA	OTHER SPECIFIED FORMS OF GLAUCOMA	0	0	1	0	0	1	0	
3659	360–389	Diseases of the sense organs	NA	UNSPECIFIED GLAUCOMA	0	0	1	0	0	1	0	
366	360–389	Diseases of the sense organs	NA	CATARACT	0	0	1	0	0	1	0	
3660	360–389	Diseases of the sense organs	NA	INFNTILE JUV&PRESENILE CATARACT	0	0	1	0	0	1	0	
36600	360–389	Diseases of the sense organs	NA	Nonsenile cataract, unspecified	0	0	1	0	0	1	0	
36601	360–389	Diseases of the sense organs	NA	Anterior subcapsular polar cataract	0	0	1	0	0	1	0	
36602	360–389	Diseases of the sense organs	NA	Posterior subcapsular polar cataract	0	0	1	0	0	1	0	

36603	360–389	Diseases of the sense organs	NA	Cortical, lamellar, or zonular cataract	0	0	1	0	0	1	0
36604	360–389	Diseases of the sense organs	NA	Nuclear cataract	0	0	1	0	0	1	0
36609	360–389	Diseases of the sense organs	NA	Other and combined forms of nonsenile cataract	0	0	1	0	0	1	0
3685	360–389	Diseases of the sense organs	NA	COLOR VISION DEFICIENCIES	1	0	0	0	1	0	0
36851	360–389	Diseases of the sense organs	NA	Protan defect	1	1	0	0	1	0	0
36852	360–389	Diseases of the sense organs	NA	Deutan defect	1	1	0	0	1	0	0
36853	360–389	Diseases of the sense organs	NA	Tritan defect	1	1	0	0	1	0	0
36854	360–389	Diseases of the sense organs	NA	Achromatopsia	0	1	0	0	1	0	0
36859	360–389	Diseases of the sense organs	NA	Other color vision deficiencies	1	0	0	0	0	1	0
36861	360–389	Diseases of the sense organs	NA	Congenital night blindness	1	1	0	0	1	0	0
37114	360–389	Diseases of the sense organs	NA	Kayser-Fleischer ring	1	1	0	0	1	0	0
37115	360–389	Diseases of the sense organs	NA	Other corneal deposits associated with metabolic disc	0	1	0	0	1	0	0
3714	360–389	Diseases of the sense organs	NA	CORNEAL DEGENERATIONS	0	0	0	0	0	1	0
3715	360–389	Diseases of the sense organs	NA	HEREDITARY CORNEAL DYSTROPHIES	0	0	0	0	1	0	0
37150	360–389	Diseases of the sense organs	NA	Hereditary corneal dystrophy, unspecified	0	1	0	0	1	0	0
37151	360–389	Diseases of the sense organs	NA	Juvenile epithelial corneal dystrophy	0	0	0	0	0	1	0
37152	360–389	Diseases of the sense organs	NA	Other anterior corneal dystrophies	0	0	1	0	0	1	0
37153	360–389	Diseases of the sense organs	NA	Granular corneal dystrophy	0	0	1	0	0	1	0
37154	360–389	Diseases of the sense organs	NA	Lattice corneal dystrophy	0	0	1	0	0	1	0
37155	360–389	Diseases of the sense organs	NA	Macular corneal dystrophy	0	0	1	0	0	1	0
37156	360–389	Diseases of the sense organs	NA	Other stromal corneal dystrophies	0	0	1	0	0	1	0
37157	360–389	Diseases of the sense organs	NA	Endothelial corneal dystrophy	0	0	1	0	0	1	0
37158	360–389	Diseases of the sense organs	NA	Other posterior corneal dystrophies	0	0	1	0	0	1	0
3716	360–389	Diseases of the sense organs	NA	KERATOCONUS	0	0	0	0	0	0	1
37160	360–389	Diseases of the sense organs	NA	Keratoconus, unspecified	0	0	1	0	0	1	0
37161	360–389	Diseases of the sense organs	NA	Keratoconus, stable condition	0	0	1	0	0	1	0
37162	360–389	Diseases of the sense organs	NA	Keratoconus, acute hydrops	0	0	1	0	0	1	0
3717	360–389	Diseases of the sense organs	NA	OTHER CORNEAL DEFORMITIES	0	0	0	1	0	0	1
37170	360–389	Diseases of the sense organs	NA	Corneal deformity, unspecified	0	0	1	0	0	1	0
3718	360–389	Diseases of the sense organs	NA	OTHER CORNEAL DISORDERS	0	0	0	1	0	0	1
3719	360–389	Diseases of the sense organs	NA	UNSPECIFIED CORNEAL DISORDER	0	0	0	1	0	0	1
3742	360–389	Diseases of the sense organs	NA	LAGOPHTHALMOS	0	0	0	1	0	0	1
37446	360–389	Diseases of the sense organs	NA	Blepharophimosis	0	0	0	1	0	0	1
3764	360–389	Diseases of the sense organs	NA	DEFORMITY OF ORBIT	0	0	0	1	0	0	1
37641	360–389	Diseases of the sense organs	NA	HYPERTELORISM OF ORBIT	0	0	0	1	0	0	1
3765	360–389	Diseases of the sense organs	NA	ENOPHTHALMOS	0	0	0	1	0	0	1
37650	360–389	Diseases of the sense organs	NA	Enophthalmos, unspecified as to cause	0	0	0	1	0	0	1
37651	360–389	Diseases of the sense organs	NA	Enophthalmos due to atrophy of orbital tissue	0	0	0	1	0	0	1
3771	360–389	Diseases of the sense organs	NA	OPTIC ATROPHY	0	0	0	1	0	0	1
37710	360–389	Diseases of the sense organs	NA	Optic atrophy, unspecified	0	0	1	0	0	1	0
37711	360–389	Diseases of the sense organs	NA	Primary optic atrophy	0	0	1	0	0	1	0
37713	360–389	Diseases of the sense organs	NA	Optic atrophy associated with retinal dystrophies	0	0	1	0	0	1	0
37716	360–389	Diseases of the sense organs	NA	Hereditary optic atrophy	0	1	0	0	1	0	0
37723	360–389	Diseases of the sense organs	NA	Coloboma of optic disc	0	0	0	1	0	0	1
37743	360–389	Diseases of the sense organs	NA	Optic nerve hypoplasia	0	0	0	1	0	0	1
37855	360–389	Diseases of the sense organs	NA	Paralytic strabismus, external ophthalmoplegia	0	0	0	0	0	0	1
37871	360–389	Diseases of the sense organs	NA	Duane's syndrome	0	0	0	1	0	0	1
37872	360–389	Diseases of the sense organs	NA	Progressive external ophthalmoplegia	0	0	0	0	0	1	0
37873	360–389	Diseases of the sense organs	NA	Strabismus in other neuromuscular disorders	0	0	0	0	0	1	0
37886	360–389	Diseases of the sense organs	NA	Internuclear ophthalmoplegia	0	0	0	1	0	0	1
37932	360–389	Diseases of the sense organs	NA	Subluxation of lens	0	1	0	0	1	0	0
37933	360–389	Diseases of the sense organs	NA	Anterior dislocation of lens	0	1	0	0	1	0	0
37934	360–389	Diseases of the sense organs	NA	Posterior dislocation of lens	0	1	0	0	1	0	0
37939	360–389	Diseases of the sense organs	NA	Other disorders of lens	0	0	0	1	0	0	1
37950	360–389	Diseases of the sense organs	NA	Nystagmus, unspecified	0	0	1	0	0	1	0
37951	360–389	Diseases of the sense organs	NA	Congenital nystagmus	0	0	1	0	0	1	0
389	360–389	Diseases of the sense organs	NA	HEARING LOSS	0	0	0	1	0	0	1
3891	360–389	Diseases of the sense organs	NA	SENSORINEURAL HEARING LOSS	0	0	0	1	0	0	1
38910	360–389	Diseases of the sense organs	NA	Sensorineural hearing loss, unspecified	0	0	1	0	0	1	0
38911	360–389	Diseases of the sense organs	NA	Sensory hearing loss, bilateral	0	0	1	0	0	1	0
38912	360–389	Diseases of the sense organs	NA	Neural hearing loss, bilateral	0	0	1	0	0	1	0
38913	360–389	Diseases of the sense organs	NA	Neural hearing loss, unilateral	0	0	1	0	0	1	0
38914	360–389	Diseases of the sense organs	NA	Central hearing loss	0	0	1	0	0	1	0
38915	360–389	Diseases of the sense organs	NA	Sensorineural hearing loss, unilateral	0	0	1	0	0	1	0
38916	360–389	Diseases of the sense organs	NA	Sensorineural hearing loss, asymmetrical	0	0	1	0	0	1	0
38917	360–389	Diseases of the sense organs	NA	Sensory hearing loss, unilateral	0	0	1	0	0	1	0
38918	360–389	Diseases of the sense organs	NA	Sensorineural hearing loss, bilateral	0	0	1	0	0	1	0
3892	360–389	Diseases of the sense organs	NA	MIX CONDCTIVE&SENSINURAL HEARNG LSS	0	0	0	1	0	0	1
38920	360–389	Diseases of the sense organs	NA	Mixed hearing loss, unspecified	0	0	0	1	0	0	1
38921	360–389	Diseases of the sense organs	NA	Mixed hearing loss, unilateral	0	0	0	1	0	0	1
38922	360–389	Diseases of the sense organs	NA	Mixed hearing loss, bilateral	0	0	0	1	0	0	1
3897	360–389	Diseases of the sense organs	NA	Deaf, nonspeaking, not elsewhere classifiable	0	0	0	0	0	1	0
3898	360–389	Diseases of the sense organs	NA	Other specified forms of hearing loss	0	0	0	1	0	0	1
3899	360–389	Diseases of the sense organs	NA	UNSPECIFIED HEARING LOSS	0	0	0	1	0	0	0
3940	390–459	Diseases of the circulatory system	NA	Mitral stenosis	0	0	0	1	0	0	1
3942	390–459	Diseases of the circulatory system	NA	Mitral stenosis with insufficiency	0	0	0	1	0	0	1
395	390–459	Diseases of the circulatory system	NA	DISEASES OF AORTIC VALVE	0	0	0	1	0	0	1
3960	390–459	Diseases of the circulatory system	NA	Mitral valve stenosis and aortic valve stenosis	0	0	0	1	0	0	1
3961	390–459	Diseases of the circulatory system	NA	Mitral valve stenosis and aortic valve insufficiency	0	0	0	1	0	0	1
3962	390–459	Diseases of the circulatory system	NA	Mitral valve insufficiency and aortic valve stenosis	0	0	0	1	0	0	1
3963	390–459	Diseases of the circulatory system	NA	Mitral valve insufficiency and aortic valve insufficienc	0	0	0	1	0	0	1
3968	390–459	Diseases of the circulatory system	NA	Multiple involvement of mitral and aortic valves	0	0	0	1	0	0	1
3969	390–459	Diseases of the circulatory system	NA	Mitral and aortic valve diseases, unspecified	0	0	0	1	0	0	1
397	390–459	Diseases of the circulatory system	NA	DISEASES OTH ENDOCARDIAL STRUCTURES	0	0	0	1	0	0	1
3970	390–459	Diseases of the circulatory system	NA	Diseases of tricuspid valve	0	0	0	1	0	0	1
4240	390–459	Diseases of the circulatory system	NA	Mitral valve disorders	0	0	0	1	0	0	1
4241	390–459	Diseases of the circulatory system	NA	Aortic valve disorders	0	0	0	1	0	0	1
4242	390–459	Diseases of the circulatory system	NA	Tricuspid valve disorders, specified as nonrheumatic	0	0	0	1	0	0	1
4243	390–459	Diseases of the circulatory system	NA	Pulmonary valve disorders	0	0	0	1	0	0	1
425	390–459	Diseases of the circulatory system	NA	CARDIOMYOPATHY	0	0	0	1	0	0	1
4250	390–459	Diseases of the circulatory system	NA	ENDOMYOCARDIAL FIBROSIS	0	0	0	1	0	0	1
42511	390–459	Diseases of the circulatory system	NA	Hypertrophic obstructive cardiomyopathy	0	0	0	0	0	0	1
42518	390–459	Diseases of the circulatory system	NA	Other hypertrophic cardiomyopathy	0	0	0	0	0	0	1
4253	390–459	Diseases of the circulatory system	NA	Endocardial fibroelastosis	0	1	0	0	1	0	0
4254	390–459	Diseases of the circulatory system	NA	Other primary cardiomyopathies	0	0	1	0	0	1	0
4257	390–459	Diseases of the circulatory system	NA	NUTRITIONAL&METAB CARDIOMYOPATHY	0	0	0	1	0	0	1
4258	390–459	Diseases of the circulatory system	NA	CARDIOMYOPATHY OTH DZ CLASS ELSW	0	0	0	1	0	0	1
426	390–459	Diseases of the circulatory system	NA	CONDUCTION DISORDERS	0	0	0	1	0	0	1
4260	390–459	Diseases of the circulatory system	NA	Atrioventricular block, complete	0	0	0	1	0	0	1
4261	390–459	Diseases of the circulatory system	NA	ATRIOVENTRICULAR BLOCK OTHER&UNSPEC	0	0	0	1	0	0	1
42610	390–459	Diseases of the circulatory system	NA	Atrioventricular block, unspecified	0	0	0	0	0	1	0
42611	390–459	Diseases of the circulatory system	NA	First degree atrioventricular block	0	0	0	1	0	0	1
42612	390–459	Diseases of the circulatory system	NA	Mobitz (type) II atrioventricular block	0	0	0	1	0	0	1
4262	390–459	Diseases of the circulatory system	NA	Left bundle branch hemiblock	0	0	0	1	0	0	1
4263	390–459	Diseases of the circulatory system	NA	Other left bundle branch block	0	0	0	1	0	0	1
4264	390–459	Diseases of the circulatory system	NA	Right bundle branch block	0	0	0	1	0	0	1
4265	390–459	Diseases of the circulatory system	NA	BUNDLE BRANCH BLOCK OTHER&UNSPEC	0	0	0	1	0	0	1
42650	390–459	Diseases of the circulatory system	NA	Bundle branch block, unspecified	0	0	0	1	0	0	1
42651	390–459	Diseases of the circulatory system	NA	Right bundle branch block and left posterior fascicular	0	0	0	1	0	0	1
42652	390–459	Diseases of the circulatory system	NA	Right bundle branch block and left anterior fascicular l	0	0	0	1	0	0	1
42653	390–459	Diseases of the circulatory system	NA	Other bilateral bundle branch block	0	0	0	1	0	0	1
4266	390–459	Diseases of the circulatory system	NA	OTHER HEART BLOCK	0	0	0	1	0	0	1
4267	390–459	Diseases of the circulatory system	NA	Anomalous atrioventricular excitation	0	0	0	1	0	0	1
4268	390–459	Diseases of the circulatory system	NA	OTHER SPEC CONDUCTION DISORDERS	0	0	0	1	0	0	1
42681	390–459	Diseases of the circulatory system	NA	Lown-Ganong-Levine syndrome	0	0	0	1	0	0	1
42682	390–459	Diseases of the circulatory system	NA	Long QT syndrome	0	0	1	0	0	1	0
42689	390–459	Diseases of the circulatory system	NA	Other specified conduction disorders	0	0	0	1	0	0	1
4269	390–459	Diseases of the circulatory system	NA	Conduction disorder, unspecified	0	0	0	1	0	0	1
427	390–459	Diseases of the circulatory system	NA	CARDIAC DYSRHYTHMIAS	0	0	0	1	0	0	1
4271	390–459	Diseases of the circulatory system	NA	PAROXYSMAL VENTRICULAR TACHYCARDIA	0	0	0	1	0	0	1
4272	390–459	Diseases of the circulatory system	NA	UNSPECIFIED PAROXYSMAL TACHYCARDIA	0	0	0	1	0	0	1
4273	390–459	Diseases of the circulatory system	NA	ATRIAL FIBRILLATION AND FLUTTER	0	0	0	1	0	0	1
42731	390–459	Diseases of the circulatory system	NA	Atrial fibrillation	0	0	0	0	0	1	0
4274	390–459	Diseases of the circulatory system	NA	VENTRICULAR FIBRILLATION&FLUTTER	0	0	0	1	0	0	1
42741	390–459	Diseases of the circulatory system	NA	Ventricular fibrillation	0	0	1	0	0	1	0
42742	390–459	Diseases of the circulatory system	NA	Ventricular flutter	0	0	0	0	0	1	0
4278	390–459	Diseases of the circulatory system	NA	OTHER SPEC CARDIAC DYSRHYTHMIAS	0	0	0	1	0	0	1
42781	390–459	Diseases of the circulatory system	NA	Sinoatrial node dysfunction	0	0	1	0	0	1	0
4279	390–459	Diseases of the circulatory system	NA	UNSPECIFIED CARDIAC DYSRHYTHMIA	0	0	0	1	0	0	1

428	390–459	Diseases of the circulatory system	NA	HEART FAILURE	0	0	0	1	0	0	1
4280	390–459	Diseases of the circulatory system	NA	Congestive heart failure, unspecified	0	0	0	1	0	0	1
4281	390–459	Diseases of the circulatory system	NA	Left heart failure	0	0	0	1	0	0	1
4282	390–459	Diseases of the circulatory system	NA	SYSTOLIC HEART FAILURE	0	0	0	1	0	0	1
4283	390–459	Diseases of the circulatory system	NA	DIASTOLIC HEART FAILURE	0	0	0	1	0	0	1
4284	390–459	Diseases of the circulatory system	NA	COMB SYSTOLIC&DIASTOLIC HEART FAIL	0	0	0	1	0	0	1
4289	390–459	Diseases of the circulatory system	NA	Heart failure, unspecified	0	0	0	1	0	0	1
4293	390–459	Diseases of the circulatory system	NA	Cardiomegaly	0	0	0	1	0	0	1
4373	390–459	Diseases of the circulatory system	NA	Cerebral aneurysm, nonruptured	0	0	0	0	0	0	1
4375	390–459	Diseases of the circulatory system	NA	Moyamoya disease	0	0	1	0	0	1	0
440	390–459	Diseases of the circulatory system	NA	ATHEROSCLEROSIS	0	0	0	0	0	1	0
4408	390–459	Diseases of the circulatory system	NA	ATHEROSCLEROSIS OTHER SPEC ARTERIES	0	0	0	0	0	1	0
4409	390–459	Diseases of the circulatory system	NA	GENERALIZED&UNSPEC ATHEROSCLEROSIS	0	0	0	0	0	1	0
441	390–459	Diseases of the circulatory system	NA	AORTIC ANEURYSM AND DISSECTION	0	0	1	0	0	1	0
4410	390–459	Diseases of the circulatory system	NA	DISSECTION OF AORTA	0	0	0	0	0	1	0
44100	390–459	Diseases of the circulatory system	NA	Dissection of aorta, unspecified site	0	0	1	0	0	1	0
44101	390–459	Diseases of the circulatory system	NA	Dissection of aorta, thoracic	0	0	1	0	0	1	0
4411	390–459	Diseases of the circulatory system	NA	Thoracic aneurysm, ruptured	0	0	1	0	0	1	0
4412	390–459	Diseases of the circulatory system	NA	Thoracic aneurysm without mention of rupture	0	0	1	0	0	1	0
4413	390–459	Diseases of the circulatory system	NA	ABDOMINAL ANEURYSM, RUPTURED	0	0	0	0	0	0	1
4414	390–459	Diseases of the circulatory system	NA	ABD ANEUR WITHOUT MENTION RUPTURE	0	0	0	0	0	0	1
4415	390–459	Diseases of the circulatory system	NA	AORTIC ANEUR UNSPEC SITE RUPTURED	0	0	0	0	0	0	1
4416	390–459	Diseases of the circulatory system	NA	THORACOABDOMINAL ANEURYSM, RUPTURED	0	0	0	0	0	0	1
4417	390–459	Diseases of the circulatory system	NA	THORACOABD ANEUR W/O MENTION RUP	0	0	0	0	0	0	1
4419	390–459	Diseases of the circulatory system	NA	Aortic aneurysm of unspecified site without mention	0	0	1	0	0	1	0
442	390–459	Diseases of the circulatory system	NA	OTHER ANEURYSM	0	0	0	0	0	1	0
4420	390–459	Diseases of the circulatory system	NA	Aneurysm of artery of upper extremity	0	0	1	0	0	1	0
4421	390–459	Diseases of the circulatory system	NA	Aneurysm of renal artery	0	0	1	0	0	1	0
4422	390–459	Diseases of the circulatory system	NA	Aneurysm of iliac artery	0	0	1	0	0	1	0
4423	390–459	Diseases of the circulatory system	NA	Aneurysm of artery of lower extremity	0	0	1	0	0	1	0
44281	390–459	Diseases of the circulatory system	NA	Aneurysm of artery of neck	0	0	1	0	0	1	0
44282	390–459	Diseases of the circulatory system	NA	Aneurysm of subclavian artery	0	0	1	0	0	1	0
4429	390–459	Diseases of the circulatory system	NA	OTHER ANEURYSM OF UNSPECIFIED SITE	0	0	0	1	0	0	1
44329	390–459	Diseases of the circulatory system	NA	Dissection of other artery	0	0	0	0	0	0	1
44382	390–459	Diseases of the circulatory system	NA	Erythromelalgia	1	0	0	0	0	0	1
4472	390–459	Diseases of the circulatory system	NA	RUPTURE OF ARTERY	0	0	0	1	0	0	1
4473	390–459	Diseases of the circulatory system	NA	HYPERPLASIA OF RENAL ARTERY	0	0	0	0	0	1	0
44770	390–459	Diseases of the circulatory system	NA	Aortic ectasia, unspecified site	0	0	1	0	0	1	0
44771	390–459	Diseases of the circulatory system	NA	Thoracic aortic ectasia	0	0	1	0	0	1	0
4480	390–459	Diseases of the circulatory system	NA	Hereditary hemorrhagic telangiectasia	0	1	0	0	1	0	0
4940	460–519	Diseases of the respiratory system	NA	Bronchiectasis without acute exacerbation	0	0	1	0	0	1	0
4941	460–519	Diseases of the respiratory system	NA	Bronchiectasis with acute exacerbation	0	0	1	0	0	1	0
5120	460–519	Diseases of the respiratory system	NA	Spontaneous tension pneumothorax	0	0	0	0	0	1	0
5128	460–519	Diseases of the respiratory system	NA	OTHER SPONTANEOUS PNEUMOTHORAX	0	0	0	1	0	0	1
51281	460–519	Diseases of the respiratory system	NA	Primary spontaneous pneumothorax	0	0	0	0	0	1	0
5160	460–519	Diseases of the respiratory system	NA	Pulmonary alveolar proteinosis	0	0	1	0	0	1	0
5161	460–519	Diseases of the respiratory system	NA	Idiopathic pulmonary hemosiderosis	0	0	1	0	0	1	0
5163	460–519	Diseases of the respiratory system	NA	IDIOPATHIC FIBROSING ALVEOLITIS	0	0	0	0	0	0	1
51630	460–519	Diseases of the respiratory system	NA	Idiopathic interstitial pneumonia, not otherwise speci	0	0	1	0	0	1	0
51631	460–519	Diseases of the respiratory system	NA	Idiopathic pulmonary fibrosis	0	0	0	0	0	1	0
51632	460–519	Diseases of the respiratory system	NA	Idiopathic non-specific interstitial pneumonitis	0	0	1	0	0	0	1
51635	460–519	Diseases of the respiratory system	NA	Idiopathic lymphoid interstitial pneumonia	0	0	1	0	0	0	1
51637	460–519	Diseases of the respiratory system	NA	Desquamative interstitial pneumonia	0	0	1	0	0	0	1
5164	460–519	Diseases of the respiratory system	NA	Lymphangioleiomyomatosis	1	1	0	0	1	0	0
51663	460–519	Diseases of the respiratory system	NA	Surfactant mutations of the lung	0	1	0	0	1	0	0
51664	460–519	Diseases of the respiratory system	NA	Alveolar capillary dysplasia with vein misalignment	1	1	0	0	1	0	0
5200	520–579	Diseases of the digestive system	NA	Anodontia	0	0	0	0	0	1	0
5202	520–579	Diseases of the digestive system	NA	ABNORMALITIES OF SIZE&FORM OF TEETH	0	0	0	0	0	1	0
5204	520–579	Diseases of the digestive system	NA	Disturbances of tooth formation	0	0	0	0	0	1	0
5205	520–579	Diseases of the digestive system	NA	Hereditary disturbances in tooth structure, not elsewh	0	1	0	0	1	0	0
5240	520–579	Diseases of the digestive system	NA	MAJOR ANOMALIES OF JAW SIZE	0	0	0	1	0	0	1
52400	520–579	Diseases of the digestive system	NA	Unspecified major anomaly of jaw size	0	0	0	1	0	0	1
52403	520–579	Diseases of the digestive system	NA	Major anomalies of jaw size, maxillary hypoplasia	0	0	0	1	0	0	1
52404	520–579	Diseases of the digestive system	NA	Major anomalies of jaw size, mandibular hypoplasia	0	0	0	1	0	0	1
52406	520–579	Diseases of the digestive system	NA	Microgenia	0	0	0	1	0	0	1
52473	520–579	Diseases of the digestive system	NA	Alveolar maxillary hypoplasia	0	0	0	1	0	0	1
52474	520–579	Diseases of the digestive system	NA	Alveolar mandibular hypoplasia	0	0	0	1	0	0	1
5249	520–579	Diseases of the digestive system	NA	Unspecified dentofacial anomalies	0	0	0	1	0	0	1
5260	520–579	Diseases of the digestive system	NA	Developmental odontogenic cysts	0	0	0	1	0	0	1
5262	520–579	Diseases of the digestive system	NA	OTHER CYSTS OF JAWS	0	0	0	1	0	0	1
53084	520–579	Diseases of the digestive system	NA	Tracheoesophageal fistula	0	0	0	1	0	0	1
5513	520–579	Diseases of the digestive system	NA	Diaphragmatic hernia with gangrene	0	0	0	1	0	0	1
5523	520–579	Diseases of the digestive system	NA	Diaphragmatic hernia with obstruction	0	0	0	1	0	0	1
5533	520–579	Diseases of the digestive system	NA	Diaphragmatic hernia without mention of obstruction	0	0	0	1	0	0	1
5647	520–579	Diseases of the digestive system	NA	Megacolon, other than Hirschsprung's	0	0	0	1	0	0	1
5771	520–579	Diseases of the digestive system	NA	Chronic pancreatitis	0	0	0	0	0	1	0
58181	580-629	Diseases of the genitourinary system	NA	Nephrotic syndrome in diseases classified elsewhere	0	0	1	0	0	0	1
5831	580-629	Diseases of the genitourinary system	NA	Nephritis and nephropathy, not specified as acute or	0	0	0	0	0	0	1
5832	580-629	Diseases of the genitourinary system	NA	Nephritis and nephropathy, not specified as acute or	0	0	0	0	0	0	1
58381	580-629	Diseases of the genitourinary system	NA	Nephritis and nephropathy, not specified as acute or	0	0	0	1	0	0	1
58389	580-629	Diseases of the genitourinary system	NA	Other nephritis and nephropathy, not specified as acu	0	0	0	1	0	0	1
5839	580-629	Diseases of the genitourinary system	NA	Nephritis and nephropathy, not specified as acute or	0	0	0	0	0	0	1
5881	580-629	Diseases of the genitourinary system	NA	NEPHROGENIC DIABETES INSIPIDUS	0	0	0	1	0	0	1
589	580-629	Diseases of the genitourinary system	NA	SMALL KIDNEY OF UNKNOWN CAUSE	0	0	0	1	0	0	1
5890	580-629	Diseases of the genitourinary system	NA	UNILATERAL SMALL KIDNEY	0	0	0	1	0	0	1
5891	580-629	Diseases of the genitourinary system	NA	BILATERAL SMALL KIDNEYS	0	0	0	1	0	0	1
5899	580-629	Diseases of the genitourinary system	NA	UNSPECIFIED SMALL KIDNEY	0	0	0	1	0	0	1
591	580-629	Diseases of the genitourinary system	NA	HYDRONEPHROSIS	0	0	0	1	0	0	1
5920	580-629	Diseases of the genitourinary system	NA	Calculus of kidney	0	0	0	0	0	0	1
5931	580-629	Diseases of the genitourinary system	NA	HYPERTROPHY OF KIDNEY	0	0	0	1	0	0	1
5933	580-629	Diseases of the genitourinary system	NA	STRICTURE OR KINKING OF URETER	0	0	0	1	0	0	1
5934	580-629	Diseases of the genitourinary system	NA	OTHER URETERIC OBSTRUCTION	0	0	0	1	0	0	1
5935	580-629	Diseases of the genitourinary system	NA	HYDROURETER	0	0	0	1	0	0	1
606	580-629	Diseases of the genitourinary system	NA	MALE INFERTILITY	0	0	0	0	0	0	0
6060	580-629	Diseases of the genitourinary system	NA	AZOOSPERMIA	0	0	0	0	0	0	0
6061	580-629	Diseases of the genitourinary system	NA	OLIGOSPERMIA	0	0	0	0	0	0	0
61182	580-629	Diseases of the genitourinary system	NA	Hypoplasia of breast	0	0	0	0	0	0	1
6186	580-629	Diseases of the genitourinary system	NA	VAGINAL ENTEROCELE CONGENITAL/ACQ	0	0	0	1	0	0	1
6191	580-629	Diseases of the genitourinary system	NA	Digestive-genital tract fistula, female	0	0	0	1	0	0	1
65360	630-679	Complications of pregnancy, childbirth,	NA	Hydrocephalic fetus causing disproportion, unspecifie	0	0	0	1	0	0	1
65361	630-679	Complications of pregnancy, childbirth,	NA	Hydrocephalic fetus causing disproportion, delivered,	0	0	1	0	0	1	0
65363	630-679	Complications of pregnancy, childbirth,	NA	Hydrocephalic fetus causing disproportion, antepartu	0	0	1	0	0	1	0
65370	630-679	Complications of pregnancy, childbirth,	NA	Other fetal abnormality causing disproportion, unspe	0	0	1	0	0	1	0
65371	630-679	Complications of pregnancy, childbirth,	NA	Other fetal abnormality causing disproportion, delive	0	0	1	0	0	1	0
65373	630-679	Complications of pregnancy, childbirth,	NA	Other fetal abnormality causing disproportion, antepa	0	0	1	0	0	1	0
65500	630-679	Complications of pregnancy, childbirth,	NA	Central nervous system malformation in fetus, unspe	0	0	1	0	0	1	0
65501	630-679	Complications of pregnancy, childbirth,	NA	Central nervous system malformation in fetus, delive	0	0	1	0	0	1	0
65503	630-679	Complications of pregnancy, childbirth,	NA	Central nervous system malformation in fetus, antep	0	0	1	0	0	1	0
65510	630-679	Complications of pregnancy, childbirth,	NA	Chromosomal abnormality in fetus, affecting manage	1	1	0	0	1	0	0
65511	630-679	Complications of pregnancy, childbirth,	NA	Chromosomal abnormality in fetus, affecting manage	1	1	0	0	1	0	0
65513	630-679	Complications of pregnancy, childbirth,	NA	Chromosomal abnormality in fetus, affecting manage	1	1	0	0	1	0	0
65520	630-679	Complications of pregnancy, childbirth,	NA	Hereditary disease in family possibly affecting fetus,	1	1	0	0	1	0	0
65521	630-679	Complications of pregnancy, childbirth,	NA	Hereditary disease in family possibly affecting fetus,	1	1	0	0	1	0	0
65523	630-679	Complications of pregnancy, childbirth,	NA	Hereditary disease in family possibly affecting fetus,	1	1	0	0	1	0	0
65570	630-679	Complications of pregnancy, childbirth,	NA	Decreased fetal movements, affecting management	0	0	0	1	0	0	0
65571	630-679	Complications of pregnancy, childbirth,	NA	Decreased fetal movements, affecting management	0	0	0	1	0	0	0
65573	630-679	Complications of pregnancy, childbirth,	NA	Decreased fetal movements, affecting management	0	0	0	1	0	0	0
65580	630-679	Complications of pregnancy, childbirth,	NA	Other known or suspected fetal abnormality, not else	0	0	0	1	0	0	1
65581	630-679	Complications of pregnancy, childbirth,	NA	Other known or suspected fetal abnormality, not else	0	0	0	1	0	0	1
65583	630-679	Complications of pregnancy, childbirth,	NA	Other known or suspected fetal abnormality, not else	0	0	0	1	0	0	1
65590	630-679	Complications of pregnancy, childbirth,	NA	Unspecified suspected fetal abnormality, affecting ma	0	0	0	1	0	0	1
65591	630-679	Complications of pregnancy, childbirth,	NA	Unspecified suspected fetal abnormality, affecting ma	0	0	0	1	0	0	1
65593	630-679	Complications of pregnancy, childbirth,	NA	Unspecified suspected fetal abnormality, affecting ma	0	0	0	1	0	0	1
65650	630-679	Complications of pregnancy, childbirth,	NA	Poor fetal growth, affecting management of mother,	0	0	0	1	0	0	1
65651	630-679	Complications of pregnancy, childbirth,	NA	Poor fetal growth, affecting management of mother,	0	0	0	1	0	0	1
65653	630-679	Complications of pregnancy, childbirth,	NA	Poor fetal growth, affecting management of mother,	0	0	0	1	0	0	1
65660	630-679	Complications of pregnancy, childbirth,	NA	Excessive fetal growth, affecting management of mo	0	0	0	1	0	0	1
65661	630-679	Complications of pregnancy, childbirth,	NA	Excessive fetal growth, affecting management of mo	0	0	0	1	0	0	1
65663	630-679	Complications of pregnancy, childbirth,	NA	Excessive fetal growth, affecting management of mo	0	0	0	1	0	0	1

65700	630-679	Complications of pregnancy, childbirth,	NA	Polyhydramnios, unspecified as to episode of care or	0	0	0	1	0	0	1	
65701	630-679	Complications of pregnancy, childbirth,	NA	Polyhydramnios, delivered, with or without mention c	0	0	0	1	0	0	1	
65703	630-679	Complications of pregnancy, childbirth,	NA	Polyhydramnios, antepartum condition or complicatic	0	0	0	1	0	0	1	
65800	630-679	Complications of pregnancy, childbirth,	NA	Oligohydramnios, unspecified as to episode of care or	0	0	0	1	0	0	1	
65801	630-679	Complications of pregnancy, childbirth,	NA	Oligohydramnios, delivered, with or without mention	0	0	0	1	0	0	1	
65803	630-679	Complications of pregnancy, childbirth,	NA	Oligohydramnios, antepartum condition or complicati	0	0	0	1	0	0	1	
67800	630-679	Complications of pregnancy, childbirth,	NA	Fetal hematologic conditions, unspecified as to episoc	0	0	1	0	0	1	0	
67801	630-679	Complications of pregnancy, childbirth,	NA	Fetal hematologic conditions, delivered, with or witho	0	0	1	0	0	1	0	
67803	630-679	Complications of pregnancy, childbirth,	NA	Fetal hematologic conditions, antepartum condition o	0	0	1	0	0	1	0	
705	680-709	Diseases of the skin and subcutaneous	NA	DISORDERS OF SWEAT GLANDS	0	0	0	1	0	0	1	
7050	680-709	Diseases of the skin and subcutaneous	NA	ANHIDROSIS	0	0	0	0	0	1	0	
7059	680-709	Diseases of the skin and subcutaneous	NA	UNSPECIFIED DISORDER SWEAT GLANDS	0	0	0	0	0	0	1	
70909	680-709	Diseases of the skin and subcutaneous	NA	Other dyschromia	0	0	0	1	0	0	1	
7108	710–739	Diseases of the musculoskeletal syster	NA	Other specified diffuse disease of connective tissue	0	0	1	0	0	0	1	
7109	710–739	Diseases of the musculoskeletal syster	NA	Unspecified diffuse connective tissue disease	0	0	0	0	0	0	1	
7121	710–739	Diseases of the musculoskeletal syster	NA	CHONDROCALCINOS-DICALCM PO4 CRYSTL	0	0	0	0	0	1	0	
7122	710–739	Diseases of the musculoskeletal syster	NA	CHONDROCALCINOSIS-PYROPHOSPHATE	0	0	0	0	0	1	0	
7123	710–739	Diseases of the musculoskeletal syster	NA	CHONDROCALCINOSIS CAUSE UNSPECIFIED	0	0	0	0	0	1	0	
71230	710–739	Diseases of the musculoskeletal syster	NA	Chondrocalcinosis, cause unspecified, involving unspe	1	0	0	0	0	0	1	
71231	710–739	Diseases of the musculoskeletal syster	NA	Chondrocalcinosis, cause unspecified, involving shoul	1	0	0	0	0	0	1	
71232	710–739	Diseases of the musculoskeletal syster	NA	Chondrocalcinosis, cause unspecified, involving upper	1	0	0	0	0	0	1	
71233	710–739	Diseases of the musculoskeletal syster	NA	Chondrocalcinosis, cause unspecified, involving forea	1	0	0	0	0	0	1	
71234	710–739	Diseases of the musculoskeletal syster	NA	Chondrocalcinosis, cause unspecified, involving hand	1	0	0	0	0	0	1	
71235	710–739	Diseases of the musculoskeletal syster	NA	Chondrocalcinosis, cause unspecified, involving pelvic	1	0	0	0	0	0	1	
71236	710–739	Diseases of the musculoskeletal syster	NA	Chondrocalcinosis, cause unspecified, involving lower	1	0	0	0	0	0	1	
71237	710–739	Diseases of the musculoskeletal syster	NA	Chondrocalcinosis, cause unspecified, involving ankle	1	0	0	0	0	0	1	
71238	710–739	Diseases of the musculoskeletal syster	NA	Chondrocalcinosis, cause unspecified, involving other	1	0	0	0	0	0	1	
71239	710–739	Diseases of the musculoskeletal syster	NA	Chondrocalcinosis, cause unspecified, involving multi	1	0	0	0	0	0	1	
7129	710–739	Diseases of the musculoskeletal syster	NA	UNSPECIFIED CRYSTAL ARTHROPATHY	0	0	0	0	0	1	0	
7135	710–739	Diseases of the musculoskeletal syster	NA	ARTHROPATHY ASSOC W/NEURO D/O	0	0	0	1	0	0	1	
71830	710–739	Diseases of the musculoskeletal syster	NA	Recurrent dislocation of joint, site unspecified	0	0	0	1	0	0	1	
71831	710–739	Diseases of the musculoskeletal syster	NA	Recurrent dislocation of joint, shoulder region	0	0	0	1	0	0	1	
71832	710–739	Diseases of the musculoskeletal syster	NA	Recurrent dislocation of joint, upper arm	0	0	0	1	0	0	1	
71833	710–739	Diseases of the musculoskeletal syster	NA	Recurrent dislocation of joint, forearm	0	0	0	1	0	0	1	
71834	710–739	Diseases of the musculoskeletal syster	NA	Recurrent dislocation of joint, hand	0	0	0	1	0	0	1	
71835	710–739	Diseases of the musculoskeletal syster	NA	Recurrent dislocation of joint, pelvic region and thigh	0	0	0	1	0	0	1	
71836	710–739	Diseases of the musculoskeletal syster	NA	Recurrent dislocation of joint, lower leg	0	0	0	1	0	0	1	
71837	710–739	Diseases of the musculoskeletal syster	NA	Recurrent dislocation of joint, ankle and foot	0	0	0	1	0	0	1	
71838	710–739	Diseases of the musculoskeletal syster	NA	Recurrent dislocation of joint, other specified sites	0	0	0	1	0	0	1	
71839	710–739	Diseases of the musculoskeletal syster	NA	Recurrent dislocation of joint, multiple sites	0	0	0	1	0	0	1	
71840	710–739	Diseases of the musculoskeletal syster	NA	Contracture of joint, site unspecified	0	0	0	1	0	0	1	
71841	710–739	Diseases of the musculoskeletal syster	NA	Contracture of joint, shoulder region	0	0	0	1	0	0	1	
71842	710–739	Diseases of the musculoskeletal syster	NA	Contracture of joint, upper arm	0	0	0	1	0	0	1	
71843	710–739	Diseases of the musculoskeletal syster	NA	Contracture of joint, forearm	0	0	0	1	0	0	1	
71844	710–739	Diseases of the musculoskeletal syster	NA	Contracture of joint, hand	0	0	0	1	0	0	1	
71845	710–739	Diseases of the musculoskeletal syster	NA	Contracture of joint, pelvic region and thigh	0	0	0	1	0	0	1	
71846	710–739	Diseases of the musculoskeletal syster	NA	Contracture of joint, lower leg	0	0	0	1	0	0	1	
71847	710–739	Diseases of the musculoskeletal syster	NA	Contracture of joint, ankle and foot	0	0	0	1	0	0	1	
71848	710–739	Diseases of the musculoskeletal syster	NA	Contracture of joint, other specified sites	0	0	0	1	0	0	1	
71849	710–739	Diseases of the musculoskeletal syster	NA	Contracture of joint, multiple sites	0	0	0	1	0	0	1	
71870	710–739	Diseases of the musculoskeletal syster	NA	Developmental dislocation of joint, site unspecified	0	0	1	0	0	1	0	
71871	710–739	Diseases of the musculoskeletal syster	NA	Developmental dislocation of joint, shoulder region	0	0	1	0	0	1	0	
71872	710–739	Diseases of the musculoskeletal syster	NA	Developmental dislocation of joint, upper arm	0	0	1	0	0	1	0	
71873	710–739	Diseases of the musculoskeletal syster	NA	Developmental dislocation of joint, forearm	0	0	1	0	0	1	0	
71874	710–739	Diseases of the musculoskeletal syster	NA	Developmental dislocation of joint, hand	0	0	1	0	0	1	0	
71875	710–739	Diseases of the musculoskeletal syster	NA	Developmental dislocation of joint, pelvic region and t	0	0	1	0	0	1	0	
71876	710–739	Diseases of the musculoskeletal syster	NA	Developmental dislocation of joint, lower leg	0	0	1	0	0	1	0	
71877	710–739	Diseases of the musculoskeletal syster	NA	Developmental dislocation of joint, ankle and foot	0	0	1	0	0	1	0	
71878	710–739	Diseases of the musculoskeletal syster	NA	Developmental dislocation of joint, other specified sit	0	0	1	0	0	1	0	
71879	710–739	Diseases of the musculoskeletal syster	NA	Developmental dislocation of joint, multiple sites	0	0	1	0	0	1	0	
71881	710–739	Diseases of the musculoskeletal syster	NA	Other joint derangement, not elsewhere classified, sh	0	0	0	1	0	0	1	
71882	710–739	Diseases of the musculoskeletal syster	NA	Other joint derangement, not elsewhere classified, up	0	0	0	1	0	0	1	
71883	710–739	Diseases of the musculoskeletal syster	NA	Other joint derangement, not elsewhere classified, fo	0	0	0	1	0	0	1	
71884	710–739	Diseases of the musculoskeletal syster	NA	Other joint derangement, not elsewhere classified, ha	0	0	0	1	0	0	1	
71885	710–739	Diseases of the musculoskeletal syster	NA	Other joint derangement, not elsewhere classified, pe	0	0	0	1	0	0	1	
71887	710–739	Diseases of the musculoskeletal syster	NA	Other joint derangement, not elsewhere classified, ar	0	0	0	1	0	0	1	
7246	710–739	Diseases of the musculoskeletal syster	NA	Disorders of sacrum	0	0	0	1	0	0	1	
7284	710–739	Diseases of the musculoskeletal syster	NA	Laxity of ligament	0	0	0	1	0	0	1	
7285	710–739	Diseases of the musculoskeletal syster	NA	Hypermobility syndrome	0	0	0	0	0	0	1	
72888	710–739	Diseases of the musculoskeletal syster	NA	Rhabdomyolysis	0	0	0	0	0	1	0	
732	710–739	Diseases of the musculoskeletal syster	NA	OSTEOCHONDROPATHIES	0	0	0	0	0	1	0	
7320	710–739	Diseases of the musculoskeletal syster	NA	JUVENILE OSTEOCHONDROSIS OF SPINE	0	0	0	0	0	1	0	
7321	710–739	Diseases of the musculoskeletal syster	NA	JUVENILE OSTEOCHONDROSIS HIP&PELVIS	0	0	0	0	0	1	0	
7329	710–739	Diseases of the musculoskeletal syster	NA	UNSPECIFIED OSTEOCHONDROPATHY	0	0	0	0	0	1	0	
733	710–739	Diseases of the musculoskeletal syster	NA	OTHER DISORDERS BONE AND CARTILAGE	0	0	0	1	0	0	1	
73390	710–739	Diseases of the musculoskeletal syster	NA	DISORDER BONE&CARTILAGE UNSPECIFIED	0	0	0	1	0	0	0	
73391	710–739	Diseases of the musculoskeletal syster	NA	Arrest of bone development or growth	0	0	0	1	0	0	1	
73392	710–739	Diseases of the musculoskeletal syster	NA	Chondromalacia	0	0	0	0	0	1	0	
73622	710–739	Diseases of the musculoskeletal syster	NA	Swan-neck deformity	0	0	0	1	0	0	1	
73731	710–739	Diseases of the musculoskeletal syster	NA	Resolving infantile idiopathic scoliosis	0	0	0	0	0	0	1	
73732	710–739	Diseases of the musculoskeletal syster	NA	Progressive infantile idiopathic scoliosis	0	0	1	0	0	1	0	
7400	740–759	Congenital anomalies	NervousSystem	Anencephalus	0	0	0	1	0	0	0	1
7401	740–759	Congenital anomalies	NervousSystem	Craniorachischisis	0	0	0	1	0	0	1	1
7402	740–759	Congenital anomalies	NervousSystem	Iniencephaly	0	0	0	1	0	0	1	1
74100	740–759	Congenital anomalies	NervousSystem	Spina bifida with hydrocephalus, unspecified region	0	0	0	1	0	0	1	1
74101	740–759	Congenital anomalies	NervousSystem	Spina bifida with hydrocephalus, cervical region	0	0	0	1	0	0	1	1
74102	740–759	Congenital anomalies	NervousSystem	Spina bifida with hydrocephalus, dorsal (thoracic) regi	0	0	0	1	0	0	1	1
74103	740–759	Congenital anomalies	NervousSystem	Spina bifida with hydrocephalus, lumbar region	0	0	0	1	0	0	1	1
74190	740–759	Congenital anomalies	NervousSystem	Spina bifida without mention of hydrocephalus, unspe	0	0	0	1	0	0	1	1
74191	740–759	Congenital anomalies	NervousSystem	Spina bifida without mention of hydrocephalus, cervic	0	0	0	1	0	0	1	1
74192	740–759	Congenital anomalies	NervousSystem	Spina bifida without mention of hydrocephalus, dorsa	0	0	0	1	0	0	1	1
74193	740–759	Congenital anomalies	NervousSystem	Spina bifida without mention of hydrocephalus, lumba	0	0	0	1	0	0	1	1
7420	740–759	Congenital anomalies	NervousSystem	Encephalocele	0	0	0	1	0	0	1	1
7421	740–759	Congenital anomalies	NervousSystem	Microcephalus	0	0	0	1	0	0	1	1
7422	740–759	Congenital anomalies	NervousSystem	Congenital reduction deformities of brain	0	0	0	1	0	0	1	1
7423	740–759	Congenital anomalies	NervousSystem	Congenital hydrocephalus	0	0	0	1	0	0	1	1
7424	740–759	Congenital anomalies	NervousSystem	Other specified congenital anomalies of brain	0	0	0	1	0	0	1	1
74251	740–759	Congenital anomalies	NervousSystem	Diastematomyelia	0	0	0	1	0	0	1	1
74253	740–759	Congenital anomalies	NervousSystem	Hydromyelia	0	0	0	1	0	0	1	1
74259	740–759	Congenital anomalies	NervousSystem	Other specified congenital anomalies of spinal cord	0	0	0	1	0	0	1	1
7428	740–759	Congenital anomalies	NervousSystem	Other specified congenital anomalies of nervous syst	0	0	0	1	0	0	1	1
7429	740–759	Congenital anomalies	NervousSystem	Unspecified congenital anomaly of brain, spinal cord,	0	0	0	1	0	0	1	1
743	740–759	Congenital anomalies	Eye	CONGENITAL ANOMALIES OF EYE	0	0	0	1	0	0	1	1
7430	740–759	Congenital anomalies	Eye	ANOPHTHALMOS	0	0	0	1	0	0	1	1
74300	740–759	Congenital anomalies	Eye	Clinical anophthalmos, unspecified	0	0	1	0	0	1	0	1
74303	740–759	Congenital anomalies	Eye	Cystic eyeball, congenital	0	0	1	0	0	1	0	1
74306	740–759	Congenital anomalies	Eye	Cryptophthalmos	0	0	1	0	0	1	0	1
7431	740–759	Congenital anomalies	Eye	MICROPHTHALMOS	0	0	0	1	0	0	1	1
74310	740–759	Congenital anomalies	Eye	Microphthalmos, unspecified	0	0	0	1	0	0	1	1
74311	740–759	Congenital anomalies	Eye	Simple microphthalmos	0	0	0	1	0	0	1	1
74312	740–759	Congenital anomalies	Eye	Microphthalmos associated with other anomalies of e	0	0	1	0	0	1	0	1
7432	740–759	Congenital anomalies	Eye	BUPHTHALMOS	0	0	0	1	0	0	1	1
74320	740–759	Congenital anomalies	Eye	Buphthalmos, unspecified	0	0	0	1	0	0	1	1
74321	740–759	Congenital anomalies	Eye	Simple buphthalmos	0	0	0	1	0	0	1	1
74322	740–759	Congenital anomalies	Eye	Buphthalmos associated with other ocular anomalies	0	0	0	1	0	0	1	1
7433	740–759	Congenital anomalies	Eye	CONGENITAL CATARACT&LENS ANOMALIES	0	0	0	1	0	0	1	1
74330	740–759	Congenital anomalies	Eye	Congenital cataract, unspecified	0	0	1	0	0	1	0	1
74331	740–759	Congenital anomalies	Eye	Congenital capsular and subcapsular cataract	0	0	1	0	0	1	0	1
74332	740–759	Congenital anomalies	Eye	Congenital cortical and zonular cataract	0	0	1	0	0	1	0	1
74333	740–759	Congenital anomalies	Eye	Congenital nuclear cataract	0	0	1	0	0	1	0	1
74334	740–759	Congenital anomalies	Eye	Total and subtotal cataract, congenital	0	0	1	0	0	1	0	1
74335	740–759	Congenital anomalies	Eye	Congenital aphakia	0	0	1	0	0	1	0	1
74336	740–759	Congenital anomalies	Eye	Congenital anomalies of lens shape	0	0	1	0	0	1	0	1
74337	740–759	Congenital anomalies	Eye	Congenital ectopic lens	0	1	0	0	1	0	0	1
74339	740–759	Congenital anomalies	Eye	Other congenital cataract and lens anomalies	0	0	1	0	0	1	0	1
7434	740–759	Congenital anomalies	Eye	COLOBOMA&OTH ANOMALIES ANT SEG EYE	0	0	0	1	0	0	1	1
74341	740–759	Congenital anomalies	Eye	Congenital anomalies of corneal size and shape	0	0	1	0	0	1	0	1
74342	740–759	Congenital anomalies	Eye	Corneal opacities, interfering with vision, congenital	0	0	1	0	0	1	0	1
74343	740–759	Congenital anomalies	Eye	Other congenital corneal opacity	0	0	0	1	0	0	1	1

74344	740–759	Congenital anomalies	Eye	Specified congenital anomaly of anterior chamber, ch	0	0	0	1	0	0	1	1
74345	740–759	Congenital anomalies	Eye	Aniridia	0	1	0	0	1	0	0	1
74346	740–759	Congenital anomalies	Eye	Other specified congenital anomalies of iris and ciliary	0	0	0	1	0	0	1	1
74347	740–759	Congenital anomalies	Eye	Specified congenital anomalies of sclera	0	0	0	1	0	0	1	1
74348	740–759	Congenital anomalies	Eye	Multiple and combined congenital anomalies of anter	0	0	0	1	0	0	1	1
74349	740–759	Congenital anomalies	Eye	Other congenital anomalies of anterior segment	0	0	0	1	0	0	1	1
7435	740–759	Congenital anomalies	Eye	CONGEN ANOMALIES POST SEGMENT EYE	0	0	0	1	0	0	1	1
74351	740–759	Congenital anomalies	Eye	Vitreous anomaly, congenital	0	0	0	1	0	0	1	1
74352	740–759	Congenital anomalies	Eye	Fundus coloboma	0	0	0	1	0	0	1	1
74353	740–759	Congenital anomalies	Eye	Chorioretinal degeneration, congenital	0	0	0	1	0	0	1	1
74354	740–759	Congenital anomalies	Eye	Congenital folds and cysts of posterior segment	0	0	0	1	0	0	1	1
74355	740–759	Congenital anomalies	Eye	Congenital macular changes	0	0	0	1	0	0	1	1
74356	740–759	Congenital anomalies	Eye	Other retinal changes, congenital	0	0	0	1	0	0	1	1
74357	740–759	Congenital anomalies	Eye	Specified congenital anomalies of optic disc	0	0	0	1	0	0	1	1
74359	740–759	Congenital anomalies	Eye	Other congenital anomalies of posterior segment	0	0	0	1	0	0	1	1
7436	740–759	Congenital anomalies	Eye	CONGN ANOMALIES EYELD LAC SYS&ORBIT	0	0	0	1	0	0	1	1
74361	740–759	Congenital anomalies	Eye	Congenital ptosis	0	0	0	1	0	0	1	1
74362	740–759	Congenital anomalies	Eye	Congenital deformities of eyelids	0	0	0	1	0	0	1	1
74363	740–759	Congenital anomalies	Eye	Other specified congenital anomalies of eyelid	0	0	0	1	0	0	1	1
74364	740–759	Congenital anomalies	Eye	Specified congenital anomalies of lacrimal gland	0	0	0	1	0	0	1	1
74365	740–759	Congenital anomalies	Eye	Specified congenital anomalies of lacrimal passages	0	0	0	1	0	0	1	1
74366	740–759	Congenital anomalies	Eye	Specified congenital anomalies of orbit	0	0	0	1	0	0	1	1
74369	740–759	Congenital anomalies	Eye	Other congenital anomalies of eyelids, lacrimal syster	0	0	0	1	0	0	1	1
7438	740–759	Congenital anomalies	Eye	Other specified anomalies of eye	0	0	0	1	0	0	1	1
7439	740–759	Congenital anomalies	Eye	Unspecified anomaly of eye	0	0	0	1	0	0	1	1
744	740–759	Congenital anomalies	EarFaceNeck	CONGENITAL ANOMALIES EAR FACE&NECK	0	0	0	1	0	0	1	1
7440	740–759	Congenital anomalies	EarFaceNeck	CONGEN ANOM EAR CAUS IMPAIR HEAR	0	0	0	1	0	0	1	1
74400	740–759	Congenital anomalies	EarFaceNeck	Unspecified anomaly of ear with impairment of hearii	0	0	0	1	0	0	1	1
74401	740–759	Congenital anomalies	EarFaceNeck	Absence of external ear	0	0	0	1	0	0	1	1
74402	740–759	Congenital anomalies	EarFaceNeck	Other anomalies of external ear with impairment of h	0	0	0	1	0	0	1	1
74403	740–759	Congenital anomalies	EarFaceNeck	Anomaly of middle ear, except ossicles	0	0	0	1	0	0	1	1
74404	740–759	Congenital anomalies	EarFaceNeck	Anomalies of ear ossicles	0	0	0	1	0	0	1	1
74405	740–759	Congenital anomalies	EarFaceNeck	Anomalies of inner ear	0	0	0	1	0	0	1	1
74409	740–759	Congenital anomalies	EarFaceNeck	Other anomalies of ear causing impairment of hearin	0	0	0	1	0	0	1	1
7441	740–759	Congenital anomalies	EarFaceNeck	Accessory auricle	0	0	0	1	0	0	1	1
7442	740–759	Congenital anomalies	EarFaceNeck	OTHER SPEC CONGENITAL ANOMALIES EAR	0	0	0	1	0	0	1	1
74421	740–759	Congenital anomalies	EarFaceNeck	Absence of ear lobe, congenital	0	0	0	1	0	0	1	1
74422	740–759	Congenital anomalies	EarFaceNeck	Macrotia	0	0	0	1	0	0	1	1
74423	740–759	Congenital anomalies	EarFaceNeck	Microtia	0	0	0	1	0	0	1	1
74424	740–759	Congenital anomalies	EarFaceNeck	Specified anomalies of Eustachian tube	0	0	0	1	0	0	1	1
74429	740–759	Congenital anomalies	EarFaceNeck	Other specified anomalies of ear	0	0	0	1	0	0	1	1
7443	740–759	Congenital anomalies	EarFaceNeck	Unspecified anomaly of ear	0	0	0	1	0	0	1	1
7444	740–759	Congenital anomalies	EarFaceNeck	CNGN BRNCL CLT CYST; PREAURIC SINUS	0	0	0	1	0	0	1	1
74441	740–759	Congenital anomalies	EarFaceNeck	Branchial cleft sinus or fistula	0	0	0	1	0	0	1	1
74442	740–759	Congenital anomalies	EarFaceNeck	Branchial cleft cyst	0	0	0	1	0	0	1	1
74443	740–759	Congenital anomalies	EarFaceNeck	Cervical auricle	0	0	0	1	0	0	1	1
74446	740–759	Congenital anomalies	EarFaceNeck	Preauricular sinus or fistula	0	0	0	1	0	0	1	1
74447	740–759	Congenital anomalies	EarFaceNeck	Preauricular cyst	0	0	0	1	0	0	1	1
74449	740–759	Congenital anomalies	EarFaceNeck	Other branchial cleft cyst or fistula; preauricular sinus	0	0	0	1	0	0	1	1
7445	740–759	Congenital anomalies	EarFaceNeck	Webbing of neck	0	0	0	1	0	0	1	1
7448	740–759	Congenital anomalies	EarFaceNeck	OTH SPEC CONGEN ANOMALIES FACE&NECK	0	0	0	1	0	0	1	1
74481	740–759	Congenital anomalies	EarFaceNeck	Macrocheilia	0	0	0	1	0	0	1	1
74482	740–759	Congenital anomalies	EarFaceNeck	Microcheilia	0	0	0	1	0	0	1	1
74483	740–759	Congenital anomalies	EarFaceNeck	Macrostomia	0	0	0	1	0	0	1	1
74484	740–759	Congenital anomalies	EarFaceNeck	Microstomia	0	0	0	1	0	0	1	1
74489	740–759	Congenital anomalies	EarFaceNeck	Other specified congenital anomalies of face and nec	0	0	0	1	0	0	1	1
7449	740–759	Congenital anomalies	EarFaceNeck	Unspecified congenital anomalies of face and neck	0	0	0	1	0	0	1	1
745	740–759	Congenital anomalies	Circulatory	BULBUS CORDIS&CARD SEPTL CLOS ANOM	0	0	0	1	0	0	1	1
7450	740–759	Congenital anomalies	Circulatory	Common truncus	0	0	0	1	0	0	1	1
7451	740–759	Congenital anomalies	Circulatory	TRANSPOSITION OF GREAT VESSELS	0	0	0	1	0	0	1	1
74510	740–759	Congenital anomalies	Circulatory	Complete transposition of great vessels	0	0	0	1	0	0	1	1
74511	740–759	Congenital anomalies	Circulatory	Double outlet right ventricle	0	0	0	1	0	0	1	1
74512	740–759	Congenital anomalies	Circulatory	Corrected transposition of great vessels	0	0	0	1	0	0	1	1
74519	740–759	Congenital anomalies	Circulatory	Other transposition of great vessels	0	0	0	1	0	0	1	1
7452	740–759	Congenital anomalies	Circulatory	Tetralogy of fallot	0	0	0	1	0	0	1	1
7453	740–759	Congenital anomalies	Circulatory	Common ventricle	0	0	0	1	0	0	1	1
7454	740–759	Congenital anomalies	Circulatory	Ventricular septal defect	0	0	0	1	0	0	1	1
7455	740–759	Congenital anomalies	Circulatory	OSTUM SECUND TYPE ATRIL SEPTL DEFEC	0	0	0	1	0	0	1	1
7456	740–759	Congenital anomalies	Circulatory	ENDOCARDIAL CUSHION DEFECTS	0	0	0	1	0	0	1	1
74560	740–759	Congenital anomalies	Circulatory	Endocardial cushion defect, unspecified type	0	0	0	1	0	0	1	1
74561	740–759	Congenital anomalies	Circulatory	Ostium primum defect	0	0	0	1	0	0	1	1
74569	740–759	Congenital anomalies	Circulatory	Other endocardial cushion defects	0	0	0	1	0	0	1	1
7457	740–759	Congenital anomalies	Circulatory	Cor biloculare	0	0	0	1	0	0	1	1
7458	740–759	Congenital anomalies	Circulatory	Other bulbus cordis anomalies and anomalies of card	0	0	0	1	0	0	1	1
7459	740–759	Congenital anomalies	Circulatory	Unspecified defect of septal closure	0	0	0	1	0	0	1	1
746	740–759	Congenital anomalies	Circulatory	OTHER CONGENITAL ANOMALIES OF HEART	0	0	0	1	0	0	1	1
7460	740–759	Congenital anomalies	Circulatory	CONGEN ANOMALIES PULMONARY VALVE	0	0	0	1	0	0	1	1
74600	740–759	Congenital anomalies	Circulatory	Congenital pulmonary valve anomaly, unspecified	0	0	0	1	0	0	1	1
74601	740–759	Congenital anomalies	Circulatory	Atresia of pulmonary valve, congenital	0	0	0	1	0	0	1	1
74602	740–759	Congenital anomalies	Circulatory	Stenosis of pulmonary valve, congenital	0	0	0	1	0	0	1	1
74609	740–759	Congenital anomalies	Circulatory	Other congenital anomalies of pulmonary valve	0	0	0	1	0	0	1	1
7461	740–759	Congenital anomalies	Circulatory	Tricuspid atresia and stenosis, congenital	0	0	0	1	0	0	1	1
7462	740–759	Congenital anomalies	Circulatory	Ebstein's anomaly	0	0	0	1	0	0	1	1
7463	740–759	Congenital anomalies	Circulatory	Congenital stenosis of aortic valve	0	0	0	1	0	0	1	1
7464	740–759	Congenital anomalies	Circulatory	Congenital insufficiency of aortic valve	0	0	0	1	0	0	1	1
7465	740–759	Congenital anomalies	Circulatory	Congenital mitral stenosis	0	0	0	1	0	0	1	1
7466	740–759	Congenital anomalies	Circulatory	Congenital mitral insufficiency	0	0	0	1	0	0	1	1
7467	740–759	Congenital anomalies	Circulatory	Hypoplastic left heart syndrome	0	0	0	1	0	0	1	1
7468	740–759	Congenital anomalies	Circulatory	OTH SPEC CONGENITAL ANOMALY HEART	0	0	0	1	0	0	1	1
74681	740–759	Congenital anomalies	Circulatory	Subaortic stenosis	0	0	0	1	0	0	1	1
74682	740–759	Congenital anomalies	Circulatory	Cor triatriatum	0	0	0	1	0	0	1	1
74683	740–759	Congenital anomalies	Circulatory	Infundibular pulmonic stenosis	0	0	0	1	0	0	1	1
74684	740–759	Congenital anomalies	Circulatory	Obstructive anomalies of heart, not elsewhere classif	0	0	0	1	0	0	1	1
74685	740–759	Congenital anomalies	Circulatory	Coronary artery anomaly	0	0	0	1	0	0	1	1
74687	740–759	Congenital anomalies	Circulatory	Malposition of heart and cardiac apex	0	0	0	1	0	0	1	1
74689	740–759	Congenital anomalies	Circulatory	Other specified congenital anomalies of heart	0	0	0	1	0	0	1	1
7469	740–759	Congenital anomalies	Circulatory	Unspecified congenital anomaly of heart	0	0	0	1	0	0	1	1
747	740–759	Congenital anomalies	Circulatory	OTH CONGEN ANOMALIES CIRC SYSTEM	0	0	0	1	0	0	1	1
7470	740–759	Congenital anomalies	Circulatory	PATENT DUCTUS ARTERIOSUS	0	0	0	1	0	0	1	1
7471	740–759	Congenital anomalies	Circulatory	COARCTATION OF AORTA	0	0	0	1	0	0	1	1
74710	740–759	Congenital anomalies	Circulatory	Coarctation of aorta (preductal) (postductal)	0	0	0	1	0	0	1	1
74711	740–759	Congenital anomalies	Circulatory	Interruption of aortic arch	0	0	0	1	0	0	1	1
7472	740–759	Congenital anomalies	Circulatory	OTHER CONGENITAL ANOMALY AORTA	0	0	0	1	0	0	1	1
74720	740–759	Congenital anomalies	Circulatory	Anomaly of aorta, unspecified	0	0	0	1	0	0	1	1
74721	740–759	Congenital anomalies	Circulatory	Anomalies of aortic arch	0	0	0	1	0	0	1	1
74722	740–759	Congenital anomalies	Circulatory	Atresia and stenosis of aorta	0	0	0	1	0	0	1	1
74729	740–759	Congenital anomalies	Circulatory	Other anomalies of aorta	0	0	0	1	0	0	1	1
7473	740–759	Congenital anomalies	Circulatory	CONGENITAL ANOMALIES PULMONARY ART	0	0	0	1	0	0	1	1
74731	740–759	Congenital anomalies	Circulatory	Pulmonary artery coarctation and atresia	0	0	0	1	0	0	1	1
74732	740–759	Congenital anomalies	Circulatory	Pulmonary arteriovenous malformation	0	0	0	1	0	0	1	1
74739	740–759	Congenital anomalies	Circulatory	Other anomalies of pulmonary artery and pulmonary	0	0	0	1	0	0	1	1
7474	740–759	Congenital anomalies	Circulatory	CONGENITAL ANOMALIES OF GREAT VEINS	0	0	0	1	0	0	1	1
74740	740–759	Congenital anomalies	Circulatory	Anomaly of great veins, unspecified	0	0	0	1	0	0	1	1
74741	740–759	Congenital anomalies	Circulatory	Total anomalous pulmonary venous connection	0	0	0	1	0	0	1	1
74742	740–759	Congenital anomalies	Circulatory	Partial anomalous pulmonary venous connection	0	0	0	1	0	0	1	1
74749	740–759	Congenital anomalies	Circulatory	Other anomalies of great veins	0	0	0	1	0	0	1	1
7475	740–759	Congenital anomalies	Circulatory	Absence or hypoplasia of umbilical artery	0	0	0	1	0	0	1	1
7476	740–759	Congenital anomalies	Circulatory	OTH CONGN ANOMALY PERIPH VASC SYS	0	0	0	1	0	0	1	1
74760	740–759	Congenital anomalies	Circulatory	Anomaly of the peripheral vascular system, unspecifi	0	0	0	1	0	0	1	1
74761	740–759	Congenital anomalies	Circulatory	Gastrointestinal vessel anomaly	0	0	0	1	0	0	1	1
74762	740–759	Congenital anomalies	Circulatory	Renal vessel anomaly	0	0	0	1	0	0	1	1
74763	740–759	Congenital anomalies	Circulatory	Upper limb vessel anomaly	0	0	0	1	0	0	1	1
74764	740–759	Congenital anomalies	Circulatory	Lower limb vessel anomaly	0	0	0	1	0	0	1	1
74769	740–759	Congenital anomalies	Circulatory	Anomalies of other specified sites of peripheral vascu	0	0	0	1	0	0	1	1
7478	740–759	Congenital anomalies	Circulatory	OTH SPEC CONGN ANOMALIES CIRC SYS	0	0	0	1	0	0	1	1
74781	740–759	Congenital anomalies	Circulatory	Anomalies of cerebrovascular system	0	0	0	1	0	0	1	1
74782	740–759	Congenital anomalies	Circulatory	Spinal vessel anomaly	0	0	0	1	0	0	1	1
74783	740–759	Congenital anomalies	Circulatory	Persistent fetal circulation	0	0	0	1	0	0	1	1
74789	740–759	Congenital anomalies	Circulatory	Other specified anomalies of circulatory system	0	0	0	1	0	0	1	1

7479	740–759	Congenital anomalies	Circulatory	Unspecified anomaly of circulatory system	0	0	0	1	0	0	1	1
748	740–759	Congenital anomalies	Respiratory	CONGEN ANOMALIES RESPIRATORY SYSTEM	0	0	0	1	0	0	1	1
7480	740–759	Congenital anomalies	Respiratory	Choanal atresia	0	0	0	1	0	0	1	1
7481	740–759	Congenital anomalies	Respiratory	Other anomalies of nose	0	0	0	1	0	0	1	1
7482	740–759	Congenital anomalies	Respiratory	Web of larynx	0	0	0	1	0	0	1	1
7483	740–759	Congenital anomalies	Respiratory	Other anomalies of larynx, trachea, and bronchus	0	0	0	1	0	0	1	1
7484	740–759	Congenital anomalies	Respiratory	Congenital cystic lung	0	0	0	1	0	0	1	1
7485	740–759	Congenital anomalies	Respiratory	Agenesis, hypoplasia, and dysplasia of lung	0	0	0	1	0	0	1	1
7486	740–759	Congenital anomalies	Respiratory	CONGENITAL OTHER ANOMALIES OF LUNG	0	0	0	1	0	0	1	1
74860	740–759	Congenital anomalies	Respiratory	Anomaly of lung, unspecified	0	0	0	1	0	0	1	1
74861	740–759	Congenital anomalies	Respiratory	Congenital bronchiectasis	0	0	1	0	0	1	0	1
74869	740–759	Congenital anomalies	Respiratory	Other congenital anomalies of lung	0	0	0	1	0	0	1	1
7488	740–759	Congenital anomalies	Respiratory	Other specified anomalies of respiratory system	0	0	0	1	0	0	1	1
7489	740–759	Congenital anomalies	Respiratory	Unspecified anomaly of respiratory system	0	0	0	1	0	0	1	1
749	740–759	Congenital anomalies	Digestive	CLEFT PALATE AND CLEFT LIP	0	0	0	1	0	0	1	1
7490	740–759	Congenital anomalies	Digestive	CLEFT PALATE	0	0	0	1	0	0	1	1
74900	740–759	Congenital anomalies	Digestive	Cleft palate, unspecified	0	0	0	1	0	0	1	1
74901	740–759	Congenital anomalies	Digestive	Cleft palate, unilateral, complete	0	0	0	1	0	0	1	1
74902	740–759	Congenital anomalies	Digestive	Cleft palate, unilateral, incomplete	0	0	0	1	0	0	1	1
74903	740–759	Congenital anomalies	Digestive	Cleft palate, bilateral, complete	0	0	0	1	0	0	1	1
74904	740–759	Congenital anomalies	Digestive	Cleft palate, bilateral, incomplete	0	0	0	1	0	0	1	1
7491	740–759	Congenital anomalies	Digestive	CLEFT LIP	0	0	0	1	0	0	1	1
74910	740–759	Congenital anomalies	Digestive	Cleft lip, unspecified	0	0	0	1	0	0	1	1
74911	740–759	Congenital anomalies	Digestive	Cleft lip, unilateral, complete	0	0	0	1	0	0	1	1
74912	740–759	Congenital anomalies	Digestive	Cleft lip, unilateral, incomplete	0	0	0	1	0	0	1	1
74913	740–759	Congenital anomalies	Digestive	Cleft lip, bilateral, complete	0	0	0	1	0	0	1	1
74914	740–759	Congenital anomalies	Digestive	Cleft lip, bilateral, incomplete	0	0	0	1	0	0	1	1
7492	740–759	Congenital anomalies	Digestive	CLEFT PALATE WITH CLEFT LIP	0	0	0	1	0	0	1	1
74920	740–759	Congenital anomalies	Digestive	Cleft palate with cleft lip, unspecified	0	0	0	1	0	0	1	1
74921	740–759	Congenital anomalies	Digestive	Cleft palate with cleft lip, unilateral, complete	0	0	0	1	0	0	1	1
74922	740–759	Congenital anomalies	Digestive	Cleft palate with cleft lip, unilateral, incomplete	0	0	0	1	0	0	1	1
74923	740–759	Congenital anomalies	Digestive	Cleft palate with cleft lip, bilateral, complete	0	0	0	1	0	0	1	1
74924	740–759	Congenital anomalies	Digestive	Cleft palate with cleft lip, bilateral, incomplete	0	0	0	1	0	0	1	1
74925	740–759	Congenital anomalies	Digestive	Other combinations of cleft palate with cleft lip	0	0	0	1	0	0	1	1
750	740–759	Congenital anomalies	Digestive	OTH CONGEN ANOM UP ALIMENTARY TRACT	0	0	0	1	0	0	1	1
7500	740–759	Congenital anomalies	Digestive	TONGUE TIE	0	0	0	1	0	0	1	1
7501	740–759	Congenital anomalies	Digestive	OTHER CONGENITAL ANOMALIES TONGUE	0	0	0	1	0	0	1	1
75010	740–759	Congenital anomalies	Digestive	Congenital anomaly of tongue, unspecified	0	0	0	1	0	0	1	1
75011	740–759	Congenital anomalies	Digestive	Aglossia	0	0	0	1	0	0	0	1
75012	740–759	Congenital anomalies	Digestive	Congenital adhesions of tongue	0	0	0	1	0	0	1	1
75013	740–759	Congenital anomalies	Digestive	Fissure of tongue	0	0	0	1	0	0	1	1
75015	740–759	Congenital anomalies	Digestive	Macroglossia	0	0	0	1	0	0	1	1
75016	740–759	Congenital anomalies	Digestive	Microglossia	0	0	0	1	0	0	1	1
75019	740–759	Congenital anomalies	Digestive	Other congenital anomalies of tongue	0	0	0	1	0	0	1	1
7502	740–759	Congenital anomalies	Digestive	OTH CONGN ANOMALIES MOUTH&PHARYNX	0	0	0	1	0	0	1	1
75022	740–759	Congenital anomalies	Digestive	Accessory salivary gland	0	0	0	1	0	0	1	1
75023	740–759	Congenital anomalies	Digestive	Atresia, salivary duct	0	0	0	1	0	0	1	1
75024	740–759	Congenital anomalies	Digestive	Congenital fistula of salivary gland	0	0	0	1	0	0	1	1
75025	740–759	Congenital anomalies	Digestive	Congenital fistula of lip	0	0	0	1	0	0	1	1
75026	740–759	Congenital anomalies	Digestive	Other specified anomalies of mouth	0	0	0	1	0	0	1	1
75027	740–759	Congenital anomalies	Digestive	Diverticulum of pharynx	0	0	0	1	0	0	1	1
75029	740–759	Congenital anomalies	Digestive	Other specified anomalies of pharynx	0	0	0	1	0	0	1	1
7503	740–759	Congenital anomalies	Digestive	Tracheoesophageal fistula, esophageal atresia and st	0	0	0	1	0	0	1	1
7504	740–759	Congenital anomalies	Digestive	Other specified anomalies of esophagus	0	0	0	1	0	0	1	1
7505	740–759	Congenital anomalies	Digestive	Congenital hypertrophic pyloric stenosis	0	0	0	1	0	0	1	1
7506	740–759	Congenital anomalies	Digestive	Congenital hiatus hernia	0	0	0	1	0	0	1	1
7507	740–759	Congenital anomalies	Digestive	Other specified anomalies of stomach	0	0	0	1	0	0	1	1
7508	740–759	Congenital anomalies	Digestive	Other specified anomalies of upper alimentary tract	0	0	0	1	0	0	1	1
7509	740–759	Congenital anomalies	Digestive	Unspecified anomaly of upper alimentary tract	0	0	0	1	0	0	1	1
751	740–759	Congenital anomalies	Digestive	OTH CONGN ANOMALIES DIGESTV SYSTEM	0	0	0	1	0	0	1	1
7510	740–759	Congenital anomalies	Digestive	Meckel's diverticulum	0	0	0	1	0	0	1	1
7511	740–759	Congenital anomalies	Digestive	Atresia and stenosis of small intestine	0	0	0	1	0	0	1	1
7512	740–759	Congenital anomalies	Digestive	Atresia and stenosis of large intestine, rectum, and ar	0	0	0	1	0	0	1	1
7513	740–759	Congenital anomalies	Digestive	Hirschsprung's disease and other congenital functiona	0	0	1	0	0	1	0	1
7514	740–759	Congenital anomalies	Digestive	Anomalies of intestinal fixation	0	0	0	1	0	0	1	1
7515	740–759	Congenital anomalies	Digestive	Other anomalies of intestine	0	0	0	1	0	0	1	1
7516	740–759	Congenital anomalies	Digestive	CONGN ANOMALIES GALLBLADD BDS&LIVER	0	0	0	1	0	0	1	1
75160	740–759	Congenital anomalies	Digestive	Unspecified anomaly of gallbladder, bile ducts, and liv	0	0	0	1	0	0	1	1
75161	740–759	Congenital anomalies	Digestive	Biliary atresia	0	0	0	1	0	0	1	1
75162	740–759	Congenital anomalies	Digestive	Congenital cystic disease of liver	0	0	0	1	0	0	1	1
75169	740–759	Congenital anomalies	Digestive	Other anomalies of gallbladder, bile ducts, and liver	0	0	0	1	0	0	1	1
7517	740–759	Congenital anomalies	Digestive	Anomalies of pancreas	0	0	0	1	0	0	1	1
7518	740–759	Congenital anomalies	Digestive	Other specified anomalies of digestive system	0	0	0	1	0	0	1	1
7519	740–759	Congenital anomalies	Digestive	Unspecified anomaly of digestive system	0	0	0	1	0	0	1	1
752	740–759	Congenital anomalies	Genital	CONGENITAL ANOMALIES GENITAL ORGANS	0	0	0	1	0	0	1	1
7520	740–759	Congenital anomalies	Genital	Anomalies of ovaries	0	0	0	1	0	0	1	1
7521	740–759	Congenital anomalies	Genital	CONGEN ANOMAL FALL TUBE&BROAD LIG	0	0	0	1	0	0	1	1
75210	740–759	Congenital anomalies	Genital	Unspecified anomaly of fallopian tubes and broad liga	0	0	0	1	0	0	1	1
75211	740–759	Congenital anomalies	Genital	Embryonic cyst of fallopian tubes and broad ligament	0	0	0	1	0	0	1	1
75219	740–759	Congenital anomalies	Genital	Other anomalies of fallopian tubes and broad ligame	0	0	0	1	0	0	1	1
7522	740–759	Congenital anomalies	Genital	Doubling of uterus	0	0	0	1	0	0	1	1
7523	740–759	Congenital anomalies	Genital	OTHER CONGENITAL ANOMALY OF UTERUS	0	0	0	1	0	0	1	1
75231	740–759	Congenital anomalies	Genital	Agenesis of uterus	0	0	0	1	0	0	1	1
75232	740–759	Congenital anomalies	Genital	Hypoplasia of uterus	0	0	0	1	0	0	1	1
75233	740–759	Congenital anomalies	Genital	Unicornuate uterus	0	0	0	1	0	0	1	1
75234	740–759	Congenital anomalies	Genital	Bicornuate uterus	0	0	0	1	0	0	1	1
75235	740–759	Congenital anomalies	Genital	Septate uterus	0	0	0	1	0	0	1	1
75236	740–759	Congenital anomalies	Genital	Arcuate uterus	0	0	0	1	0	0	1	1
75239	740–759	Congenital anomalies	Genital	Other anomalies of uterus	0	0	0	1	0	0	1	1
7524	740–759	Congenital anomalies	Genital	CONGN ANOMALIES CERV VAG&EXT FE GNT	0	0	0	1	0	0	1	1
75240	740–759	Congenital anomalies	Genital	Unspecified anomaly of cervix, vagina, and external f	0	0	0	1	0	0	1	1
75241	740–759	Congenital anomalies	Genital	Embryonic cyst of cervix, vagina, and external female	0	0	0	1	0	0	1	1
75242	740–759	Congenital anomalies	Genital	Imperforate hymen	0	0	0	1	0	0	1	1
75243	740–759	Congenital anomalies	Genital	Cervical agenesis	0	0	0	1	0	0	1	1
75244	740–759	Congenital anomalies	Genital	Cervical duplication	0	0	0	1	0	0	1	1
75245	740–759	Congenital anomalies	Genital	Vaginal agenesis	0	0	0	1	0	0	1	1
75246	740–759	Congenital anomalies	Genital	Transverse vaginal septum	0	0	0	1	0	0	1	1
75247	740–759	Congenital anomalies	Genital	Longitudinal vaginal septum	0	0	0	1	0	0	1	1
75249	740–759	Congenital anomalies	Genital	Other anomalies of cervix, vagina, and external fema	0	0	0	1	0	0	1	1
7525	740–759	Congenital anomalies	Genital	UNDESCENDED AND RETRACTILE TESTICLE	0	0	0	1	0	0	1	1
75251	740–759	Congenital anomalies	Genital	Undescended testis	0	0	0	1	0	0	1	1
7526	740–759	Congenital anomalies	Genital	HYPOSPAD&EPISPAD&OTH PENILE ANOM	0	0	0	1	0	0	1	1
75261	740–759	Congenital anomalies	Genital	Hypospadias	0	0	0	1	0	0	1	1
75262	740–759	Congenital anomalies	Genital	Epispadias	0	0	0	1	0	0	1	1
75263	740–759	Congenital anomalies	Genital	Congenital chordee	0	0	0	1	0	0	1	1
75264	740–759	Congenital anomalies	Genital	Micropenis	0	0	0	1	0	0	1	1
75265	740–759	Congenital anomalies	Genital	Hidden penis	0	0	0	1	0	0	1	1
75269	740–759	Congenital anomalies	Genital	Other penile anomalies	0	0	0	1	0	0	1	1
7527	740–759	Congenital anomalies	Genital	Indeterminate sex and pseudohermaphroditism	0	1	0	0	1	0	0	1
7528	740–759	Congenital anomalies	Genital	OTHER SPEC ANOMALIES GENITAL ORGANS	0	0	0	1	0	0	1	1
75281	740–759	Congenital anomalies	Genital	Scrotal transposition	0	0	0	1	0	0	1	1
75289	740–759	Congenital anomalies	Genital	Other specified anomalies of genital organs	0	0	0	1	0	0	1	1
7529	740–759	Congenital anomalies	Genital	Unspecified anomaly of genital organs	0	0	0	1	0	0	1	1
753	740–759	Congenital anomalies	Urinary	CONGENITAL ANOMALIES URINARY SYSTEM	0	0	0	1	0	0	1	1
7530	740–759	Congenital anomalies	Urinary	Renal agenesis and dysgenesis	0	0	0	1	0	0	1	1
7531	740–759	Congenital anomalies	Urinary	CONGENITAL CYSTIC KIDNEY DISEASE	0	0	0	1	0	1	0	1
75310	740–759	Congenital anomalies	Urinary	Cystic kidney disease, unspecified	0	0	0	1	0	0	1	1
75311	740–759	Congenital anomalies	Urinary	Congenital single renal cyst	0	0	0	1	0	0	1	1
75312	740–759	Congenital anomalies	Urinary	Polycystic kidney, unspecified type	0	0	0	1	0	0	1	1
75313	740–759	Congenital anomalies	Urinary	Polycystic kidney, autosomal dominant	1	1	0	0	1	0	0	1
75314	740–759	Congenital anomalies	Urinary	Polycystic kidney, autosomal recessive	1	1	0	0	1	0	0	1
75315	740–759	Congenital anomalies	Urinary	Renal dysplasia	0	0	0	1	0	0	1	1
75316	740–759	Congenital anomalies	Urinary	Medullary cystic kidney	0	0	1	0	0	1	0	1
75317	740–759	Congenital anomalies	Urinary	Medullary sponge kidney	0	0	1	0	0	1	0	1
75319	740–759	Congenital anomalies	Urinary	Other specified cystic kidney disease	0	0	0	1	0	0	1	1
7532	740–759	Congenital anomalies	Urinary	CONGEN OBST DEFEC RENAL PELV&URETER	0	0	0	1	0	0	1	1
75320	740–759	Congenital anomalies	Urinary	Unspecified obstructive defect of renal pelvis and ure	0	0	0	1	0	0	1	1
75321	740–759	Congenital anomalies	Urinary	Congenital obstruction of ureteropelvic junction	0	0	0	1	0	0	1	1
75322	740–759	Congenital anomalies	Urinary	Congenital obstruction of ureterovesical junction	0	0	0	1	0	0	1	1

75323	740–759	Congenital anomalies	Urinary	Congenital ureterocele	0	0	0	1	0	0	1	1
75329	740–759	Congenital anomalies	Urinary	Other obstructive defects of renal pelvis and ureter	0	0	0	1	0	0	1	1
7533	740–759	Congenital anomalies	Urinary	Other specified anomalies of kidney	0	0	0	1	0	0	1	1
7534	740–759	Congenital anomalies	Urinary	Other specified anomalies of ureter	0	0	0	1	0	0	1	1
7535	740–759	Congenital anomalies	Urinary	Exstrophy of urinary bladder	0	0	0	1	0	0	1	1
7536	740–759	Congenital anomalies	Urinary	Atresia and stenosis of urethra and bladder neck	0	0	0	1	0	0	1	1
7537	740–759	Congenital anomalies	Urinary	Anomalies of urachus	0	0	0	1	0	0	1	1
7538	740–759	Congenital anomalies	Urinary	Other specified anomalies of bladder and urethra	0	0	0	1	0	0	1	1
7539	740–759	Congenital anomalies	Urinary	Unspecified anomaly of urinary system	0	0	0	1	0	0	1	1
754	740–759	Congenital anomalies	Musculoskeletal	CERTAIN CONGEN MUSCULOSKEL DEFORM	0	0	0	1	0	0	1	1
7540	740–759	Congenital anomalies	Musculoskeletal	Congenital musculoskeletal deformities of skull, face,	0	0	0	1	0	0	1	1
7541	740–759	Congenital anomalies	Musculoskeletal	Congenital musculoskeletal deformities of sternocleic	0	0	0	1	0	0	1	1
7542	740–759	Congenital anomalies	Musculoskeletal	Congenital musculoskeletal deformities of spine	0	0	0	1	0	0	1	1
7543	740–759	Congenital anomalies	Musculoskeletal	CONGENITAL DISLOCATION OF HIP	0	0	0	1	0	0	1	1
75430	740–759	Congenital anomalies	Musculoskeletal	Congenital dislocation of hip, unilateral	0	0	0	1	0	0	1	1
75431	740–759	Congenital anomalies	Musculoskeletal	Congenital dislocation of hip, bilateral	0	0	0	1	0	0	1	1
75432	740–759	Congenital anomalies	Musculoskeletal	Congenital subluxation of hip, unilateral	0	0	0	1	0	0	1	1
75433	740–759	Congenital anomalies	Musculoskeletal	Congenital subluxation of hip, bilateral	0	0	0	1	0	0	1	1
75435	740–759	Congenital anomalies	Musculoskeletal	Congenital dislocation of one hip with subluxation of c	0	0	0	1	0	0	1	1
7544	740–759	Congenital anomalies	Musculoskeletal	CONGN GENU RECURV&BOW LONG BONE LEG	0	0	0	1	0	0	1	1
75440	740–759	Congenital anomalies	Musculoskeletal	Genu recurvatum	0	0	0	1	0	0	1	1
75441	740–759	Congenital anomalies	Musculoskeletal	Congenital dislocation of knee (with genu recurvatum	0	0	0	1	0	0	1	1
75442	740–759	Congenital anomalies	Musculoskeletal	Congenital bowing of femur	0	0	0	1	0	0	1	1
75443	740–759	Congenital anomalies	Musculoskeletal	Congenital bowing of tibia and fibula	0	0	0	1	0	0	1	1
75444	740–759	Congenital anomalies	Musculoskeletal	Congenital bowing of unspecified long bones of leg	0	0	0	1	0	0	1	1
7545	740–759	Congenital anomalies	Musculoskeletal	CONGENITAL VARUS DEFORMITIES FEET	0	0	0	1	0	0	1	1
75450	740–759	Congenital anomalies	Musculoskeletal	Talipes varus	0	0	0	1	0	0	1	1
75451	740–759	Congenital anomalies	Musculoskeletal	Talipes equinovarus	0	0	0	1	0	0	1	1
75452	740–759	Congenital anomalies	Musculoskeletal	Metatarsus primus varus	0	0	0	1	0	0	1	1
75453	740–759	Congenital anomalies	Musculoskeletal	Metatarsus varus	0	0	0	1	0	0	1	1
75459	740–759	Congenital anomalies	Musculoskeletal	Other varus deformities of feet	0	0	0	1	0	0	1	1
7546	740–759	Congenital anomalies	Musculoskeletal	CONGENITAL VALGUS DEFORMITIES FEET	0	0	0	1	0	0	1	1
75460	740–759	Congenital anomalies	Musculoskeletal	Talipes valgus	0	0	0	1	0	0	1	1
75462	740–759	Congenital anomalies	Musculoskeletal	Talipes calcaneovalgus	0	0	0	1	0	0	1	1
75469	740–759	Congenital anomalies	Musculoskeletal	Other valgus deformities of feet	0	0	0	1	0	0	1	1
7547	740–759	Congenital anomalies	Musculoskeletal	OTHER CONGENITAL DEFORMITY OF FEET	0	0	0	1	0	0	1	1
75470	740–759	Congenital anomalies	Musculoskeletal	Talipes, unspecified	0	0	0	1	0	0	1	1
75471	740–759	Congenital anomalies	Musculoskeletal	Talipes cavus	0	0	0	1	0	0	1	1
75479	740–759	Congenital anomalies	Musculoskeletal	Other deformities of feet	0	0	0	1	0	0	1	1
7548	740–759	Congenital anomalies	Musculoskeletal	OTH SPEC NONTERATOGENIC ANOMALIES	0	0	0	1	0	0	1	1
75481	740–759	Congenital anomalies	Musculoskeletal	Pectus excavatum	0	0	0	1	0	0	1	1
75482	740–759	Congenital anomalies	Musculoskeletal	Pectus carinatum	0	0	0	1	0	0	1	1
75489	740–759	Congenital anomalies	Musculoskeletal	Other specified nonteratogenic anomalies	0	0	0	1	0	0	1	1
755	740–759	Congenital anomalies	Musculoskeletal	OTHER CONGENITAL ANOMALIES OF LIMBS	0	0	0	1	0	0	1	1
7550	740–759	Congenital anomalies	Musculoskeletal	POLYDACTYLY	0	0	1	0	0	1	0	1
75500	740–759	Congenital anomalies	Musculoskeletal	Polydactyly, unspecified digits	0	0	1	0	0	1	0	1
75501	740–759	Congenital anomalies	Musculoskeletal	Polydactyly of fingers	0	0	1	0	0	1	0	1
75502	740–759	Congenital anomalies	Musculoskeletal	Polydactyly of toes	0	0	1	0	0	1	0	1
7551	740–759	Congenital anomalies	Musculoskeletal	SYNDACTYLY	0	0	1	0	0	1	0	1
75510	740–759	Congenital anomalies	Musculoskeletal	Syndactyly of multiple and unspecified sites	0	0	1	0	0	1	0	1
75511	740–759	Congenital anomalies	Musculoskeletal	Syndactyly of fingers without fusion of bone	0	0	1	0	0	1	0	1
75512	740–759	Congenital anomalies	Musculoskeletal	Syndactyly of fingers with fusion of bone	0	0	1	0	0	1	0	1
75513	740–759	Congenital anomalies	Musculoskeletal	Syndactyly of toes without fusion of bone	0	0	1	0	0	1	0	1
75514	740–759	Congenital anomalies	Musculoskeletal	Syndactyly of toes with fusion of bone	0	0	1	0	0	1	0	1
7552	740–759	Congenital anomalies	Musculoskeletal	CONGEN REDUCTION DEFORM UPPER LIMB	0	0	0	1	0	0	1	1
75520	740–759	Congenital anomalies	Musculoskeletal	Unspecified reduction deformity of upper limb	0	0	0	1	0	0	1	1
75521	740–759	Congenital anomalies	Musculoskeletal	Transverse deficiency of upper limb	0	0	0	1	0	0	1	1
75522	740–759	Congenital anomalies	Musculoskeletal	Longitudinal deficiency of upper limb, not elsewhere c	0	0	0	1	0	0	1	1
75523	740–759	Congenital anomalies	Musculoskeletal	Longitudinal deficiency, combined, involving humerus	0	0	0	1	0	0	1	1
75524	740–759	Congenital anomalies	Musculoskeletal	Longitudinal deficiency, humeral, complete or partial (	0	0	0	1	0	0	1	1
75525	740–759	Congenital anomalies	Musculoskeletal	Longitudinal deficiency, radioulnar, complete or partia	0	0	0	1	0	0	1	1
75526	740–759	Congenital anomalies	Musculoskeletal	Longitudinal deficiency, radial, complete or partial (wi	0	0	0	1	0	0	1	1
75527	740–759	Congenital anomalies	Musculoskeletal	Longitudinal deficiency, ulnar, complete or partial (wit	0	0	0	1	0	0	1	1
75528	740–759	Congenital anomalies	Musculoskeletal	Longitudinal deficiency, carpals or metacarpals, comp	0	0	0	1	0	0	1	1
75529	740–759	Congenital anomalies	Musculoskeletal	Longitudinal deficiency, phalanges, complete or partia	0	0	0	1	0	0	1	1
7553	740–759	Congenital anomalies	Musculoskeletal	CONGEN REDUCTION DEFORM LOWER LIMB	0	0	0	1	0	0	1	1
75530	740–759	Congenital anomalies	Musculoskeletal	Unspecified reduction deformity of lower limb,	0	0	0	1	0	0	1	1
75531	740–759	Congenital anomalies	Musculoskeletal	Transverse deficiency of lower limb	0	0	0	1	0	0	1	1
75532	740–759	Congenital anomalies	Musculoskeletal	Longitudinal deficiency of lower limb, not elsewhere c	0	0	0	1	0	0	1	1
75533	740–759	Congenital anomalies	Musculoskeletal	Longitudinal deficiency, combined, involving femur, ti	0	0	0	1	0	0	1	1
75534	740–759	Congenital anomalies	Musculoskeletal	Longitudinal deficiency, femoral, complete or partial (	0	0	0	1	0	0	1	1
75535	740–759	Congenital anomalies	Musculoskeletal	Longitudinal deficiency, tibiofibular, complete or parti	0	0	0	1	0	0	1	1
75536	740–759	Congenital anomalies	Musculoskeletal	Longitudinal deficiency, tibia, complete or partial (with	0	0	0	1	0	0	1	1
75537	740–759	Congenital anomalies	Musculoskeletal	Longitudinal deficiency, fibular, complete or partial (w	0	0	0	1	0	0	1	1
75538	740–759	Congenital anomalies	Musculoskeletal	Longitudinal deficiency, tarsals or metatarsals, compl	0	0	0	1	0	0	1	1
75539	740–759	Congenital anomalies	Musculoskeletal	Longitudinal deficiency, phalanges, complete or partia	0	0	0	1	0	0	1	1
7554	740–759	Congenital anomalies	Musculoskeletal	Reduction deformities, unspecified limb	0	0	0	1	0	0	1	1
7555	740–759	Congenital anomalies	Musculoskeletal	OTH CONGN ANOM UP LIMB-SHLDR GIRDL	0	0	0	1	0	0	1	1
75550	740–759	Congenital anomalies	Musculoskeletal	Unspecified anomaly of upper limb	0	0	0	1	0	0	1	1
75551	740–759	Congenital anomalies	Musculoskeletal	Congenital deformity of clavicle	0	0	0	1	0	0	1	1
75552	740–759	Congenital anomalies	Musculoskeletal	Congenital elevation of scapula	0	0	0	1	0	0	1	1
75553	740–759	Congenital anomalies	Musculoskeletal	Radioulnar synostosis	0	0	0	1	0	0	1	1
75554	740–759	Congenital anomalies	Musculoskeletal	Madelung's deformity	0	0	0	1	0	0	1	1
75555	740–759	Congenital anomalies	Musculoskeletal	Acrocephalosyndactyly	0	0	0	1	0	0	1	1
75556	740–759	Congenital anomalies	Musculoskeletal	Accessory carpal bones	0	0	0	1	0	0	1	1
75557	740–759	Congenital anomalies	Musculoskeletal	Macrodactylia (fingers)	0	0	0	1	0	0	1	1
75558	740–759	Congenital anomalies	Musculoskeletal	Cleft hand, congenital	0	0	0	1	0	0	1	1
75559	740–759	Congenital anomalies	Musculoskeletal	Other anomalies of upper limb, including shoulder gir	0	0	0	1	0	0	1	1
7556	740–759	Congenital anomalies	Musculoskeletal	OTH CONGEN ANOMY LW LIMB-PELV GIRDL	0	0	0	1	0	0	1	1
75560	740–759	Congenital anomalies	Musculoskeletal	Unspecified anomaly of lower limb	0	0	0	1	0	0	1	1
75561	740–759	Congenital anomalies	Musculoskeletal	Coxa valga, congenital	0	0	0	1	0	0	1	1
75562	740–759	Congenital anomalies	Musculoskeletal	Coxa vara, congenital	0	0	0	1	0	0	1	1
75563	740–759	Congenital anomalies	Musculoskeletal	Other congenital deformity of hip (joint)	0	0	0	1	0	0	1	1
75564	740–759	Congenital anomalies	Musculoskeletal	Congenital deformity of knee (joint)	0	0	0	1	0	0	1	1
75565	740–759	Congenital anomalies	Musculoskeletal	Macrodactylia of toes	0	0	0	1	0	0	1	1
75566	740–759	Congenital anomalies	Musculoskeletal	Other anomalies of toes	0	0	0	1	0	0	1	1
75567	740–759	Congenital anomalies	Musculoskeletal	Anomalies of foot, not elsewhere classified	0	0	0	1	0	0	1	1
75569	740–759	Congenital anomalies	Musculoskeletal	Other anomalies of lower limb, including pelvic girdle	0	0	0	1	0	0	1	1
7558	740–759	Congenital anomalies	Musculoskeletal	Other specified anomalies of unspecified limb	0	0	0	1	0	0	1	1
7559	740–759	Congenital anomalies	Musculoskeletal	Unspecified anomaly of unspecified limb	0	0	0	1	0	0	1	1
756	740–759	Congenital anomalies	Musculoskeletal	OTH CONGEN MUSCULOSKEL ANOMALIES	0	0	0	1	0	0	1	1
7560	740–759	Congenital anomalies	Musculoskeletal	Anomalies of skull and face bones	0	0	0	1	0	0	1	1
7561	740–759	Congenital anomalies	Musculoskeletal	CONGENITAL ANOMALIES OF SPINE	0	0	0	1	0	0	1	1
75610	740–759	Congenital anomalies	Musculoskeletal	Anomaly of spine, unspecified	0	0	0	1	0	0	1	1
75611	740–759	Congenital anomalies	Musculoskeletal	Spondylolysis, lumbosacral region	0	0	0	1	0	0	1	1
75612	740–759	Congenital anomalies	Musculoskeletal	Spondylolisthesis	0	0	0	1	0	0	1	1
75613	740–759	Congenital anomalies	Musculoskeletal	Absence of vertebra, congenital	0	0	0	1	0	0	1	1
75614	740–759	Congenital anomalies	Musculoskeletal	Hemivertebra	0	0	0	1	0	0	1	1
75615	740–759	Congenital anomalies	Musculoskeletal	Fusion of spine (vertebra), congenital	0	0	0	1	0	0	1	1
75616	740–759	Congenital anomalies	Musculoskeletal	Klippel-Feil syndrome	0	0	0	1	0	0	1	1
75617	740–759	Congenital anomalies	Musculoskeletal	Spina bifida occulta	0	0	0	1	0	0	1	1
75619	740–759	Congenital anomalies	Musculoskeletal	Other anomalies of spine	0	0	0	1	0	0	1	1
7562	740–759	Congenital anomalies	Musculoskeletal	Cervical rib	0	0	0	1	0	0	1	1
7563	740–759	Congenital anomalies	Musculoskeletal	Other anomalies of ribs and sternum	0	0	0	1	0	0	1	1
7564	740–759	Congenital anomalies	Musculoskeletal	Chondrodystrophy	0	0	1	0	0	1	0	1
7565	740–759	Congenital anomalies	Musculoskeletal	CONGENITAL OSTEODYSTROPHIES	0	0	1	0	0	1	0	1
75650	740–759	Congenital anomalies	Musculoskeletal	Congenital osteodystrophy, unspecified	0	0	1	0	0	1	0	1
75651	740–759	Congenital anomalies	Musculoskeletal	Osteogenesis imperfecta	0	1	0	0	1	0	0	1
75652	740–759	Congenital anomalies	Musculoskeletal	Osteopetrosis	0	1	0	0	1	0	0	1
75653	740–759	Congenital anomalies	Musculoskeletal	Osteopoikilosis	0	1	0	0	1	0	0	1
75654	740–759	Congenital anomalies	Musculoskeletal	Polyostotic fibrous dysplasia of bone	0	0	0	0	0	1	0	1
75655	740–759	Congenital anomalies	Musculoskeletal	Chondroectodermal dysplasia	0	0	1	0	0	1	0	1
75656	740–759	Congenital anomalies	Musculoskeletal	Multiple epiphyseal dysplasia	0	1	0	0	1	0	0	1
75659	740–759	Congenital anomalies	Musculoskeletal	Other osteodystrophies	0	0	1	0	0	1	0	1
7566	740–759	Congenital anomalies	Musculoskeletal	Anomalies of diaphragm	0	0	0	1	0	0	1	1
7567	740–759	Congenital anomalies	Musculoskeletal	CONGENITAL ANOMALY ABDOMINAL WALL	0	0	0	1	0	0	1	1
75670	740–759	Congenital anomalies	Musculoskeletal	Anomaly of abdominal wall, unspecified	0	0	0	1	0	0	1	1
75671	740–759	Congenital anomalies	Musculoskeletal	Prune belly syndrome	0	0	0	1	0	0	1	1
75672	740–759	Congenital anomalies	Musculoskeletal	Omphalocele	0	0	1	0	0	1	0	1

75673	740–759	Congenital anomalies	Musculoskeletal	Gastroschisis	0	0	0	1	0	0	1	1
75679	740–759	Congenital anomalies	Musculoskeletal	Other congenital anomalies of abdominal wall	0	0	0	1	0	0	1	1
7568	740–759	Congenital anomalies	Musculoskeletal	OTH CONGN ANOM MUSC TEND&CNCTV TISS	0	0	0	1	0	0	1	1
75681	740–759	Congenital anomalies	Musculoskeletal	Absence of muscle and tendon	0	0	0	1	0	0	1	1
75683	740–759	Congenital anomalies	Musculoskeletal	Ehlers-Danlos syndrome	0	0	1	0	0	0	1	1
75689	740–759	Congenital anomalies	Musculoskeletal	Other specified anomalies of muscle, tendon, fascia, a	0	0	0	1	0	0	1	1
7569	740–759	Congenital anomalies	Musculoskeletal	Other and unspecified anomalies of musculoskeletal	0	0	0	1	0	0	1	1
757	740–759	Congenital anomalies	Integument	CONGENITAL ANOMALIES THE INTEGUMENT	0	0	0	1	0	0	1	1
7570	740–759	Congenital anomalies	Integument	Hereditary edema of legs	0	1	0	0	1	0	0	1
7571	740–759	Congenital anomalies	Integument	Ichthyosis congenita	0	1	0	0	1	0	0	1
7572	740–759	Congenital anomalies	Integument	Dermatoglyphic anomalies	0	0	0	1	0	0	1	1
7573	740–759	Congenital anomalies	Integument	OTH SPEC CONGENITAL ANOMALIES SKIN	0	0	0	1	0	0	1	1
75731	740–759	Congenital anomalies	Integument	Congenital ectodermal dysplasia	0	1	0	0	1	0	0	1
75732	740–759	Congenital anomalies	Integument	Vascular hamartomas	0	0	0	1	0	0	1	1
75733	740–759	Congenital anomalies	Integument	Congenital pigmentary anomalies of skin	0	0	0	1	0	0	1	1
7574	740–759	Congenital anomalies	Integument	Specified anomalies of hair	0	0	1	0	0	1	0	1
7575	740–759	Congenital anomalies	Integument	Specified anomalies of nails	0	0	1	0	0	1	0	1
7576	740–759	Congenital anomalies	Integument	Specified congenital anomalies of breast	0	0	1	0	0	1	0	1
7578	740–759	Congenital anomalies	Integument	Other specified anomalies of the integument	0	0	0	1	0	0	1	1
7579	740–759	Congenital anomalies	Integument	Unspecified congenital anomaly of the integument	0	0	0	1	0	0	1	1
758	740–759	Congenital anomalies	Chromosomal	CHROMOSOMAL ANOMALIES	1	1	0	0	1	0	0	
7580	740–759	Congenital anomalies	Chromosomal	Down's syndrome	1	1	0	0	1	0	0	
7581	740–759	Congenital anomalies	Chromosomal	Patau's syndrome	1	1	0	0	1	0	0	
7582	740–759	Congenital anomalies	Chromosomal	Edwards' syndrome	1	1	0	0	1	0	0	
7583	740–759	Congenital anomalies	Chromosomal	AUTOSOMAL DELETION SYNDROMES	1	1	0	0	1	0	0	
75831	740–759	Congenital anomalies	Chromosomal	Cri-du-chat syndrome	1	1	0	0	1	0	0	
75832	740–759	Congenital anomalies	Chromosomal	Velo-cardio-facial syndrome	1	1	0	0	1	0	0	
75833	740–759	Congenital anomalies	Chromosomal	Other microdeletions	0	1	0	0	1	0	0	
75839	740–759	Congenital anomalies	Chromosomal	Other autosomal deletions	0	1	0	0	1	0	0	
7584	740–759	Congenital anomalies	Chromosomal	Balanced autosomal translocation in normal individua	1	0	0	1	0	0	1	
7585	740–759	Congenital anomalies	Chromosomal	Other conditions due to autosomal anomalies	1	1	0	0	1	0	0	
7586	740–759	Congenital anomalies	Chromosomal	Gonadal dysgenesis	1	0	1	0	0	1	0	
7587	740–759	Congenital anomalies	Chromosomal	Klinefelter's syndrome	1	1	0	0	1	0	0	
7588	740–759	Congenital anomalies	Chromosomal	OTH COND D/T CHROMOSOME ANOMALIES	0	1	0	0	1	0	0	
75881	740–759	Congenital anomalies	Chromosomal	Other conditions due to sex chromosome anomalies	0	1	0	0	1	0	0	
75889	740–759	Congenital anomalies	Chromosomal	Other conditions due to chromosome anomalies	0	1	0	0	1	0	0	
7589	740–759	Congenital anomalies	Chromosomal	Conditions due to anomaly of unspecified chromosom	0	1	0	0	1	0	0	
759	740–759	Congenital anomalies	Other	OTHER&UNSPEC CONGENITAL ANOMALIES	0	0	0	1	0	0	1	1
7590	740–759	Congenital anomalies	Other	Anomalies of spleen	0	0	0	1	0	0	1	1
7591	740–759	Congenital anomalies	Other	Anomalies of adrenal gland	0	0	0	1	0	0	1	1
7592	740–759	Congenital anomalies	Other	Anomalies of other endocrine glands	0	0	0	1	0	0	1	1
7593	740–759	Congenital anomalies	Other	Situs inversus	0	0	0	1	0	0	1	1
7594	740–759	Congenital anomalies	Other	Conjoined twins	0	0	0	1	0	0	1	1
7595	740–759	Congenital anomalies	Other	Tuberous sclerosis	1	1	0	0	1	0	0	1
7596	740–759	Congenital anomalies	Other	Other hamartoses, not elsewhere classified	0	0	1	0	0	1	0	1
7597	740–759	Congenital anomalies	Multiple	Multiple congenital anomalies, so described	0	0	0	1	0	0	1	1
7598	740–759	Congenital anomalies	Other	OTHER SPECIFIED ANOMALIES	0	0	0	1	0	0	1	1
75981	740–759	Congenital anomalies	Other	Prader-Willi syndrome	1	1	0	0	1	0	0	
75982	740–759	Congenital anomalies	Other	Marfan syndrome	1	1	0	0	1	0	0	
75983	740–759	Congenital anomalies	Other	Fragile X syndrome	1	1	0	0	1	0	0	
75989	740–759	Congenital anomalies	Multiple	Other specified congenital anomalies	0	0	0	1	0	0	1	1
7599	740–759	Congenital anomalies	Other	Congenital anomaly, unspecified	0	0	0	1	0	0	1	1
7612	760-779	Certain conditions originating in the per	NA	Fetus or newborn affected by oligohydramnios	0	0	0	1	0	0	0	
7613	760-779	Certain conditions originating in the per	NA	Fetus or newborn affected by polyhydramnios	0	0	0	1	0	0	0	
7740	760-779	Certain conditions originating in the per	NA	Perinatal jaundice from hereditary hemolytic anemias	0	1	0	0	1	0	0	
7751	760-779	Certain conditions originating in the per	NA	Neonatal diabetes mellitus	0	0	1	0	0	0	1	
7752	760-779	Certain conditions originating in the per	NA	NEONATAL MYASTHENIA GRAVIS	0	0	1	0	0	1	0	
776	760-779	Certain conditions originating in the per	NA	HEMATOLOGICAL DISORDERS OF NEWBORN	0	0	0	1	0	0	1	
7765	760-779	Certain conditions originating in the per	NA	Congenital anemia	0	0	1	0	0	1	0	
7771	760-779	Certain conditions originating in the per	NA	FETAL&NEWBORN MECONIUM OBSTRUCTION	0	0	0	1	0	0	1	
7780	760-779	Certain conditions originating in the per	NA	Hydrops fetalis not due to isoimmunization	0	0	1	0	0	0	1	
7785	760-779	Certain conditions originating in the per	NA	Other and unspecified edema of newborn	0	0	0	1	0	0	0	
7790	760-779	Certain conditions originating in the per	NA	Convulsions in newborn	0	0	1	0	0	1	0	
7792	760-779	Certain conditions originating in the per	NA	Cerebral depression, coma, and other abnormal cereb	0	0	1	0	0	0	0	
78001	780-799	Symptoms, signs, and ill-defined condit	NA	Coma	0	0	0	1	0	0	0	
7803	780-799	Symptoms, signs, and ill-defined condit	NA	CONVULSIONS	0	0	0	1	0	0	1	
78039	780-799	Symptoms, signs, and ill-defined condit	NA	OTHER CONVULSIONS	0	0	0	1	0	0	0	
7810	780-799	Symptoms, signs, and ill-defined condit	NA	ABNORMAL INVOLUNTARY MOVEMENTS	0	0	0	1	0	0	1	
7811	780-799	Symptoms, signs, and ill-defined condit	NA	Disturbances of sensation of smell and taste	0	0	0	0	0	0	1	
78341	780-799	Symptoms, signs, and ill-defined condit	NA	FAILURE TO THRIVE	0	0	0	1	0	0	0	
7891	780-799	Symptoms, signs, and ill-defined condit	NA	Hepatomegaly	0	0	0	0	0	0	1	
7892	780-799	Symptoms, signs, and ill-defined condit	NA	Splenomegaly	0	0	0	0	0	0	1	
7965	780-799	Symptoms, signs, and ill-defined condit	NA	Abnormal finding on antenatal screening	0	0	0	1	0	0	0	
7966	780-799	Symptoms, signs, and ill-defined condit	NA	Nonspecific abnormal findings on neonatal screening	0	0	0	1	0	0	0	
9951	800-999	Injury and poisoning	NA	ANGIONEUROTIC EDEMA NEC	0	0	0	0	0	1	0	
99586	800-999	Injury and poisoning	NA	Malignant hyperthermia	1	1	0	0	1	0	0	
V1362	V01-V89	Supplementary classificaiton of factors	NA	Personal history of other (corrected) congenital malfo	0	0	0	1	0	0	1	
V1363	V01-V89	Supplementary classificaiton of factors	NA	Personal history of (corrected) congenital malformati	0	0	0	1	0	0	1	
V1364	V01-V89	Supplementary classificaiton of factors	NA	Personal history of (corrected) congenital malformati	0	0	0	1	0	0	1	
V1365	V01-V89	Supplementary classificaiton of factors	NA	Personal history of (corrected) congenital malformati	0	0	0	1	0	0	1	
V1366	V01-V89	Supplementary classificaiton of factors	NA	Personal history of (corrected) congenital malformati	0	0	0	1	0	0	1	
V1367	V01-V89	Supplementary classificaiton of factors	NA	Personal history of (corrected) congenital malformati	0	0	0	1	0	0	1	
V1368	V01-V89	Supplementary classificaiton of factors	NA	Personal history of (corrected) congenital malformati	0	0	0	1	0	0	1	
V1369	V01-V89	Supplementary classificaiton of factors	NA	Personal history of other (corrected) congenital malfo	0	0	0	1	0	0	1	
V172	V01-V89	Supplementary classificaiton of factors	NA	Family history of other neurological diseases	0	0	0	0	0	0	1	
V1741	V01-V89	Supplementary classificaiton of factors	NA	Family history of sudden cardiac death [SCD]	0	0	0	0	0	0	1	
V1789	V01-V89	Supplementary classificaiton of factors	NA	Family history of, Other musculoskeletal diseases	0	0	0	0	0	0	1	
V1811	V01-V89	Supplementary classificaiton of factors	NA	Family history of multiple endocrine neoplasia [MEN]	0	0	0	0	0	0	1	
V184	V01-V89	Supplementary classificaiton of factors	NA	Family history of intellectual disabilities	0	0	0	0	0	0	1	
V1851	V01-V89	Supplementary classificaiton of factors	NA	Family history, Colonic polyps	0	0	0	0	0	0	1	
V1861	V01-V89	Supplementary classificaiton of factors	NA	Family history of polycystic kidney	0	0	0	0	0	0	1	
V189	V01-V89	Supplementary classificaiton of factors	NA	Family history of, Genetic disease carrier	0	0	0	0	0	0	1	
V190	V01-V89	Supplementary classificaiton of factors	NA	Family history of blindness or visual loss	0	0	0	0	0	0	1	
V1911	V01-V89	Supplementary classificaiton of factors	NA	Family history of glaucoma	0	0	0	0	0	0	1	
V192	V01-V89	Supplementary classificaiton of factors	NA	Family history of deafness or hearing loss	0	0	0	0	0	0	1	
V195	V01-V89	Supplementary classificaiton of factors	NA	Family history of congenital anomalies	0	0	0	0	0	0	1	
V197	V01-V89	Supplementary classificaiton of factors	NA	Family history of consanguinity	0	0	0	0	0	0	1	
V198	V01-V89	Supplementary classificaiton of factors	NA	Family history of other condition	0	0	0	0	0	0	1	
V263	V01-V89	Supplementary classificaiton of factors	NA	GENETIC COUNSELING AND TESTING	0	0	0	1	0	0	1	
V2631	V01-V89	Supplementary classificaiton of factors	NA	Testing of female for genetic disease carrier status	0	0	0	0	0	0	0	
V2632	V01-V89	Supplementary classificaiton of factors	NA	OTHER GENETIC TESTING OF FEMALE	0	0	0	1	0	0	1	
V2633	V01-V89	Supplementary classificaiton of factors	NA	GENETIC COUNSELING	0	0	0	1	0	0	1	
V2634	V01-V89	Supplementary classificaiton of factors	NA	Testing of male for genetic disease carrier status	0	0	0	0	0	0	0	
V2639	V01-V89	Supplementary classificaiton of factors	NA	OTHER GENETIC TESTING OF MALE	0	0	0	1	0	0	1	
V293	V01-V89	Supplementary classificaiton of factors	NA	Observation for suspected genetic or metabolic condi	0	0	0	1	0	0	1	
V8401	V01-V89	Supplementary classificaiton of factors	NA	Genetic susceptibility to malignant neoplasm of breas	0	0	0	0	0	0	0	
V8402	V01-V89	Supplementary classificaiton of factors	NA	Genetic susceptibility to malignant neoplasm of ovary	0	0	0	0	0	0	0	
V8403	V01-V89	Supplementary classificaiton of factors	NA	Genetic susceptibility to malignant neoplasm of prost	0	0	0	0	0	0	0	
V8404	V01-V89	Supplementary classificaiton of factors	NA	Genetic susceptibility to malignant neoplasm of endo	0	0	0	0	0	0	0	
V8409	V01-V89	Supplementary classificaiton of factors	NA	Genetic susceptibility to other malignant neoplasm	0	0	0	0	0	0	0	
V8481	V01-V89	Supplementary classificaiton of factors	NA	Genetic susceptibility to multiple endocrine neoplasia	0	0	0	0	0	0	0	
V8489	V01-V89	Supplementary classificaiton of factors	NA	GENETIC SUSCEPTIBILITY OTH DISEASE	0	0	0	1	0	0	0	

ICD-10 Code	Category Number	Category Name	Multiple Congenital Anomalies Category	Description	Targeted Testing	Newborn_Definite	Newborn_Probable	Newborn_Possible	Ped_Definite	Ped_Probable	Ped_Possible	MCA, ID,DD, Epilepsy
A810	A00-B99	Certain infectious and parasitic diseases	NA	Creutzfeldt-Jakob disease	0	0	0	0	0	0	0	
A8100	A00-B99	Certain infectious and parasitic diseases	NA	Creutzfeldt-Jakob disease, unspecified	0	0	0	0	0	0	0	
A8101	A00-B99	Certain infectious and parasitic diseases	NA	Variant Creutzfeldt-Jakob disease	0	0	0	0	0	0	0	
A8109	A00-B99	Certain infectious and parasitic diseases	NA	Other Creutzfeldt-Jakob disease	0	0	0	0	0	0	0	
A8182	A00-B99	Certain infectious and parasitic diseases	NA	Gerstmann-Straussler-Scheinker syndrome	1	0	0	0	0	0	0	
A8183	A00-B99	Certain infectious and parasitic diseases	NA	Fatal familial insomnia	1	0	0	0	0	0	0	
C965	C00-D49	Neoplasms	NA	Multifocal and unisystemic Langerhans-cell histiocytosis	0	0	0	1	0	0	0	
C966	C00-D49	Neoplasms	NA	Unifocal Langerhans-cell histiocytosis	0	0	0	1	0	0	0	
C96A	C00-D49	Neoplasms	NA	Histiocytic sarcoma	0	0	0	1	0	0	0	
D512	D50-D89	Diseases of the blood and blood-forming org	NA	TRANSCOBALAMIN II DEFICIENCY	1	1	0	0	0	0	0	
D55	D50-D89	Diseases of the blood and blood-forming org	NA	Anemia due to enzyme disorders	1	1	0	0	1	0	0	
D550	D50-D89	Diseases of the blood and blood-forming org	NA	Anemia due to glucose-6-phosphatedehydrogenase [G6PD] deficiency	1	1	0	0	1	0	0	
D551	D50-D89	Diseases of the blood and blood-forming org	NA	Anemia due to other disorders of glutathione metabolism	1	1	0	0	1	0	0	
D552	D50-D89	Diseases of the blood and blood-forming org	NA	Anemia due to disorders of glycolytic enzymes	1	1	0	0	1	0	0	
D553	D50-D89	Diseases of the blood and blood-forming org	NA	Anemia due to disorders of nucleotide metabolism	1	1	0	0	1	0	0	
D558	D50-D89	Diseases of the blood and blood-forming org	NA	Other anemias due to enzyme disorders	1	1	0	0	1	0	0	
D559	D50-D89	Diseases of the blood and blood-forming org	NA	Anemia due to enzyme disorder, unspecified	1	1	0	0	1	0	0	
D56	D50-D89	Diseases of the blood and blood-forming org	NA	Thalassemia	1	1	0	0	1	0	0	
D560	D50-D89	Diseases of the blood and blood-forming org	NA	Alpha thalassemia	1	1	0	0	1	0	0	
D561	D50-D89	Diseases of the blood and blood-forming org	NA	Beta thalassemia	1	1	0	0	1	0	0	
D562	D50-D89	Diseases of the blood and blood-forming org	NA	Delta-beta thalassemia	1	1	0	0	1	0	0	
D563	D50-D89	Diseases of the blood and blood-forming org	NA	Thalassemia minor	1	1	0	0	1	0	0	
D564	D50-D89	Diseases of the blood and blood-forming org	NA	Hereditary persistence of fetal hemoglobin [HPFH]	1	1	0	0	1	0	0	
D565	D50-D89	Diseases of the blood and blood-forming org	NA	Hemoglobin E-beta thalassemia	1	1	0	0	1	0	0	
D568	D50-D89	Diseases of the blood and blood-forming org	NA	Other thalassemias	1	1	0	0	1	0	0	
D569	D50-D89	Diseases of the blood and blood-forming org	NA	Thalassemia, unspecified	1	1	0	0	1	0	0	
D57	D50-D89	Diseases of the blood and blood-forming org	NA	Sickle-cell disorders	1	1	0	0	1	0	0	
D570	D50-D89	Diseases of the blood and blood-forming org	NA	Hb-SS disease with crisis	1	1	0	0	1	0	0	
D5700	D50-D89	Diseases of the blood and blood-forming org	NA	Hb-SS disease with crisis, unspecified	1	1	0	0	1	0	0	
D5701	D50-D89	Diseases of the blood and blood-forming org	NA	Hb-SS disease with acute chest syndrome	1	1	0	0	1	0	0	
D5702	D50-D89	Diseases of the blood and blood-forming org	NA	Hb-SS disease with splenic sequestration	1	1	0	0	1	0	0	
D571	D50-D89	Diseases of the blood and blood-forming org	NA	Sickle-cell disease without crisis	1	1	0	0	1	0	0	
D572	D50-D89	Diseases of the blood and blood-forming org	NA	Sickle-cell/Hb-C disease	1	1	0	0	1	0	0	
D5720	D50-D89	Diseases of the blood and blood-forming org	NA	Sickle-cell/Hb-C disease without crisis	1	1	0	0	1	0	0	
D5721	D50-D89	Diseases of the blood and blood-forming org	NA	Sickle-cell/Hb-C disease with crisis	1	1	0	0	1	0	0	
D57211	D50-D89	Diseases of the blood and blood-forming org	NA	Sickle-cell/Hb-C disease with acute chest syndrome	1	1	0	0	1	0	0	
D57212	D50-D89	Diseases of the blood and blood-forming org	NA	Sickle-cell/Hb-C disease with splenic sequestration	1	1	0	0	1	0	0	
D57219	D50-D89	Diseases of the blood and blood-forming org	NA	Sickle-cell/Hb-C disease with crisis, unspecified	1	1	0	0	1	0	0	
D573	D50-D89	Diseases of the blood and blood-forming org	NA	Sickle-cell trait	1	0	0	1	0	0	1	
D574	D50-D89	Diseases of the blood and blood-forming org	NA	Sickle-cell thalassemia	1	1	0	0	1	0	0	
D5740	D50-D89	Diseases of the blood and blood-forming org	NA	Sickle-cell thalassemia without crisis	1	1	0	0	1	0	0	
D5741	D50-D89	Diseases of the blood and blood-forming org	NA	Sickle-cell thalassemia with crisis	1	1	0	0	1	0	0	
D57411	D50-D89	Diseases of the blood and blood-forming org	NA	Sickle-cell thalassemia with acutechest syndrome	1	1	0	0	1	0	0	
D57412	D50-D89	Diseases of the blood and blood-forming org	NA	Sickle-cell thalassemia with splenic sequestration	1	1	0	0	1	0	0	
D57419	D50-D89	Diseases of the blood and blood-forming org	NA	Sickle-cell thalassemia with crisis, unspecified	1	1	0	0	1	0	0	
D578	D50-D89	Diseases of the blood and blood-forming org	NA	Other sickle-cell disorders	1	1	0	0	1	0	0	
D5780	D50-D89	Diseases of the blood and blood-forming org	NA	Other sickle-cell disorders without crisis	1	1	0	0	1	0	0	
D5781	D50-D89	Diseases of the blood and blood-forming org	NA	Other sickle-cell disorders with crisis	1	1	0	0	1	0	0	
D57811	D50-D89	Diseases of the blood and blood-forming org	NA	Other sickle-cell disorders with acute chest syndrome	1	1	0	0	1	0	0	
D57812	D50-D89	Diseases of the blood and blood-forming org	NA	Other sickle-cell disorders with splenic sequestration	1	1	0	0	1	0	0	
D57819	D50-D89	Diseases of the blood and blood-forming org	NA	Other sickle-cell disorders with crisis, unspecified	1	1	0	0	1	0	0	
D58	D50-D89	Diseases of the blood and blood-forming org	NA	Other hereditary hemolytic anemias	0	1	0	0	1	0	0	
D580	D50-D89	Diseases of the blood and blood-forming org	NA	Hereditary spherocytosis	1	1	0	0	1	0	0	
D581	D50-D89	Diseases of the blood and blood-forming org	NA	Hereditary elliptocytosis	1	1	0	0	1	0	0	
D582	D50-D89	Diseases of the blood and blood-forming org	NA	Other hemoglobinopathies	1	1	0	0	1	0	0	
D588	D50-D89	Diseases of the blood and blood-forming org	NA	Other specified hereditary hemolytic anemias	1	1	0	0	1	0	0	
D589	D50-D89	Diseases of the blood and blood-forming org	NA	Hereditary hemolytic anemia, unspecified	0	1	0	0	1	0	0	
D595	D50-D89	Diseases of the blood and blood-forming org	NA	Paroxysmal nocturnal hemoglobinuria [Marchiafava-Micheli]	1	1	0	0	1	0	0	
D61	D50-D89	Diseases of the blood and blood-forming org	NA	Other aplastic anemias and other bone marrow failure syndromes	0	0	0	1	0	0	1	
D610	D50-D89	Diseases of the blood and blood-forming org	NA	Constitutional aplastic anemia	0	0	1	0	0	1	0	
D6101	D50-D89	Diseases of the blood and blood-forming org	NA	Constitutional (pure) red blood cell aplasia	0	0	1	0	0	1	0	
D6109	D50-D89	Diseases of the blood and blood-forming org	NA	Other constitutional aplastic anemia	0	0	0	1	0	0	1	
D613	D50-D89	Diseases of the blood and blood-forming org	NA	Idiopathic aplastic anemia	0	0	0	1	0	0	1	
D618	D50-D89	Diseases of the blood and blood-forming org	NA	Other specified aplastic anemias and other bone marrow failure syndromes	0	0	0	1	0	0	1	
D6189	D50-D89	Diseases of the blood and blood-forming org	NA	Other specified aplastic anemias and other bone marrow failure syndromes	0	0	0	1	0	0	1	
D619	D50-D89	Diseases of the blood and blood-forming org	NA	Aplastic anemia, unspecified	0	0	0	1	0	0	1	
D640	D50-D89	Diseases of the blood and blood-forming org	NA	Hereditary sideroblastic anemia	1	1	0	0	1	0	0	
D643	D50-D89	Diseases of the blood and blood-forming org	NA	OTHER SIDEROBLASTIC ANEMIAS	0	0	0	0	0	0	1	
D644	D50-D89	Diseases of the blood and blood-forming org	NA	Congenital dyserythropoietic anemia	1	1	0	0	1	0	0	
D66	D50-D89	Diseases of the blood and blood-forming org	NA	Hereditary factor VIII deficiency	1	1	0	0	1	0	0	
D67	D50-D89	Diseases of the blood and blood-forming org	NA	Hereditary factor IX deficiency	0	1	0	0	1	0	0	
D680	D50-D89	Diseases of the blood and blood-forming org	NA	Von Willebrand's disease	1	1	0	0	1	0	0	
D681	D50-D89	Diseases of the blood and blood-forming org	NA	Hereditary factor XI deficiency	1	1	0	0	1	0	0	
D682	D50-D89	Diseases of the blood and blood-forming org	NA	Hereditary deficiency of other clotting factors	0	1	0	0	1	0	0	
D6852	D50-D89	Diseases of the blood and blood-forming org	NA	Prothrombin gene mutation	1	1	0	0	1	0	0	
D6859	D50-D89	Diseases of the blood and blood-forming org	NA	OTHER PRIMARY THROMBOPHILIA	0	0	0	0	0	1	0	
D688	D50-D89	Diseases of the blood and blood-forming org	NA	OTHER SPECIFIED COAGULATION DEFECTS	1	0	0	1	0	0	1	
D689	D50-D89	Diseases of the blood and blood-forming org	NA	COAGULATION DEFECT UNSPECIFIED	0	0	0	1	0	0	1	
D691	D50-D89	Diseases of the blood and blood-forming org	NA	Qualitative platelet defects	0	0	1	0	0	1	0	
D6942	D50-D89	Diseases of the blood and blood-forming org	NA	Congenital and hereditary thrombocytopenia purpura	0	0	1	0	0	1	0	
D6949	D50-D89	Diseases of the blood and blood-forming org	NA	Other primary thrombocytopenia	0	0	1	0	0	1	0	
D700	D50-D89	Diseases of the blood and blood-forming org	NA	Congenital agranulocytosis	0	0	1	0	0	1	0	
D704	D50-D89	Diseases of the blood and blood-forming org	NA	Cyclic neutropenia	0	0	1	0	0	1	0	
D71	D50-D89	Diseases of the blood and blood-forming org	NA	Functional disorders of polymorphonuclear neutrophils	0	0	1	0	0	1	0	
D720	D50-D89	Diseases of the blood and blood-forming org	NA	Genetic anomalies of leukocytes	0	1	0	0	1	0	0	
D740	D50-D89	Diseases of the blood and blood-forming org	NA	Congenital methemoglobinemia	1	1	0	0	1	0	0	
D748	D50-D89	Diseases of the blood and blood-forming org	NA	OTHER METHEMOGLOBINEMIAS	1	0	1	0	0	0	1	
D749	D50-D89	Diseases of the blood and blood-forming org	NA	METHEMOGLOBINEMIA UNSPECIFIED	1	0	1	0	0	0	1	
D750	D50-D89	Diseases of the blood and blood-forming org	NA	Familial erythrocytosis	1	1	0	0	1	0	0	
D761	D50-D89	Diseases of the blood and blood-forming org	NA	Hemophagocytic lymphohistiocytosis	0	1	0	0	1	0	0	
D80	D50-D89	Diseases of the blood and blood-forming org	NA	Immunodeficiency with predominantly antibody defects	0	1	0	0	1	0	0	
D800	D50-D89	Diseases of the blood and blood-forming org	NA	Hereditary hypogammaglobulinemia	0	1	0	0	1	0	0	
D801	D50-D89	Diseases of the blood and blood-forming org	NA	Nonfamilial hypogammaglobulinemia	0	0	0	0	0	1	0	
D802	D50-D89	Diseases of the blood and blood-forming org	NA	Selective deficiency of immunoglobulin A [IgA]	0	0	0	0	0	0	1	
D803	D50-D89	Diseases of the blood and blood-forming org	NA	Selective deficiency of immunoglobulin G [IgG] subclasses	0	0	0	0	0	0	1	
D804	D50-D89	Diseases of the blood and blood-forming org	NA	Selective deficiency of immunoglobulin M [IgM]	0	0	0	0	0	1	0	
D805	D50-D89	Diseases of the blood and blood-forming org	NA	Immunodeficiency with increased immunoglobulin M [IgM]	0	0	1	0	0	1	0	
D806	D50-D89	Diseases of the blood and blood-forming org	NA	Antibody deficiency with near-normal immunoglobulins or with hyperimmunoglobulin	0	0	0	0	0	1	0	
D808	D50-D89	Diseases of the blood and blood-forming org	NA	Other immunodeficiencies with predominantly antibody defects	0	1	0	0	1	0	0	
D809	D50-D89	Diseases of the blood and blood-forming org	NA	Immunodeficiency with predominantly antibody defects, unspecified	0	1	0	0	1	0	0	
D810	D50-D89	Diseases of the blood and blood-forming org	NA	Severe combined immunodeficiency [SCID] with reticular dysgenesis	0	1	0	0	1	0	0	
D811	D50-D89	Diseases of the blood and blood-forming org	NA	Severe combined immunodeficiency [SCID] with low T- and B-cell numbers	1	1	0	0	1	0	0	
D812	D50-D89	Diseases of the blood and blood-forming org	NA	Severe combined immunodeficiency [SCID] with low or normal B-cell numbers	1	1	0	0	1	0	0	
D813	D50-D89	Diseases of the blood and blood-forming org	NA	ADENOSINE DEAMINASE DEFICIENCY	1	1	0	0	1	0	0	
D814	D50-D89	Diseases of the blood and blood-forming org	NA	Nezelof's syndrome	0	1	0	0	1	0	0	
D816	D50-D89	Diseases of the blood and blood-forming org	NA	MAJ HISTOCOMPAT CMPLX CLASS I DEF	1	1	0	0	1	0	0	
D81810	D50-D89	Diseases of the blood and blood-forming org	NA	BIOTINIDASE DEFICIENCY	1	1	0	0	1	0	0	
D81818	D50-D89	Diseases of the blood and blood-forming org	NA	OTH BIOTIN-DEPEND CARBOXYLASE DEFIC	1	1	0	0	1	0	0	
D81819	D50-D89	Diseases of the blood and blood-forming org	NA	BIOTIN-DEPEND CARBOXYLASE DEFIC UNS	1	1	0	0	1	0	0	
D8189	D50-D89	Diseases of the blood and blood-forming org	NA	OTHER COMBINED IMMUNODEFICIENES	0	1	0	0	1	0	0	
D819	D50-D89	Diseases of the blood and blood-forming org	NA	Combined immunodeficiency, unspecified	0	1	0	0	1	0	0	
D82	D50-D89	Diseases of the blood and blood-forming org	NA	Immunodeficiency associated with other major defects	0	1	0	0	1	0	0	
D820	D50-D89	Diseases of the blood and blood-forming org	NA	Wiskott-Aldrich syndrome	0	1	0	0	1	0	0	
D821	D50-D89	Diseases of the blood and blood-forming org	NA	Di George's syndrome	1	1	0	0	1	0	0	
D822	D50-D89	Diseases of the blood and blood-forming org	NA	Immunodeficiency with short-limbedstature	0	1	0	0	1	0	0	
D823	D50-D89	Diseases of the blood and blood-forming org	NA	Immunodeficiency following hereditary defective response to Epstein-Barr virus	0	1	0	0	1	0	0	
D824	D50-D89	Diseases of the blood and blood-forming org	NA	Hyperimmunoglobulin E [IgE] syndrome	1	0	0	0	0	1	0	
D828	D50-D89	Diseases of the blood and blood-forming org	NA	Immunodeficiency associated with other specified major defects	0	0	1	0	0	1	0	
D829	D50-D89	Diseases of the blood and blood-forming org	NA	Immunodeficiency associated with major defect, unspecified	0	0	1	0	0	1	0	
D83	D50-D89	Diseases of the blood and blood-forming org	NA	Common variable immunodeficiency	0	0	0	0	0	1	0	
D830	D50-D89	Diseases of the blood and blood-forming org	NA	Common variable immunodeficiency with predominant abnormalities of B-cell numbe	0	0	0	0	0	1	0	
D831	D50-D89	Diseases of the blood and blood-forming org	NA	Common variable immunodeficiency with predominant immunoregulatory T-cell disor	0	0	0	0	0	1	0	
D832	D50-D89	Diseases of the blood and blood-forming org	NA	Common variable immunodeficiency with autoantibodies to B- or T-cells	0	0	0	0	0	1	0	
D838	D50-D89	Diseases of the blood and blood-forming org	NA	Other common variable immunodeficiencies	0	0	0	0	0	1	0	
D839	D50-D89	Diseases of the blood and blood-forming org	NA	Common variable immunodeficiency,unspecified	0	0	0	0	0	1	0	
D840	D50-D89	Diseases of the blood and blood-forming org	NA	LYMPHOCYTE FUNCT ANTIGEN-1 DEFECT	1	1	0	0	1	0	0	
D841	D50-D89	Diseases of the blood and blood-forming org	NA	Defects in the complement system	1	1	0	0	1	0	0	
D848	D50-D89	Diseases of the blood and blood-forming org	NA	OTHER SPECIFIED IMMUNODEFICIENCIES	0	0	1	0	0	1	0	
D849	D50-D89	Diseases of the blood and blood-forming org	NA	IMMUNODEFICIENCY UNSPECIFIED	0	0	1	0	0	1	0	
D894	D50-D89	Diseases of the blood and blood-forming org	NA	Mast cell activation syndrome andrelated disorders	0	0	0	1	0	0	1	
D8942	D50-D89	Diseases of the blood and blood-forming org	NA	Idiopathic mast cell activation syndrome	0	0	0	1	0	0	1	
D8982	D50-D89	Diseases of the blood and blood-forming org	NA	Autoimmune lymphoproliferative syndrome [ALPS]	0	1	0	0	1	0	0	
D899	D50-D89	Diseases of the blood and blood-forming org	NA	Disorder involving the immune mechanism, unspecified	0	0	1	0	0	1	0	
E030	E00-E89	Endocrine, nutritional and metabolic disease	NA	Congenital hypothyroidism with diffuse goiter	0	0	0	1	0	0	1	
E031	E00-E89	Endocrine, nutritional and metabolic disease	NA	Congenital hypothyroidism withoutgoiter	0	0	0	1	0	0	1	
E071	E00-E89	Endocrine, nutritional and metabolic disease	NA	Dyshormogenetic goiter	1	1	0	0	1	0	0	
E15	E00-E89	Endocrine, nutritional and metabolic disease	NA	Nondiabetic hypoglycemic coma	0	0	0	1	0	0	1	
E201	E00-E89	Endocrine, nutritional and metabolic disease	NA	PSEUDOHYPOPARATHYROIDISM	0	0	0	0	1	0	0	
E209	E00-E89	Endocrine, nutritional and metabolic disease	NA	Hypoparathyroidism, unspecified	0	0	0	1	0	0	1	
E232	E00-E89	Endocrine, nutritional and metabolic disease	NA	Diabetes insipidus	0	0	0	1	0	0	1	
E250	E00-E89	Endocrine, nutritional and metabolic disease	NA	Congenital adrenogenital disordersassociated with enzyme deficiency	1	1	0	0	1	0	0	
E2681	E00-E89	Endocrine, nutritional and metabolic disease	NA	Bartter's syndrome	0	1	0	0	1	0	0	
E271	E00-E89	Endocrine, nutritional and metabolic disease	NA	Primary adrenocortical insufficiency	0	0	0	1	0	0	1	
E2749	E00-E89	Endocrine, nutritional and metabolic disease	NA	Other adrenocortical insufficiency	0	0	0	1	0	0	1	
E300	E00-E89	Endocrine, nutritional and metabolic disease	NA	Delayed puberty	0	0	0	0	0	0	1	
E312	E00-E89	Endocrine, nutritional and metabolic disease	NA	Multiple endocrine neoplasia [MEN]syndromes	1	1	0	0	1	0	0	
E3120	E00-E89	Endocrine, nutritional and metabolic disease	NA	Multiple endocrine neoplasia [MEN]syndrome, unspecified	1	1	0	0	1	0	0	
E3121	E00-E89	Endocrine, nutritional and metabolic disease	NA	Multiple endocrine neoplasia [MEN] type I	1	1	0	0	1	0	0	
E3122	E00-E89	Endocrine, nutritional and metabolic disease	NA	Multiple endocrine neoplasia [MEN] type IIA	1	1	0	0	1	0	0	
E3123	E00-E89	Endocrine, nutritional and metabolic disease	NA	Multiple endocrine neoplasia [MEN] type IIB	1	1	0	0	1	0	0	
E343	E00-E89	Endocrine, nutritional and metabolic disease	NA	Short stature due to endocrine disorder	0	0	0	0	0	0	1	
E345	E00-E89	Endocrine, nutritional and metabolic disease	NA	Androgen insensitivity syndrome	1	1	0	0	1	0	0	
E3450	E00-E89	Endocrine, nutritional and metabolic disease	NA	Androgen insensitivity syndrome, unspecified	1	1	0	0	1	0	0	
E3451	E00-E89	Endocrine, nutritional and metabolic disease	NA	Complete androgen insensitivity syndrome	1	1	0	0	1	0	0	
E3452	E00-E89	Endocrine, nutritional and metabolic disease	NA	Partial androgen insensitivity syndrome	1	1	0	0	1	0	0	
E700	E00-E89	Endocrine, nutritional and metabolic disease	NA	Classical phenylketonuria	1	1	0	0	1	0	0	
E701	E00-E89	Endocrine, nutritional and metabolic disease	NA	Other hyperphenylalaninemias	0	1	0	0	1	0	0	
E702	E00-E89	Endocrine, nutritional and metabolic disease	NA	Disorders of tyrosine metabolism	0	1	0	0	1	0	0	
E7020	E00-E89	Endocrine, nutritional and metabolic disease	NA	Disorder of tyrosine metabolism, unspecified	0	1	0	0	1	0	0	
E7021	E00-E89	Endocrine, nutritional and metabolic disease	NA	Tyrosinemia	1	1	0	0	1	0	0	
E7029	E00-E89	Endocrine, nutritional and metabolic disease	NA	Other disorders of tyrosine metabolism	0	1	0	0	1	0	0	
E703	E00-E89	Endocrine, nutritional and metabolic disease	NA	Albinism	1	1	0	0	1	0	0	
E7030	E00-E89	Endocrine, nutritional and metabolic disease	NA	Albinism, unspecified	0	1	0	0	1	0	0	
E7031	E00-E89	Endocrine, nutritional and metabolic disease	NA	Ocular albinism	1	1	0	0	1	0	0	
E70310	E00-E89	Endocrine, nutritional and metabolic disease	NA	X-linked ocular albinism	1	1	0	0	1	0	0	
E70311	E00-E89	Endocrine, nutritional and metabolic disease	NA	Autosomal recessive ocular albinism	1	1	0	0	1	0	0	
E70318	E00-E89	Endocrine, nutritional and metabolic disease	NA	Other ocular albinism	0	1	0	0	1	0	0	
E70319	E00-E89	Endocrine, nutritional and metabolic disease	NA	Ocular albinism, unspecified	0	1	0	0	1	0	0	
E7032	E00-E89	Endocrine, nutritional and metabolic disease	NA	Oculocutaneous albinism	1	1	0	0	1	0	0	
E70320	E00-E89	Endocrine, nutritional and metabolic disease	NA	Tyrosinase negative oculocutaneousalbinism	1	1	0	0	1	0	0	
E70321	E00-E89	Endocrine, nutritional and metabolic disease	NA	Tyrosinase positive oculocutaneousalbinism	0	1	0	0	1	0	0	
E70328	E00-E89	Endocrine, nutritional and metabolic disease	NA	Other oculocutaneous albinism	1	1	0	0	1	0	0	
E70329	E00-E89	Endocrine, nutritional and metabolic disease	NA	Oculocutaneous albinism, unspecified	1	1	0	0	1	0	0	
E7033	E00-E89	Endocrine, nutritional and metabolic disease	NA	Albinism with hematologic abnormality	0	1	0	0	1	0	0	
E70330	E00-E89	Endocrine, nutritional and metabolic disease	NA	Chediak-Higashi syndrome	1	1	0	0	1	0	0	
E70331	E00-E89	Endocrine, nutritional and metabolic disease	NA	Hermansky-Pudlak syndrome	1	1	0	0	1	0	0	
E70338	E00-E89	Endocrine, nutritional and metabolic disease	NA	Other albinism with hematologic abnormality	0	1	0	0	1	0	0	
E70339	E00-E89	Endocrine, nutritional and metabolic disease	NA	Albinism with hematologic abnormality, unspecified	0	1	0	0	1	0	0	
E7039	E00-E89	Endocrine, nutritional and metabolic disease	NA	Other specified albinism	0	1	0	0	1	0	0	
E704	E00-E89	Endocrine, nutritional and metabolic disease	NA	Disorders of histidine metabolism	1	1	0	0	1	0	0	
E7040	E00-E89	Endocrine, nutritional and metabolic disease	NA	Disorders of histidine metabolism,unspecified	1	1	0	0	1	0	0	
E7041	E00-E89	Endocrine, nutritional and metabolic disease	NA	Histidinemia	1	1	0	0	1	0	0	
E7049	E00-E89	Endocrine, nutritional and metabolic disease	NA	Other disorders of histidine metabolism	1	1	0	0	1	0	0	
E705	E00-E89	Endocrine, nutritional and metabolic disease	NA	Disorders of tryptophan metabolism	0	1	0	0	1	0	0	
E708	E00-E89	Endocrine, nutritional and metabolic disease	NA	Other disorders of aromatic amino-acid metabolism	0	1	0	0	1	0	0	
E709	E00-E89	Endocrine, nutritional and metabolic disease	NA	Disorder of aromatic amino-acid metabolism, unspecified	0	1	0	0	1	0	0	
E71	E00-E89	Endocrine, nutritional and metabolic disease	NA	Disorders of branched-chain amino-acid metabolism and fatty-acid metabolism	1	1	0	0	1	0	0	
E710	E00-E89	Endocrine, nutritional and metabolic disease	NA	Maple-syrup-urine disease	1	1	0	0	1	0	0	
E711	E00-E89	Endocrine, nutritional and metabolic disease	NA	Other disorders of branched-chainamino-acid metabolism	0	1	0	0	1	0	0	
E7111	E00-E89	Endocrine, nutritional and metabolic disease	NA	Branched-chain organic acidurias	1	1	0	0	1	0	0	
E71110	E00-E89	Endocrine, nutritional and metabolic disease	NA	Isovaleric acidemia	1	1	0	0	1	0	0	
E71111	E00-E89	Endocrine, nutritional and metabolic disease	NA	3-methylglutaconic aciduria	0	0	1	0	0	1	0	
E71118	E00-E89	Endocrine, nutritional and metabolic disease	NA	Other branched-chain organic acidurias	0	1	0	0	1	0	0	
E7112	E00-E89	Endocrine, nutritional and metabolic disease	NA	Disorders of propionate metabolism	1	1	0	0	1	0	0	
E71120	E00-E89	Endocrine, nutritional and metabolic disease	NA	Methylmalonic acidemia	1	1	0	0	1	0	0	
E71121	E00-E89	Endocrine, nutritional and metabolic disease	NA	Propionic acidemia	1	1	0	0	1	0	0	
E71128	E00-E89	Endocrine, nutritional and metabolic disease	NA	Other disorders of propionate metabolism	0	1	0	0	1	0	0	
E7119	E00-E89	Endocrine, nutritional and metabolic disease	NA	Other disorders of branched-chainamino-acid metabolism	0	1	0	0	1	0	0	
E712	E00-E89	Endocrine, nutritional and metabolic disease	NA	Disorder of branched-chain amino-acid metabolism, unspecified	0	1	0	0	1	0	0	
E713	E00-E89	Endocrine, nutritional and metabolic disease	NA	Disorders of fatty-acid metabolism	0	1	0	0	1	0	0	
E7130	E00-E89	Endocrine, nutritional and metabolic disease	NA	Disorder of fatty-acid metabolism,unspecified	0	1	0	0	1	0	0	
E7131	E00-E89	Endocrine, nutritional and metabolic disease	NA	Disorders of fatty-acid oxidation	0	1	0	0	1	0	0	
E71310	E00-E89	Endocrine, nutritional and metabolic disease	NA	Long chain/very long chain acyl CoA dehydrogenase deficiency	1	1	0	0	1	0	0	
E71311	E00-E89	Endocrine, nutritional and metabolic disease	NA	Medium chain acyl CoA dehydrogenase deficiency	1	1	0	0	1	0	0	
E71312	E00-E89	Endocrine, nutritional and metabolic disease	NA	Short chain acyl CoA dehydrogenasedeficiency	1	1	0	0	1	0	0	
E71313	E00-E89	Endocrine, nutritional and metabolic disease	NA	Glutaric aciduria type II	1	1	0	0	1	0	0	
E71314	E00-E89	Endocrine, nutritional and metabolic disease	NA	Muscle carnitine palmitoyltransferase deficiency	1	1	0	0	1	0	0	
E71318	E00-E89	Endocrine, nutritional and metabolic disease	NA	Other disorders of fatty-acid oxidation	0	1	0	0	1	0	0	
E7132	E00-E89	Endocrine, nutritional and metabolic disease	NA	Disorders of ketone metabolism	0	1	0	0	1	0	0	
E7139	E00-E89	Endocrine, nutritional and metabolic disease	NA	Other disorders of fatty-acid metabolism	0	1	0	0	1	0	0	
E714	E00-E89	Endocrine, nutritional and metabolic disease	NA	Disorders of carnitine metabolism	0	1	0	0	1	0	0	
E7140	E00-E89	Endocrine, nutritional and metabolic disease	NA	Disorder of carnitine metabolism,unspecified	0	1	0	0	1	0	0	
E7141	E00-E89	Endocrine, nutritional and metabolic disease	NA	Primary carnitine deficiency	0	1	0	0	1	0	0	
E7142	E00-E89	Endocrine, nutritional and metabolic disease	NA	Carnitine deficiency due to inbornerrors of metabolism	0	1	0	0	1	0	0	
E7144	E00-E89	Endocrine, nutritional and metabolic disease	NA	Other secondary carnitine deficiency	0	0	1	0	0	1	0	
E71440	E00-E89	Endocrine, nutritional and metabolic disease	NA	Ruvalcaba-Myhre-Smith syndrome	1	1	0	0	1	0	0	
E71448	E00-E89	Endocrine, nutritional and metabolic disease	NA	Other secondary carnitine deficiency	0	0	1	0	0	1	0	
E715	E00-E89	Endocrine, nutritional and metabolic disease	NA	Peroxisomal disorders	0	1	0	0	1	0	0	
E7150	E00-E89	Endocrine, nutritional and metabolic disease	NA	Peroxisomal disorder, unspecified	0	1	0	0	1	0	0	
E7151	E00-E89	Endocrine, nutritional and metabolic disease	NA	Disorders of peroxisome biogenesis	0	1	0	0	1	0	0	
E71510	E00-E89	Endocrine, nutritional and metabolic disease	NA	Zellweger syndrome	0	1	0	0	1	0	0	
E71511	E00-E89	Endocrine, nutritional and metabolic disease	NA	Neonatal adrenoleukodystrophy	1	1	0	0	1	0	0	
E71518	E00-E89	Endocrine, nutritional and metabolic disease	NA	Other disorders of peroxisome biogenesis	0	1	0	0	1	0	0	
E7152	E00-E89	Endocrine, nutritional and metabolic disease	NA	X-linked adrenoleukodystrophy	1	1	0	0	1	0	0	

E71520	E00-E89	Endocrine, nutritional and metabolic disease	NA	Childhood cerebral X-linked adrenoleukodystrophy	1	1	0	0	1	0	0	
E71521	E00-E89	Endocrine, nutritional and metabolic disease	NA	Adolescent X-linked adrenoleukodystrophy	1	1	0	0	1	0	0	
E71522	E00-E89	Endocrine, nutritional and metabolic disease	NA	Adrenomyeloneuropathy	1	1	0	0	1	0	0	
E71528	E00-E89	Endocrine, nutritional and metabolic disease	NA	Other X-linked adrenoleukodystrophy	0	1	0	0	1	0	0	
E71529	E00-E89	Endocrine, nutritional and metabolic disease	NA	X-linked adrenoleukodystrophy, unspecified type	1	1	0	0	1	0	0	
E7153	E00-E89	Endocrine, nutritional and metabolic disease	NA	Other group 2 peroxisomal disorders	0	1	0	0	1	0	0	
E7154	E00-E89	Endocrine, nutritional and metabolic disease	NA	Other peroxisomal disorders	0	1	0	0	1	0	0	
E71540	E00-E89	Endocrine, nutritional and metabolic disease	NA	Rhizomelic chondrodysplasia punctata	1	1	0	0	1	0	0	
E71541	E00-E89	Endocrine, nutritional and metabolic disease	NA	Zellweger-like syndrome	0	1	0	0	1	0	0	
E71542	E00-E89	Endocrine, nutritional and metabolic disease	NA	Other group 3 peroxisomal disorders	0	1	0	0	1	0	0	
E71548	E00-E89	Endocrine, nutritional and metabolic disease	NA	Other peroxisomal disorders	0	1	0	0	1	0	0	
E72	E00-E89	Endocrine, nutritional and metabolic disease	NA	Other disorders of amino-acid metabolism	0	1	0	0	1	0	0	
E720	E00-E89	Endocrine, nutritional and metabolic disease	NA	Disorders of amino-acid transport	0	1	0	0	1	0	0	
E7200	E00-E89	Endocrine, nutritional and metabolic disease	NA	Disorders of amino-acid transport,unspecified	0	1	0	0	1	0	0	
E7201	E00-E89	Endocrine, nutritional and metabolic disease	NA	Cystinuria	1	1	0	0	1	0	0	
E7202	E00-E89	Endocrine, nutritional and metabolic disease	NA	Hartnup's disease	1	1	0	0	1	0	0	
E7203	E00-E89	Endocrine, nutritional and metabolic disease	NA	Lowe's syndrome	1	1	0	0	1	0	0	
E7204	E00-E89	Endocrine, nutritional and metabolic disease	NA	Cystinosis	1	1	0	0	1	0	0	
E7209	E00-E89	Endocrine, nutritional and metabolic disease	NA	Other disorders of amino-acid transport	0	1	0	0	1	0	0	
E721	E00-E89	Endocrine, nutritional and metabolic disease	NA	Disorders of sulfur-bearing amino-acid metabolism	0	1	0	0	1	0	0	
E7210	E00-E89	Endocrine, nutritional and metabolic disease	NA	Disorders of sulfur-bearing amino-acid metabolism, unspecified	0	1	0	0	1	0	0	
E7211	E00-E89	Endocrine, nutritional and metabolic disease	NA	Homocystinuria	1	1	0	0	1	0	0	
E7219	E00-E89	Endocrine, nutritional and metabolic disease	NA	Other disorders of sulfur-bearingamino-acid metabolism	0	1	0	0	1	0	0	
E722	E00-E89	Endocrine, nutritional and metabolic disease	NA	Disorders of urea cycle metabolism	1	1	0	0	1	0	0	
E7220	E00-E89	Endocrine, nutritional and metabolic disease	NA	Disorder of urea cycle metabolism,unspecified	1	1	0	0	1	0	0	
E7221	E00-E89	Endocrine, nutritional and metabolic disease	NA	Argininemia	1	1	0	0	1	0	0	
E7222	E00-E89	Endocrine, nutritional and metabolic disease	NA	Arginosuccinic aciduria	1	1	0	0	1	0	0	
E7223	E00-E89	Endocrine, nutritional and metabolic disease	NA	Citrullinemia	1	1	0	0	1	0	0	
E7229	E00-E89	Endocrine, nutritional and metabolic disease	NA	Other disorders of urea cycle metabolism	0	1	0	0	1	0	0	
E723	E00-E89	Endocrine, nutritional and metabolic disease	NA	Disorders of lysine and hydroxylysine metabolism	0	1	0	0	1	0	0	
E724	E00-E89	Endocrine, nutritional and metabolic disease	NA	Disorders of ornithine metabolism	0	1	0	0	1	0	0	
E725	E00-E89	Endocrine, nutritional and metabolic disease	NA	Disorders of glycine metabolism	0	1	0	0	1	0	0	
E7250	E00-E89	Endocrine, nutritional and metabolic disease	NA	Disorder of glycine metabolism, unspecified	0	1	0	0	1	0	0	
E7251	E00-E89	Endocrine, nutritional and metabolic disease	NA	Non-ketotic hyperglycinemia	1	1	0	0	1	0	0	
E7252	E00-E89	Endocrine, nutritional and metabolic disease	NA	Trimethylaminuria	1	1	0	0	1	0	0	
E7253	E00-E89	Endocrine, nutritional and metabolic disease	NA	Hyperoxaluria	1	1	0	0	1	0	0	
E7259	E00-E89	Endocrine, nutritional and metabolic disease	NA	Other disorders of glycine metabolism	0	1	0	0	1	0	0	
E728	E00-E89	Endocrine, nutritional and metabolic disease	NA	Other specified disorders of amino#NAME?	0	1	0	0	1	0	0	
E729	E00-E89	Endocrine, nutritional and metabolic disease	NA	Disorder of amino-acid metabolism,unspecified	0	1	0	0	1	0	0	
E73	E00-E89	Endocrine, nutritional and metabolic disease	NA	Lactose intolerance	0	0	0	1	0	0	1	
E730	E00-E89	Endocrine, nutritional and metabolic disease	NA	Congenital lactase deficiency	1	1	0	0	1	0	0	
E738	E00-E89	Endocrine, nutritional and metabolic disease	NA	Other lactose intolerance	0	0	0	1	0	0	1	
E74	E00-E89	Endocrine, nutritional and metabolic disease	NA	Other disorders of carbohydrate metabolism	0	1	0	0	1	0	0	
E740	E00-E89	Endocrine, nutritional and metabolic disease	NA	Glycogen storage disease	0	1	0	0	1	0	0	
E7400	E00-E89	Endocrine, nutritional and metabolic disease	NA	Glycogen storage disease, unspecified	0	1	0	0	1	0	0	
E7401	E00-E89	Endocrine, nutritional and metabolic disease	NA	von Gierke disease	1	1	0	0	1	0	0	
E7402	E00-E89	Endocrine, nutritional and metabolic disease	NA	Pompe disease	1	1	0	0	1	0	0	
E7403	E00-E89	Endocrine, nutritional and metabolic disease	NA	Cori disease	1	1	0	0	1	0	0	
E7404	E00-E89	Endocrine, nutritional and metabolic disease	NA	McArdle disease	1	1	0	0	1	0	0	
E7409	E00-E89	Endocrine, nutritional and metabolic disease	NA	Other glycogen storage disease	0	1	0	0	1	0	0	
E741	E00-E89	Endocrine, nutritional and metabolic disease	NA	Disorders of fructose metabolism	0	1	0	0	1	0	0	
E7410	E00-E89	Endocrine, nutritional and metabolic disease	NA	Disorder of fructose metabolism, unspecified	0	1	0	0	1	0	0	
E7411	E00-E89	Endocrine, nutritional and metabolic disease	NA	Essential fructosuria	1	1	0	0	1	0	0	
E7412	E00-E89	Endocrine, nutritional and metabolic disease	NA	Hereditary fructose intolerance	1	1	0	0	1	0	0	
E7419	E00-E89	Endocrine, nutritional and metabolic disease	NA	Other disorders of fructose metabolism	0	1	0	0	1	0	0	
E742	E00-E89	Endocrine, nutritional and metabolic disease	NA	Disorders of galactose metabolism	1	1	0	0	1	0	0	
E7420	E00-E89	Endocrine, nutritional and metabolic disease	NA	Disorders of galactose metabolism,unspecified	1	1	0	0	1	0	0	
E7421	E00-E89	Endocrine, nutritional and metabolic disease	NA	Galactosemia	1	1	0	0	1	0	0	
E7429	E00-E89	Endocrine, nutritional and metabolic disease	NA	Other disorders of galactose metabolism	0	1	0	0	1	0	0	
E743	E00-E89	Endocrine, nutritional and metabolic disease	NA	Other disorders of intestinal carbohydrate absorption	0	0	1	0	0	1	0	
E7431	E00-E89	Endocrine, nutritional and metabolic disease	NA	Sucrase-isomaltase deficiency	1	1	0	0	1	0	0	
E7439	E00-E89	Endocrine, nutritional and metabolic disease	NA	Other disorders of intestinal carbohydrate absorption	0	0	1	0	0	1	0	
E744	E00-E89	Endocrine, nutritional and metabolic disease	NA	Disorders of pyruvate metabolism and gluconeogenesis	0	1	0	0	1	0	0	
E748	E00-E89	Endocrine, nutritional and metabolic disease	NA	Other specified disorders of carbohydrate metabolism	0	1	0	0	1	0	0	
E749	E00-E89	Endocrine, nutritional and metabolic disease	NA	Disorder of carbohydrate metabolism, unspecified	0	1	0	0	1	0	0	
E75	E00-E89	Endocrine, nutritional and metabolic disease	NA	Disorders of sphingolipid metabolism and other lipid storage disorders	0	1	0	0	1	0	0	
E750	E00-E89	Endocrine, nutritional and metabolic disease	NA	GM2 gangliosidosis	1	1	0	0	1	0	0	
E7500	E00-E89	Endocrine, nutritional and metabolic disease	NA	GM2 gangliosidosis, unspecified	1	1	0	0	1	0	0	
E7501	E00-E89	Endocrine, nutritional and metabolic disease	NA	Sandhoff disease	1	1	0	0	1	0	0	
E7502	E00-E89	Endocrine, nutritional and metabolic disease	NA	Tay-Sachs disease	1	1	0	0	1	0	0	
E7509	E00-E89	Endocrine, nutritional and metabolic disease	NA	Other GM2 gangliosidosis	0	1	0	0	1	0	0	
E751	E00-E89	Endocrine, nutritional and metabolic disease	NA	Other and unspecified gangliosidosis	0	1	0	0	1	0	0	
E7510	E00-E89	Endocrine, nutritional and metabolic disease	NA	Unspecified gangliosidosis	0	1	0	0	1	0	0	
E7511	E00-E89	Endocrine, nutritional and metabolic disease	NA	Mucolipidosis IV	1	1	0	0	1	0	0	
E7519	E00-E89	Endocrine, nutritional and metabolic disease	NA	Other gangliosidosis	0	1	0	0	1	0	0	
E752	E00-E89	Endocrine, nutritional and metabolic disease	NA	Other sphingolipidosis	0	1	0	0	1	0	0	
E7521	E00-E89	Endocrine, nutritional and metabolic disease	NA	Fabry (-Anderson) disease	1	1	0	0	1	0	0	
E7522	E00-E89	Endocrine, nutritional and metabolic disease	NA	Gaucher disease	1	1	0	0	1	0	0	
E7523	E00-E89	Endocrine, nutritional and metabolic disease	NA	Krabbe disease	1	1	0	0	1	0	0	
E7524	E00-E89	Endocrine, nutritional and metabolic disease	NA	Niemann-Pick disease	1	1	0	0	1	0	0	
E75240	E00-E89	Endocrine, nutritional and metabolic disease	NA	Niemann-Pick disease type A	1	1	0	0	1	0	0	
E75241	E00-E89	Endocrine, nutritional and metabolic disease	NA	Niemann-Pick disease type B	1	1	0	0	1	0	0	
E75242	E00-E89	Endocrine, nutritional and metabolic disease	NA	Niemann-Pick disease type C	1	1	0	0	1	0	0	
E75243	E00-E89	Endocrine, nutritional and metabolic disease	NA	Niemann-Pick disease type D	1	1	0	0	1	0	0	
E75248	E00-E89	Endocrine, nutritional and metabolic disease	NA	Other Niemann-Pick disease	1	1	0	0	1	0	0	
E75249	E00-E89	Endocrine, nutritional and metabolic disease	NA	Niemann-Pick disease, unspecified	1	1	0	0	1	0	0	
E7525	E00-E89	Endocrine, nutritional and metabolic disease	NA	Metachromatic leukodystrophy	1	1	0	0	1	0	0	
E7529	E00-E89	Endocrine, nutritional and metabolic disease	NA	Other sphingolipidosis	0	1	0	0	1	0	0	
E753	E00-E89	Endocrine, nutritional and metabolic disease	NA	Sphingolipidosis, unspecified	0	1	0	0	1	0	0	
E754	E00-E89	Endocrine, nutritional and metabolic disease	NA	Neuronal ceroid lipofuscinosis	1	1	0	0	1	0	0	
E755	E00-E89	Endocrine, nutritional and metabolic disease	NA	Other lipid storage disorders	0	1	0	0	1	0	0	
E756	E00-E89	Endocrine, nutritional and metabolic disease	NA	Lipid storage disorder, unspecified	0	1	0	0	1	0	0	
E76	E00-E89	Endocrine, nutritional and metabolic disease	NA	Disorders of glycosaminoglycan metabolism	0	1	0	0	1	0	0	
E760	E00-E89	Endocrine, nutritional and metabolic disease	NA	Mucopolysaccharidosis, type I	1	1	0	0	1	0	0	
E7601	E00-E89	Endocrine, nutritional and metabolic disease	NA	Hurler's syndrome	1	1	0	0	1	0	0	
E7602	E00-E89	Endocrine, nutritional and metabolic disease	NA	Hurler-Scheie syndrome	1	1	0	0	1	0	0	
E7603	E00-E89	Endocrine, nutritional and metabolic disease	NA	Scheie's syndrome	1	1	0	0	1	0	0	
E761	E00-E89	Endocrine, nutritional and metabolic disease	NA	Mucopolysaccharidosis, type II	1	1	0	0	1	0	0	
E762	E00-E89	Endocrine, nutritional and metabolic disease	NA	Other mucopolysaccharidoses	1	1	0	0	1	0	0	
E7621	E00-E89	Endocrine, nutritional and metabolic disease	NA	Morquio mucopolysaccharidoses	1	1	0	0	1	0	0	
E76210	E00-E89	Endocrine, nutritional and metabolic disease	NA	Morquio A mucopolysaccharidoses	1	1	0	0	1	0	0	
E76211	E00-E89	Endocrine, nutritional and metabolic disease	NA	Morquio B mucopolysaccharidoses	1	1	0	0	1	0	0	
E76219	E00-E89	Endocrine, nutritional and metabolic disease	NA	Morquio mucopolysaccharidoses, unspecified	1	1	0	0	1	0	0	
E7622	E00-E89	Endocrine, nutritional and metabolic disease	NA	Sanfilippo mucopolysaccharidoses	1	1	0	0	1	0	0	
E7629	E00-E89	Endocrine, nutritional and metabolic disease	NA	Other mucopolysaccharidoses	1	1	0	0	1	0	0	
E763	E00-E89	Endocrine, nutritional and metabolic disease	NA	Mucopolysaccharidosis, unspecified	1	1	0	0	1	0	0	
E768	E00-E89	Endocrine, nutritional and metabolic disease	NA	Other disorders of glucosaminoglycan metabolism	0	1	0	0	1	0	0	
E769	E00-E89	Endocrine, nutritional and metabolic disease	NA	Glucosaminoglycan metabolism disorder, unspecified	0	1	0	0	1	0	0	
E77	E00-E89	Endocrine, nutritional and metabolic disease	NA	Disorders of glycoprotein metabolism	0	1	0	0	1	0	0	
E770	E00-E89	Endocrine, nutritional and metabolic disease	NA	Defects in post-translational modification of lysosomal enzymes	0	1	0	0	1	0	0	
E771	E00-E89	Endocrine, nutritional and metabolic disease	NA	Defects in glycoprotein degradation	0	1	0	0	1	0	0	
E778	E00-E89	Endocrine, nutritional and metabolic disease	NA	Other disorders of glycoprotein metabolism	0	1	0	0	1	0	0	
E779	E00-E89	Endocrine, nutritional and metabolic disease	NA	Disorder of glycoprotein metabolism, unspecified	0	1	0	0	1	0	0	
E78	E00-E89	Endocrine, nutritional and metabolic disease	NA	Disorders of lipoprotein metabolism and other lipidemias	0	1	0	0	1	0	0	
E780	E00-E89	Endocrine, nutritional and metabolic disease	NA	Pure hypercholesterolemia	0	0	1	0	0	0	1	
E7800	E00-E89	Endocrine, nutritional and metabolic disease	NA	Pure hypercholesterolemia, unspecified	0	0	0	1	0	0	1	
E7801	E00-E89	Endocrine, nutritional and metabolic disease	NA	Familial hypercholesterolemia	1	1	0	0	1	0	0	
E781	E00-E89	Endocrine, nutritional and metabolic disease	NA	Pure hyperglyceridemia	0	0	0	1	0	0	1	
E783	E00-E89	Endocrine, nutritional and metabolic disease	NA	Hyperchylomicronemia	0	0	0	1	0	0	1	
E786	E00-E89	Endocrine, nutritional and metabolic disease	NA	Lipoprotein deficiency	0	1	0	0	1	0	0	
E787	E00-E89	Endocrine, nutritional and metabolic disease	NA	Disorders of bile acid and cholesterol metabolism	0	1	0	0	1	0	0	
E7870	E00-E89	Endocrine, nutritional and metabolic disease	NA	Disorder of bile acid and cholesterol metabolism, unspecified	0	1	0	0	1	0	0	
E7871	E00-E89	Endocrine, nutritional and metabolic disease	NA	Barth syndrome	0	1	0	0	1	0	0	
E7872	E00-E89	Endocrine, nutritional and metabolic disease	NA	Smith-Lemli-Opitz syndrome	1	1	0	0	1	0	0	
E7879	E00-E89	Endocrine, nutritional and metabolic disease	NA	Other disorders of bile acid and cholesterol metabolism	0	1	0	0	1	0	0	
E788	E00-E89	Endocrine, nutritional and metabolic disease	NA	Other disorders of lipoprotein metabolism	0	1	0	0	1	0	0	
E7881	E00-E89	Endocrine, nutritional and metabolic disease	NA	Lipoid dermatoarthritis	0	0	0	0	0	0	1	
E7889	E00-E89	Endocrine, nutritional and metabolic disease	NA	Other lipoprotein metabolism disorders	0	0	0	1	0	0	1	
E789	E00-E89	Endocrine, nutritional and metabolic disease	NA	Disorder of lipoprotein metabolism, unspecified	0	0	0	1	0	0	1	
E79	E00-E89	Endocrine, nutritional and metabolic disease	NA	Disorders of purine and pyrimidinemetabolism	0	1	0	0	1	0	0	
E790	E00-E89	Endocrine, nutritional and metabolic disease	NA	Hyperuricemia without signs of inflammatory arthritis and tophaceous disease	0	0	0	1	0	0	1	
E791	E00-E89	Endocrine, nutritional and metabolic disease	NA	Lesch-Nyhan syndrome	1	1	0	0	1	0	0	
E792	E00-E89	Endocrine, nutritional and metabolic disease	NA	Myoadenylate deaminase deficiency	1	1	0	0	1	0	0	
E798	E00-E89	Endocrine, nutritional and metabolic disease	NA	Other disorders of purine and pyrimidine metabolism	0	1	0	0	1	0	0	
E799	E00-E89	Endocrine, nutritional and metabolic disease	NA	Disorder of purine and pyrimidinemetabolism, unspecified	0	1	0	0	1	0	0	
E80	E00-E89	Endocrine, nutritional and metabolic disease	NA	Disorders of porphyrin and bilirubin metabolism	0	1	0	0	1	0	0	
E800	E00-E89	Endocrine, nutritional and metabolic disease	NA	Hereditary erythropoietic porphyria	1	1	0	0	1	0	0	
E801	E00-E89	Endocrine, nutritional and metabolic disease	NA	Porphyria cutanea tarda	1	1	0	0	1	0	0	
E802	E00-E89	Endocrine, nutritional and metabolic disease	NA	Other and unspecified porphyria	1	1	0	0	1	0	0	
E8020	E00-E89	Endocrine, nutritional and metabolic disease	NA	Unspecified porphyria	0	1	0	0	1	0	0	
E8021	E00-E89	Endocrine, nutritional and metabolic disease	NA	Acute intermittent (hepatic) porphyria	0	1	0	0	1	0	0	
E8029	E00-E89	Endocrine, nutritional and metabolic disease	NA	Other porphyria	0	1	0	0	1	0	0	
E803	E00-E89	Endocrine, nutritional and metabolic disease	NA	Defects of catalase and peroxidase	0	1	0	0	1	0	0	
E804	E00-E89	Endocrine, nutritional and metabolic disease	NA	Gilbert syndrome	1	1	0	0	1	0	0	
E805	E00-E89	Endocrine, nutritional and metabolic disease	NA	Crigler-Najjar syndrome	1	1	0	0	1	0	0	
E806	E00-E89	Endocrine, nutritional and metabolic disease	NA	Other disorders of bilirubin metabolism	0	0	1	0	0	1	0	
E807	E00-E89	Endocrine, nutritional and metabolic disease	NA	Disorder of bilirubin metabolism,unspecified	0	0	1	0	0	1	0	
E83	E00-E89	Endocrine, nutritional and metabolic disease	NA	Disorders of mineral metabolism	0	1	0	0	1	0	0	
E830	E00-E89	Endocrine, nutritional and metabolic disease	NA	Disorders of copper metabolism	0	1	0	0	1	0	0	
E8300	E00-E89	Endocrine, nutritional and metabolic disease	NA	Disorder of copper metabolism, unspecified	0	1	0	0	1	0	0	
E8301	E00-E89	Endocrine, nutritional and metabolic disease	NA	Wilson's disease	1	1	0	0	1	0	0	
E8309	E00-E89	Endocrine, nutritional and metabolic disease	NA	Other disorders of copper metabolism	0	1	0	0	1	0	0	
E831	E00-E89	Endocrine, nutritional and metabolic disease	NA	Disorders of iron metabolism	0	1	0	0	1	0	0	
E8310	E00-E89	Endocrine, nutritional and metabolic disease	NA	Disorder of iron metabolism, unspecified	0	1	0	0	1	0	0	
E8311	E00-E89	Endocrine, nutritional and metabolic disease	NA	Hemochromatosis	0	0	1	0	0	1	0	
E83110	E00-E89	Endocrine, nutritional and metabolic disease	NA	Hereditary hemochromatosis	0	1	0	0	1	0	0	
E83118	E00-E89	Endocrine, nutritional and metabolic disease	NA	Other hemochromatosis	0	0	0	1	0	0	1	
E83119	E00-E89	Endocrine, nutritional and metabolic disease	NA	Hemochromatosis, unspecified	0	0	0	1	0	0	1	
E8319	E00-E89	Endocrine, nutritional and metabolic disease	NA	Other disorders of iron metabolism	0	0	1	0	0	1	0	
E832	E00-E89	Endocrine, nutritional and metabolic disease	NA	Disorders of zinc metabolism	0	1	0	0	1	0	0	
E833	E00-E89	Endocrine, nutritional and metabolic disease	NA	Disorders of phosphorus metabolismand phosphatases	0	1	0	0	1	0	0	
E8330	E00-E89	Endocrine, nutritional and metabolic disease	NA	Disorder of phosphorus metabolism,unspecified	0	1	0	0	1	0	0	
E8331	E00-E89	Endocrine, nutritional and metabolic disease	NA	Familial hypophosphatemia	0	1	0	0	1	0	0	
E8332	E00-E89	Endocrine, nutritional and metabolic disease	NA	Hereditary vitamin D-dependent rickets (type 1) (type 2)	1	1	0	0	1	0	0	
E8339	E00-E89	Endocrine, nutritional and metabolic disease	NA	Other disorders of phosphorus metabolism	0	0	1	0	0	1	0	
E8340	E00-E89	Endocrine, nutritional and metabolic disease	NA	Disorders of magnesium metabolism, unspecified	0	1	0	0	1	0	0	
E84	E00-E89	Endocrine, nutritional and metabolic disease	NA	Cystic fibrosis	1	1	0	0	1	0	0	
E840	E00-E89	Endocrine, nutritional and metabolic disease	NA	Cystic fibrosis with pulmonary manifestations	1	1	0	0	1	0	0	
E841	E00-E89	Endocrine, nutritional and metabolic disease	NA	Cystic fibrosis with intestinal manifestations	1	1	0	0	1	0	0	
E8411	E00-E89	Endocrine, nutritional and metabolic disease	NA	Meconium ileus in cystic fibrosis	1	1	0	0	1	0	0	
E8419	E00-E89	Endocrine, nutritional and metabolic disease	NA	Cystic fibrosis with other intestinal manifestations	1	1	0	0	1	0	0	
E848	E00-E89	Endocrine, nutritional and metabolic disease	NA	Cystic fibrosis with other manifestations	1	1	0	0	1	0	0	
E849	E00-E89	Endocrine, nutritional and metabolic disease	NA	Cystic fibrosis, unspecified	1	1	0	0	1	0	0	
E85	E00-E89	Endocrine, nutritional and metabolic disease	NA	Amyloidosis	0	0	0	0	0	1	0	
E850	E00-E89	Endocrine, nutritional and metabolic disease	NA	Non-neuropathic heredofamilial amyloidosis	0	1	0	0	1	0	0	
E851	E00-E89	Endocrine, nutritional and metabolic disease	NA	Neuropathic heredofamilial amyloidosis	0	1	0	0	1	0	0	
E852	E00-E89	Endocrine, nutritional and metabolic disease	NA	Heredofamilial amyloidosis, unspecified	0	1	0	0	1	0	0	
E854	E00-E89	Endocrine, nutritional and metabolic disease	NA	Organ-limited amyloidosis	0	0	0	0	0	1	0	
E858	E00-E89	Endocrine, nutritional and metabolic disease	NA	Other amyloidosis	0	0	0	0	0	0	1	
E8589	E00-E89	Endocrine, nutritional and metabolic disease	NA	Other amyloidosis	0	0	0	0	0	0	1	
E859	E00-E89	Endocrine, nutritional and metabolic disease	NA	Amyloidosis, unspecified	0	0	0	0	0	0	1	
E8801	E00-E89	Endocrine, nutritional and metabolic disease	NA	Alpha-1-antitrypsin deficiency	1	1	0	0	1	0	0	
E881	E00-E89	Endocrine, nutritional and metabolic disease	NA	Lipodystrophy, not elsewhere classified	0	1	0	0	1	0	0	
E882	E00-E89	Endocrine, nutritional and metabolic disease	NA	LIPOMATOSIS NEC	0	0	0	0	0	0	1	
E884	E00-E89	Endocrine, nutritional and metabolic disease	NA	Mitochondrial metabolism disorders	0	1	0	0	1	0	0	
E8840	E00-E89	Endocrine, nutritional and metabolic disease	NA	Mitochondrial metabolism disorder,unspecified	0	1	0	0	1	0	0	
E8841	E00-E89	Endocrine, nutritional and metabolic disease	NA	MELAS syndrome	1	1	0	0	1	0	0	
E8842	E00-E89	Endocrine, nutritional and metabolic disease	NA	MERRF syndrome	1	1	0	0	1	0	0	
E8849	E00-E89	Endocrine, nutritional and metabolic disease	NA	Other mitochondrial metabolism disorders	0	1	0	0	1	0	0	
E888	E00-E89	Endocrine, nutritional and metabolic disease	NA	Other specified metabolic disorders	0	0	1	0	0	1	0	
E8889	E00-E89	Endocrine, nutritional and metabolic disease	NA	Other specified metabolic disorders	0	0	1	0	0	1	0	
E889	E00-E89	Endocrine, nutritional and metabolic disease	NA	Metabolic disorder, unspecified	0	0	1	0	0	1	0	
F200	F01-F99	Mental, behavioral, and neurodevelopment	NA	Paranoid schizophrenia	0	0	0	0	0	0	1	
F201	F01-F99	Mental, behavioral, and neurodevelopment	NA	Disorganized schizophrenia	0	0	0	0	0	0	1	
F202	F01-F99	Mental, behavioral, and neurodevelopment	NA	Catatonic schizophrenia	0	0	0	0	0	0	1	
F203	F01-F99	Mental, behavioral, and neurodevelopment	NA	Undifferentiated schizophrenia	0	0	0	0	0	0	1	
F205	F01-F99	Mental, behavioral, and neurodevelopment	NA	Residual schizophrenia	0	0	0	0	0	0	1	
F2081	F01-F99	Mental, behavioral, and neurodevelopment	NA	Schizophreniform disorder	0	0	0	0	0	0	1	
F2089	F01-F99	Mental, behavioral, and neurodevelopment	NA	Other schizophrenia	0	0	0	0	0	0	1	
F209	F01-F99	Mental, behavioral, and neurodevelopment	NA	Schizophrenia, unspecified	0	0	0	0	0	0	1	
F21	F01-F99	Mental, behavioral, and neurodevelopment	NA	Schizotypal disorder	0	0	0	0	0	0	1	
F22	F01-F99	Mental, behavioral, and neurodevelopment	NA	Delusional disorders	0	0	0	0	0	0	1	
F23	F01-F99	Mental, behavioral, and neurodevelopment	NA	Brief psychotic disorder	0	0	0	0	0	0	1	
F24	F01-F99	Mental, behavioral, and neurodevelopment	NA	Shared psychotic disorder	0	0	0	0	0	0	1	
F250	F01-F99	Mental, behavioral, and neurodevelopment	NA	Schizoaffective disorder, bipolar type	0	0	0	0	0	0	1	
F251	F01-F99	Mental, behavioral, and neurodevelopment	NA	Schizoaffective disorder, depressive type	0	0	0	0	0	0	1	
F258	F01-F99	Mental, behavioral, and neurodevelopment	NA	Other schizoaffective disorders	0	0	0	0	0	0	1	
F259	F01-F99	Mental, behavioral, and neurodevelopment	NA	Schizoaffective disorder, unspecified	0	0	0	0	0	0	1	
F28	F01-F99	Mental, behavioral, and neurodevelopment	NA	Other psychotic disorder not due to a substance or known physiological condition	0	0	0	0	0	0	1	
F29	F01-F99	Mental, behavioral, and neurodevelopment	NA	Unspecified psychosis not due to a substance or known physiological condition	0	0	0	0	0	0	1	
F70	F01-F99	Mental, behavioral, and neurodevelopment	NA	Mild intellectual disabilities	0	0	0	0	0	0	1	
F71	F01-F99	Mental, behavioral, and neurodevelopment	NA	Moderate intellectual disabilities	0	0	0	0	0	0	1	1
F72	F01-F99	Mental, behavioral, and neurodevelopment	NA	Severe intellectual disabilities	0	0	0	0	0	1	0	1
F73	F01-F99	Mental, behavioral, and neurodevelopment	NA	Profound intellectual disabilities	0	0	0	0	0	1	0	1
F78	F01-F99	Mental, behavioral, and neurodevelopment	NA	Other intellectual disabilities	0	0	0	0	0	0	1	1
F79	F01-F99	Mental, behavioral, and neurodevelopment	NA	Unspecified intellectual disabilities	0	0	0	0	0	0	1	1
F80	F01-F99	Mental, behavioral, and neurodevelopment	NA	Specific developmental disorders of speech and language	0	0	0	0	0	0	1	
F804	F01-F99	Mental, behavioral, and neurodevelopment	NA	Speech and language development delay due to hearing loss	0	0	0	0	0	0	1	
F808	F01-F99	Mental, behavioral, and neurodevelopment	NA	Other developmental disorders of speech and language	0	0	0	0	0	0	1	
F84	F01-F99	Mental, behavioral, and neurodevelopment	NA	Pervasive developmental disorders	0	0	0	0	0	0	1	
F840	F01-F99	Mental, behavioral, and neurodevelopment	NA	Autistic disorder	0	0	0	0	0	0	1	
F842	F01-F99	Mental, behavioral, and neurodevelopment	NA	Rett's syndrome	1	1	0	0	1	0	0	
F843	F01-F99	Mental, behavioral, and neurodevelopment	NA	Other childhood disintegrative disorder	0	0	0	0	0	0	1	
F848	F01-F99	Mental, behavioral, and neurodevelopment	NA	Other pervasive developmental disorders	0	0	0	0	0	0	1	
F849	F01-F99	Mental, behavioral, and neurodevelopment	NA	Pervasive developmental disorder,unspecified	0	0	0	0	0	0	1	
F984	F01-F99	Mental, behavioral, and neurodevelopment	NA	Stereotyped movement disorders	0	0	0	0	0	0	1	
G10	G00-G99	Diseases of the nervous system	NA	Huntington's disease	1	1	0	0	1	0	0	
G11	G00-G99	Diseases of the nervous system	NA	Hereditary ataxia	0	1	0	0	1	0	0	

G110	G00-G99	Diseases of the nervous system	NA	Congenital nonprogressive ataxia	0	0	0	1	0	0	1	
G111	G00-G99	Diseases of the nervous system	NA	Early-onset cerebellar ataxia	0	0	0	0	0	1	0	
G112	G00-G99	Diseases of the nervous system	NA	Late-onset cerebellar ataxia	0	0	0	0	0	1	0	
G113	G00-G99	Diseases of the nervous system	NA	Cerebellar ataxia with defective DNA repair	0	1	0	0	1	0	0	
G114	G00-G99	Diseases of the nervous system	NA	Hereditary spastic paraplegia	0	1	0	0	1	0	0	
G118	G00-G99	Diseases of the nervous system	NA	Other hereditary ataxias	0	1	0	0	1	0	0	
G119	G00-G99	Diseases of the nervous system	NA	Hereditary ataxia, unspecified	0	1	0	0	1	0	0	
G12	G00-G99	Diseases of the nervous system	NA	Spinal muscular atrophy and related syndromes	1	1	0	0	1	0	0	
G120	G00-G99	Diseases of the nervous system	NA	Infantile spinal muscular atrophy,type I [Werdnig-Hoffman]	1	1	0	0	1	0	0	
G121	G00-G99	Diseases of the nervous system	NA	Other inherited spinal muscular atrophy	0	1	0	0	1	0	0	
G122	G00-G99	Diseases of the nervous system	NA	Motor neuron disease	0	0	1	0	0	1	0	
G1220	G00-G99	Diseases of the nervous system	NA	Motor neuron disease, unspecified	0	0	1	0	0	1	0	
G1221	G00-G99	Diseases of the nervous system	NA	Amyotrophic lateral sclerosis	0	0	0	0	0	0	1	
G1222	G00-G99	Diseases of the nervous system	NA	Progressive bulbar palsy	0	0	0	1	0	0	1	
G1223	G00-G99	Diseases of the nervous system	NA	Primary lateral sclerosis	0	0	0	0	0	0	1	
G1224	G00-G99	Diseases of the nervous system	NA	Familial motor neuron disease	0	1	0	0	1	0	0	
G1225	G00-G99	Diseases of the nervous system	NA	Progressive spinal muscle atrophy	0	1	0	0	1	0	0	
G1229	G00-G99	Diseases of the nervous system	NA	Other motor neuron disease	0	0	1	0	0	1	0	
G128	G00-G99	Diseases of the nervous system	NA	Other spinal muscular atrophies and related syndromes	0	0	1	0	0	1	0	
G129	G00-G99	Diseases of the nervous system	NA	Spinal muscular atrophy, unspecified	1	1	0	0	1	0	0	
G13	G00-G99	Diseases of the nervous system	NA	Systemic atrophies primarily affecting central nervous system in diseases classified els	0	0	0	0	0	0	1	
G23	G00-G99	Diseases of the nervous system	NA	Other degenerative diseases of basal ganglia	0	0	1	0	0	1	0	
G230	G00-G99	Diseases of the nervous system	NA	Hallervorden-Spatz disease	1	1	0	0	1	0	0	
G231	G00-G99	Diseases of the nervous system	NA	Progressive supranuclear ophthalmoplegia [Steele-Richardson-Olszewski]	0	0	1	0	0	1	0	
G232	G00-G99	Diseases of the nervous system	NA	Striatonigral degeneration	0	0	1	0	0	1	0	
G238	G00-G99	Diseases of the nervous system	NA	Other specified degenerative diseases of basal ganglia	0	0	1	0	0	1	0	
G239	G00-G99	Diseases of the nervous system	NA	Degenerative disease of basal ganglia, unspecified	0	0	1	0	0	1	0	
G24	G00-G99	Diseases of the nervous system	NA	Dystonia	0	0	0	1	0	0	1	
G241	G00-G99	Diseases of the nervous system	NA	Genetic torsion dystonia	0	1	0	0	1	0	0	
G242	G00-G99	Diseases of the nervous system	NA	Idiopathic nonfamilial dystonia	0	0	1	0	0	1	0	
G243	G00-G99	Diseases of the nervous system	NA	Spasmodic torticollis	0	0	0	1	0	0	1	
G250	G00-G99	Diseases of the nervous system	NA	Essential tremor	0	0	0	0	0	0	1	
G2582	G00-G99	Diseases of the nervous system	NA	Stiff-man syndrome	0	0	1	0	0	1	0	
G300	G00-G99	Diseases of the nervous system	NA	Alzheimer's disease with early onset	0	0	0	0	0	1	0	
G310	G00-G99	Diseases of the nervous system	NA	Frontotemporal dementia	0	0	0	0	0	1	0	
G3101	G00-G99	Diseases of the nervous system	NA	Pick's disease	1	1	0	0	1	0	0	
G3109	G00-G99	Diseases of the nervous system	NA	Other frontotemporal dementia	0	0	0	0	0	1	0	
G3181	G00-G99	Diseases of the nervous system	NA	Alpers disease	1	1	0	0	1	0	0	
G3182	G00-G99	Diseases of the nervous system	NA	Leigh's disease	1	1	0	0	1	0	0	
G3183	G00-G99	Diseases of the nervous system	NA	Dementia with Lewy bodies	0	0	0	0	0	1	0	
G3185	G00-G99	Diseases of the nervous system	NA	Corticobasal degeneration	0	0	1	0	0	1	0	
G3189	G00-G99	Diseases of the nervous system	NA	Other specified degenerative diseases of nervous system	0	0	1	0	0	1	0	
G319	G00-G99	Diseases of the nervous system	NA	Degenerative disease of nervous system, unspecified	0	0	1	0	0	1	0	
G32	G00-G99	Diseases of the nervous system	NA	Other degenerative disorders of nervous system in diseases classified elsewhere	0	0	1	0	0	1	0	
G3281	G00-G99	Diseases of the nervous system	NA	Cerebellar ataxia in diseases classified elsewhere	0	0	0	0	0	0	1	
G3289	G00-G99	Diseases of the nervous system	NA	Other specified degenerative disorders of nervous system in diseases classified elsew	0	0	1	0	0	1	0	
G370	G00-G99	Diseases of the nervous system	NA	Diffuse sclerosis of central nervous system	0	0	0	0	0	0	1	
G371	G00-G99	Diseases of the nervous system	NA	Central demyelination of corpus callosum	0	0	0	1	0	0	1	
G40	G00-G99	Diseases of the nervous system	NA	Epilepsy and recurrent seizures	0	0	0	1	0	0	1	1
G400	G00-G99	Diseases of the nervous system	NA	Localization-related (focal) (partial) idiopathic epilepsy and epileptic syndromes with s	0	0	0	1	0	0	1	1
G4000	G00-G99	Diseases of the nervous system	NA	Localization-related (focal) (partial) idiopathic epilepsy and epileptic syndromes with s	0	0	0	1	0	0	1	1
G40001	G00-G99	Diseases of the nervous system	NA	Localization-related (focal) (partial) idiopathic epilepsy and epileptic syndromes with s	0	0	0	1	0	0	1	1
G40009	G00-G99	Diseases of the nervous system	NA	Localization-related (focal) (partial) idiopathic epilepsy and epileptic syndromes with s	0	0	0	1	0	0	1	1
G4001	G00-G99	Diseases of the nervous system	NA	Localization-related (focal) (partial) idiopathic epilepsy and epileptic syndromes with s	0	0	0	1	0	0	1	1
G40011	G00-G99	Diseases of the nervous system	NA	Localization-related (focal) (partial) idiopathic epilepsy and epileptic syndromes with s	0	0	0	1	0	0	1	1
G40019	G00-G99	Diseases of the nervous system	NA	Localization-related (focal) (partial) idiopathic epilepsy and epileptic syndromes with s	0	0	0	1	0	0	1	1
G401	G00-G99	Diseases of the nervous system	NA	Localization-related (focal) (partial) symptomatic epilepsy and epileptic syndromes wit	0	0	0	1	0	0	1	1
G4010	G00-G99	Diseases of the nervous system	NA	Localization-related (focal) (partial) symptomatic epilepsy and epileptic syndromes wit	0	0	0	1	0	0	1	1
G40101	G00-G99	Diseases of the nervous system	NA	Localization-related (focal) (partial) symptomatic epilepsy and epileptic syndromes wit	0	0	0	1	0	0	1	1
G40109	G00-G99	Diseases of the nervous system	NA	Localization-related (focal) (partial) symptomatic epilepsy and epileptic syndromes wit	0	0	0	1	0	0	1	1
G4011	G00-G99	Diseases of the nervous system	NA	Localization-related (focal) (partial) symptomatic epilepsy and epileptic syndromes wit	0	0	0	1	0	0	1	1
G40111	G00-G99	Diseases of the nervous system	NA	Localization-related (focal) (partial) symptomatic epilepsy and epileptic syndromes wit	0	0	0	1	0	0	1	1
G40119	G00-G99	Diseases of the nervous system	NA	Localization-related (focal) (partial) symptomatic epilepsy and epileptic syndromes wit	0	0	0	1	0	0	1	1
G402	G00-G99	Diseases of the nervous system	NA	Localization-related (focal) (partial) symptomatic epilepsy and epileptic syndromes wit	0	0	0	1	0	0	1	1
G4020	G00-G99	Diseases of the nervous system	NA	Localization-related (focal) (partial) symptomatic epilepsy and epileptic syndromes wit	0	0	0	1	0	0	1	1
G40201	G00-G99	Diseases of the nervous system	NA	Localization-related (focal) (partial) symptomatic epilepsy and epileptic syndromes wit	0	0	0	1	0	0	1	1
G40209	G00-G99	Diseases of the nervous system	NA	Localization-related (focal) (partial) symptomatic epilepsy and epileptic syndromes wit	0	0	0	1	0	0	1	1
G4021	G00-G99	Diseases of the nervous system	NA	Localization-related (focal) (partial) symptomatic epilepsy and epileptic syndromes wit	0	0	0	1	0	0	1	1
G40211	G00-G99	Diseases of the nervous system	NA	Localization-related (focal) (partial) symptomatic epilepsy and epileptic syndromes wit	0	0	0	1	0	0	1	1
G40219	G00-G99	Diseases of the nervous system	NA	Localization-related (focal) (partial) symptomatic epilepsy and epileptic syndromes wit	0	0	0	1	0	0	1	1
G403	G00-G99	Diseases of the nervous system	NA	Generalized idiopathic epilepsy and epileptic syndromes	0	0	0	1	0	0	1	1
G4030	G00-G99	Diseases of the nervous system	NA	Generalized idiopathic epilepsy and epileptic syndromes, not intractable	0	0	0	1	0	0	1	1
G40301	G00-G99	Diseases of the nervous system	NA	Generalized idiopathic epilepsy and epileptic syndromes, not intractable, with status ep	0	0	0	1	0	0	1	1
G40309	G00-G99	Diseases of the nervous system	NA	Generalized idiopathic epilepsy and epileptic syndromes, not intractable, without statu	0	0	0	1	0	0	1	1
G4031	G00-G99	Diseases of the nervous system	NA	Generalized idiopathic epilepsy and epileptic syndromes, intractable	0	0	1	0	0	0	1	1
G40311	G00-G99	Diseases of the nervous system	NA	Generalized idiopathic epilepsy and epileptic syndromes, intractable, with status epilep	0	0	1	0	0	0	1	1
G40319	G00-G99	Diseases of the nervous system	NA	Generalized idiopathic epilepsy and epileptic syndromes, intractable, without status ep	0	0	1	0	0	0	1	1
G404	G00-G99	Diseases of the nervous system	NA	Other generalized epilepsy and epileptic syndromes	0	0	0	1	0	0	1	1
G4040	G00-G99	Diseases of the nervous system	NA	Other generalized epilepsy and epileptic syndromes, not intractable	0	0	0	1	0	0	1	1
G40401	G00-G99	Diseases of the nervous system	NA	Other generalized epilepsy and epileptic syndromes, not intractable, with status epilep	0	0	0	1	0	0	1	1
G40409	G00-G99	Diseases of the nervous system	NA	Other generalized epilepsy and epileptic syndromes, not intractable, without status ep	0	0	0	1	0	0	1	1
G4041	G00-G99	Diseases of the nervous system	NA	Other generalized epilepsy and epileptic syndromes, intractable	0	0	0	1	0	0	1	1
G40411	G00-G99	Diseases of the nervous system	NA	Other generalized epilepsy and epileptic syndromes, intractable, with status epilepticu	0	0	0	1	0	0	1	1
G40419	G00-G99	Diseases of the nervous system	NA	Other generalized epilepsy and epileptic syndromes, intractable, without status epilep	0	0	0	1	0	0	1	1
G408	G00-G99	Diseases of the nervous system	NA	Other epilepsy and recurrent seizures	0	0	0	1	0	0	1	1
G4080	G00-G99	Diseases of the nervous system	NA	Other epilepsy	0	0	0	1	0	0	1	1
G40801	G00-G99	Diseases of the nervous system	NA	Other epilepsy, not intractable, with status epilepticus	0	0	0	1	0	0	1	1
G40802	G00-G99	Diseases of the nervous system	NA	Other epilepsy, not intractable, without status epilepticus	0	0	0	1	0	0	1	1
G40803	G00-G99	Diseases of the nervous system	NA	Other epilepsy, intractable, withstatus epilepticus	0	0	0	1	0	0	1	1
G40804	G00-G99	Diseases of the nervous system	NA	Other epilepsy, intractable, without status epilepticus	0	0	0	1	0	0	1	1
G4082	G00-G99	Diseases of the nervous system	NA	Epileptic spasms	0	0	1	0	0	1	0	1
G40821	G00-G99	Diseases of the nervous system	NA	Epileptic spasms, not intractable,with status epilepticus	0	0	0	1	0	0	1	1
G40822	G00-G99	Diseases of the nervous system	NA	Epileptic spasms, not intractable,without status epilepticus	0	0	0	1	0	0	1	1
G40823	G00-G99	Diseases of the nervous system	NA	Epileptic spasms, intractable, with status epilepticus	0	0	1	0	0	1	0	1
G40824	G00-G99	Diseases of the nervous system	NA	Epileptic spasms, intractable, without status epilepticus	0	0	1	0	0	1	0	1
G409	G00-G99	Diseases of the nervous system	NA	Epilepsy, unspecified	0	0	0	1	0	0	1	1
G4090	G00-G99	Diseases of the nervous system	NA	Epilepsy, unspecified, not intractable	0	0	0	1	0	0	1	1
G40901	G00-G99	Diseases of the nervous system	NA	Epilepsy, unspecified, not intractable, with status epilepticus	0	0	0	1	0	0	1	1
G4091	G00-G99	Diseases of the nervous system	NA	Epilepsy, unspecified, intractable	0	0	1	0	0	0	1	1
G40911	G00-G99	Diseases of the nervous system	NA	Epilepsy, unspecified, intractable, with status epilepticus	0	0	1	0	0	0	1	1
G40919	G00-G99	Diseases of the nervous system	NA	Epilepsy, unspecified, intractable, without status epilepticus	0	0	1	0	0	1	0	1
G40A11	G00-G99	Diseases of the nervous system	NA	Absence epileptic syndrome, intractable, with status epilepticus	0	0	0	1	0	0	1	1
G40B	G00-G99	Diseases of the nervous system	NA	Juvenile myoclonic epilepsy [impulsive petit mal]	0	0	0	0	0	0	1	1
G40B0	G00-G99	Diseases of the nervous system	NA	Juvenile myoclonic epilepsy, not intractable	0	0	0	0	0	0	1	1
G40B01	G00-G99	Diseases of the nervous system	NA	Juvenile myoclonic epilepsy, not intractable, with status epilepticus	0	0	0	0	0	0	1	1
G40B09	G00-G99	Diseases of the nervous system	NA	Juvenile myoclonic epilepsy, not intractable, without status epilepticus	0	0	0	0	0	0	1	1
G40B1	G00-G99	Diseases of the nervous system	NA	Juvenile myoclonic epilepsy, intractable	0	0	1	0	0	1	0	1
G40B11	G00-G99	Diseases of the nervous system	NA	Juvenile myoclonic epilepsy, intractable, with status epilepticus	0	0	1	0	0	1	0	1
G40B19	G00-G99	Diseases of the nervous system	NA	Juvenile myoclonic epilepsy, intractable, without status epilepticus	0	0	1	0	0	1	0	1
G43A	G00-G99	Diseases of the nervous system	NA	Cyclical vomiting	0	0	0	0	0	0	1	
G43A1	G00-G99	Diseases of the nervous system	NA	Cyclical vomiting, intractable	0	0	0	0	0	0	1	
G4731	G00-G99	Diseases of the nervous system	NA	Primary central sleep apnea	0	0	0	1	0	0	1	
G4735	G00-G99	Diseases of the nervous system	NA	Congenital central alveolar hypoventilation syndrome	0	0	1	0	0	1	0	
G4737	G00-G99	Diseases of the nervous system	NA	Central sleep apnea in conditionsclassified elsewhere	0	0	0	1	0	0	1	
G60	G00-G99	Diseases of the nervous system	NA	Hereditary and idiopathic neuropathy	0	1	0	0	1	0	0	
G600	G00-G99	Diseases of the nervous system	NA	Hereditary motor and sensory neuropathy	0	1	0	0	1	0	0	
G601	G00-G99	Diseases of the nervous system	NA	Refsum's disease	1	1	0	0	1	0	0	
G602	G00-G99	Diseases of the nervous system	NA	Neuropathy in association with hereditary ataxia	0	1	0	0	1	0	0	
G603	G00-G99	Diseases of the nervous system	NA	Idiopathic progressive neuropathy	0	0	1	0	0	1	0	
G608	G00-G99	Diseases of the nervous system	NA	Other hereditary and idiopathic neuropathies	0	1	0	0	1	0	0	
G609	G00-G99	Diseases of the nervous system	NA	Hereditary and idiopathic neuropathy, unspecified	0	1	0	0	1	0	0	
G702	G00-G99	Diseases of the nervous system	NA	Congenital and developmental myasthenia	0	0	1	0	0	1	0	
G71	G00-G99	Diseases of the nervous system	NA	Primary disorders of muscles	0	0	1	0	0	1	0	
G710	G00-G99	Diseases of the nervous system	NA	Muscular dystrophy	1	1	0	0	1	0	0	
G711	G00-G99	Diseases of the nervous system	NA	Myotonic disorders	0	0	1	0	0	1	0	
G7111	G00-G99	Diseases of the nervous system	NA	Myotonic muscular dystrophy	1	1	0	0	1	0	0	
G7112	G00-G99	Diseases of the nervous system	NA	Myotonia congenita	1	1	0	0	1	0	0	
G7113	G00-G99	Diseases of the nervous system	NA	Myotonic chondrodystrophy	1	1	0	0	1	0	0	
G7119	G00-G99	Diseases of the nervous system	NA	Other specified myotonic disorders	0	0	0	1	0	0	1	
G712	G00-G99	Diseases of the nervous system	NA	Congenital myopathies	0	0	1	0	0	1	0	
G713	G00-G99	Diseases of the nervous system	NA	Mitochondrial myopathy, not elsewhere classified	0	1	0	0	1	0	0	
G718	G00-G99	Diseases of the nervous system	NA	Other primary disorders of muscles	0	0	1	0	0	1	0	
G719	G00-G99	Diseases of the nervous system	NA	Primary disorder of muscle, unspecified	0	0	1	0	0	1	0	
G72	G00-G99	Diseases of the nervous system	NA	Other and unspecified myopathies	0	0	1	0	0	1	0	
G723	G00-G99	Diseases of the nervous system	NA	Periodic paralysis	1	1	0	0	1	0	0	
G7289	G00-G99	Diseases of the nervous system	NA	Other specified myopathies	0	0	0	1	0	0	1	
G729	G00-G99	Diseases of the nervous system	NA	Myopathy, unspecified	0	0	0	1	0	0	1	
G80	G00-G99	Diseases of the nervous system	NA	Cerebral palsy	0	0	0	0	0	0	1	
G800	G00-G99	Diseases of the nervous system	NA	Spastic quadriplegic cerebral palsy	0	0	0	0	0	0	1	
G801	G00-G99	Diseases of the nervous system	NA	Spastic diplegic cerebral palsy	0	0	0	0	0	0	1	
G802	G00-G99	Diseases of the nervous system	NA	Spastic hemiplegic cerebral palsy	0	0	0	0	0	0	1	
G803	G00-G99	Diseases of the nervous system	NA	Athetoid cerebral palsy	0	0	0	0	0	0	1	
G804	G00-G99	Diseases of the nervous system	NA	Ataxic cerebral palsy	0	0	0	0	0	0	1	
G809	G00-G99	Diseases of the nervous system	NA	Cerebral palsy, unspecified	0	0	0	0	0	0	1	
G901	G00-G99	Diseases of the nervous system	NA	Familial dysautonomia [Riley-Day]	1	1	0	0	1	0	0	
G91	G00-G99	Diseases of the nervous system	NA	Hydrocephalus	0	0	0	1	0	0	1	
G910	G00-G99	Diseases of the nervous system	NA	Communicating hydrocephalus	0	0	0	1	0	0	1	
G911	G00-G99	Diseases of the nervous system	NA	Obstructive hydrocephalus	0	0	0	1	0	0	1	
G912	G00-G99	Diseases of the nervous system	NA	(Idiopathic) normal pressure hydrocephalus	0	0	0	1	0	0	1	
G918	G00-G99	Diseases of the nervous system	NA	Other hydrocephalus	0	0	0	1	0	0	1	
G919	G00-G99	Diseases of the nervous system	NA	Hydrocephalus, unspecified	0	0	0	1	0	0	1	
G9340	G00-G99	Diseases of the nervous system	NA	Encephalopathy, unspecified	0	0	0	1	0	0	0	
G9341	G00-G99	Diseases of the nervous system	NA	Metabolic encephalopathy	0	0	1	0	0	1	0	
G937	G00-G99	Diseases of the nervous system	NA	Reye's syndrome	0	0	0	0	0	0	1	
G950	G00-G99	Diseases of the nervous system	NA	Syringomyelia and syringobulbia	0	0	0	1	0	0	0	
G990	G00-G99	Diseases of the nervous system	NA	AUTONOMIC NEUROPATHY DZ CLASS ELSW	0	0	0	0	0	1	0	
H0252	H00-H59	Diseases of the eye and adnexa	NA	Blepharophimosis	0	0	0	1	0	0	1	
H02521	H00-H59	Diseases of the eye and adnexa	NA	Blepharophimosis right upper eyelid	0	0	0	1	0	0	1	
H02522	H00-H59	Diseases of the eye and adnexa	NA	Blepharophimosis right lower eyelid	0	0	0	1	0	0	1	
H02523	H00-H59	Diseases of the eye and adnexa	NA	Blepharophimosis right eye, unspecified eyelid	0	0	0	1	0	0	1	
H02524	H00-H59	Diseases of the eye and adnexa	NA	Blepharophimosis left upper eyelid	0	0	0	1	0	0	1	
H02525	H00-H59	Diseases of the eye and adnexa	NA	Blepharophimosis left lower eyelid	0	0	0	1	0	0	1	
H02526	H00-H59	Diseases of the eye and adnexa	NA	Blepharophimosis left eye, unspecified eyelid	0	0	0	1	0	0	1	
H02529	H00-H59	Diseases of the eye and adnexa	NA	Blepharophimosis unspecified eye,unspecified lid	0	0	0	1	0	0	1	
H18039	H00-H59	Diseases of the eye and adnexa	NA	Corneal deposits in metabolic disorders, unspecified eye	0	0	0	0	0	0	1	
H1804	H00-H59	Diseases of the eye and adnexa	NA	Kayser-Fleischer ring	1	0	0	0	1	0	0	
H18041	H00-H59	Diseases of the eye and adnexa	NA	Kayser-Fleischer ring, right eye	1	0	0	0	1	0	0	
H18042	H00-H59	Diseases of the eye and adnexa	NA	Kayser-Fleischer ring, left eye	1	0	0	0	1	0	0	
H18043	H00-H59	Diseases of the eye and adnexa	NA	Kayser-Fleischer ring, bilateral	1	0	0	0	1	0	0	
H18049	H00-H59	Diseases of the eye and adnexa	NA	Kayser-Fleischer ring, unspecifiedeye	1	0	0	0	1	0	0	
H185	H00-H59	Diseases of the eye and adnexa	NA	Hereditary corneal dystrophies	0	1	0	0	1	0	0	
H1850	H00-H59	Diseases of the eye and adnexa	NA	Unspecified hereditary corneal dystrophies	0	1	0	0	1	0	0	
H1851	H00-H59	Diseases of the eye and adnexa	NA	Endothelial corneal dystrophy	0	0	0	0	0	0	1	
H1852	H00-H59	Diseases of the eye and adnexa	NA	Epithelial (juvenile) corneal dystrophy	0	1	0	0	1	0	0	
H1853	H00-H59	Diseases of the eye and adnexa	NA	Granular corneal dystrophy	0	0	0	0	0	0	1	
H1854	H00-H59	Diseases of the eye and adnexa	NA	Lattice corneal dystrophy	1	1	0	0	1	0	0	
H1855	H00-H59	Diseases of the eye and adnexa	NA	Macular corneal dystrophy	0	0	0	0	0	0	1	
H1859	H00-H59	Diseases of the eye and adnexa	NA	Other hereditary corneal dystrophies	0	1	0	0	1	0	0	
H186	H00-H59	Diseases of the eye and adnexa	NA	Keratoconus	0	0	1	0	0	1	0	
H1860	H00-H59	Diseases of the eye and adnexa	NA	Keratoconus, unspecified	0	0	0	1	0	0	1	
H18601	H00-H59	Diseases of the eye and adnexa	NA	Keratoconus, unspecified, right eye	0	0	0	1	0	0	1	
H18602	H00-H59	Diseases of the eye and adnexa	NA	Keratoconus, unspecified, left eye	0	0	0	1	0	0	1	
H18603	H00-H59	Diseases of the eye and adnexa	NA	Keratoconus, unspecified, bilateral	0	0	0	1	0	0	1	
H18609	H00-H59	Diseases of the eye and adnexa	NA	Keratoconus, unspecified, unspecified eye	0	0	0	1	0	0	1	
H1861	H00-H59	Diseases of the eye and adnexa	NA	Keratoconus, stable	0	0	0	1	0	0	1	
H18611	H00-H59	Diseases of the eye and adnexa	NA	Keratoconus, stable, right eye	0	0	0	1	0	0	1	
H18612	H00-H59	Diseases of the eye and adnexa	NA	Keratoconus, stable, left eye	0	0	0	1	0	0	1	
H18613	H00-H59	Diseases of the eye and adnexa	NA	Keratoconus, stable, bilateral	0	0	0	1	0	0	1	
H18619	H00-H59	Diseases of the eye and adnexa	NA	Keratoconus, stable, unspecified eye	0	0	0	1	0	0	1	
H1862	H00-H59	Diseases of the eye and adnexa	NA	Keratoconus, unstable	0	0	0	1	0	0	1	
H18621	H00-H59	Diseases of the eye and adnexa	NA	Keratoconus, unstable, right eye	0	0	0	1	0	0	1	
H18622	H00-H59	Diseases of the eye and adnexa	NA	Keratoconus, unstable, left eye	0	0	0	1	0	0	1	
H18623	H00-H59	Diseases of the eye and adnexa	NA	Keratoconus, unstable, bilateral	0	0	1	0	0	1	0	
H18629	H00-H59	Diseases of the eye and adnexa	NA	Keratoconus, unstable, unspecifiedeye	0	0	0	1	0	0	1	
H1870	H00-H59	Diseases of the eye and adnexa	NA	Unspecified corneal deformity	0	0	0	0	0	0	1	
H18793	H00-H59	Diseases of the eye and adnexa	NA	OTHER CORNEAL DEFORMITIES BILATERAL	0	0	1	0	0	1	0	
H2125	H00-H59	Diseases of the eye and adnexa	NA	Iridoschisis	0	0	0	1	0	0	1	
H21251	H00-H59	Diseases of the eye and adnexa	NA	Iridoschisis, right eye	0	0	0	1	0	0	1	
H21252	H00-H59	Diseases of the eye and adnexa	NA	Iridoschisis, left eye	0	0	0	1	0	0	1	
H21253	H00-H59	Diseases of the eye and adnexa	NA	Iridoschisis, bilateral	0	0	0	1	0	0	1	
H21259	H00-H59	Diseases of the eye and adnexa	NA	Iridoschisis, unspecified eye	0	0	0	1	0	0	1	
H260	H00-H59	Diseases of the eye and adnexa	NA	Infantile and juvenile cataract	0	0	1	0	0	1	0	
H2600	H00-H59	Diseases of the eye and adnexa	NA	Unspecified infantile and juvenilecataract	0	0	1	0	0	1	0	
H26001	H00-H59	Diseases of the eye and adnexa	NA	Unspecified infantile and juvenilecataract, right eye	0	0	1	0	0	1	0	
H26002	H00-H59	Diseases of the eye and adnexa	NA	Unspecified infantile and juvenilecataract, left eye	0	0	1	0	0	1	0	
H26003	H00-H59	Diseases of the eye and adnexa	NA	Unspecified infantile and juvenilecataract, bilateral	0	0	1	0	0	1	0	
H26009	H00-H59	Diseases of the eye and adnexa	NA	Unspecified infantile and juvenilecataract, unspecified eye	0	0	1	0	0	1	0	
H2601	H00-H59	Diseases of the eye and adnexa	NA	Infantile and juvenile cortical, lamellar, or zonular cataract	0	0	1	0	0	1	0	
H26011	H00-H59	Diseases of the eye and adnexa	NA	Infantile and juvenile cortical, lamellar, or zonular cataract, right eye	0	0	1	0	0	1	0	
H26012	H00-H59	Diseases of the eye and adnexa	NA	Infantile and juvenile cortical, lamellar, or zonular cataract, left eye	0	0	1	0	0	1	0	
H26013	H00-H59	Diseases of the eye and adnexa	NA	Infantile and juvenile cortical, lamellar, or zonular cataract, bilateral	0	0	1	0	0	1	0	
H26019	H00-H59	Diseases of the eye and adnexa	NA	Infantile and juvenile cortical, lamellar, or zonular cataract, unspecified eye	0	0	1	0	0	1	0	
H2603	H00-H59	Diseases of the eye and adnexa	NA	Infantile and juvenile nuclear cataract	0	0	1	0	0	1	0	
H26031	H00-H59	Diseases of the eye and adnexa	NA	Infantile and juvenile nuclear cataract, right eye	0	0	1	0	0	1	0	
H26032	H00-H59	Diseases of the eye and adnexa	NA	Infantile and juvenile nuclear cataract, left eye	0	0	1	0	0	1	0	
H26033	H00-H59	Diseases of the eye and adnexa	NA	Infantile and juvenile nuclear cataract, bilateral	0	0	1	0	0	1	0	
H26039	H00-H59	Diseases of the eye and adnexa	NA	Infantile and juvenile nuclear cataract, unspecified eye	0	0	1	0	0	1	0	
H2604	H00-H59	Diseases of the eye and adnexa	NA	Anterior subcapsular polar infantile and juvenile cataract	0	0	1	0	0	1	0	
H26041	H00-H59	Diseases of the eye and adnexa	NA	Anterior subcapsular polar infantile and juvenile cataract, right eye	0	0	1	0	0	1	0	
H26042	H00-H59	Diseases of the eye and adnexa	NA	Anterior subcapsular polar infantile and juvenile cataract, left eye	0	0	1	0	0	1	0	
H26043	H00-H59	Diseases of the eye and adnexa	NA	Anterior subcapsular polar infantile and juvenile cataract, bilateral	0	0	1	0	0	1	0	
H26049	H00-H59	Diseases of the eye and adnexa	NA	Anterior subcapsular polar infantile and juvenile cataract, unspecified eye	0	0	1	0	0	1	0	
H2605	H00-H59	Diseases of the eye and adnexa	NA	Posterior subcapsular polar infantile and juvenile cataract	0	0	1	0	0	1	0	
H26051	H00-H59	Diseases of the eye and adnexa	NA	Posterior subcapsular polar infantile and juvenile cataract, right eye	0	0	1	0	0	1	0	
H26052	H00-H59	Diseases of the eye and adnexa	NA	Posterior subcapsular polar infantile and juvenile cataract, left eye	0	0	1	0	0	1	0	
H26053	H00-H59	Diseases of the eye and adnexa	NA	Posterior subcapsular polar infantile and juvenile cataract, bilateral	0	0	1	0	0	1	0	
H26059	H00-H59	Diseases of the eye and adnexa	NA	Posterior subcapsular polar infantile and juvenile cataract, unspecified eye	0	0	1	0	0	1	0	
H2606	H00-H59	Diseases of the eye and adnexa	NA	Combined forms of infantile and juvenile cataract	0	0	1	0	0	1	0	

H26061	H00-H59	Diseases of the eye and adnexa	NA	Combined forms of infantile and juvenile cataract, right eye	0	0	1	0	0	1	0
H26062	H00-H59	Diseases of the eye and adnexa	NA	Combined forms of infantile and juvenile cataract, left eye	0	0	1	0	0	1	0
H26063	H00-H59	Diseases of the eye and adnexa	NA	Combined forms of infantile and juvenile cataract, bilateral	0	0	1	0	0	1	0
H26069	H00-H59	Diseases of the eye and adnexa	NA	Combined forms of infantile and juvenile cataract, unspecified eye	0	0	1	0	0	1	0
H2609	H00-H59	Diseases of the eye and adnexa	NA	Other infantile and juvenile cataract	0	0	1	0	0	1	0
H268	H00-H59	Diseases of the eye and adnexa	NA	OTHER SPECIFIED CATARACT	0	0	1	0	0	0	1
H269	H00-H59	Diseases of the eye and adnexa	NA	UNSPECIFIED CATARACT	0	0	1	0	0	0	1
H2700	H00-H59	Diseases of the eye and adnexa	NA	APHAKIA UNSPECIFIED EYE	0	0	0	1	0	0	1
H2701	H00-H59	Diseases of the eye and adnexa	NA	APHAKIA RIGHT EYE	0	0	1	0	0	0	1
H2702	H00-H59	Diseases of the eye and adnexa	NA	APHAKIA LEFT EYE	0	0	0	1	0	0	1
H2703	H00-H59	Diseases of the eye and adnexa	NA	APHAKIA BILATERAL	0	0	1	0	0	0	1
H271	H00-H59	Diseases of the eye and adnexa	NA	Dislocation of lens	0	0	1	0	0	1	0
H2710	H00-H59	Diseases of the eye and adnexa	NA	Unspecified dislocation of lens	0	0	0	1	0	0	1
H2711	H00-H59	Diseases of the eye and adnexa	NA	Subluxation of lens	0	0	1	0	0	1	0
H27111	H00-H59	Diseases of the eye and adnexa	NA	Subluxation of lens, right eye	0	0	1	0	0	1	0
H27112	H00-H59	Diseases of the eye and adnexa	NA	Subluxation of lens, left eye	0	0	1	0	0	1	0
H27113	H00-H59	Diseases of the eye and adnexa	NA	Subluxation of lens, bilateral	0	0	1	0	0	1	0
H27119	H00-H59	Diseases of the eye and adnexa	NA	Subluxation of lens, unspecified eye	0	0	1	0	0	1	0
H2712	H00-H59	Diseases of the eye and adnexa	NA	Anterior dislocation of lens	0	0	1	0	0	1	0
H27121	H00-H59	Diseases of the eye and adnexa	NA	Anterior dislocation of lens, right eye	0	0	1	0	0	1	0
H27122	H00-H59	Diseases of the eye and adnexa	NA	Anterior dislocation of lens, lefteye	0	0	1	0	0	1	0
H27123	H00-H59	Diseases of the eye and adnexa	NA	Anterior dislocation of lens, bilateral	0	0	1	0	0	1	0
H27129	H00-H59	Diseases of the eye and adnexa	NA	Anterior dislocation of lens, unspecified eye	0	0	1	0	0	1	0
H2713	H00-H59	Diseases of the eye and adnexa	NA	Posterior dislocation of lens	0	0	1	0	0	1	0
H27131	H00-H59	Diseases of the eye and adnexa	NA	Posterior dislocation of lens, right eye	0	0	1	0	0	1	0
H27132	H00-H59	Diseases of the eye and adnexa	NA	Posterior dislocation of lens, left eye	0	0	1	0	0	1	0
H27133	H00-H59	Diseases of the eye and adnexa	NA	Posterior dislocation of lens, bilateral	0	0	1	0	0	1	0
H27139	H00-H59	Diseases of the eye and adnexa	NA	Posterior dislocation of lens, unspecified eye	0	0	1	0	0	1	0
H278	H00-H59	Diseases of the eye and adnexa	NA	Other specified disorders of lens	0	0	0	1	0	0	0
H31101	H00-H59	Diseases of the eye and adnexa	NA	Choroidal degeneration, unspecified, right eye	0	0	0	1	0	0	1
H312	H00-H59	Diseases of the eye and adnexa	NA	Hereditary choroidal dystrophy	0	1	0	0	1	0	0
H3120	H00-H59	Diseases of the eye and adnexa	NA	Hereditary choroidal dystrophy, unspecified	0	1	0	0	1	0	0
H3121	H00-H59	Diseases of the eye and adnexa	NA	Choroideremia	1	1	0	0	1	0	0
H3122	H00-H59	Diseases of the eye and adnexa	NA	Choroidal dystrophy (central areolar) (generalized) (peripapillary)	0	0	1	0	0	1	0
H3123	H00-H59	Diseases of the eye and adnexa	NA	Gyrate atrophy, choroid	1	1	0	0	1	0	0
H3129	H00-H59	Diseases of the eye and adnexa	NA	Other hereditary choroidal dystrophy	0	1	0	0	1	0	0
H331	H00-H59	Diseases of the eye and adnexa	NA	Retinoschisis and retinal cysts	0	0	1	0	0	1	0
H33101	H00-H59	Diseases of the eye and adnexa	NA	Unspecified retinoschisis, right eye	0	0	0	1	0	0	1
H33102	H00-H59	Diseases of the eye and adnexa	NA	Unspecified retinoschisis, left eye	0	0	0	1	0	0	1
H33103	H00-H59	Diseases of the eye and adnexa	NA	Unspecified retinoschisis, bilateral	0	0	0	1	0	0	1
H33109	H00-H59	Diseases of the eye and adnexa	NA	Unspecified retinoschisis, unspecified eye	0	0	0	1	0	0	1
H3319	H00-H59	Diseases of the eye and adnexa	NA	Other retinoschisis and retinal cysts	0	0	1	0	0	1	0
H33191	H00-H59	Diseases of the eye and adnexa	NA	Other retinoschisis and retinal cysts, right eye	0	0	1	0	0	1	0
H33192	H00-H59	Diseases of the eye and adnexa	NA	Other retinoschisis and retinal cysts, left eye	0	0	1	0	0	1	0
H33193	H00-H59	Diseases of the eye and adnexa	NA	Other retinoschisis and retinal cysts, bilateral	0	0	1	0	0	1	0
H33199	H00-H59	Diseases of the eye and adnexa	NA	Other retinoschisis and retinal cysts, unspecified eye	0	0	1	0	0	1	0
H3507	H00-H59	Diseases of the eye and adnexa	NA	Retinal telangiectasis	0	0	0	1	0	0	1
H35071	H00-H59	Diseases of the eye and adnexa	NA	Retinal telangiectasis, right eye	0	0	0	1	0	0	1
H35072	H00-H59	Diseases of the eye and adnexa	NA	Retinal telangiectasis, left eye	0	0	0	1	0	0	1
H35073	H00-H59	Diseases of the eye and adnexa	NA	Retinal telangiectasis, bilateral	0	0	0	1	0	0	1
H35079	H00-H59	Diseases of the eye and adnexa	NA	Retinal telangiectasis, unspecified eye	0	0	0	1	0	0	1
H355	H00-H59	Diseases of the eye and adnexa	NA	Hereditary retinal dystrophy	0	1	0	0	1	0	0
H3550	H00-H59	Diseases of the eye and adnexa	NA	Unspecified hereditary retinal dystrophy	0	1	0	0	1	0	0
H3551	H00-H59	Diseases of the eye and adnexa	NA	Vitreoretinal dystrophy	0	0	1	0	0	1	0
H3552	H00-H59	Diseases of the eye and adnexa	NA	Pigmentary retinal dystrophy	0	0	1	0	0	1	0
H3553	H00-H59	Diseases of the eye and adnexa	NA	Other dystrophies primarily involving the sensory retina	0	0	0	1	0	0	1
H3554	H00-H59	Diseases of the eye and adnexa	NA	Dystrophies primarily involving the retinal pigment epithelium	0	0	1	0	0	1	0
H401110	H00-H59	Diseases of the eye and adnexa	NA	Primary open-angle glaucoma, right eye, stage unspecified	0	0	0	1	0	0	1
H4703	H00-H59	Diseases of the eye and adnexa	NA	Optic nerve hypoplasia	0	0	1	0	0	1	0
H47031	H00-H59	Diseases of the eye and adnexa	NA	Optic nerve hypoplasia, right eye	0	0	1	0	0	1	0
H47032	H00-H59	Diseases of the eye and adnexa	NA	Optic nerve hypoplasia, left eye	0	0	1	0	0	1	0
H47033	H00-H59	Diseases of the eye and adnexa	NA	Optic nerve hypoplasia, bilateral	0	0	1	0	0	1	0
H47039	H00-H59	Diseases of the eye and adnexa	NA	Optic nerve hypoplasia, unspecified eye	0	0	1	0	0	1	0
H4720	H00-H59	Diseases of the eye and adnexa	NA	Unspecified optic atrophy	0	0	0	1	0	0	1
H47211	H00-H59	Diseases of the eye and adnexa	NA	PRIMARY OPTIC ATROPHY RIGHT EYE	0	0	1	0	0	1	0
H47212	H00-H59	Diseases of the eye and adnexa	NA	PRIMARY OPTIC ATROPHY LEFT EYE	0	0	1	0	0	1	0
H47213	H00-H59	Diseases of the eye and adnexa	NA	PRIMARY OPTIC ATROPHY BILATERAL	0	0	1	0	0	1	0
H47219	H00-H59	Diseases of the eye and adnexa	NA	Primary optic atrophy, unspecified eye	0	0	0	1	0	0	1
H4722	H00-H59	Diseases of the eye and adnexa	NA	Hereditary optic atrophy	0	1	0	0	1	0	0
H47233	H00-H59	Diseases of the eye and adnexa	NA	GLAUCOMATOUS OPTIC ATROPHY BILAT	0	0	1	0	0	0	0
H47292	H00-H59	Diseases of the eye and adnexa	NA	OTHER OPTIC ATROPHY LEFT EYE	0	0	1	0	0	0	0
H47293	H00-H59	Diseases of the eye and adnexa	NA	OTHER OPTIC ATROPHY BILATERAL	0	0	1	0	0	0	0
H47299	H00-H59	Diseases of the eye and adnexa	NA	Other optic atrophy, unspecified eye	0	0	0	1	0	0	1
H47311	H00-H59	Diseases of the eye and adnexa	NA	COLOBOMA OF OPTIC DISC RIGHT EYE	0	0	0	1	0	1	1
H47312	H00-H59	Diseases of the eye and adnexa	NA	COLOBOMA OF OPTIC DISC LEFT EYE	0	0	0	1	0	1	1
H47313	H00-H59	Diseases of the eye and adnexa	NA	COLOBOMA OF OPTIC DISC BILATERAL	0	0	0	1	0	1	1
H47319	H00-H59	Diseases of the eye and adnexa	NA	Coloboma of optic disc, unspecified eye	0	0	0	1	0	0	1
H494	H00-H59	Diseases of the eye and adnexa	NA	Progressive external ophthalmoplegia	0	0	1	0	0	1	0
H4940	H00-H59	Diseases of the eye and adnexa	NA	Progressive external ophthalmoplegia, unspecified eye	0	0	1	0	0	1	0
H4941	H00-H59	Diseases of the eye and adnexa	NA	Progressive external ophthalmoplegia, right eye	0	0	1	0	0	1	0
H4942	H00-H59	Diseases of the eye and adnexa	NA	Progressive external ophthalmoplegia, left eye	0	0	1	0	0	1	0
H4943	H00-H59	Diseases of the eye and adnexa	NA	Progressive external ophthalmoplegia, bilateral	0	0	1	0	0	1	0
H4981	H00-H59	Diseases of the eye and adnexa	NA	Kearns-Sayre syndrome	1	1	0	0	1	0	0
H49811	H00-H59	Diseases of the eye and adnexa	NA	Kearns-Sayre syndrome, right eye	1	1	0	0	1	0	0
H49812	H00-H59	Diseases of the eye and adnexa	NA	Kearns-Sayre syndrome, left eye	1	1	0	0	1	0	0
H49813	H00-H59	Diseases of the eye and adnexa	NA	Kearns-Sayre syndrome, bilateral	1	1	0	0	1	0	0
H49819	H00-H59	Diseases of the eye and adnexa	NA	Kearns-Sayre syndrome, unspecifiedeye	1	1	0	0	1	0	0
H5081	H00-H59	Diseases of the eye and adnexa	NA	Duane's syndrome	0	0	0	1	0	0	1
H50811	H00-H59	Diseases of the eye and adnexa	NA	Duane's syndrome, right eye	0	0	0	1	0	0	1
H50812	H00-H59	Diseases of the eye and adnexa	NA	Duane's syndrome, left eye	0	0	0	1	0	0	1
H5120	H00-H59	Diseases of the eye and adnexa	NA	Internuclear ophthalmoplegia, unspecified eye	0	0	0	1	0	0	1
H535	H00-H59	Diseases of the eye and adnexa	NA	Color vision deficiencies	1	1	0	0	1	0	0
H5350	H00-H59	Diseases of the eye and adnexa	NA	Unspecified color vision deficiencies	1	1	0	0	1	0	0
H5351	H00-H59	Diseases of the eye and adnexa	NA	Achromatopsia	1	1	0	0	1	0	0
H5353	H00-H59	Diseases of the eye and adnexa	NA	Deuteranomaly	1	1	0	0	1	0	0
H5354	H00-H59	Diseases of the eye and adnexa	NA	Protanomaly	1	1	0	0	1	0	0
H5355	H00-H59	Diseases of the eye and adnexa	NA	Tritanomaly	1	1	0	0	1	0	0
H5359	H00-H59	Diseases of the eye and adnexa	NA	Other color vision deficiencies	1	1	0	0	1	0	0
H5363	H00-H59	Diseases of the eye and adnexa	NA	Congenital night blindness	0	1	0	0	1	0	0
H540	H00-H59	Diseases of the eye and adnexa	NA	BLINDNESS BOTH EYES	0	0	0	1	0	0	1
H5500	H00-H59	Diseases of the eye and adnexa	NA	Unspecified nystagmus	0	0	0	1	0	0	1
H5501	H00-H59	Diseases of the eye and adnexa	NA	Congenital nystagmus	0	0	1	0	0	1	0
H90	H60-H95	Diseases of the ear and mastoid process	NA	Conductive and sensorineural hearing loss	0	0	1	0	0	1	0
H903	H60-H95	Diseases of the ear and mastoid process	NA	Sensorineural hearing loss, bilateral	0	0	1	0	0	1	0
H904	H60-H95	Diseases of the ear and mastoid process	NA	Sensorineural hearing loss, unilateral with unrestricted hearing on the contralateral sid	0	0	1	0	0	1	0
H9041	H60-H95	Diseases of the ear and mastoid process	NA	Sensorineural hearing loss, unilateral, right ear, with unrestricted hearing on the contra	0	0	1	0	0	1	0
H9042	H60-H95	Diseases of the ear and mastoid process	NA	Sensorineural hearing loss, unilateral, left ear, with unrestricted hearing on the contrala	0	0	1	0	0	1	0
H905	H60-H95	Diseases of the ear and mastoid process	NA	Unspecified sensorineural hearingloss	0	0	1	0	0	1	0
H906	H60-H95	Diseases of the ear and mastoid process	NA	Mixed conductive and sensorineuralhearing loss, bilateral	0	0	1	0	0	1	0
H907	H60-H95	Diseases of the ear and mastoid process	NA	Mixed conductive and sensorineuralhearing loss, unilateral with unrestricted hearing o	0	0	1	0	0	1	0
H9071	H60-H95	Diseases of the ear and mastoid process	NA	Mixed conductive and sensorineuralhearing loss, unilateral, right ear, with unrestricted	0	0	1	0	0	1	0
H9072	H60-H95	Diseases of the ear and mastoid process	NA	Mixed conductive and sensorineuralhearing loss, unilateral, left ear, with unrestricted h	0	0	1	0	0	1	0
H908	H60-H95	Diseases of the ear and mastoid process	NA	Mixed conductive and sensorineuralhearing loss, unspecified	0	0	1	0	0	1	0
H90A	H60-H95	Diseases of the ear and mastoid process	NA	Conductive and sensorineural hearing loss with restricted hearing on the contralateral	0	0	1	0	0	1	0
H90A2	H60-H95	Diseases of the ear and mastoid process	NA	Sensorineural hearing loss, unilateral, with restricted hearing on the contralateral side	0	0	1	0	0	1	0
H90A21	H60-H95	Diseases of the ear and mastoid process	NA	Sensorineural hearing loss, unilateral, right ear, with restricted hearing on the contralat	0	0	1	0	0	1	0
H90A22	H60-H95	Diseases of the ear and mastoid process	NA	Sensorineural hearing loss, unilateral, left ear, with restricted hearing on the contralate	0	0	1	0	0	1	0
H90A3	H60-H95	Diseases of the ear and mastoid process	NA	Mixed conductive and sensorineuralhearing loss, unilateral with restricted hearing on t	0	0	1	0	0	1	0
H90A31	H60-H95	Diseases of the ear and mastoid process	NA	Mixed conductive and sensorineuralhearing loss, unilateral, right ear with restricted he	0	0	1	0	0	1	0
H90A32	H60-H95	Diseases of the ear and mastoid process	NA	Mixed conductive and sensorineuralhearing loss, unilateral, left ear with restricted hea	0	0	1	0	0	1	0
H913	H60-H95	Diseases of the ear and mastoid process	NA	Deaf nonspeaking, not elsewhere classified	0	0	0	0	0	0	1
H918X9	H60-H95	Diseases of the ear and mastoid process	NA	Other specified hearing loss, unspecified ear	0	0	0	0	0	0	1
I150	I00-I99	Diseases of the circulatory system	NA	Renovascular hypertension	0	0	0	0	0	0	1
I2541	I00-I99	Diseases of the circulatory system	NA	CORONARY ARTERY ANEURYSM	0	0	0	1	0	0	0
I270	I00-I99	Diseases of the circulatory system	NA	Primary pulmonary hypertension	0	0	0	0	0	1	0
I2720	I00-I99	Diseases of the circulatory system	NA	Pulmonary hypertension, unspecified	0	0	0	0	0	0	1
I2783	I00-I99	Diseases of the circulatory system	NA	Eisenmenger's syndrome	0	0	0	0	0	0	1
I340	I00-I99	Diseases of the circulatory system	NA	Nonrheumatic mitral (valve) insufficiency	0	0	0	1	0	0	1
I341	I00-I99	Diseases of the circulatory system	NA	Nonrheumatic mitral (valve) prolapse	0	0	0	1	0	0	1
I342	I00-I99	Diseases of the circulatory system	NA	Nonrheumatic mitral (valve) stenosis	0	0	0	1	0	0	1
I348	I00-I99	Diseases of the circulatory system	NA	Other nonrheumatic mitral valve disorders	0	0	0	1	0	0	1
I349	I00-I99	Diseases of the circulatory system	NA	Nonrheumatic mitral valve disorder, unspecified	0	0	0	1	0	0	1
I350	I00-I99	Diseases of the circulatory system	NA	Nonrheumatic aortic (valve) stenosis	0	0	0	1	0	0	1
I351	I00-I99	Diseases of the circulatory system	NA	Nonrheumatic aortic (valve) insufficiency	0	0	0	1	0	0	1
I352	I00-I99	Diseases of the circulatory system	NA	Nonrheumatic aortic (valve) stenosis with insufficiency	0	0	0	1	0	0	1
I358	I00-I99	Diseases of the circulatory system	NA	Other nonrheumatic aortic valve disorders	0	0	0	1	0	0	1
I359	I00-I99	Diseases of the circulatory system	NA	Nonrheumatic aortic valve disorder, unspecified	0	0	0	1	0	0	1
I360	I00-I99	Diseases of the circulatory system	NA	Nonrheumatic tricuspid (valve) stenosis	0	0	0	1	0	0	1
I361	I00-I99	Diseases of the circulatory system	NA	Nonrheumatic tricuspid (valve) insufficiency	0	0	0	1	0	0	1
I362	I00-I99	Diseases of the circulatory system	NA	Nonrheumatic tricuspid (valve) stenosis with insufficiency	0	0	0	1	0	0	1
I368	I00-I99	Diseases of the circulatory system	NA	Other nonrheumatic tricuspid valve disorders	0	0	0	1	0	0	1
I369	I00-I99	Diseases of the circulatory system	NA	Nonrheumatic tricuspid valve disorder, unspecified	0	0	0	1	0	0	1
I370	I00-I99	Diseases of the circulatory system	NA	Nonrheumatic pulmonary valve stenosis	0	0	0	1	0	0	1
I371	I00-I99	Diseases of the circulatory system	NA	Nonrheumatic pulmonary valve insufficiency	0	0	0	1	0	0	1
I372	I00-I99	Diseases of the circulatory system	NA	Nonrheumatic pulmonary valve stenosis with insufficiency	0	0	0	1	0	0	1
I378	I00-I99	Diseases of the circulatory system	NA	Other nonrheumatic pulmonary valve disorders	0	0	0	1	0	0	1
I379	I00-I99	Diseases of the circulatory system	NA	Nonrheumatic pulmonary valve disorder, unspecified	0	0	0	1	0	0	1
I42	I00-I99	Diseases of the circulatory system	NA	CARDIOMYOPATHY	0	0	0	1	0	0	1
I420	I00-I99	Diseases of the circulatory system	NA	Dilated cardiomyopathy	0	0	0	1	0	0	1
I421	I00-I99	Diseases of the circulatory system	NA	Obstructive hypertrophic cardiomyopathy	0	0	1	0	0	1	0
I422	I00-I99	Diseases of the circulatory system	NA	Other hypertrophic cardiomyopathy	0	0	0	1	0	0	1
I424	I00-I99	Diseases of the circulatory system	NA	Endocardial fibroelastosis	0	0	0	1	0	0	1
I425	I00-I99	Diseases of the circulatory system	NA	Other restrictive cardiomyopathy	0	0	0	1	0	0	1
I428	I00-I99	Diseases of the circulatory system	NA	Other cardiomyopathies	0	0	0	1	0	0	1
I429	I00-I99	Diseases of the circulatory system	NA	Cardiomyopathy, unspecified	0	0	0	1	0	0	1
I43	I00-I99	Diseases of the circulatory system	NA	CARDIOMYOPATHY DZ CLASSIFIED ELSW	0	0	0	0	0	0	1
I440	I00-I99	Diseases of the circulatory system	NA	Atrioventricular block, first degree	0	0	0	1	0	0	1
I441	I00-I99	Diseases of the circulatory system	NA	Atrioventricular block, second degree	0	0	0	1	0	0	1
I442	I00-I99	Diseases of the circulatory system	NA	Atrioventricular block, complete	0	0	0	1	0	0	0
I444	I00-I99	Diseases of the circulatory system	NA	Left anterior fascicular block	0	0	0	1	0	0	0
I447	I00-I99	Diseases of the circulatory system	NA	Left bundle-branch block, unspecified	0	0	0	1	0	0	0
I4510	I00-I99	Diseases of the circulatory system	NA	Unspecified right bundle-branch block	0	0	0	1	0	0	0
I456	I00-I99	Diseases of the circulatory system	NA	Pre-excitation syndrome	0	0	0	1	0	0	1
I4581	I00-I99	Diseases of the circulatory system	NA	Long QT syndrome	0	1	0	0	1	0	0
I4589	I00-I99	Diseases of the circulatory system	NA	Other specified conduction disorders	0	0	0	1	0	0	0
I459	I00-I99	Diseases of the circulatory system	NA	Conduction disorder, unspecified	0	0	0	1	0	0	0
I470	I00-I99	Diseases of the circulatory system	NA	Re-entry ventricular arrhythmia	0	0	0	1	0	0	1
I472	I00-I99	Diseases of the circulatory system	NA	VENTRICULAR TACHYCARDIA	0	0	1	0	0	1	0
I481	I00-I99	Diseases of the circulatory system	NA	PERSISTENT ATRIAL FIBRILLATION	0	0	1	0	0	1	0
I482	I00-I99	Diseases of the circulatory system	NA	CHRONIC ATRIAL FIBRILLATION	0	0	1	0	0	1	0
I4891	I00-I99	Diseases of the circulatory system	NA	Unspecified atrial fibrillation	0	0	0	1	0	0	0
I4901	I00-I99	Diseases of the circulatory system	NA	Ventricular fibrillation	0	0	0	1	0	0	0
I4902	I00-I99	Diseases of the circulatory system	NA	Ventricular flutter	0	0	0	1	0	0	0
I495	I00-I99	Diseases of the circulatory system	NA	Sick sinus syndrome	0	0	0	1	0	0	1
I499	I00-I99	Diseases of the circulatory system	NA	Cardiac arrhythmia, unspecified	0	0	0	1	0	0	0
I501	I00-I99	Diseases of the circulatory system	NA	Left ventricular failure, unspecified	0	0	0	1	0	0	0
I5020	I00-I99	Diseases of the circulatory system	NA	Unspecified systolic (congestive) heart failure	0	0	0	1	0	0	1
I5021	I00-I99	Diseases of the circulatory system	NA	Acute systolic (congestive) heart failure	0	0	0	1	0	0	1
I5022	I00-I99	Diseases of the circulatory system	NA	Chronic systolic (congestive) heart failure	0	0	0	1	0	0	1
I5023	I00-I99	Diseases of the circulatory system	NA	Acute on chronic systolic (congestive) heart failure	0	0	0	1	0	0	1
I5030	I00-I99	Diseases of the circulatory system	NA	Unspecified diastolic (congestive) heart failure	0	0	0	1	0	0	1
I5031	I00-I99	Diseases of the circulatory system	NA	Acute diastolic (congestive) heart failure	0	0	0	1	0	0	1
I5032	I00-I99	Diseases of the circulatory system	NA	Chronic diastolic (congestive) heart failure	0	0	0	1	0	0	1
I5033	I00-I99	Diseases of the circulatory system	NA	Acute on chronic diastolic (congestive) heart failure	0	0	0	1	0	0	1
I5040	I00-I99	Diseases of the circulatory system	NA	Unspecified combined systolic (congestive) and diastolic (congestive) heart failure	0	0	0	1	0	0	1
I5041	I00-I99	Diseases of the circulatory system	NA	Acute combined systolic (congestive) and diastolic (congestive) heart failure	0	0	0	1	0	0	1
I5042	I00-I99	Diseases of the circulatory system	NA	Chronic combined systolic (congestive) and diastolic (congestive) heart failure	0	0	0	1	0	0	1
I5043	I00-I99	Diseases of the circulatory system	NA	Acute on chronic combined systolic (congestive) and diastolic (congestive) heart failur	0	0	0	1	0	0	1
I50810	I00-I99	Diseases of the circulatory system	NA	Right heart failure, unspecified	0	0	0	1	0	0	0
I50811	I00-I99	Diseases of the circulatory system	NA	Acute right heart failure	0	0	0	1	0	0	1
I50812	I00-I99	Diseases of the circulatory system	NA	Chronic right heart failure	0	0	0	1	0	0	1
I50813	I00-I99	Diseases of the circulatory system	NA	Acute on chronic right heart failure	0	0	0	1	0	0	1
I50814	I00-I99	Diseases of the circulatory system	NA	Right heart failure due to left heart failure	0	0	0	1	0	0	0
I5082	I00-I99	Diseases of the circulatory system	NA	Biventricular heart failure	0	0	0	1	0	0	1
I5083	I00-I99	Diseases of the circulatory system	NA	High output heart failure	0	0	0	1	0	0	1
I5084	I00-I99	Diseases of the circulatory system	NA	End stage heart failure	0	0	0	1	0	0	1
I5089	I00-I99	Diseases of the circulatory system	NA	Other heart failure	0	0	0	1	0	0	1
I517	I00-I99	Diseases of the circulatory system	NA	Cardiomegaly	0	0	0	1	0	0	0
I670	I00-I99	Diseases of the circulatory system	NA	Dissection of cerebral arteries, nonruptured	0	0	0	1	0	0	1
I671	I00-I99	Diseases of the circulatory system	NA	Cerebral aneurysm, nonruptured	0	0	0	1	0	0	1
I673	I00-I99	Diseases of the circulatory system	NA	Progressive vascular leukoencephalopathy	0	0	0	1	0	0	1
I675	I00-I99	Diseases of the circulatory system	NA	Moyamoya disease	0	0	0	1	0	0	1
I708	I00-I99	Diseases of the circulatory system	NA	ATHEROSCLEROSIS OF OTHER ARTERIES	0	0	1	0	0	1	0
I7090	I00-I99	Diseases of the circulatory system	NA	UNSPECIFIED ATHEROSCLEROSIS	0	0	1	0	0	1	0
I7091	I00-I99	Diseases of the circulatory system	NA	Generalized atherosclerosis	0	0	0	1	0	0	1
I71	I00-I99	Diseases of the circulatory system	NA	Aortic aneurysm and dissection	0	0	0	1	0	0	1
I710	I00-I99	Diseases of the circulatory system	NA	Dissection of aorta	0	0	0	1	0	0	1
I7100	I00-I99	Diseases of the circulatory system	NA	Dissection of unspecified site ofaorta	0	0	0	1	0	0	1
I7101	I00-I99	Diseases of the circulatory system	NA	Dissection of thoracic aorta	0	0	0	1	0	0	0
I7102	I00-I99	Diseases of the circulatory system	NA	DISSECTION OF ABDOMINAL AORTA	0	0	0	0	0	1	0
I7103	I00-I99	Diseases of the circulatory system	NA	DISSECTION THORACOABDOMINAL AORTA	0	0	0	0	0	0	1
I711	I00-I99	Diseases of the circulatory system	NA	Thoracic aortic aneurysm, ruptured	0	0	0	1	0	0	1
I712	I00-I99	Diseases of the circulatory system	NA	Thoracic aortic aneurysm, withoutrupture	0	0	0	1	0	0	1
I714	I00-I99	Diseases of the circulatory system	NA	ABDOMINAL AORTIC ANEUR W/O RUPTURED	0	0	0	0	0	0	1
I715	I00-I99	Diseases of the circulatory system	NA	THORACOABDOMINAL AA RUPTURED	0	0	0	0	0	0	1
I716	I00-I99	Diseases of the circulatory system	NA	THORACOABDOMINAL AA W/O RUPTURE	0	0	0	0	0	0	1
I719	I00-I99	Diseases of the circulatory system	NA	Aortic aneurysm of unspecified site, without rupture	0	0	0	1	0	0	0
I720	I00-I99	Diseases of the circulatory system	NA	Aneurysm of carotid artery	0	0	0	1	0	0	0
I721	I00-I99	Diseases of the circulatory system	NA	Aneurysm of artery of upper extremity	0	0	0	1	0	0	0
I722	I00-I99	Diseases of the circulatory system	NA	Aneurysm of renal artery	0	0	0	1	0	0	0
I723	I00-I99	Diseases of the circulatory system	NA	Aneurysm of iliac artery	0	0	0	1	0	0	0
I724	I00-I99	Diseases of the circulatory system	NA	Aneurysm of artery of lower extremity	0	0	0	1	0	0	0
I726	I00-I99	Diseases of the circulatory system	NA	ANEURYSM OF VERTEBRAL ARTERY	0	0	0	0	0	0	1
I728	I00-I99	Diseases of the circulatory system	NA	Aneurysm of other specified arteries	0	0	0	1	0	0	0
I729	I00-I99	Diseases of the circulatory system	NA	ANEURYSM OF UNSPECIFIED SITE	0	0	0	0	0	0	1
I7381	I00-I99	Diseases of the circulatory system	NA	Erythromelalgia	0	0	0	0	0	0	1
I7771	I00-I99	Diseases of the circulatory system	NA	DISSECTION OF CAROTID ARTERY	0	0	0	0	0	0	1
I7772	I00-I99	Diseases of the circulatory system	NA	DISSECTION OF ILIAC ARTERY	0	0	0	0	0	0	1

I7774	I00-I99	Diseases of the circulatory system	NA	DISSECTION OF VERTEBRAL ARTERY	0	0	0	0	0	0	1	
I7779	I00-I99	Diseases of the circulatory system	NA	DISSECTION OF OTH SPECIFIED ARTERY	0	0	0	0	0	0	1	
I7781	I00-I99	Diseases of the circulatory system	NA	Aortic ectasia	0	0	1	0	0	1	0	
I77810	I00-I99	Diseases of the circulatory system	NA	Thoracic aortic ectasia	0	0	1	0	0	1	0	
I77819	I00-I99	Diseases of the circulatory system	NA	Aortic ectasia, unspecified site	0	0	0	1	0	0	0	
I780	I00-I99	Diseases of the circulatory system	NA	Hereditary hemorrhagic telangiectasia	0	1	0	0	1	0	0	
I790	I00-I99	Diseases of the circulatory system	NA	Aneurysm of aorta in diseases classified elsewhere	0	0	0	1	0	0	1	
I890	I00-I99	Diseases of the circulatory system	NA	Lymphedema, not elsewhere classified	0	0	0	1	0	0	1	
J471	J00-J99	Diseases of the respiratory system	NA	Bronchiectasis with (acute) exacerbation	0	0	0	1	0	0	0	
J479	J00-J99	Diseases of the respiratory system	NA	Bronchiectasis, uncomplicated	0	0	0	1	0	0	0	
J8401	J00-J99	Diseases of the respiratory system	NA	Alveolar proteinosis	0	0	0	1	0	0	0	
J8403	J00-J99	Diseases of the respiratory system	NA	Idiopathic pulmonary hemosiderosis	0	0	0	1	0	0	0	
J84111	J00-J99	Diseases of the respiratory system	NA	Idiopathic interstitial pneumonia, not otherwise specified	0	0	0	1	0	0	0	
J84112	J00-J99	Diseases of the respiratory system	NA	Idiopathic pulmonary fibrosis	0	0	0	1	0	0	0	
J84113	J00-J99	Diseases of the respiratory system	NA	Idiopathic non-specific interstitial pneumonitis	0	0	0	1	0	0	0	
J84117	J00-J99	Diseases of the respiratory system	NA	Desquamative interstitial pneumonia	0	0	0	1	0	0	0	
J842	J00-J99	Diseases of the respiratory system	NA	Lymphoid interstitial pneumonia	0	0	0	1	0	0	0	
J8481	J00-J99	Diseases of the respiratory system	NA	Lymphangioleiomyomatosis	0	0	0	1	0	0	0	
J8483	J00-J99	Diseases of the respiratory system	NA	Surfactant mutations of the lung	0	0	0	1	0	0	0	
J84843	J00-J99	Diseases of the respiratory system	NA	Alveolar capillary dysplasia with vein misalignment	0	0	0	1	0	0	0	
J930	J00-J99	Diseases of the respiratory system	NA	Spontaneous tension pneumothorax	0	0	0	0	0	0	1	
J9311	J00-J99	Diseases of the respiratory system	NA	Primary spontaneous pneumothorax	0	0	0	0	0	0	1	
K000	K00-K95	Diseases of the digestive system	NA	Anodontia	0	0	0	0	0	0	1	
K004	K00-K95	Diseases of the digestive system	NA	Disturbances in tooth formation	0	0	0	0	0	0	1	
K005	K00-K95	Diseases of the digestive system	NA	Hereditary disturbances in tooth structure, not elsewhere classified	0	1	0	0	1	0	0	
K090	K00-K95	Diseases of the digestive system	NA	Developmental odontogenic cysts	0	0	0	1	0	0	1	
K440	K00-K95	Diseases of the digestive system	NA	Diaphragmatic hernia with obstruction, without gangrene	0	0	0	1	0	0	0	
K441	K00-K95	Diseases of the digestive system	NA	Diaphragmatic hernia with gangrene	0	0	0	1	0	0	0	
K449	K00-K95	Diseases of the digestive system	NA	Diaphragmatic hernia without obstruction or gangrene	0	0	0	1	0	0	0	
K5939	K00-K95	Diseases of the digestive system	NA	Other megacolon	0	0	0	1	0	0	0	
K861	K00-K95	Diseases of the digestive system	NA	Other chronic pancreatitis	0	0	0	0	0	0	1	
L603	L00-L99	Diseases of the skin and subcutaneous syste	NA	Nail dystrophy	0	0	1	0	0	1	0	
L744	L00-L99	Diseases of the skin and subcutaneous syste	NA	ANHIDROSIS	0	0	1	0	0	0	0	
L816	L00-L99	Diseases of the skin and subcutaneous syste	NA	DISORDERS DIMINISHED MELANIN FORM	0	0	1	0	0	0	0	
L818	L00-L99	Diseases of the skin and subcutaneous syste	NA	OTHER SPEC DISORDERS PIGMENTATION	0	0	1	0	0	0	0	
L819	L00-L99	Diseases of the skin and subcutaneous syste	NA	DISORDER PIGMENTATION UNSPECIFIED	0	0	1	0	0	0	0	
M04	M00-M99	Diseases of the musculoskeletal system and	NA	Autoinflammatory syndromes	0	0	1	0	0	1	0	
M041	M00-M99	Diseases of the musculoskeletal system and	NA	Periodic fever syndromes	0	0	0	1	0	0	0	
M042	M00-M99	Diseases of the musculoskeletal system and	NA	Cryopyrin-associated periodic syndromes	0	1	0	0	1	0	0	
M048	M00-M99	Diseases of the musculoskeletal system and	NA	Other autoinflammatory syndromes	0	0	0	1	0	0	0	
M049	M00-M99	Diseases of the musculoskeletal system and	NA	Autoinflammatory syndrome, unspecified	0	0	1	0	0	1	0	
M109	M00-M99	Diseases of the musculoskeletal system and	NA	Gout, unspecified	0	0	0	0	0	0	1	
M111	M00-M99	Diseases of the musculoskeletal system and	NA	Familial chondrocalcinosis	0	0	1	0	0	1	0	
M1110	M00-M99	Diseases of the musculoskeletal system and	NA	Familial chondrocalcinosis, unspecified site	0	0	1	0	0	1	0	
M1111	M00-M99	Diseases of the musculoskeletal system and	NA	Familial chondrocalcinosis, shoulder	0	0	1	0	0	1	0	
M11111	M00-M99	Diseases of the musculoskeletal system and	NA	Familial chondrocalcinosis, rightshoulder	0	0	1	0	0	1	0	
M11112	M00-M99	Diseases of the musculoskeletal system and	NA	Familial chondrocalcinosis, left shoulder	0	0	1	0	0	1	0	
M11119	M00-M99	Diseases of the musculoskeletal system and	NA	Familial chondrocalcinosis, unspecified shoulder	0	0	1	0	0	1	0	
M1112	M00-M99	Diseases of the musculoskeletal system and	NA	Familial chondrocalcinosis, elbow	0	0	1	0	0	1	0	
M11121	M00-M99	Diseases of the musculoskeletal system and	NA	Familial chondrocalcinosis, rightelbow	0	0	1	0	0	1	0	
M11122	M00-M99	Diseases of the musculoskeletal system and	NA	Familial chondrocalcinosis, left elbow	0	0	1	0	0	1	0	
M11129	M00-M99	Diseases of the musculoskeletal system and	NA	Familial chondrocalcinosis, unspecified elbow	0	0	1	0	0	1	0	
M1113	M00-M99	Diseases of the musculoskeletal system and	NA	Familial chondrocalcinosis, wrist	0	0	1	0	0	1	0	
M11131	M00-M99	Diseases of the musculoskeletal system and	NA	Familial chondrocalcinosis, rightwrist	0	0	1	0	0	1	0	
M11132	M00-M99	Diseases of the musculoskeletal system and	NA	Familial chondrocalcinosis, left wrist	0	0	1	0	0	1	0	
M11139	M00-M99	Diseases of the musculoskeletal system and	NA	Familial chondrocalcinosis, unspecified wrist	0	0	1	0	0	1	0	
M1114	M00-M99	Diseases of the musculoskeletal system and	NA	Familial chondrocalcinosis, hand	0	0	1	0	0	1	0	
M11141	M00-M99	Diseases of the musculoskeletal system and	NA	Familial chondrocalcinosis, righthand	0	0	1	0	0	1	0	
M11142	M00-M99	Diseases of the musculoskeletal system and	NA	Familial chondrocalcinosis, left hand	0	0	1	0	0	1	0	
M11149	M00-M99	Diseases of the musculoskeletal system and	NA	Familial chondrocalcinosis, unspecified hand	0	0	1	0	0	1	0	
M1115	M00-M99	Diseases of the musculoskeletal system and	NA	Familial chondrocalcinosis, hip	0	0	1	0	0	1	0	
M11151	M00-M99	Diseases of the musculoskeletal system and	NA	Familial chondrocalcinosis, righthip	0	0	1	0	0	1	0	
M11152	M00-M99	Diseases of the musculoskeletal system and	NA	Familial chondrocalcinosis, left hip	0	0	1	0	0	1	0	
M11159	M00-M99	Diseases of the musculoskeletal system and	NA	Familial chondrocalcinosis, unspecified hip	0	0	1	0	0	1	0	
M1116	M00-M99	Diseases of the musculoskeletal system and	NA	Familial chondrocalcinosis, knee	0	0	1	0	0	1	0	
M11161	M00-M99	Diseases of the musculoskeletal system and	NA	Familial chondrocalcinosis, rightknee	0	0	1	0	0	1	0	
M11162	M00-M99	Diseases of the musculoskeletal system and	NA	Familial chondrocalcinosis, left knee	0	0	1	0	0	1	0	
M11169	M00-M99	Diseases of the musculoskeletal system and	NA	Familial chondrocalcinosis, unspecified knee	0	0	1	0	0	1	0	
M1117	M00-M99	Diseases of the musculoskeletal system and	NA	Familial chondrocalcinosis, ankleand foot	0	0	1	0	0	1	0	
M11171	M00-M99	Diseases of the musculoskeletal system and	NA	Familial chondrocalcinosis, rightankle and foot	0	0	1	0	0	1	0	
M11172	M00-M99	Diseases of the musculoskeletal system and	NA	Familial chondrocalcinosis, left ankle and foot	0	0	1	0	0	1	0	
M11179	M00-M99	Diseases of the musculoskeletal system and	NA	Familial chondrocalcinosis, unspecified ankle and foot	0	0	1	0	0	1	0	
M1118	M00-M99	Diseases of the musculoskeletal system and	NA	Familial chondrocalcinosis, vertebrae	0	0	1	0	0	1	0	
M1119	M00-M99	Diseases of the musculoskeletal system and	NA	Familial chondrocalcinosis, multiple sites	0	0	1	0	0	1	0	
M1A00X1	M00-M99	Diseases of the musculoskeletal system and	NA	Idiopathic chronic gout, unspecified site, with tophus (tophi)	0	0	0	0	0	0	1	
M1A9XX0	M00-M99	Diseases of the musculoskeletal system and	NA	Chronic gout, unspecified, without tophus (tophi)	0	0	0	0	0	0	1	
M20039	M00-M99	Diseases of the musculoskeletal system and	NA	Swan-neck deformity of unspecified finger(s)	0	0	0	0	0	0	1	
M2420	M00-M99	Diseases of the musculoskeletal system and	NA	Disorder of ligament, unspecified site	0	0	0	0	0	0	1	
M2440	M00-M99	Diseases of the musculoskeletal system and	NA	Recurrent dislocation, unspecified joint	0	0	0	0	0	0	1	
M24419	M00-M99	Diseases of the musculoskeletal system and	NA	Recurrent dislocation, unspecified shoulder	0	0	0	0	0	0	1	
M24429	M00-M99	Diseases of the musculoskeletal system and	NA	Recurrent dislocation, unspecified elbow	0	0	0	0	0	0	1	
M24439	M00-M99	Diseases of the musculoskeletal system and	NA	Recurrent dislocation, unspecified wrist	0	0	0	0	0	0	1	
M24443	M00-M99	Diseases of the musculoskeletal system and	NA	Recurrent dislocation, unspecified hand	0	0	0	0	0	0	1	
M24459	M00-M99	Diseases of the musculoskeletal system and	NA	Recurrent dislocation, unspecified hip	0	0	0	0	0	0	1	
M24469	M00-M99	Diseases of the musculoskeletal system and	NA	Recurrent dislocation, unspecified knee	0	0	0	0	0	0	1	
M24473	M00-M99	Diseases of the musculoskeletal system and	NA	Recurrent dislocation, unspecified ankle	0	0	0	0	0	0	1	
M24519	M00-M99	Diseases of the musculoskeletal system and	NA	Contracture, unspecified shoulder	0	0	0	0	0	0	1	
M24529	M00-M99	Diseases of the musculoskeletal system and	NA	Contracture, unspecified elbow	0	0	0	0	0	0	1	
M24539	M00-M99	Diseases of the musculoskeletal system and	NA	Contracture, unspecified wrist	0	0	0	0	0	0	1	
M24549	M00-M99	Diseases of the musculoskeletal system and	NA	Contracture, unspecified hand	0	0	0	0	0	0	1	
M24559	M00-M99	Diseases of the musculoskeletal system and	NA	Contracture, unspecified hip	0	0	0	0	0	0	1	
M24569	M00-M99	Diseases of the musculoskeletal system and	NA	Contracture, unspecified knee	0	0	0	0	0	0	1	
M24573	M00-M99	Diseases of the musculoskeletal system and	NA	Contracture, unspecified ankle	0	0	0	0	0	0	1	
M24819	M00-M99	Diseases of the musculoskeletal system and	NA	Other specific joint derangements of unspecified shoulder, not elsewhere classified	0	0	0	0	0	0	1	
M24829	M00-M99	Diseases of the musculoskeletal system and	NA	Other specific joint derangements of unspecified elbow, not elsewhere classified	0	0	0	0	0	0	1	
M24839	M00-M99	Diseases of the musculoskeletal system and	NA	Other specific joint derangements of unspecified wrist, not elsewhere classified	0	0	0	0	0	0	1	
M24849	M00-M99	Diseases of the musculoskeletal system and	NA	Other specific joint derangements of unspecified hand, not elsewhere classified	0	0	0	0	0	0	1	
M24859	M00-M99	Diseases of the musculoskeletal system and	NA	Other specific joint derangements of unspecified hip, not elsewhere classified	0	0	0	0	0	0	1	
M24873	M00-M99	Diseases of the musculoskeletal system and	NA	Other specific joint derangements of unspecified ankle, not elsewhere classified	0	0	0	0	0	0	1	
M260	M00-M99	Diseases of the musculoskeletal system and	NA	Major anomalies of jaw size	0	0	0	1	0	0	1	
M2600	M00-M99	Diseases of the musculoskeletal system and	NA	Unspecified anomaly of jaw size	0	0	0	1	0	0	1	
M2602	M00-M99	Diseases of the musculoskeletal system and	NA	Maxillary hypoplasia	0	0	0	1	0	0	1	
M2604	M00-M99	Diseases of the musculoskeletal system and	NA	Mandibular hypoplasia	0	0	0	1	0	0	1	
M2606	M00-M99	Diseases of the musculoskeletal system and	NA	Microgenia	0	0	0	1	0	0	1	
M2673	M00-M99	Diseases of the musculoskeletal system and	NA	Alveolar maxillary hypoplasia	0	0	0	1	0	0	1	
M2674	M00-M99	Diseases of the musculoskeletal system and	NA	Alveolar mandibular hypoplasia	0	0	0	1	0	0	1	
M269	M00-M99	Diseases of the musculoskeletal system and	NA	Dentofacial anomaly, unspecified	0	0	0	1	0	0	1	
M410	M00-M99	Diseases of the musculoskeletal system and	NA	Infantile idiopathic scoliosis	0	0	1	0	0	1	0	
M4100	M00-M99	Diseases of the musculoskeletal system and	NA	Infantile idiopathic scoliosis, site unspecified	0	0	1	0	0	1	0	
M4102	M00-M99	Diseases of the musculoskeletal system and	NA	Infantile idiopathic scoliosis, cervical region	0	0	1	0	0	1	0	
M4103	M00-M99	Diseases of the musculoskeletal system and	NA	Infantile idiopathic scoliosis, cervicothoracic region	0	0	1	0	0	1	0	
M4104	M00-M99	Diseases of the musculoskeletal system and	NA	Infantile idiopathic scoliosis, thoracic region	0	0	1	0	0	1	0	
M4105	M00-M99	Diseases of the musculoskeletal system and	NA	Infantile idiopathic scoliosis, thoracolumbar region	0	0	1	0	0	1	0	
M4106	M00-M99	Diseases of the musculoskeletal system and	NA	Infantile idiopathic scoliosis, lumbar region	0	0	1	0	0	1	0	
M4107	M00-M99	Diseases of the musculoskeletal system and	NA	Infantile idiopathic scoliosis, lumbosacral region	0	0	1	0	0	1	0	
M4108	M00-M99	Diseases of the musculoskeletal system and	NA	Infantile idiopathic scoliosis, sacral and sacrococcygeal region	0	0	1	0	0	1	0	
M4327	M00-M99	Diseases of the musculoskeletal system and	NA	Fusion of spine, lumbosacral region	0	0	0	0	0	0	1	
M434	M00-M99	Diseases of the musculoskeletal system and	NA	Other recurrent atlantoaxial dislocation	0	0	0	0	0	0	1	
M435X9	M00-M99	Diseases of the musculoskeletal system and	NA	Other recurrent vertebral dislocation, site unspecified	0	0	0	0	0	0	1	
M436	M00-M99	Diseases of the musculoskeletal system and	NA	Torticollis	0	0	0	1	0	0	0	
M6282	M00-M99	Diseases of the musculoskeletal system and	NA	Rhabdomyolysis	0	0	0	0	0	0	1	
M89129	M00-M99	Diseases of the musculoskeletal system and	NA	Physeal arrest, humerus, unspecified	0	0	0	0	0	0	1	
M9420	M00-M99	Diseases of the musculoskeletal system and	NA	Chondromalacia, unspecified site	0	0	0	0	0	0	1	
N07	N00-N99	Disease of the genitourinary system	NA	Hereditary nephropathy, not elsewhere classified	0	1	0	0	1	0	0	
N070	N00-N99	Disease of the genitourinary system	NA	Hereditary nephropathy, not elsewhere classified with minor glomerular abnormality	0	1	0	0	1	0	0	
N071	N00-N99	Disease of the genitourinary system	NA	Hereditary nephropathy, not elsewhere classified with focal and segmental glomerula	0	1	0	0	1	0	0	
N072	N00-N99	Disease of the genitourinary system	NA	Hereditary nephropathy, not elsewhere classified with diffuse membranous glomerulo	0	1	0	0	1	0	0	
N073	N00-N99	Disease of the genitourinary system	NA	Hereditary nephropathy, not elsewhere classified with diffuse mesangial proliferative	0	1	0	0	1	0	0	
N074	N00-N99	Disease of the genitourinary system	NA	Hereditary nephropathy, not elsewhere classified with diffuse endocapillary proliferati	0	1	0	0	1	0	0	
N075	N00-N99	Disease of the genitourinary system	NA	Hereditary nephropathy, not elsewhere classified with diffuse mesangiocapillary glom	0	1	0	0	1	0	0	
N076	N00-N99	Disease of the genitourinary system	NA	Hereditary nephropathy, not elsewhere classified with dense deposit disease	0	1	0	0	1	0	0	
N077	N00-N99	Disease of the genitourinary system	NA	Hereditary nephropathy, not elsewhere classified with diffuse crescentic glomerulone	0	1	0	0	1	0	0	
N078	N00-N99	Disease of the genitourinary system	NA	Hereditary nephropathy, not elsewhere classified with other morphologic lesions	0	1	0	0	1	0	0	
N079	N00-N99	Disease of the genitourinary system	NA	Hereditary nephropathy, not elsewhere classified with unspecified morphologic lesions	0	1	0	0	1	0	0	
N08	N00-N99	Disease of the genitourinary system	NA	Glomerular disorders in diseases classified elsewhere	0	0	0	0	0	0	1	
N130	N00-N99	Disease of the genitourinary system	NA	HYDRONEPHR URETEROPELVIC JUNCT OBST	0	0	0	1	0	0	1	
N131	N00-N99	Disease of the genitourinary system	NA	HYDRONEPHROSIS W/URETRL STRICT NEC	0	0	0	1	0	0	1	
N132	N00-N99	Disease of the genitourinary system	NA	HYDRONPHROS RENL&URETRL CALCUL OBST	0	0	0	1	0	0	1	
N1330	N00-N99	Disease of the genitourinary system	NA	UNSPECIFIED HYDRONEPHROSIS	0	0	0	1	0	0	1	
N1339	N00-N99	Disease of the genitourinary system	NA	OTHER HYDRONEPHROSIS	0	0	0	1	0	0	1	
N134	N00-N99	Disease of the genitourinary system	NA	HYDROURETER	0	0	0	1	0	0	1	
N1370	N00-N99	Disease of the genitourinary system	NA	VESICOURETERAL-REFLUX UNSPECIFIED	0	0	0	1	0	0	1	
N1371	N00-N99	Disease of the genitourinary system	NA	VUR W/O REFLUX NEPHROPATHY	0	0	0	1	0	0	1	
N13721	N00-N99	Disease of the genitourinary system	NA	VUR W/RN W/O HYDROURETER UNI	0	0	0	1	0	0	1	
N13722	N00-N99	Disease of the genitourinary system	NA	VUR W/RN W/O HYDROURETER BIL	0	0	0	1	0	0	1	
N13729	N00-N99	Disease of the genitourinary system	NA	VUR W/RN W/O HYDROURETER UNS	0	0	0	0	0	0	1	
N13731	N00-N99	Disease of the genitourinary system	NA	VUR W/RN W/HYDROURETER UNI	0	0	0	1	0	0	1	
N13732	N00-N99	Disease of the genitourinary system	NA	VUR W/RN W/HYDROURETER BIL	0	0	0	1	0	0	1	
N13739	N00-N99	Disease of the genitourinary system	NA	VUR W/RN W/HYDROURETER UNS	0	0	0	1	0	0	1	
N138	N00-N99	Disease of the genitourinary system	NA	OTHER OBSTRUCTIVE & REFLUX UROPATHY	0	0	0	1	0	0	1	
N139	N00-N99	Disease of the genitourinary system	NA	OBSTRUCTIVE & REFLUX UROPATHY UNS	0	0	0	1	0	0	1	
N2881	N00-N99	Disease of the genitourinary system	NA	HYPERTROPHY OF KIDNEY	0	0	0	1	0	0	1	
N2882	N00-N99	Disease of the genitourinary system	NA	MEGALOURETER	0	0	0	1	0	0	1	
N46	N00-N99	Disease of the genitourinary system	NA	Male infertility	0	0	0	0	0	0	0	
N460	N00-N99	Disease of the genitourinary system	NA	Azoospermia	0	0	0	0	0	0	0	
N4601	N00-N99	Disease of the genitourinary system	NA	Organic azoospermia	0	0	0	0	0	0	0	
N509	N00-N99	Disease of the genitourinary system	NA	DISORDER MALE GENITAL ORGANS UNS	0	0	1	0	0	0	1	
N6482	N00-N99	Disease of the genitourinary system	NA	Hypoplasia of breast	0	0	0	1	0	0	1	
N824	N00-N99	Disease of the genitourinary system	NA	Other female intestinal-genital tract fistulae	0	0	0	1	0	0	1	
O285	O00-O9A	Pregnancy, childbirth, and the puerperium	NA	Abnormal chromosomal and genetic finding on antenatal screening of mother	1	1	0	0	1	0	0	
O352	O00-O9A	Pregnancy, childbirth, and the puerperium	NA	Maternal care for (suspected) hereditary disease in fetus	0	0	0	1	0	0	1	
O352XX0	O00-O9A	Pregnancy, childbirth, and the puerperium	NA	Maternal care for (suspected) hereditary disease in fetus, not applicable or unspecified	0	1	0	0	1	0	0	
O352XX1	O00-O9A	Pregnancy, childbirth, and the puerperium	NA	Maternal care for (suspected) hereditary disease in fetus, fetus 1	0	1	0	0	1	0	0	
O352XX2	O00-O9A	Pregnancy, childbirth, and the puerperium	NA	Maternal care for (suspected) hereditary disease in fetus, fetus 2	0	1	0	0	1	0	0	
O352XX3	O00-O9A	Pregnancy, childbirth, and the puerperium	NA	Maternal care for (suspected) hereditary disease in fetus, fetus 3	0	1	0	0	1	0	0	
O352XX4	O00-O9A	Pregnancy, childbirth, and the puerperium	NA	Maternal care for (suspected) hereditary disease in fetus, fetus 4	0	1	0	0	1	0	0	
O352XX5	O00-O9A	Pregnancy, childbirth, and the puerperium	NA	Maternal care for (suspected) hereditary disease in fetus, fetus 5	0	1	0	0	1	0	0	
O352XX9	O00-O9A	Pregnancy, childbirth, and the puerperium	NA	Maternal care for (suspected) hereditary disease in fetus, other fetus	0	1	0	0	1	0	0	
O3660X0	O00-O9A	Pregnancy, childbirth, and the puerperium	NA	Maternal care for excessive fetal growth, unspecified trimester, not applicable or unsp	0	0	0	1	0	0	0	
O368190	O00-O9A	Pregnancy, childbirth, and the puerperium	NA	Decreased fetal movements, unspecified trimester, not applicable or unspecified	0	0	0	1	0	0	0	
O40	O00-O9A	Pregnancy, childbirth, and the puerperium	NA	Polyhydramnios	0	0	0	1	0	0	1	
O401XX0	O00-O9A	Pregnancy, childbirth, and the puerperium	NA	POLYHYDRAMNIOS FIRST TRI NA/UNS	0	0	0	1	0	0	1	
O401XX1	O00-O9A	Pregnancy, childbirth, and the puerperium	NA	POLYHYDRAMNIOS FIRST TRI FETUS 1	0	0	0	1	0	0	1	
O402XX0	O00-O9A	Pregnancy, childbirth, and the puerperium	NA	POLYHYDRAMNIOS SECOND TRI NA/UNS	0	0	0	1	0	0	1	
O402XX1	O00-O9A	Pregnancy, childbirth, and the puerperium	NA	POLYHYDRAMNIOS SECOND TRI FETUS 1	0	0	0	1	0	0	1	
O403XX0	O00-O9A	Pregnancy, childbirth, and the puerperium	NA	POLYHYDRAMNIOS THIRD TRI NA/UNS	0	0	0	1	0	0	1	
O403XX1	O00-O9A	Pregnancy, childbirth, and the puerperium	NA	POLYHYDRAMNIOS THIRD TRI FETUS 1	0	0	0	1	0	0	1	
O403XX2	O00-O9A	Pregnancy, childbirth, and the puerperium	NA	POLYHYDRAMNIOS THIRD TRI FETUS 2	0	0	0	1	0	0	1	
O403XX9	O00-O9A	Pregnancy, childbirth, and the puerperium	NA	POLYHYDRAMNIOS THIRD TRI OTH FETUS	0	0	0	1	0	0	1	
O409XX0	O00-O9A	Pregnancy, childbirth, and the puerperium	NA	Polyhydramnios, unspecified trimester, not applicable or unspecified	0	0	0	1	0	0	0	
O409XX1	O00-O9A	Pregnancy, childbirth, and the puerperium	NA	POLYHYDRAMNIOS UNS TRI FETUS 1	0	0	0	1	0	0	1	
O409XX9	O00-O9A	Pregnancy, childbirth, and the puerperium	NA	POLYHYDRAMNIOS UNS TRI OTH FETUS	0	0	0	1	0	0	1	
O410	O00-O9A	Pregnancy, childbirth, and the puerperium	NA	Oligohydramnios	0	0	0	1	0	0	1	
O4100X0	O00-O9A	Pregnancy, childbirth, and the puerperium	NA	Oligohydramnios, unspecified trimester, not applicable or unspecified	0	0	0	1	0	0	0	
O4100X1	O00-O9A	Pregnancy, childbirth, and the puerperium	NA	OLIGOHYDRAMNIOS UNS TRI FETUS 1	0	0	0	1	0	0	1	
O4100X9	O00-O9A	Pregnancy, childbirth, and the puerperium	NA	OLIGOHYDRAMNIOS UNS TRI OTH FETUS	0	0	0	1	0	0	1	
O4101X0	O00-O9A	Pregnancy, childbirth, and the puerperium	NA	OLIGOHYDRAMNIOS FIRST TRI NA/UNS	0	0	0	1	0	0	1	
O4101X3	O00-O9A	Pregnancy, childbirth, and the puerperium	NA	OLIGOHYDRAMNIOS FIRST TRI FETUS 3	0	0	0	1	0	0	1	
O4102X0	O00-O9A	Pregnancy, childbirth, and the puerperium	NA	OLIGOHYDRAMNIOS SECOND TRI NA/UNS	0	0	0	1	0	0	1	
O4102X1	O00-O9A	Pregnancy, childbirth, and the puerperium	NA	OLIGOHYDRAMNIOS SECOND TRI FETUS 1	0	0	0	1	0	0	1	
O4102X2	O00-O9A	Pregnancy, childbirth, and the puerperium	NA	OLIGOHYDRAMNIOS SECOND TRI FETUS 2	0	0	0	1	0	0	1	
O4103X0	O00-O9A	Pregnancy, childbirth, and the puerperium	NA	OLIGOHYDRAMNIOS THIRD TRI NA/UNS	0	0	0	1	0	0	1	
O4103X1	O00-O9A	Pregnancy, childbirth, and the puerperium	NA	OLIGOHYDRAMNIOS THIRD TRI FETUS 1	0	0	0	1	0	0	1	
O4103X9	O00-O9A	Pregnancy, childbirth, and the puerperium	NA	OLIGOHYDRAMNIOS THIRD TRI OTH FETUS	0	0	0	1	0	0	1	
P012	P00-P96	Certain conditions originating in the perinata	NA	Newborn affected by oligohydramnios	0	0	0	1	0	0	1	
P013	P00-P96	Certain conditions originating in the perinata	NA	Newborn affected by polyhydramnios	0	0	0	1	0	0	1	
P0381	P00-P96	Certain conditions originating in the perinata	NA	Newborn affected by abnormality infetal (intrauterine) heart rate or rhythm	0	0	0	1	0	0	1	
P03810	P00-P96	Certain conditions originating in the perinata	NA	Newborn affected by abnormality in fetal (intrauterine) heart rate or rhythm before th	0	0	0	1	0	0	1	
P03811	P00-P96	Certain conditions originating in the perinata	NA	Newborn affected by abnormality in fetal (intrauterine) heart rate or rhythm during lab	0	0	0	1	0	0	1	
P03819	P00-P96	Certain conditions originating in the perinata	NA	Newborn affected by abnormality in fetal (intrauterine) heart rate or rhythm unspecifi	0	0	0	1	0	0	1	
P09	P00-P96	Certain conditions originating in the perinata	NA	Abnormal findings on neonatal screening	0	0	1	0	0	1	0	
P290	P00-P96	Certain conditions originating in the perinata	NA	Neonatal cardiac failure	0	0	0	1	0	0	1	
P291	P00-P96	Certain conditions originating in the perinata	NA	Neonatal cardiac dysrhythmia	0	0	0	1	0	0	1	
P2911	P00-P96	Certain conditions originating in the perinata	NA	Neonatal tachycardia	0	0	0	1	0	0	1	
P2912	P00-P96	Certain conditions originating in the perinata	NA	Neonatal bradycardia	0	0	0	1	0	0	1	
P588	P00-P96	Certain conditions originating in the perinata	NA	Neonatal jaundice due to other specified excessive hemolysis	0	0	0	1	0	0	0	
P592	P00-P96	Certain conditions originating in the perinata	NA	Neonatal jaundice from other and unspecified hepatocellular damage	0	0	0	1	0	0	1	
P5920	P00-P96	Certain conditions originating in the perinata	NA	Neonatal jaundice from unspecifiedhepatocellular damage	0	0	0	1	0	0	1	
P614	P00-P96	Certain conditions originating in the perinata	NA	Other congenital anemias, not elsewhere classified	0	0	1	0	0	1	0	
P702	P00-P96	Certain conditions originating in the perinata	NA	Neonatal diabetes mellitus	0	0	1	0	0	1	0	
P7881	P00-P96	Certain conditions originating in the perinata	NA	Congenital cirrhosis (of liver)	0	0	1	0	0	1	0	
P832	P00-P96	Certain conditions originating in the perinata	NA	Hydrops fetalis not due to hemolytic disease	0	0	1	0	0	1	0	
P8330	P00-P96	Certain conditions originating in the perinata	NA	Unspecified edema specific to newborn	0	0	0	1	0	0	1	
P8339	P00-P96	Certain conditions originating in the perinata	NA	Other edema specific to newborn	0	0	0	1	0	0	1	
P90	P00-P96	Certain conditions originating in the perinata	NA	Convulsions of newborn	0	0	0	1	0	0	1	
P914	P00-P96	Certain conditions originating in the perinata	NA	Neonatal cerebral depression	0	0	0	1	0	0	0	
P915	P00-P96	Certain conditions originating in the perinata	NA	Neonatal coma	0	0	1	0	0	1	0	
P94	P00-P96	Certain conditions originating in the perinata	NA	Disorders of muscle tone of newborn	0	0	0	1	0	0	1	
P941	P00-P96	Certain conditions originating in the perinata	NA	Congenital hypertonia	0	0	0	1	0	0	1	
P942	P00-P96	Certain conditions originating in the perinata	NA	Congenital hypotonia	0	0	0	1	0	0	1	
P948	P00-P96	Certain conditions originating in the perinata	NA	Other disorders of muscle tone ofnewborn	0	0	0	1	0	0	1	
P949	P00-P96	Certain conditions originating in the perinata	NA	Disorder of muscle tone of newborn, unspecified	0	0	0	1	0	0	1	
P960	P00-P96	Certain conditions originating in the perinata	NA	Congenital renal failure	0	0	1	0	0	1	0	
P963	P00-P96	Certain conditions originating in the perinata	NA	Wide cranial sutures of newborn	0	0	0	1	0	0	1	
Q00	Q00-Q99	Congenital malformations, deformations and	NervousSystem	Anencephaly and similar malformations	0	0	0	1	0	0	1	1
Q000	Q00-Q99	Congenital malformations, deformations and	NervousSystem	Anencephaly	0	0	0	1	0	0	1	1
Q001	Q00-Q99	Congenital malformations, deformations and	NervousSystem	Craniorachischisis	0	0	0	1	0	0	1	1
Q002	Q00-Q99	Congenital malformations, deformations and	NervousSystem	Iniencephaly	0	0	0	1	0	0	1	1

Q01	Q00-Q99	Congenital malformations, deformations and	NervousSystem	Encephalocele	0	0	0	1	0	0	1	1
Q010	Q00-Q99	Congenital malformations, deformations and	NervousSystem	Frontal encephalocele	0	0	0	1	0	0	1	1
Q011	Q00-Q99	Congenital malformations, deformations and	NervousSystem	Nasofrontal encephalocele	0	0	0	1	0	0	1	1
Q012	Q00-Q99	Congenital malformations, deformations and	NervousSystem	Occipital encephalocele	0	0	0	1	0	0	1	1
Q018	Q00-Q99	Congenital malformations, deformations and	NervousSystem	Encephalocele of other sites	0	0	0	1	0	0	1	1
Q019	Q00-Q99	Congenital malformations, deformations and	NervousSystem	Encephalocele, unspecified	0	0	0	1	0	0	1	1
Q02	Q00-Q99	Congenital malformations, deformations and	NervousSystem	Microcephaly	0	0	0	1	0	0	1	1
Q03	Q00-Q99	Congenital malformations, deformations and	NervousSystem	Congenital hydrocephalus	0	0	0	1	0	0	1	1
Q030	Q00-Q99	Congenital malformations, deformations and	NervousSystem	Malformations of aqueduct of Sylvius	0	0	1	0	0	1	0	1
Q031	Q00-Q99	Congenital malformations, deformations and	NervousSystem	Atresia of foramina of Magendie and Luschka	0	0	0	1	0	0	1	1
Q038	Q00-Q99	Congenital malformations, deformations and	NervousSystem	Other congenital hydrocephalus	0	0	0	1	0	0	1	1
Q039	Q00-Q99	Congenital malformations, deformations and	NervousSystem	Congenital hydrocephalus, unspecified	0	0	0	1	0	0	1	1
Q04	Q00-Q99	Congenital malformations, deformations and	NervousSystem	Other congenital malformations ofbrain	0	0	0	1	0	0	1	1
Q040	Q00-Q99	Congenital malformations, deformations and	NervousSystem	Congenital malformations of corpuscallosum	0	0	0	1	0	0	1	1
Q041	Q00-Q99	Congenital malformations, deformations and	NervousSystem	Arhinencephaly	0	0	0	1	0	0	1	1
Q042	Q00-Q99	Congenital malformations, deformations and	NervousSystem	Holoprosencephaly	0	0	0	1	0	0	1	1
Q043	Q00-Q99	Congenital malformations, deformations and	NervousSystem	Other reduction deformities of brain	0	0	0	1	0	0	1	1
Q044	Q00-Q99	Congenital malformations, deformations and	NervousSystem	Septo-optic dysplasia of brain	0	0	0	1	0	0	1	1
Q045	Q00-Q99	Congenital malformations, deformations and	NervousSystem	Megalencephaly	0	0	0	1	0	0	1	1
Q046	Q00-Q99	Congenital malformations, deformations and	NervousSystem	Congenital cerebral cysts	0	0	0	1	0	0	1	1
Q048	Q00-Q99	Congenital malformations, deformations and	NervousSystem	Other specified congenital malformations of brain	0	0	0	1	0	0	1	1
Q049	Q00-Q99	Congenital malformations, deformations and	NervousSystem	Congenital malformation of brain,unspecified	0	0	0	1	0	0	1	1
Q05	Q00-Q99	Congenital malformations, deformations and	NervousSystem	Spina bifida	0	0	0	1	0	0	1	1
Q050	Q00-Q99	Congenital malformations, deformations and	NervousSystem	Cervical spina bifida with hydrocephalus	0	0	0	1	0	0	1	1
Q051	Q00-Q99	Congenital malformations, deformations and	NervousSystem	Thoracic spina bifida with hydrocephalus	0	0	0	1	0	0	1	1
Q052	Q00-Q99	Congenital malformations, deformations and	NervousSystem	Lumbar spina bifida with hydrocephalus	0	0	0	1	0	0	1	1
Q053	Q00-Q99	Congenital malformations, deformations and	NervousSystem	Sacral spina bifida with hydrocephalus	0	0	0	1	0	0	1	1
Q054	Q00-Q99	Congenital malformations, deformations and	NervousSystem	Unspecified spina bifida with hydrocephalus	0	0	0	1	0	0	1	1
Q055	Q00-Q99	Congenital malformations, deformations and	NervousSystem	Cervical spina bifida without hydrocephalus	0	0	0	1	0	0	1	1
Q056	Q00-Q99	Congenital malformations, deformations and	NervousSystem	Thoracic spina bifida without hydrocephalus	0	0	0	1	0	0	1	1
Q057	Q00-Q99	Congenital malformations, deformations and	NervousSystem	Lumbar spina bifida without hydrocephalus	0	0	0	1	0	0	1	1
Q058	Q00-Q99	Congenital malformations, deformations and	NervousSystem	Sacral spina bifida without hydrocephalus	0	0	0	1	0	0	1	1
Q059	Q00-Q99	Congenital malformations, deformations and	NervousSystem	Spina bifida, unspecified	0	0	0	1	0	0	1	1
Q06	Q00-Q99	Congenital malformations, deformations and	NervousSystem	Other congenital malformations ofspinal cord	0	0	0	1	0	0	1	1
Q060	Q00-Q99	Congenital malformations, deformations and	NervousSystem	Amyelia	0	0	0	1	0	0	1	1
Q061	Q00-Q99	Congenital malformations, deformations and	NervousSystem	Hypoplasia and dysplasia of spinalcord	0	0	0	1	0	0	1	1
Q062	Q00-Q99	Congenital malformations, deformations and	NervousSystem	Diastematomyelia	0	0	0	1	0	0	1	1
Q063	Q00-Q99	Congenital malformations, deformations and	NervousSystem	Other congenital cauda equina malformations	0	0	0	1	0	0	1	1
Q064	Q00-Q99	Congenital malformations, deformations and	NervousSystem	Hydromyelia	0	0	0	1	0	0	1	1
Q068	Q00-Q99	Congenital malformations, deformations and	NervousSystem	Other specified congenital malformations of spinal cord	0	0	0	1	0	0	1	1
Q069	Q00-Q99	Congenital malformations, deformations and	NervousSystem	Congenital malformation of spinalcord, unspecified	0	0	0	1	0	0	1	1
Q07	Q00-Q99	Congenital malformations, deformations and	NervousSystem	Other congenital malformations ofnervous system	0	0	0	1	0	0	1	1
Q070	Q00-Q99	Congenital malformations, deformations and	NervousSystem	Arnold-Chiari syndrome	0	0	0	1	0	0	1	1
Q0700	Q00-Q99	Congenital malformations, deformations and	NervousSystem	Arnold-Chiari syndrome without spina bifida or hydrocephalus	0	0	0	1	0	0	1	1
Q0701	Q00-Q99	Congenital malformations, deformations and	NervousSystem	Arnold-Chiari syndrome with spinabifida	0	0	0	1	0	0	1	1
Q0702	Q00-Q99	Congenital malformations, deformations and	NervousSystem	Arnold-Chiari syndrome with hydrocephalus	0	0	0	1	0	0	1	1
Q0703	Q00-Q99	Congenital malformations, deformations and	NervousSystem	Arnold-Chiari syndrome with spinabifida and hydrocephalus	0	0	0	1	0	0	1	1
Q078	Q00-Q99	Congenital malformations, deformations and	NervousSystem	Other specified congenital malformations of nervous system	0	0	0	1	0	0	1	1
Q079	Q00-Q99	Congenital malformations, deformations and	NervousSystem	Congenital malformation of nervoussystem, unspecified	0	0	0	1	0	0	1	1
Q10	Q00-Q99	Congenital malformations, deformations and	Eye	Congenital malformations of eyelid, lacrimal apparatus and orbit	0	0	0	1	0	0	1	1
Q100	Q00-Q99	Congenital malformations, deformations and	Eye	Congenital ptosis	0	0	0	1	0	0	1	1
Q101	Q00-Q99	Congenital malformations, deformations and	Eye	Congenital ectropion	0	0	0	1	0	0	1	1
Q102	Q00-Q99	Congenital malformations, deformations and	Eye	Congenital entropion	0	0	0	1	0	0	1	1
Q104	Q00-Q99	Congenital malformations, deformations and	Eye	Absence and agenesis of lacrimal apparatus	0	0	0	1	0	0	1	1
Q106	Q00-Q99	Congenital malformations, deformations and	Eye	Other congenital malformations oflacrimal apparatus	0	0	0	1	0	0	1	1
Q107	Q00-Q99	Congenital malformations, deformations and	Eye	Congenital malformation of orbit	0	0	0	1	0	0	1	1
Q11	Q00-Q99	Congenital malformations, deformations and	Eye	Anophthalmos, microphthalmos and macrophthalmos	0	0	1	0	0	1	0	1
Q110	Q00-Q99	Congenital malformations, deformations and	Eye	Cystic eyeball	0	0	0	1	0	0	1	1
Q111	Q00-Q99	Congenital malformations, deformations and	Eye	Other anophthalmos	0	0	1	0	0	1	0	1
Q112	Q00-Q99	Congenital malformations, deformations and	Eye	Microphthalmos	0	0	1	0	0	1	0	1
Q113	Q00-Q99	Congenital malformations, deformations and	Eye	Macrophthalmos	0	0	0	1	0	0	1	1
Q12	Q00-Q99	Congenital malformations, deformations and	Eye	Congenital lens malformations	0	0	0	1	0	0	1	1
Q120	Q00-Q99	Congenital malformations, deformations and	Eye	Congenital cataract	0	0	1	0	0	1	0	1
Q121	Q00-Q99	Congenital malformations, deformations and	Eye	Congenital displaced lens	0	0	1	0	0	1	0	1
Q122	Q00-Q99	Congenital malformations, deformations and	Eye	Coloboma of lens	0	0	1	0	0	1	0	1
Q123	Q00-Q99	Congenital malformations, deformations and	Eye	Congenital aphakia	0	0	1	0	0	1	0	1
Q124	Q00-Q99	Congenital malformations, deformations and	Eye	Spherophakia	0	0	0	1	0	0	1	1
Q128	Q00-Q99	Congenital malformations, deformations and	Eye	Other congenital lens malformations	0	0	0	1	0	0	1	1
Q129	Q00-Q99	Congenital malformations, deformations and	Eye	Congenital lens malformation, unspecified	0	0	0	1	0	0	1	1
Q13	Q00-Q99	Congenital malformations, deformations and	Eye	Congenital malformations of anterior segment of eye	0	0	0	1	0	0	1	1
Q130	Q00-Q99	Congenital malformations, deformations and	Eye	Coloboma of iris	0	0	1	0	0	1	0	1
Q131	Q00-Q99	Congenital malformations, deformations and	Eye	Absence of iris	0	0	1	0	0	1	0	1
Q132	Q00-Q99	Congenital malformations, deformations and	Eye	Other congenital malformations ofiris	0	0	0	1	0	0	1	1
Q133	Q00-Q99	Congenital malformations, deformations and	Eye	Congenital corneal opacity	0	0	0	1	0	0	1	1
Q134	Q00-Q99	Congenital malformations, deformations and	Eye	Other congenital corneal malformations	0	0	0	1	0	0	1	1
Q135	Q00-Q99	Congenital malformations, deformations and	Eye	Blue sclera	0	0	0	1	0	0	1	1
Q138	Q00-Q99	Congenital malformations, deformations and	Eye	Other congenital malformations ofanterior segment of eye	0	0	0	1	0	0	1	1
Q1381	Q00-Q99	Congenital malformations, deformations and	Eye	Rieger's anomaly	0	0	1	0	0	1	0	1
Q1389	Q00-Q99	Congenital malformations, deformations and	Eye	Other congenital malformations ofanterior segment of eye	0	0	0	1	0	0	1	1
Q139	Q00-Q99	Congenital malformations, deformations and	Eye	Congenital malformation of anterior segment of eye, unspecified	0	0	0	1	0	0	1	1
Q14	Q00-Q99	Congenital malformations, deformations and	Eye	Congenital malformations of posterior segment of eye	0	0	0	1	0	0	1	1
Q140	Q00-Q99	Congenital malformations, deformations and	Eye	Congenital malformation of vitreous humor	0	0	0	1	0	0	1	1
Q141	Q00-Q99	Congenital malformations, deformations and	Eye	Congenital malformation of retina	0	0	0	1	0	0	1	1
Q142	Q00-Q99	Congenital malformations, deformations and	Eye	Congenital malformation of optic disc	0	0	0	1	0	0	1	1
Q143	Q00-Q99	Congenital malformations, deformations and	Eye	Congenital malformation of choroid	0	0	0	1	0	0	1	1
Q148	Q00-Q99	Congenital malformations, deformations and	Eye	Other congenital malformations ofposterior segment of eye	0	0	0	1	0	0	1	1
Q149	Q00-Q99	Congenital malformations, deformations and	Eye	Congenital malformation of posterior segment of eye, unspecified	0	0	0	1	0	0	1	1
Q15	Q00-Q99	Congenital malformations, deformations and	Eye	Other congenital malformations ofeye	0	0	0	1	0	0	1	1
Q150	Q00-Q99	Congenital malformations, deformations and	Eye	Congenital glaucoma	0	0	1	0	0	1	0	1
Q158	Q00-Q99	Congenital malformations, deformations and	Eye	Other specified congenital malformations of eye	0	0	0	1	0	0	1	1
Q159	Q00-Q99	Congenital malformations, deformations and	Eye	Congenital malformation of eye, unspecified	0	0	0	1	0	0	1	1
Q16	Q00-Q99	Congenital malformations, deformations and	Ear	Congenital malformations of ear causing impairment of hearing	0	0	0	1	0	0	1	1
Q160	Q00-Q99	Congenital malformations, deformations and	Ear	Congenital absence of (ear) auricle	0	0	0	1	0	0	1	1
Q161	Q00-Q99	Congenital malformations, deformations and	Ear	Congenital absence, atresia and stricture of auditory canal (external)	0	0	0	1	0	0	1	1
Q162	Q00-Q99	Congenital malformations, deformations and	Ear	Absence of eustachian tube	0	0	0	1	0	0	1	1
Q163	Q00-Q99	Congenital malformations, deformations and	Ear	Congenital malformation of ear ossicles	0	0	0	1	0	0	1	1
Q164	Q00-Q99	Congenital malformations, deformations and	Ear	Other congenital malformations ofmiddle ear	0	0	0	1	0	0	1	1
Q165	Q00-Q99	Congenital malformations, deformations and	Ear	Congenital malformation of inner ear	0	0	1	0	0	1	0	1
Q169	Q00-Q99	Congenital malformations, deformations and	Ear	Congenital malformation of ear causing impairment of hearing, unspecified	0	0	0	1	0	0	1	1
Q17	Q00-Q99	Congenital malformations, deformations and	Ear	Other congenital malformations ofear	0	0	0	1	0	0	1	1
Q170	Q00-Q99	Congenital malformations, deformations and	Ear	Accessory auricle	0	0	0	1	0	0	1	1
Q171	Q00-Q99	Congenital malformations, deformations and	Ear	Macrotia	0	0	0	1	0	0	1	1
Q172	Q00-Q99	Congenital malformations, deformations and	Ear	Microtia	0	0	0	1	0	0	1	1
Q173	Q00-Q99	Congenital malformations, deformations and	Ear	Other misshapen ear	0	0	0	1	0	0	1	1
Q174	Q00-Q99	Congenital malformations, deformations and	Ear	Misplaced ear	0	0	0	1	0	0	1	1
Q175	Q00-Q99	Congenital malformations, deformations and	Ear	Prominent ear	0	0	0	1	0	0	1	1
Q178	Q00-Q99	Congenital malformations, deformations and	Ear	Other specified congenital malformations of ear	0	0	0	1	0	0	1	1
Q179	Q00-Q99	Congenital malformations, deformations and	Ear	Congenital malformation of ear, unspecified	0	0	0	1	0	0	1	1
Q18	Q00-Q99	Congenital malformations, deformations and	FaceNeck	Other congenital malformations offace and neck	0	0	0	1	0	0	1	1
Q180	Q00-Q99	Congenital malformations, deformations and	FaceNeck	Sinus, fistula and cyst of branchial cleft	0	0	0	1	0	0	1	1
Q181	Q00-Q99	Congenital malformations, deformations and	FaceNeck	Preauricular sinus and cyst	0	0	0	1	0	0	1	1
Q182	Q00-Q99	Congenital malformations, deformations and	FaceNeck	Other branchial cleft malformations	0	0	0	1	0	0	1	1
Q183	Q00-Q99	Congenital malformations, deformations and	FaceNeck	Webbing of neck	0	0	1	0	0	1	0	1
Q184	Q00-Q99	Congenital malformations, deformations and	FaceNeck	Macrostomia	0	0	0	1	0	0	1	1
Q185	Q00-Q99	Congenital malformations, deformations and	FaceNeck	Microstomia	0	0	0	1	0	0	1	1
Q186	Q00-Q99	Congenital malformations, deformations and	FaceNeck	Macrocheilia	0	0	0	1	0	0	1	1
Q187	Q00-Q99	Congenital malformations, deformations and	FaceNeck	Microcheilia	0	0	0	1	0	0	1	1
Q188	Q00-Q99	Congenital malformations, deformations and	FaceNeck	Other specified congenital malformations of face and neck	0	0	0	1	0	0	1	1
Q189	Q00-Q99	Congenital malformations, deformations and	FaceNeck	Congenital malformation of face and neck, unspecified	0	0	0	1	0	0	1	1
Q20	Q00-Q99	Congenital malformations, deformations and	Circulatory	Congenital malformations of cardiac chambers and connections	0	0	0	1	0	0	1	1
Q200	Q00-Q99	Congenital malformations, deformations and	Circulatory	Common arterial trunk	0	0	0	1	0	0	1	1
Q201	Q00-Q99	Congenital malformations, deformations and	Circulatory	Double outlet right ventricle	0	0	0	1	0	0	1	1
Q202	Q00-Q99	Congenital malformations, deformations and	Circulatory	Double outlet left ventricle	0	0	0	1	0	0	1	1
Q203	Q00-Q99	Congenital malformations, deformations and	Circulatory	Discordant ventriculoarterial connection	0	0	0	1	0	0	1	1
Q204	Q00-Q99	Congenital malformations, deformations and	Circulatory	Double inlet ventricle	0	0	0	1	0	0	1	1
Q205	Q00-Q99	Congenital malformations, deformations and	Circulatory	Discordant atrioventricular connection	0	0	0	1	0	0	1	1
Q206	Q00-Q99	Congenital malformations, deformations and	Circulatory	Isomerism of atrial appendages	0	0	0	1	0	0	1	1
Q208	Q00-Q99	Congenital malformations, deformations and	Circulatory	Other congenital malformations ofcardiac chambers and connections	0	0	0	1	0	0	1	1
Q209	Q00-Q99	Congenital malformations, deformations and	Circulatory	Congenital malformation of cardiacchambers and connections, unspecified	0	0	0	1	0	0	1	1
Q21	Q00-Q99	Congenital malformations, deformations and	Circulatory	Congenital malformations of cardiac septa	0	0	0	1	0	0	1	1
Q212	Q00-Q99	Congenital malformations, deformations and	Circulatory	Atrioventricular septal defect	0	0	0	1	0	0	1	1
Q213	Q00-Q99	Congenital malformations, deformations and	Circulatory	Tetralogy of Fallot	0	0	0	1	0	0	1	1
Q214	Q00-Q99	Congenital malformations, deformations and	Circulatory	Aortopulmonary septal defect	0	0	0	1	0	0	1	1
Q218	Q00-Q99	Congenital malformations, deformations and	Circulatory	Other congenital malformations ofcardiac septa	0	0	0	1	0	0	1	1
Q219	Q00-Q99	Congenital malformations, deformations and	Circulatory	Congenital malformation of cardiacseptum, unspecified	0	0	0	1	0	0	1	1
Q22	Q00-Q99	Congenital malformations, deformations and	Circulatory	Congenital malformations of pulmonary and tricuspid valves	0	0	0	1	0	0	1	1
Q220	Q00-Q99	Congenital malformations, deformations and	Circulatory	Pulmonary valve atresia	0	0	0	1	0	0	1	1
Q221	Q00-Q99	Congenital malformations, deformations and	Circulatory	Congenital pulmonary valve stenosis	0	0	0	1	0	0	1	1
Q222	Q00-Q99	Congenital malformations, deformations and	Circulatory	Congenital pulmonary valve insufficiency	0	0	0	1	0	0	1	1
Q223	Q00-Q99	Congenital malformations, deformations and	Circulatory	Other congenital malformations ofpulmonary valve	0	0	0	1	0	0	1	1
Q224	Q00-Q99	Congenital malformations, deformations and	Circulatory	Congenital tricuspid stenosis	0	0	0	1	0	0	1	1
Q225	Q00-Q99	Congenital malformations, deformations and	Circulatory	Ebstein's anomaly	0	0	0	1	0	0	1	1
Q226	Q00-Q99	Congenital malformations, deformations and	Circulatory	Hypoplastic right heart syndrome	0	0	0	1	0	0	1	1
Q228	Q00-Q99	Congenital malformations, deformations and	Circulatory	Other congenital malformations oftricuspid valve	0	0	0	1	0	0	1	1
Q229	Q00-Q99	Congenital malformations, deformations and	Circulatory	Congenital malformation of tricuspid valve, unspecified	0	0	0	1	0	0	1	1
Q23	Q00-Q99	Congenital malformations, deformations and	Circulatory	Congenital malformations of aorticand mitral valves	0	0	0	1	0	0	1	1
Q230	Q00-Q99	Congenital malformations, deformations and	Circulatory	Congenital stenosis of aortic valve	0	0	0	1	0	0	1	1
Q231	Q00-Q99	Congenital malformations, deformations and	Circulatory	Congenital insufficiency of aorticvalve	0	0	0	1	0	0	1	1
Q232	Q00-Q99	Congenital malformations, deformations and	Circulatory	Congenital mitral stenosis	0	0	0	1	0	0	1	1
Q233	Q00-Q99	Congenital malformations, deformations and	Circulatory	Congenital mitral insufficiency	0	0	0	1	0	0	1	1
Q234	Q00-Q99	Congenital malformations, deformations and	Circulatory	Hypoplastic left heart syndrome	0	0	0	1	0	0	1	1
Q238	Q00-Q99	Congenital malformations, deformations and	Circulatory	Other congenital malformations ofaortic and mitral valves	0	0	0	1	0	0	1	1
Q239	Q00-Q99	Congenital malformations, deformations and	Circulatory	Congenital malformation of aorticand mitral valves, unspecified	0	0	0	1	0	0	1	1
Q24	Q00-Q99	Congenital malformations, deformations and	Circulatory	Other congenital malformations ofheart	0	0	0	1	0	0	1	1
Q240	Q00-Q99	Congenital malformations, deformations and	Circulatory	Dextrocardia	0	0	0	1	0	0	1	1
Q242	Q00-Q99	Congenital malformations, deformations and	Circulatory	Cor triatriatum	0	0	0	1	0	0	1	1
Q243	Q00-Q99	Congenital malformations, deformations and	Circulatory	Pulmonary infundibular stenosis	0	0	0	1	0	0	1	1
Q244	Q00-Q99	Congenital malformations, deformations and	Circulatory	Congenital subaortic stenosis	0	0	0	1	0	0	1	1
Q245	Q00-Q99	Congenital malformations, deformations and	Circulatory	Malformation of coronary vessels	0	0	0	1	0	0	1	1
Q246	Q00-Q99	Congenital malformations, deformations and	Circulatory	Congenital heart block	0	0	0	1	0	0	1	1
Q248	Q00-Q99	Congenital malformations, deformations and	Circulatory	Other specified congenital malformations of heart	0	0	0	1	0	0	1	1
Q249	Q00-Q99	Congenital malformations, deformations and	Circulatory	Congenital malformation of heart,unspecified	0	0	0	1	0	0	1	1
Q25	Q00-Q99	Congenital malformations, deformations and	Circulatory	Congenital malformations of greatarteries	0	0	0	1	0	0	1	1
Q251	Q00-Q99	Congenital malformations, deformations and	Circulatory	Coarctation of aorta	0	0	0	1	0	0	1	1
Q252	Q00-Q99	Congenital malformations, deformations and	Circulatory	Atresia of aorta	0	0	0	1	0	0	1	1
Q2521	Q00-Q99	Congenital malformations, deformations and	Circulatory	Interruption of aortic arch	0	0	0	1	0	0	1	1
Q2529	Q00-Q99	Congenital malformations, deformations and	Circulatory	Other atresia of aorta	0	0	0	1	0	0	1	1
Q253	Q00-Q99	Congenital malformations, deformations and	Circulatory	Supravalvular aortic stenosis	0	0	1	0	0	1	0	1
Q254	Q00-Q99	Congenital malformations, deformations and	Circulatory	Other congenital malformations ofaorta	0	0	0	1	0	0	1	1
Q2540	Q00-Q99	Congenital malformations, deformations and	Circulatory	Congenital malformation of aorta unspecified	0	0	0	1	0	0	1	1
Q2541	Q00-Q99	Congenital malformations, deformations and	Circulatory	Absence and aplasia of aorta	0	0	0	1	0	0	1	1
Q2542	Q00-Q99	Congenital malformations, deformations and	Circulatory	Hypoplasia of aorta	0	0	0	1	0	0	1	1
Q2543	Q00-Q99	Congenital malformations, deformations and	Circulatory	Congenital aneurysm of aorta	0	0	0	1	0	0	1	1
Q2544	Q00-Q99	Congenital malformations, deformations and	Circulatory	Congenital dilation of aorta	0	0	0	1	0	0	1	1
Q2545	Q00-Q99	Congenital malformations, deformations and	Circulatory	Double aortic arch	0	0	0	1	0	0	1	1
Q2546	Q00-Q99	Congenital malformations, deformations and	Circulatory	Tortuous aortic arch	0	0	1	0	0	1	0	1
Q2547	Q00-Q99	Congenital malformations, deformations and	Circulatory	Right aortic arch	0	0	0	1	0	0	1	1
Q2548	Q00-Q99	Congenital malformations, deformations and	Circulatory	Anomalous origin of subclavian artery	0	0	0	1	0	0	1	1
Q2549	Q00-Q99	Congenital malformations, deformations and	Circulatory	Other congenital malformations ofaorta	0	0	0	1	0	0	1	1
Q255	Q00-Q99	Congenital malformations, deformations and	Circulatory	Atresia of pulmonary artery	0	0	0	1	0	0	1	1
Q256	Q00-Q99	Congenital malformations, deformations and	Circulatory	Stenosis of pulmonary artery	0	0	0	1	0	0	1	1
Q257	Q00-Q99	Congenital malformations, deformations and	Circulatory	Other congenital malformations ofpulmonary artery	0	0	0	1	0	0	1	1
Q2571	Q00-Q99	Congenital malformations, deformations and	Circulatory	Coarctation of pulmonary artery	0	0	0	1	0	0	1	1
Q2572	Q00-Q99	Congenital malformations, deformations and	Circulatory	Congenital pulmonary arteriovenousmalformation	0	0	0	1	0	0	1	1
Q2579	Q00-Q99	Congenital malformations, deformations and	Circulatory	Other congenital malformations ofpulmonary artery	0	0	0	1	0	0	1	1
Q258	Q00-Q99	Congenital malformations, deformations and	Circulatory	Other congenital malformations ofother great arteries	0	0	0	1	0	0	1	1
Q259	Q00-Q99	Congenital malformations, deformations and	Circulatory	Congenital malformation of great arteries, unspecified	0	0	0	1	0	0	1	1
Q26	Q00-Q99	Congenital malformations, deformations and	Circulatory	Congenital malformations of greatveins	0	0	0	1	0	0	1	1
Q260	Q00-Q99	Congenital malformations, deformations and	Circulatory	Congenital stenosis of vena cava	0	0	0	1	0	0	1	1
Q261	Q00-Q99	Congenital malformations, deformations and	Circulatory	Persistent left superior vena cava	0	0	0	1	0	0	1	1
Q262	Q00-Q99	Congenital malformations, deformations and	Circulatory	Total anomalous pulmonary venous connection	0	0	0	1	0	0	1	1
Q263	Q00-Q99	Congenital malformations, deformations and	Circulatory	Partial anomalous pulmonary venousconnection	0	0	0	1	0	0	1	1
Q264	Q00-Q99	Congenital malformations, deformations and	Circulatory	Anomalous pulmonary venous connection, unspecified	0	0	0	1	0	0	1	1
Q265	Q00-Q99	Congenital malformations, deformations and	Circulatory	Anomalous portal venous connection	0	0	0	1	0	0	1	1
Q266	Q00-Q99	Congenital malformations, deformations and	Circulatory	Portal vein-hepatic artery fistula	0	0	0	1	0	0	1	1
Q268	Q00-Q99	Congenital malformations, deformations and	Circulatory	Other congenital malformations ofgreat veins	0	0	0	1	0	0	1	1
Q269	Q00-Q99	Congenital malformations, deformations and	Circulatory	Congenital malformation of great vein, unspecified	0	0	0	1	0	0	1	1
Q27	Q00-Q99	Congenital malformations, deformations and	Circulatory	Other congenital malformations ofperipheral vascular system	0	0	0	1	0	0	1	1
Q270	Q00-Q99	Congenital malformations, deformations and	Circulatory	Congenital absence and hypoplasiaof umbilical artery	0	0	0	1	0	0	1	1
Q271	Q00-Q99	Congenital malformations, deformations and	Circulatory	Congenital renal artery stenosis	0	0	0	1	0	0	1	1
Q272	Q00-Q99	Congenital malformations, deformations and	Circulatory	Other congenital malformations ofrenal artery	0	0	0	1	0	0	1	1
Q273	Q00-Q99	Congenital malformations, deformations and	Circulatory	Arteriovenous malformation (peripheral)	0	0	0	1	0	0	1	1
Q2730	Q00-Q99	Congenital malformations, deformations and	Circulatory	Arteriovenous malformation, site unspecified	0	0	0	1	0	0	1	1
Q2731	Q00-Q99	Congenital malformations, deformations and	Circulatory	Arteriovenous malformation of vessel of upper limb	0	0	0	1	0	0	1	1
Q2732	Q00-Q99	Congenital malformations, deformations and	Circulatory	Arteriovenous malformation of vessel of lower limb	0	0	0	1	0	0	1	1
Q2733	Q00-Q99	Congenital malformations, deformations and	Circulatory	Arteriovenous malformation of digestive system vessel	0	0	0	1	0	0	1	1
Q2734	Q00-Q99	Congenital malformations, deformations and	Circulatory	Arteriovenous malformation of renal vessel	0	0	0	1	0	0	1	1
Q2739	Q00-Q99	Congenital malformations, deformations and	Circulatory	Arteriovenous malformation, othersite	0	0	0	1	0	0	1	1
Q274	Q00-Q99	Congenital malformations, deformations and	Circulatory	Congenital phlebectasia	0	0	0	1	0	0	1	1
Q278	Q00-Q99	Congenital malformations, deformations and	Circulatory	Other specified congenital malformations of peripheral vascular system	0	0	0	1	0	0	1	1
Q279	Q00-Q99	Congenital malformations, deformations and	Circulatory	Congenital malformation of peripheral vascular system, unspecified	0	0	0	1	0	0	1	1
Q28	Q00-Q99	Congenital malformations, deformations and	Circulatory	Other congenital malformations ofcirculatory system	0	0	0	1	0	0	1	1
Q280	Q00-Q99	Congenital malformations, deformations and	Circulatory	Arteriovenous malformation of precerebral vessels	0	0	0	1	0	0	1	1
Q281	Q00-Q99	Congenital malformations, deformations and	Circulatory	Other malformations of precerebralvessels	0	0	0	1	0	0	1	1
Q282	Q00-Q99	Congenital malformations, deformations and	Circulatory	Arteriovenous malformation of cerebral vessels	0	0	0	1	0	0	1	1
Q283	Q00-Q99	Congenital malformations, deformations and	Circulatory	Other malformations of cerebral vessels	0	0	0	1	0	0	1	1
Q288	Q00-Q99	Congenital malformations, deformations and	Circulatory	Other specified congenital malformations of circulatory system	0	0	0	1	0	0	1	1
Q289	Q00-Q99	Congenital malformations, deformations and	Circulatory	Congenital malformation of circulatory system, unspecified	0	0	0	1	0	0	1	1
Q30	Q00-Q99	Congenital malformations, deformations and	Respiratory	Congenital malformations of nose	0	0	0	1	0	0	1	1
Q300	Q00-Q99	Congenital malformations, deformations and	Respiratory	Choanal atresia	0	0	0	1	0	0	1	1
Q301	Q00-Q99	Congenital malformations, deformations and	Respiratory	Agenesis and underdevelopment of nose	0	0	0	1	0	0	1	1
Q302	Q00-Q99	Congenital malformations, deformations and	Respiratory	Fissured, notched and cleft nose	0	0	0	1	0	0	1	1
Q303	Q00-Q99	Congenital malformations, deformations and	Respiratory	Congenital perforated nasal septum	0	0	0	1	0	0	1	1
Q308	Q00-Q99	Congenital malformations, deformations and	Respiratory	Other congenital malformations ofnose	0	0	0	1	0	0	1	1
Q309	Q00-Q99	Congenital malformations, deformations and	Respiratory	Congenital malformation of nose, unspecified	0	0	0	1	0	0	1	1
Q31	Q00-Q99	Congenital malformations, deformations and	Respiratory	Congenital malformations of larynx	0	0	0	1	0	0	1	1
Q310	Q00-Q99	Congenital malformations, deformations and	Respiratory	Web of larynx	0	0	0	1	0	0	1	1
Q311	Q00-Q99	Congenital malformations, deformations and	Respiratory	Congenital subglottic stenosis	0	0	0	1	0	0	1	1

Q312	Q00-Q99	Congenital malformations, deformations anc	Respiratory	Laryngeal hypoplasia	0	0	0	1	0	0	1	1
Q313	Q00-Q99	Congenital malformations, deformations anc	Respiratory	Laryngocele	0	0	0	1	0	0	1	1
Q315	Q00-Q99	Congenital malformations, deformations anc	Respiratory	Congenital laryngomalacia	0	0	0	1	0	0	1	1
Q318	Q00-Q99	Congenital malformations, deformations anc	Respiratory	Other congenital malformations oflarynx	0	0	0	1	0	0	1	1
Q319	Q00-Q99	Congenital malformations, deformations anc	Respiratory	Congenital malformation of larynx,unspecified	0	0	0	1	0	0	1	1
Q32	Q00-Q99	Congenital malformations, deformations anc	Respiratory	Congenital malformations of trachea and bronchus	0	0	0	1	0	0	1	1
Q320	Q00-Q99	Congenital malformations, deformations anc	Respiratory	Congenital tracheomalacia	0	0	0	1	0	0	1	1
Q321	Q00-Q99	Congenital malformations, deformations anc	Respiratory	Other congenital malformations oftrachea	0	0	0	1	0	0	1	1
Q322	Q00-Q99	Congenital malformations, deformations anc	Respiratory	Congenital bronchomalacia	0	0	0	1	0	0	1	1
Q323	Q00-Q99	Congenital malformations, deformations anc	Respiratory	Congenital stenosis of bronchus	0	0	0	1	0	0	1	1
Q324	Q00-Q99	Congenital malformations, deformations anc	Respiratory	Other congenital malformations ofbronchus	0	0	0	1	0	0	1	1
Q33	Q00-Q99	Congenital malformations, deformations anc	Respiratory	Congenital malformations of lung	0	0	0	1	0	0	1	1
Q330	Q00-Q99	Congenital malformations, deformations anc	Respiratory	Congenital cystic lung	0	0	0	1	0	0	1	1
Q331	Q00-Q99	Congenital malformations, deformations anc	Respiratory	Accessory lobe of lung	0	0	0	1	0	0	1	1
Q332	Q00-Q99	Congenital malformations, deformations anc	Respiratory	Sequestration of lung	0	0	0	1	0	0	1	1
Q333	Q00-Q99	Congenital malformations, deformations anc	Respiratory	Agenesis of lung	0	0	0	1	0	0	1	1
Q334	Q00-Q99	Congenital malformations, deformations anc	Respiratory	Congenital bronchiectasis	0	0	0	1	0	0	1	1
Q336	Q00-Q99	Congenital malformations, deformations anc	Respiratory	Congenital hypoplasia and dysplasia of lung	0	0	0	1	0	0	1	1
Q338	Q00-Q99	Congenital malformations, deformations anc	Respiratory	Other congenital malformations oflung	0	0	0	1	0	0	1	1
Q339	Q00-Q99	Congenital malformations, deformations anc	Respiratory	Congenital malformation of lung, unspecified	0	0	0	1	0	0	1	1
Q34	Q00-Q99	Congenital malformations, deformations anc	Respiratory	Other congenital malformations ofrespiratory system	0	0	0	1	0	0	1	1
Q340	Q00-Q99	Congenital malformations, deformations anc	Respiratory	Anomaly of pleura	0	0	0	1	0	0	1	1
Q341	Q00-Q99	Congenital malformations, deformations anc	Respiratory	Congenital cyst of mediastinum	0	0	0	1	0	0	1	1
Q348	Q00-Q99	Congenital malformations, deformations anc	Respiratory	Other specified congenital malformations of respiratory system	0	0	0	1	0	0	1	1
Q349	Q00-Q99	Congenital malformations, deformations anc	Respiratory	Congenital malformation of respiratory system, unspecified	0	0	0	1	0	0	1	1
Q35	Q00-Q99	Congenital malformations, deformations anc	Digestive	Cleft palate	0	0	0	1	0	0	1	1
Q351	Q00-Q99	Congenital malformations, deformations anc	Digestive	Cleft hard palate	0	0	0	1	0	0	1	1
Q353	Q00-Q99	Congenital malformations, deformations anc	Digestive	Cleft soft palate	0	0	0	1	0	0	1	1
Q355	Q00-Q99	Congenital malformations, deformations anc	Digestive	Cleft hard palate with cleft softpalate	0	0	0	1	0	0	1	1
Q357	Q00-Q99	Congenital malformations, deformations anc	Digestive	Cleft uvula	0	0	0	1	0	0	1	1
Q359	Q00-Q99	Congenital malformations, deformations anc	Digestive	Cleft palate, unspecified	0	0	0	1	0	0	1	1
Q36	Q00-Q99	Congenital malformations, deformations anc	Digestive	Cleft lip	0	0	0	1	0	0	1	1
Q360	Q00-Q99	Congenital malformations, deformations anc	Digestive	Cleft lip, bilateral	0	0	0	1	0	0	1	1
Q361	Q00-Q99	Congenital malformations, deformations anc	Digestive	Cleft lip, median	0	0	0	1	0	0	1	1
Q369	Q00-Q99	Congenital malformations, deformations anc	Digestive	Cleft lip, unilateral	0	0	0	1	0	0	1	1
Q37	Q00-Q99	Congenital malformations, deformations anc	Digestive	Cleft palate with cleft lip	0	0	0	1	0	0	1	1
Q370	Q00-Q99	Congenital malformations, deformations anc	Digestive	Cleft hard palate with bilateral cleft lip	0	0	0	1	0	0	1	1
Q371	Q00-Q99	Congenital malformations, deformations anc	Digestive	Cleft hard palate with unilateralcleft lip	0	0	0	1	0	0	1	1
Q372	Q00-Q99	Congenital malformations, deformations anc	Digestive	Cleft soft palate with bilateral cleft lip	0	0	0	1	0	0	1	1
Q373	Q00-Q99	Congenital malformations, deformations anc	Digestive	Cleft soft palate with unilateralcleft lip	0	0	0	1	0	0	1	1
Q374	Q00-Q99	Congenital malformations, deformations anc	Digestive	Cleft hard and soft palate with bilateral cleft lip	0	0	0	1	0	0	1	1
Q375	Q00-Q99	Congenital malformations, deformations anc	Digestive	Cleft hard and soft palate with unilateral cleft lip	0	0	0	1	0	0	1	1
Q378	Q00-Q99	Congenital malformations, deformations anc	Digestive	Unspecified cleft palate with bilateral cleft lip	0	0	0	1	0	0	1	1
Q379	Q00-Q99	Congenital malformations, deformations anc	Digestive	Unspecified cleft palate with unilateral cleft lip	0	0	0	1	0	0	1	1
Q38	Q00-Q99	Congenital malformations, deformations anc	Digestive	Other congenital malformations oftongue, mouth and pharynx	0	0	0	1	0	0	1	1
Q380	Q00-Q99	Congenital malformations, deformations anc	Digestive	Congenital malformations of lips,not elsewhere classified	0	0	0	1	0	0	1	1
Q382	Q00-Q99	Congenital malformations, deformations anc	Digestive	Macroglossia	0	0	0	1	0	0	1	1
Q383	Q00-Q99	Congenital malformations, deformations anc	Digestive	Other congenital malformations oftongue	0	0	0	1	0	0	1	1
Q384	Q00-Q99	Congenital malformations, deformations anc	Digestive	Congenital malformations of salivary glands and ducts	0	0	0	1	0	0	1	1
Q385	Q00-Q99	Congenital malformations, deformations anc	Digestive	Congenital malformations of palate, not elsewhere classified	0	0	0	1	0	0	1	1
Q386	Q00-Q99	Congenital malformations, deformations anc	Digestive	Other congenital malformations ofmouth	0	0	0	1	0	0	1	1
Q387	Q00-Q99	Congenital malformations, deformations anc	Digestive	Congenital pharyngeal pouch	0	0	0	1	0	0	1	1
Q388	Q00-Q99	Congenital malformations, deformations anc	Digestive	Other congenital malformations ofpharynx	0	0	0	1	0	0	1	1
Q39	Q00-Q99	Congenital malformations, deformations anc	Digestive	Congenital malformations of esophagus	0	0	0	1	0	0	1	1
Q390	Q00-Q99	Congenital malformations, deformations anc	Digestive	Atresia of esophagus without fistula	0	0	0	1	0	0	1	1
Q391	Q00-Q99	Congenital malformations, deformations anc	Digestive	Atresia of esophagus with tracheo-esophageal fistula	0	0	0	1	0	0	1	1
Q392	Q00-Q99	Congenital malformations, deformations anc	Digestive	Congenital tracheo-esophageal fistula without atresia	0	0	0	1	0	0	1	1
Q393	Q00-Q99	Congenital malformations, deformations anc	Digestive	Congenital stenosis and strictureof esophagus	0	0	0	1	0	0	1	1
Q394	Q00-Q99	Congenital malformations, deformations anc	Digestive	Esophageal web	0	0	0	1	0	0	1	1
Q395	Q00-Q99	Congenital malformations, deformations anc	Digestive	Congenital dilatation of esophagus	0	0	0	1	0	0	1	1
Q396	Q00-Q99	Congenital malformations, deformations anc	Digestive	Congenital diverticulum of esophagus	0	0	0	1	0	0	1	1
Q398	Q00-Q99	Congenital malformations, deformations anc	Digestive	Other congenital malformations ofesophagus	0	0	0	1	0	0	1	1
Q399	Q00-Q99	Congenital malformations, deformations anc	Digestive	Congenital malformation of esophagus, unspecified	0	0	0	1	0	0	1	1
Q40	Q00-Q99	Congenital malformations, deformations anc	Digestive	Other congenital malformations ofupper alimentary tract	0	0	0	1	0	0	1	1
Q400	Q00-Q99	Congenital malformations, deformations anc	Digestive	Congenital hypertrophic pyloric stenosis	0	0	0	1	0	0	1	1
Q401	Q00-Q99	Congenital malformations, deformations anc	Digestive	Congenital hiatus hernia	0	0	0	1	0	0	1	1
Q402	Q00-Q99	Congenital malformations, deformations anc	Digestive	Other specified congenital malformations of stomach	0	0	0	1	0	0	1	1
Q403	Q00-Q99	Congenital malformations, deformations anc	Digestive	Congenital malformation of stomach, unspecified	0	0	0	1	0	0	1	1
Q408	Q00-Q99	Congenital malformations, deformations anc	Digestive	Other specified congenital malformations of upper alimentary tract	0	0	0	1	0	0	1	1
Q409	Q00-Q99	Congenital malformations, deformations anc	Digestive	Congenital malformation of upper alimentary tract, unspecified	0	0	0	1	0	0	1	1
Q41	Q00-Q99	Congenital malformations, deformations anc	Digestive	Congenital absence, atresia and stenosis of small intestine	0	0	0	1	0	0	1	1
Q410	Q00-Q99	Congenital malformations, deformations anc	Digestive	Congenital absence, atresia and stenosis of duodenum	0	0	0	1	0	0	1	1
Q411	Q00-Q99	Congenital malformations, deformations anc	Digestive	Congenital absence, atresia and stenosis of jejunum	0	0	0	1	0	0	1	1
Q412	Q00-Q99	Congenital malformations, deformations anc	Digestive	Congenital absence, atresia and stenosis of ileum	0	0	0	1	0	0	1	1
Q418	Q00-Q99	Congenital malformations, deformations anc	Digestive	Congenital absence, atresia and stenosis of other specified parts of small intestine	0	0	0	1	0	0	1	1
Q419	Q00-Q99	Congenital malformations, deformations anc	Digestive	Congenital absence, atresia and stenosis of small intestine, part unspecified	0	0	0	1	0	0	1	1
Q42	Q00-Q99	Congenital malformations, deformations anc	Digestive	Congenital absence, atresia and stenosis of large intestine	0	0	0	1	0	0	1	1
Q420	Q00-Q99	Congenital malformations, deformations anc	Digestive	Congenital absence, atresia and stenosis of rectum with fistula	0	0	0	1	0	0	1	1
Q421	Q00-Q99	Congenital malformations, deformations anc	Digestive	Congenital absence, atresia and stenosis of rectum without fistula	0	0	0	1	0	0	1	1
Q422	Q00-Q99	Congenital malformations, deformations anc	Digestive	Congenital absence, atresia and stenosis of anus with fistula	0	0	0	1	0	0	1	1
Q423	Q00-Q99	Congenital malformations, deformations anc	Digestive	Congenital absence, atresia and stenosis of anus without fistula	0	0	0	1	0	0	1	1
Q428	Q00-Q99	Congenital malformations, deformations anc	Digestive	Congenital absence, atresia and stenosis of other parts of large intestine	0	0	0	1	0	0	1	1
Q429	Q00-Q99	Congenital malformations, deformations anc	Digestive	Congenital absence, atresia and stenosis of large intestine, part unspecified	0	0	0	1	0	0	1	1
Q43	Q00-Q99	Congenital malformations, deformations anc	Digestive	Other congenital malformations ofintestine	0	0	0	1	0	0	1	1
Q430	Q00-Q99	Congenital malformations, deformations anc	Digestive	Meckel's diverticulum (displaced)(hypertrophic)	0	0	0	1	0	0	1	1
Q431	Q00-Q99	Congenital malformations, deformations anc	Digestive	Hirschsprung's disease	0	0	0	1	0	0	1	1
Q432	Q00-Q99	Congenital malformations, deformations anc	Digestive	Other congenital functional disorders of colon	0	0	0	1	0	0	1	1
Q433	Q00-Q99	Congenital malformations, deformations anc	Digestive	Congenital malformations of intestinal fixation	0	0	0	1	0	0	1	1
Q434	Q00-Q99	Congenital malformations, deformations anc	Digestive	Duplication of intestine	0	0	0	1	0	0	1	1
Q435	Q00-Q99	Congenital malformations, deformations anc	Digestive	Ectopic anus	0	0	0	1	0	0	1	1
Q436	Q00-Q99	Congenital malformations, deformations anc	Digestive	Congenital fistula of rectum and anus	0	0	0	1	0	0	1	1
Q437	Q00-Q99	Congenital malformations, deformations anc	Digestive	Persistent cloaca	0	0	0	1	0	0	1	1
Q438	Q00-Q99	Congenital malformations, deformations anc	Digestive	Other specified congenital malformations of intestine	0	0	0	1	0	0	1	1
Q439	Q00-Q99	Congenital malformations, deformations anc	Digestive	Congenital malformation of intestine, unspecified	0	0	0	1	0	0	1	1
Q44	Q00-Q99	Congenital malformations, deformations anc	Digestive	Congenital malformations of gallbladder, bile ducts and liver	0	0	0	1	0	0	1	1
Q440	Q00-Q99	Congenital malformations, deformations anc	Digestive	Agenesis, aplasia and hypoplasia of gallbladder	0	0	0	1	0	0	1	1
Q441	Q00-Q99	Congenital malformations, deformations anc	Digestive	Other congenital malformations ofgallbladder	0	0	0	1	0	0	1	1
Q442	Q00-Q99	Congenital malformations, deformations anc	Digestive	Atresia of bile ducts	0	0	0	1	0	0	1	1
Q443	Q00-Q99	Congenital malformations, deformations anc	Digestive	Congenital stenosis and strictureof bile ducts	0	0	0	1	0	0	1	1
Q444	Q00-Q99	Congenital malformations, deformations anc	Digestive	Choledochal cyst	0	0	0	1	0	0	1	1
Q445	Q00-Q99	Congenital malformations, deformations anc	Digestive	Other congenital malformations ofbile ducts	0	0	0	1	0	0	1	1
Q446	Q00-Q99	Congenital malformations, deformations anc	Digestive	Cystic disease of liver	0	0	0	1	0	0	1	1
Q447	Q00-Q99	Congenital malformations, deformations anc	Digestive	Other congenital malformations ofliver	0	0	0	1	0	0	1	1
Q45	Q00-Q99	Congenital malformations, deformations anc	Digestive	Other congenital malformations ofdigestive system	0	0	0	1	0	0	1	1
Q450	Q00-Q99	Congenital malformations, deformations anc	Digestive	Agenesis, aplasia and hypoplasia of pancreas	0	0	0	1	0	0	1	1
Q451	Q00-Q99	Congenital malformations, deformations anc	Digestive	Annular pancreas	0	0	0	1	0	0	1	1
Q452	Q00-Q99	Congenital malformations, deformations anc	Digestive	Congenital pancreatic cyst	0	0	0	1	0	0	1	1
Q453	Q00-Q99	Congenital malformations, deformations anc	Digestive	Other congenital malformations ofpancreas and pancreatic duct	0	0	0	1	0	0	1	1
Q458	Q00-Q99	Congenital malformations, deformations anc	Digestive	Other specified congenital malformations of digestive system	0	0	0	1	0	0	1	1
Q459	Q00-Q99	Congenital malformations, deformations anc	Digestive	Congenital malformation of digestive system, unspecified	0	0	0	1	0	0	1	1
Q50	Q00-Q99	Congenital malformations, deformations anc	Genital	Congenital malformations of ovaries, fallopian tubes and broad ligaments	0	0	0	1	0	0	1	1
Q500	Q00-Q99	Congenital malformations, deformations anc	Genital	Congenital absence of ovary	0	0	0	1	0	0	1	1
Q5001	Q00-Q99	Congenital malformations, deformations anc	Genital	Congenital absence of ovary, unilateral	0	0	0	1	0	0	1	1
Q5002	Q00-Q99	Congenital malformations, deformations anc	Genital	Congenital absence of ovary, bilateral	0	0	0	1	0	0	1	1
Q501	Q00-Q99	Congenital malformations, deformations anc	Genital	Developmental ovarian cyst	0	0	0	1	0	0	1	1
Q502	Q00-Q99	Congenital malformations, deformations anc	Genital	Congenital torsion of ovary	0	0	0	1	0	0	1	1
Q503	Q00-Q99	Congenital malformations, deformations anc	Genital	Other congenital malformations ofovary	0	0	0	1	0	0	1	1
Q5031	Q00-Q99	Congenital malformations, deformations anc	Genital	Accessory ovary	0	0	0	1	0	0	1	1
Q5032	Q00-Q99	Congenital malformations, deformations anc	Genital	Ovarian streak	0	0	0	1	0	0	1	1
Q5039	Q00-Q99	Congenital malformations, deformations anc	Genital	Other congenital malformation of ovary	0	0	0	1	0	0	1	1
Q504	Q00-Q99	Congenital malformations, deformations anc	Genital	Embryonic cyst of fallopian tube	0	0	0	0	0	0	1	1
Q506	Q00-Q99	Congenital malformations, deformations anc	Genital	Other congenital malformations offallopian tube and broad ligament	0	0	0	1	0	0	1	1
Q51	Q00-Q99	Congenital malformations, deformations anc	Genital	Congenital malformations of uterusand cervix	0	0	0	1	0	0	1	1
Q510	Q00-Q99	Congenital malformations, deformations anc	Genital	Agenesis and aplasia of uterus	0	0	0	1	0	0	1	1
Q511	Q00-Q99	Congenital malformations, deformations anc	Genital	Doubling of uterus with doubling of cervix and vagina	0	0	0	1	0	0	1	1
Q5110	Q00-Q99	Congenital malformations, deformations anc	Genital	Doubling of uterus with doubling of cervix and vagina without obstruction	0	0	0	1	0	0	1	1
Q5111	Q00-Q99	Congenital malformations, deformations anc	Genital	Doubling of uterus with doubling of cervix and vagina with obstruction	0	0	0	1	0	0	1	1
Q512	Q00-Q99	Congenital malformations, deformations anc	Genital	Other doubling of uterus	0	0	0	1	0	0	1	1
Q513	Q00-Q99	Congenital malformations, deformations anc	Genital	Bicornate uterus	0	0	0	1	0	0	1	1
Q514	Q00-Q99	Congenital malformations, deformations anc	Genital	Unicornate uterus	0	0	0	1	0	0	1	1
Q515	Q00-Q99	Congenital malformations, deformations anc	Genital	Agenesis and aplasia of cervix	0	0	0	1	0	0	1	1
Q516	Q00-Q99	Congenital malformations, deformations anc	Genital	Embryonic cyst of cervix	0	0	0	0	0	0	1	1
Q517	Q00-Q99	Congenital malformations, deformations anc	Genital	Congenital fistulae between uterusand digestive and urinary tracts	0	0	0	1	0	0	1	1
Q518	Q00-Q99	Congenital malformations, deformations anc	Genital	Other congenital malformations ofuterus and cervix	0	0	0	1	0	0	1	1
Q5181	Q00-Q99	Congenital malformations, deformations anc	Genital	Other congenital malformations ofuterus	0	0	0	1	0	0	1	1
Q51810	Q00-Q99	Congenital malformations, deformations anc	Genital	Arcuate uterus	0	0	0	1	0	0	1	1
Q51811	Q00-Q99	Congenital malformations, deformations anc	Genital	Hypoplasia of uterus	0	0	0	1	0	0	1	1
Q51818	Q00-Q99	Congenital malformations, deformations anc	Genital	Other congenital malformations ofuterus	0	0	0	1	0	0	1	1
Q5182	Q00-Q99	Congenital malformations, deformations anc	Genital	Other congenital malformations ofcervix	0	0	0	1	0	0	1	1
Q51820	Q00-Q99	Congenital malformations, deformations anc	Genital	Cervical duplication	0	0	0	1	0	0	1	1
Q51821	Q00-Q99	Congenital malformations, deformations anc	Genital	Hypoplasia of cervix	0	0	0	1	0	0	1	1
Q51828	Q00-Q99	Congenital malformations, deformations anc	Genital	Other congenital malformations ofcervix	0	0	0	1	0	0	1	1
Q519	Q00-Q99	Congenital malformations, deformations anc	Genital	Congenital malformation of uterusand cervix, unspecified	0	0	0	1	0	0	1	1
Q52	Q00-Q99	Congenital malformations, deformations anc	Genital	Other congenital malformations offemale genitalia	0	0	0	1	0	0	1	1
Q520	Q00-Q99	Congenital malformations, deformations anc	Genital	Congenital absence of vagina	0	0	0	1	0	0	1	1
Q521	Q00-Q99	Congenital malformations, deformations anc	Genital	Doubling of vagina	0	0	0	1	0	0	1	1
Q5210	Q00-Q99	Congenital malformations, deformations anc	Genital	Doubling of vagina, unspecified	0	0	0	1	0	0	1	1
Q5211	Q00-Q99	Congenital malformations, deformations anc	Genital	Transverse vaginal septum	0	0	0	1	0	0	1	1
Q5212	Q00-Q99	Congenital malformations, deformations anc	Genital	Longitudinal vaginal septum	0	0	0	1	0	0	1	1
Q52120	Q00-Q99	Congenital malformations, deformations anc	Genital	Longitudinal vaginal septum, nonobstructing	0	0	0	1	0	0	1	1
Q52121	Q00-Q99	Congenital malformations, deformations anc	Genital	Longitudinal vaginal septum, obstructing, right side	0	0	0	1	0	0	1	1
Q52122	Q00-Q99	Congenital malformations, deformations anc	Genital	Longitudinal vaginal septum, obstructing, left side	0	0	0	1	0	0	1	1
Q52123	Q00-Q99	Congenital malformations, deformations anc	Genital	Longitudinal vaginal septum, microperforate, right side	0	0	0	1	0	0	1	1
Q52124	Q00-Q99	Congenital malformations, deformations anc	Genital	Longitudinal vaginal septum, microperforate, left side	0	0	0	1	0	0	1	1
Q52129	Q00-Q99	Congenital malformations, deformations anc	Genital	Other and unspecified longitudinalvaginal septum	0	0	0	1	0	0	1	1
Q522	Q00-Q99	Congenital malformations, deformations anc	Genital	Congenital rectovaginal fistula	0	0	0	1	0	0	1	1
Q523	Q00-Q99	Congenital malformations, deformations anc	Genital	Imperforate hymen	0	0	0	1	0	0	1	1
Q524	Q00-Q99	Congenital malformations, deformations anc	Genital	Other congenital malformations ofvagina	0	0	0	1	0	0	1	1
Q525	Q00-Q99	Congenital malformations, deformations anc	Genital	Fusion of labia	0	0	0	1	0	0	1	1
Q526	Q00-Q99	Congenital malformations, deformations anc	Genital	Congenital malformation of clitoris	0	0	0	1	0	0	1	1
Q527	Q00-Q99	Congenital malformations, deformations anc	Genital	Other and unspecified congenital malformations of vulva	0	0	0	1	0	0	1	1
Q5270	Q00-Q99	Congenital malformations, deformations anc	Genital	Unspecified congenital malformations of vulva	0	0	0	1	0	0	1	1
Q5271	Q00-Q99	Congenital malformations, deformations anc	Genital	Congenital absence of vulva	0	0	0	1	0	0	1	1
Q5279	Q00-Q99	Congenital malformations, deformations anc	Genital	Other congenital malformations ofvulva	0	0	0	1	0	0	1	1
Q528	Q00-Q99	Congenital malformations, deformations anc	Genital	Other specified congenital malformations of female genitalia	0	0	0	1	0	0	1	1
Q529	Q00-Q99	Congenital malformations, deformations anc	Genital	Congenital malformation of femalegenitalia, unspecified	0	0	0	1	0	0	1	1
Q53	Q00-Q99	Congenital malformations, deformations anc	Genital	Undescended and ectopic testicle	0	0	0	1	0	0	1	1
Q530	Q00-Q99	Congenital malformations, deformations anc	Genital	Ectopic testis	0	0	0	1	0	0	1	1
Q5300	Q00-Q99	Congenital malformations, deformations anc	Genital	Ectopic testis, unspecified	0	0	0	1	0	0	1	1
Q5301	Q00-Q99	Congenital malformations, deformations anc	Genital	Ectopic testis, unilateral	0	0	0	1	0	0	1	1
Q5302	Q00-Q99	Congenital malformations, deformations anc	Genital	Ectopic testes, bilateral	0	0	0	1	0	0	1	1
Q531	Q00-Q99	Congenital malformations, deformations anc	Genital	Undescended testicle, unilateral	0	0	0	1	0	0	1	1
Q5311	Q00-Q99	Congenital malformations, deformations anc	Genital	Abdominal testis, unilateral	0	0	0	1	0	0	1	1
Q53111	Q00-Q99	Congenital malformations, deformations anc	Genital	Unilateral intraabdominal testis	0	0	0	1	0	0	1	1
Q5312	Q00-Q99	Congenital malformations, deformations anc	Genital	Ectopic perineal testis, unilateral	0	0	0	1	0	0	1	1
Q5313	Q00-Q99	Congenital malformations, deformations anc	Genital	Unilateral high scrotal testis	0	0	0	0	0	0	1	1
Q532	Q00-Q99	Congenital malformations, deformations anc	Genital	Undescended testicle, bilateral	0	0	0	1	0	0	1	1
Q5320	Q00-Q99	Congenital malformations, deformations anc	Genital	Undescended testicle, unspecified,bilateral	0	0	0	1	0	0	1	1
Q5321	Q00-Q99	Congenital malformations, deformations anc	Genital	Abdominal testis, bilateral	0	0	0	1	0	0	1	1
Q53211	Q00-Q99	Congenital malformations, deformations anc	Genital	Bilateral intraabdominal testes	0	0	0	1	0	0	1	1
Q5322	Q00-Q99	Congenital malformations, deformations anc	Genital	Ectopic perineal testis, bilateral	0	0	0	1	0	0	1	1
Q54	Q00-Q99	Congenital malformations, deformations anc	Genital	Hypospadias	0	0	0	1	0	0	1	1
Q540	Q00-Q99	Congenital malformations, deformations anc	Genital	Hypospadias, balanic	0	0	0	1	0	0	1	1
Q541	Q00-Q99	Congenital malformations, deformations anc	Genital	Hypospadias, penile	0	0	0	1	0	0	1	1
Q542	Q00-Q99	Congenital malformations, deformations anc	Genital	Hypospadias, penoscrotal	0	0	0	1	0	0	1	1
Q543	Q00-Q99	Congenital malformations, deformations anc	Genital	Hypospadias, perineal	0	0	0	1	0	0	1	1
Q544	Q00-Q99	Congenital malformations, deformations anc	Genital	Congenital chordee	0	0	0	1	0	0	1	1
Q548	Q00-Q99	Congenital malformations, deformations anc	Genital	Other hypospadias	0	0	0	1	0	0	1	1
Q549	Q00-Q99	Congenital malformations, deformations anc	Genital	Hypospadias, unspecified	0	0	0	1	0	0	1	1
Q55	Q00-Q99	Congenital malformations, deformations anc	Genital	Other congenital malformations ofmale genital organs	0	0	0	1	0	0	1	1
Q550	Q00-Q99	Congenital malformations, deformations anc	Genital	Absence and aplasia of testis	0	0	0	1	0	0	1	1
Q551	Q00-Q99	Congenital malformations, deformations anc	Genital	Hypoplasia of testis and scrotum	0	0	0	1	0	0	1	1
Q552	Q00-Q99	Congenital malformations, deformations anc	Genital	Other and unspecified congenital malformations of testis and scrotum	0	0	0	1	0	0	1	1
Q5520	Q00-Q99	Congenital malformations, deformations anc	Genital	Unspecified congenital malformations of testis and scrotum	0	0	0	1	0	0	1	1
Q5521	Q00-Q99	Congenital malformations, deformations anc	Genital	Polyorchism	0	0	0	1	0	0	1	1
Q5523	Q00-Q99	Congenital malformations, deformations anc	Genital	Scrotal transposition	0	0	0	1	0	0	1	1
Q5529	Q00-Q99	Congenital malformations, deformations anc	Genital	Other congenital malformations oftestis and scrotum	0	0	0	1	0	0	1	1
Q553	Q00-Q99	Congenital malformations, deformations anc	Genital	Atresia of vas deferens	0	0	0	1	0	0	1	1
Q554	Q00-Q99	Congenital malformations, deformations anc	Genital	Other congenital malformations ofvas deferens, epididymis, seminal vesicles and pros	0	0	0	1	0	0	1	1
Q555	Q00-Q99	Congenital malformations, deformations anc	Genital	Congenital absence and aplasia ofpenis	0	0	0	1	0	0	1	1
Q556	Q00-Q99	Congenital malformations, deformations anc	Genital	Other congenital malformations ofpenis	0	0	0	1	0	0	1	1
Q5562	Q00-Q99	Congenital malformations, deformations anc	Genital	Hypoplasia of penis	0	0	0	1	0	0	1	1
Q5563	Q00-Q99	Congenital malformations, deformations anc	Genital	Congenital torsion of penis	0	0	0	1	0	0	1	1
Q5564	Q00-Q99	Congenital malformations, deformations anc	Genital	Hidden penis	0	0	0	1	0	0	1	1
Q5569	Q00-Q99	Congenital malformations, deformations anc	Genital	Other congenital malformation of penis	0	0	0	1	0	0	1	1
Q557	Q00-Q99	Congenital malformations, deformations anc	Genital	Congenital vasocutaneous fistula	0	0	0	1	0	0	1	1
Q558	Q00-Q99	Congenital malformations, deformations anc	Genital	Other specified congenital malformations of male genital organs	0	0	0	1	0	0	1	1
Q559	Q00-Q99	Congenital malformations, deformations anc	Genital	Congenital malformation of male genital organ, unspecified	0	0	0	1	0	0	1	1
Q56	Q00-Q99	Congenital malformations, deformations anc	Genital	Indeterminate sex and pseudohermaphroditism	0	0	0	1	0	0	1	1
Q560	Q00-Q99	Congenital malformations, deformations anc	Genital	Hermaphroditism, not elsewhere classified	0	0	0	1	0	0	1	1
Q561	Q00-Q99	Congenital malformations, deformations anc	Genital	Male pseudohermaphroditism, not elsewhere classified	0	0	0	1	0	0	1	1
Q562	Q00-Q99	Congenital malformations, deformations anc	Genital	Female pseudohermaphroditism, notelsewhere classified	0	0	0	1	0	0	1	1
Q563	Q00-Q99	Congenital malformations, deformations anc	Genital	Pseudohermaphroditism, unspecified	0	0	0	1	0	0	1	1
Q564	Q00-Q99	Congenital malformations, deformations anc	Genital	Indeterminate sex, unspecified	0	0	0	1	0	0	1	1
Q60	Q00-Q99	Congenital malformations, deformations anc	Urinary	Renal agenesis and other reductiondefects of kidney	0	0	0	1	0	0	1	1
Q600	Q00-Q99	Congenital malformations, deformations anc	Urinary	Renal agenesis, unilateral	0	0	0	1	0	0	1	1
Q601	Q00-Q99	Congenital malformations, deformations anc	Urinary	Renal agenesis, bilateral	0	0	0	1	0	0	1	1
Q602	Q00-Q99	Congenital malformations, deformations anc	Urinary	Renal agenesis, unspecified	0	0	0	1	0	0	1	1
Q603	Q00-Q99	Congenital malformations, deformations anc	Urinary	Renal hypoplasia, unilateral	0	0	0	1	0	0	1	1
Q604	Q00-Q99	Congenital malformations, deformations anc	Urinary	Renal hypoplasia, bilateral	0	0	0	1	0	0	1	1
Q605	Q00-Q99	Congenital malformations, deformations anc	Urinary	Renal hypoplasia, unspecified	0	0	0	1	0	0	1	1
Q606	Q00-Q99	Congenital malformations, deformations anc	Urinary	Potter's syndrome	0	0	0	1	0	0	1	1
Q61	Q00-Q99	Congenital malformations, deformations anc	Urinary	Cystic kidney disease	0	0	0	1	0	0	1	1
Q610	Q00-Q99	Congenital malformations, deformations anc	Urinary	Congenital renal cyst	0	0	0	1	0	0	1	1
Q6100	Q00-Q99	Congenital malformations, deformations anc	Urinary	Congenital renal cyst, unspecified	0	0	0	1	0	0	1	1
Q6101	Q00-Q99	Congenital malformations, deformations anc	Urinary	Congenital single renal cyst	0	0	0	1	0	0	1	1
Q6102	Q00-Q99	Congenital malformations, deformations anc	Urinary	Congenital multiple renal cysts	0	0	0	1	0	0	1	1
Q611	Q00-Q99	Congenital malformations, deformations anc	Urinary	Polycystic kidney, infantile type	0	0	1	0	0	1	0	1

Q6111	Q00-Q99	Congenital malformations, deformations anc	Urinary	Cystic dilatation of collecting ducts	0	0	0	1	0	0	1	1
Q6119	Q00-Q99	Congenital malformations, deformations anc	Urinary	Other polycystic kidney, infantiletype	0	1	0	0	1	0	0	1
Q612	Q00-Q99	Congenital malformations, deformations anc	Urinary	Polycystic kidney, adult type	1	1	0	0	1	0	0	1
Q613	Q00-Q99	Congenital malformations, deformations anc	Urinary	Polycystic kidney, unspecified	0	0	0	1	0	0	1	1
Q614	Q00-Q99	Congenital malformations, deformations anc	Urinary	Renal dysplasia	0	0	0	1	0	0	1	1
Q615	Q00-Q99	Congenital malformations, deformations anc	Urinary	Medullary cystic kidney	0	0	0	1	0	0	1	1
Q618	Q00-Q99	Congenital malformations, deformations anc	Urinary	Other cystic kidney diseases	0	0	0	1	0	0	1	1
Q619	Q00-Q99	Congenital malformations, deformations anc	Urinary	Cystic kidney disease, unspecified	0	0	0	1	0	0	1	1
Q62	Q00-Q99	Congenital malformations, deformations anc	Urinary	Congenital obstructive defects ofrenal pelvis and congenital malformations of ureter	0	0	0	1	0	0	1	1
Q620	Q00-Q99	Congenital malformations, deformations anc	Urinary	Congenital hydronephrosis	0	0	0	1	0	0	1	1
Q621	Q00-Q99	Congenital malformations, deformations anc	Urinary	Congenital occlusion of ureter	0	0	0	1	0	0	1	1
Q6210	Q00-Q99	Congenital malformations, deformations anc	Urinary	Congenital occlusion of ureter, unspecified	0	0	0	1	0	0	1	1
Q6211	Q00-Q99	Congenital malformations, deformations anc	Urinary	Congenital occlusion of ureteropelvic junction	0	0	0	1	0	0	1	1
Q6212	Q00-Q99	Congenital malformations, deformations anc	Urinary	Congenital occlusion of ureterovesical orifice	0	0	0	1	0	0	1	1
Q622	Q00-Q99	Congenital malformations, deformations anc	Urinary	Congenital megaureter	0	0	0	1	0	0	1	1
Q623	Q00-Q99	Congenital malformations, deformations anc	Urinary	Other obstructive defects of renalpelvis and ureter	0	0	0	1	0	0	1	1
Q6231	Q00-Q99	Congenital malformations, deformations anc	Urinary	Congenital ureterocele, orthotopic	0	0	0	1	0	0	1	1
Q6232	Q00-Q99	Congenital malformations, deformations anc	Urinary	Cecoureterocele	0	0	0	1	0	0	1	1
Q6239	Q00-Q99	Congenital malformations, deformations anc	Urinary	Other obstructive defects of renalpelvis and ureter	0	0	0	1	0	0	1	1
Q624	Q00-Q99	Congenital malformations, deformations anc	Urinary	Agenesis of ureter	0	0	0	1	0	0	1	1
Q625	Q00-Q99	Congenital malformations, deformations anc	Urinary	Duplication of ureter	0	0	0	1	0	0	1	1
Q626	Q00-Q99	Congenital malformations, deformations anc	Urinary	Malposition of ureter	0	0	0	1	0	0	1	1
Q6260	Q00-Q99	Congenital malformations, deformations anc	Urinary	Malposition of ureter, unspecified	0	0	0	1	0	0	1	1
Q6263	Q00-Q99	Congenital malformations, deformations anc	Urinary	Anomalous implantation of ureter	0	0	0	1	0	0	1	1
Q6269	Q00-Q99	Congenital malformations, deformations anc	Urinary	Other malposition of ureter	0	0	0	1	0	0	1	1
Q627	Q00-Q99	Congenital malformations, deformations anc	Urinary	Congenital vesico-uretero-renal reflux	0	0	0	1	0	0	1	1
Q628	Q00-Q99	Congenital malformations, deformations anc	Urinary	Other congenital malformations ofureter	0	0	0	1	0	0	1	1
Q63	Q00-Q99	Congenital malformations, deformations anc	Urinary	Other congenital malformations ofkidney	0	0	0	1	0	0	1	1
Q630	Q00-Q99	Congenital malformations, deformations anc	Urinary	Accessory kidney	0	0	0	1	0	0	1	1
Q631	Q00-Q99	Congenital malformations, deformations anc	Urinary	Lobulated, fused and horseshoe kidney	0	0	0	1	0	0	1	1
Q632	Q00-Q99	Congenital malformations, deformations anc	Urinary	Ectopic kidney	0	0	0	1	0	0	1	1
Q633	Q00-Q99	Congenital malformations, deformations anc	Urinary	Hyperplastic and giant kidney	0	0	0	1	0	0	1	1
Q638	Q00-Q99	Congenital malformations, deformations anc	Urinary	Other specified congenital malformations of kidney	0	0	0	1	0	0	1	1
Q639	Q00-Q99	Congenital malformations, deformations anc	Urinary	Congenital malformation of kidney,unspecified	0	0	0	1	0	0	1	1
Q64	Q00-Q99	Congenital malformations, deformations anc	Urinary	Other congenital malformations ofurinary system	0	0	0	1	0	0	1	1
Q640	Q00-Q99	Congenital malformations, deformations anc	Urinary	Epispadias	0	0	0	1	0	0	1	1
Q641	Q00-Q99	Congenital malformations, deformations anc	Urinary	Exstrophy of urinary bladder	0	0	0	1	0	0	1	1
Q6410	Q00-Q99	Congenital malformations, deformations anc	Urinary	Exstrophy of urinary bladder, unspecified	0	0	0	1	0	0	1	1
Q6411	Q00-Q99	Congenital malformations, deformations anc	Urinary	Supravesical fissure of urinary bladder	0	0	0	1	0	0	1	1
Q6412	Q00-Q99	Congenital malformations, deformations anc	Urinary	Cloacal extrophy of urinary bladder	0	0	0	1	0	0	1	1
Q6419	Q00-Q99	Congenital malformations, deformations anc	Urinary	Other exstrophy of urinary bladder	0	0	0	1	0	0	1	1
Q642	Q00-Q99	Congenital malformations, deformations anc	Urinary	Congenital posterior urethral valves	0	0	0	1	0	0	1	1
Q643	Q00-Q99	Congenital malformations, deformations anc	Urinary	Other atresia and stenosis of urethra and bladder neck	0	0	0	1	0	0	1	1
Q6431	Q00-Q99	Congenital malformations, deformations anc	Urinary	Congenital bladder neck obstruction	0	0	0	1	0	0	1	1
Q6432	Q00-Q99	Congenital malformations, deformations anc	Urinary	Congenital stricture of urethra	0	0	0	1	0	0	1	1
Q6439	Q00-Q99	Congenital malformations, deformations anc	Urinary	Other atresia and stenosis of urethra and bladder neck	0	0	0	1	0	0	1	1
Q644	Q00-Q99	Congenital malformations, deformations anc	Urinary	Malformation of urachus	0	0	0	1	0	0	1	1
Q645	Q00-Q99	Congenital malformations, deformations anc	Urinary	Congenital absence of bladder andurethra	0	0	0	1	0	0	1	1
Q646	Q00-Q99	Congenital malformations, deformations anc	Urinary	Congenital diverticulum of bladder	0	0	0	1	0	0	1	1
Q647	Q00-Q99	Congenital malformations, deformations anc	Urinary	Other and unspecified congenital malformations of bladder and urethra	0	0	0	1	0	0	1	1
Q6470	Q00-Q99	Congenital malformations, deformations anc	Urinary	Unspecified congenital malformation of bladder and urethra	0	0	0	1	0	0	1	1
Q6471	Q00-Q99	Congenital malformations, deformations anc	Urinary	Congenital prolapse of urethra	0	0	0	1	0	0	1	1
Q6472	Q00-Q99	Congenital malformations, deformations anc	Urinary	Congenital prolapse of urinary meatus	0	0	0	1	0	0	1	1
Q6473	Q00-Q99	Congenital malformations, deformations anc	Urinary	Congenital urethrorectal fistula	0	0	0	1	0	0	1	1
Q6474	Q00-Q99	Congenital malformations, deformations anc	Urinary	Double urethra	0	0	0	1	0	0	1	1
Q6475	Q00-Q99	Congenital malformations, deformations anc	Urinary	Double urinary meatus	0	0	0	1	0	0	1	1
Q6479	Q00-Q99	Congenital malformations, deformations anc	Urinary	Other congenital malformations ofbladder and urethra	0	0	0	1	0	0	1	1
Q648	Q00-Q99	Congenital malformations, deformations anc	Urinary	Other specified congenital malformations of urinary system	0	0	0	1	0	0	1	1
Q649	Q00-Q99	Congenital malformations, deformations anc	Urinary	Congenital malformation of urinarysystem, unspecified	0	0	0	1	0	0	1	1
Q65	Q00-Q99	Congenital malformations, deformations anc	Musculoskeletal	Congenital deformities of hip	0	0	0	1	0	0	1	1
Q650	Q00-Q99	Congenital malformations, deformations anc	Musculoskeletal	Congenital dislocation of hip, unilateral	0	0	0	1	0	0	1	1
Q6500	Q00-Q99	Congenital malformations, deformations anc	Musculoskeletal	Congenital dislocation of unspecified hip, unilateral	0	0	0	1	0	0	1	1
Q6501	Q00-Q99	Congenital malformations, deformations anc	Musculoskeletal	Congenital dislocation of right hip, unilateral	0	0	0	1	0	0	1	1
Q6502	Q00-Q99	Congenital malformations, deformations anc	Musculoskeletal	Congenital dislocation of left hip, unilateral	0	0	0	1	0	0	1	1
Q651	Q00-Q99	Congenital malformations, deformations anc	Musculoskeletal	Congenital dislocation of hip, bilateral	0	0	0	1	0	0	1	1
Q652	Q00-Q99	Congenital malformations, deformations anc	Musculoskeletal	Congenital dislocation of hip, unspecified	0	0	0	1	0	0	1	1
Q653	Q00-Q99	Congenital malformations, deformations anc	Musculoskeletal	Congenital partial dislocation ofhip, unilateral	0	0	0	1	0	0	1	1
Q6530	Q00-Q99	Congenital malformations, deformations anc	Musculoskeletal	Congenital partial dislocation ofunspecified hip, unilateral	0	0	0	1	0	0	1	1
Q6531	Q00-Q99	Congenital malformations, deformations anc	Musculoskeletal	Congenital partial dislocation ofright hip, unilateral	0	0	0	1	0	0	1	1
Q6532	Q00-Q99	Congenital malformations, deformations anc	Musculoskeletal	Congenital partial dislocation ofleft hip, unilateral	0	0	0	1	0	0	1	1
Q654	Q00-Q99	Congenital malformations, deformations anc	Musculoskeletal	Congenital partial dislocation ofhip, bilateral	0	0	0	1	0	0	1	1
Q655	Q00-Q99	Congenital malformations, deformations anc	Musculoskeletal	Congenital partial dislocation ofhip, unspecified	0	0	0	1	0	0	1	1
Q656	Q00-Q99	Congenital malformations, deformations anc	Musculoskeletal	Congenital unstable hip	0	0	0	1	0	0	1	1
Q658	Q00-Q99	Congenital malformations, deformations anc	Musculoskeletal	Other congenital deformities of hip	0	0	0	1	0	0	1	1
Q6581	Q00-Q99	Congenital malformations, deformations anc	Musculoskeletal	Congenital coxa valga	0	0	0	1	0	0	1	1
Q6582	Q00-Q99	Congenital malformations, deformations anc	Musculoskeletal	Congenital coxa vara	0	0	0	1	0	0	1	1
Q659	Q00-Q99	Congenital malformations, deformations anc	Musculoskeletal	Congenital deformity of hip, unspecified	0	0	0	1	0	0	1	1
Q66	Q00-Q99	Congenital malformations, deformations anc	Musculoskeletal	Congenital deformities of feet	0	0	0	1	0	0	1	1
Q660	Q00-Q99	Congenital malformations, deformations anc	Musculoskeletal	Congenital talipes equinovarus	0	0	0	1	0	0	1	1
Q661	Q00-Q99	Congenital malformations, deformations anc	Musculoskeletal	Congenital talipes calcaneovarus	0	0	0	1	0	0	1	1
Q662	Q00-Q99	Congenital malformations, deformations anc	Musculoskeletal	Congenital metatarsus (primus) varus	0	0	0	1	0	0	1	1
Q6621	Q00-Q99	Congenital malformations, deformations anc	Musculoskeletal	Congenital metatarsus primus varus	0	0	0	1	0	0	1	1
Q6622	Q00-Q99	Congenital malformations, deformations anc	Musculoskeletal	Congenital metatarsus adductus	0	0	0	1	0	0	1	1
Q663	Q00-Q99	Congenital malformations, deformations anc	Musculoskeletal	Other congenital varus deformitiesof feet	0	0	0	1	0	0	1	1
Q664	Q00-Q99	Congenital malformations, deformations anc	Musculoskeletal	Congenital talipes calcaneovalgus	0	0	0	1	0	0	1	1
Q665	Q00-Q99	Congenital malformations, deformations anc	Musculoskeletal	Congenital pes planus	0	0	0	1	0	0	1	1
Q6650	Q00-Q99	Congenital malformations, deformations anc	Musculoskeletal	Congenital pes planus, unspecifiedfoot	0	0	0	1	0	0	1	1
Q666	Q00-Q99	Congenital malformations, deformations anc	Musculoskeletal	Other congenital valgus deformities of feet	0	0	0	1	0	0	1	1
Q667	Q00-Q99	Congenital malformations, deformations anc	Musculoskeletal	Congenital pes cavus	0	0	0	1	0	0	1	1
Q668	Q00-Q99	Congenital malformations, deformations anc	Musculoskeletal	Other congenital deformities of feet	0	0	0	1	0	0	1	1
Q6680	Q00-Q99	Congenital malformations, deformations anc	Musculoskeletal	Congenital vertical talus deformity, unspecified foot	0	0	0	1	0	0	1	1
Q6681	Q00-Q99	Congenital malformations, deformations anc	Musculoskeletal	Congenital vertical talus deformity, right foot	0	0	0	1	0	0	1	1
Q6682	Q00-Q99	Congenital malformations, deformations anc	Musculoskeletal	Congenital vertical talus deformity, left foot	0	0	0	1	0	0	1	1
Q6689	Q00-Q99	Congenital malformations, deformations anc	Musculoskeletal	Other specified congenital deformities of feet	0	0	0	1	0	0	1	1
Q669	Q00-Q99	Congenital malformations, deformations anc	Musculoskeletal	Congenital deformity of feet, unspecified	0	0	0	1	0	0	1	1
Q67	Q00-Q99	Congenital malformations, deformations anc	Musculoskeletal	Congenital musculoskeletal deformities of head, face, spine and chest	0	0	0	1	0	0	1	1
Q670	Q00-Q99	Congenital malformations, deformations anc	Musculoskeletal	Congenital facial asymmetry	0	0	0	1	0	0	1	1
Q671	Q00-Q99	Congenital malformations, deformations anc	Musculoskeletal	Congenital compression facies	0	0	0	1	0	0	1	1
Q672	Q00-Q99	Congenital malformations, deformations anc	Musculoskeletal	Dolichocephaly	0	0	0	1	0	0	1	1
Q674	Q00-Q99	Congenital malformations, deformations anc	Musculoskeletal	Other congenital deformities of skull, face and jaw	0	0	0	1	0	0	1	1
Q675	Q00-Q99	Congenital malformations, deformations anc	Musculoskeletal	Congenital deformity of spine	0	0	0	1	0	0	1	1
Q677	Q00-Q99	Congenital malformations, deformations anc	Musculoskeletal	Pectus carinatum	0	0	0	1	0	0	1	1
Q678	Q00-Q99	Congenital malformations, deformations anc	Musculoskeletal	Other congenital deformities of chest	0	0	0	1	0	0	1	1
Q68	Q00-Q99	Congenital malformations, deformations anc	Musculoskeletal	Other congenital musculoskeletal deformities	0	0	0	1	0	0	1	1
Q681	Q00-Q99	Congenital malformations, deformations anc	Musculoskeletal	Congenital deformity of finger(s)and hand	0	0	0	1	0	0	1	1
Q682	Q00-Q99	Congenital malformations, deformations anc	Musculoskeletal	Congenital deformity of knee	0	0	0	1	0	0	1	1
Q683	Q00-Q99	Congenital malformations, deformations anc	Musculoskeletal	Congenital bowing of femur	0	0	0	1	0	0	1	1
Q684	Q00-Q99	Congenital malformations, deformations anc	Musculoskeletal	Congenital bowing of tibia and fibula	0	0	0	1	0	0	1	1
Q685	Q00-Q99	Congenital malformations, deformations anc	Musculoskeletal	Congenital bowing of long bones ofleg, unspecified	0	0	0	1	0	0	1	1
Q688	Q00-Q99	Congenital malformations, deformations anc	Musculoskeletal	Other specified congenital musculoskeletal deformities	0	0	0	1	0	0	1	1
Q69	Q00-Q99	Congenital malformations, deformations anc	Musculoskeletal	Polydactyly	0	0	1	0	0	1	0	1
Q690	Q00-Q99	Congenital malformations, deformations anc	Musculoskeletal	Accessory finger(s)	0	0	1	0	0	1	0	1
Q691	Q00-Q99	Congenital malformations, deformations anc	Musculoskeletal	Accessory thumb(s)	0	0	1	0	0	1	0	1
Q692	Q00-Q99	Congenital malformations, deformations anc	Musculoskeletal	Accessory toe(s)	0	0	1	0	0	1	0	1
Q699	Q00-Q99	Congenital malformations, deformations anc	Musculoskeletal	Polydactyly, unspecified	0	0	1	0	0	1	0	1
Q70	Q00-Q99	Congenital malformations, deformations anc	Musculoskeletal	Syndactyly	0	0	1	0	0	1	0	1
Q700	Q00-Q99	Congenital malformations, deformations anc	Musculoskeletal	Fused fingers	0	0	1	0	0	1	0	1
Q7000	Q00-Q99	Congenital malformations, deformations anc	Musculoskeletal	Fused fingers, unspecified hand	0	0	1	0	0	1	0	1
Q7001	Q00-Q99	Congenital malformations, deformations anc	Musculoskeletal	Fused fingers, right hand	0	0	1	0	0	1	0	1
Q7002	Q00-Q99	Congenital malformations, deformations anc	Musculoskeletal	Fused fingers, left hand	0	0	1	0	0	1	0	1
Q7003	Q00-Q99	Congenital malformations, deformations anc	Musculoskeletal	Fused fingers, bilateral	0	0	1	0	0	1	0	1
Q701	Q00-Q99	Congenital malformations, deformations anc	Musculoskeletal	Webbed fingers	0	0	0	1	0	0	1	1
Q7010	Q00-Q99	Congenital malformations, deformations anc	Musculoskeletal	Webbed fingers, unspecified hand	0	0	0	1	0	0	1	1
Q7011	Q00-Q99	Congenital malformations, deformations anc	Musculoskeletal	Webbed fingers, right hand	0	0	0	1	0	0	1	1
Q7012	Q00-Q99	Congenital malformations, deformations anc	Musculoskeletal	Webbed fingers, left hand	0	0	0	1	0	0	1	1
Q7013	Q00-Q99	Congenital malformations, deformations anc	Musculoskeletal	Webbed fingers, bilateral	0	0	0	1	0	0	1	1
Q702	Q00-Q99	Congenital malformations, deformations anc	Musculoskeletal	Fused toes	0	0	0	1	0	0	1	1
Q7020	Q00-Q99	Congenital malformations, deformations anc	Musculoskeletal	Fused toes, unspecified foot	0	0	0	1	0	0	1	1
Q7021	Q00-Q99	Congenital malformations, deformations anc	Musculoskeletal	Fused toes, right foot	0	0	0	1	0	0	1	1
Q7022	Q00-Q99	Congenital malformations, deformations anc	Musculoskeletal	Fused toes, left foot	0	0	0	1	0	0	1	1
Q7023	Q00-Q99	Congenital malformations, deformations anc	Musculoskeletal	Fused toes, bilateral	0	0	0	1	0	0	1	1
Q703	Q00-Q99	Congenital malformations, deformations anc	Musculoskeletal	Webbed toes	0	0	0	1	0	0	1	1
Q7030	Q00-Q99	Congenital malformations, deformations anc	Musculoskeletal	Webbed toes, unspecified foot	0	0	0	1	0	0	1	1
Q7031	Q00-Q99	Congenital malformations, deformations anc	Musculoskeletal	Webbed toes, right foot	0	0	0	1	0	0	1	1
Q7032	Q00-Q99	Congenital malformations, deformations anc	Musculoskeletal	Webbed toes, left foot	0	0	0	1	0	0	1	1
Q7033	Q00-Q99	Congenital malformations, deformations anc	Musculoskeletal	Webbed toes, bilateral	0	0	0	1	0	0	1	1
Q704	Q00-Q99	Congenital malformations, deformations anc	Musculoskeletal	Polysyndactyly, unspecified	0	0	1	0	0	1	0	1
Q709	Q00-Q99	Congenital malformations, deformations anc	Musculoskeletal	Syndactyly, unspecified	0	0	1	0	0	1	0	1
Q71	Q00-Q99	Congenital malformations, deformations anc	Musculoskeletal	Reduction defects of upper limb	0	0	0	1	0	0	1	1
Q710	Q00-Q99	Congenital malformations, deformations anc	Musculoskeletal	Congenital complete absence of upper limb	0	0	0	1	0	0	1	1
Q7100	Q00-Q99	Congenital malformations, deformations anc	Musculoskeletal	Congenital complete absence of unspecified upper limb	0	0	0	1	0	0	1	1
Q7101	Q00-Q99	Congenital malformations, deformations anc	Musculoskeletal	Congenital complete absence of right upper limb	0	0	0	1	0	0	1	1
Q7102	Q00-Q99	Congenital malformations, deformations anc	Musculoskeletal	Congenital complete absence of left upper limb	0	0	0	1	0	0	1	1
Q7103	Q00-Q99	Congenital malformations, deformations anc	Musculoskeletal	Congenital complete absence of upper limb, bilateral	0	0	1	0	0	1	0	1
Q711	Q00-Q99	Congenital malformations, deformations anc	Musculoskeletal	Congenital absence of upper arm and forearm with hand present	0	0	0	1	0	0	1	1
Q7110	Q00-Q99	Congenital malformations, deformations anc	Musculoskeletal	Congenital absence of unspecifiedupper arm and forearm with hand present	0	0	0	1	0	0	1	1
Q7111	Q00-Q99	Congenital malformations, deformations anc	Musculoskeletal	Congenital absence of right upperarm and forearm with hand present	0	0	0	1	0	0	1	1
Q7112	Q00-Q99	Congenital malformations, deformations anc	Musculoskeletal	Congenital absence of left upper arm and forearm with hand present	0	0	0	1	0	0	1	1
Q7113	Q00-Q99	Congenital malformations, deformations anc	Musculoskeletal	Congenital absence of upper arm and forearm with hand present, bilateral	0	0	1	0	0	1	0	1
Q712	Q00-Q99	Congenital malformations, deformations anc	Musculoskeletal	Congenital absence of both forearmand hand	0	0	0	1	0	0	1	1
Q7120	Q00-Q99	Congenital malformations, deformations anc	Musculoskeletal	Congenital absence of both forearmand hand, unspecified upper limb	0	0	0	1	0	0	1	1
Q7121	Q00-Q99	Congenital malformations, deformations anc	Musculoskeletal	Congenital absence of both forearmand hand, right upper limb	0	0	0	1	0	0	1	1
Q7122	Q00-Q99	Congenital malformations, deformations anc	Musculoskeletal	Congenital absence of both forearmand hand, left upper limb	0	0	0	1	0	0	1	1
Q7123	Q00-Q99	Congenital malformations, deformations anc	Musculoskeletal	Congenital absence of both forearmand hand, bilateral	0	0	0	1	0	0	1	1
Q713	Q00-Q99	Congenital malformations, deformations anc	Musculoskeletal	Congenital absence of hand and finger	0	0	0	1	0	0	1	1
Q7130	Q00-Q99	Congenital malformations, deformations anc	Musculoskeletal	Congenital absence of unspecifiedhand and finger	0	0	0	1	0	0	1	1
Q7131	Q00-Q99	Congenital malformations, deformations anc	Musculoskeletal	Congenital absence of right hand and finger	0	0	0	1	0	0	1	1
Q7132	Q00-Q99	Congenital malformations, deformations anc	Musculoskeletal	Congenital absence of left hand and finger	0	0	0	1	0	0	1	1
Q7133	Q00-Q99	Congenital malformations, deformations anc	Musculoskeletal	Congenital absence of hand and finger, bilateral	0	0	1	0	0	1	0	1
Q714	Q00-Q99	Congenital malformations, deformations anc	Musculoskeletal	Longitudinal reduction defect of radius	0	0	0	1	0	0	1	1
Q7140	Q00-Q99	Congenital malformations, deformations anc	Musculoskeletal	Longitudinal reduction defect of unspecified radius	0	0	0	1	0	0	1	1
Q7141	Q00-Q99	Congenital malformations, deformations anc	Musculoskeletal	Longitudinal reduction defect of right radius	0	0	0	1	0	0	1	1
Q7142	Q00-Q99	Congenital malformations, deformations anc	Musculoskeletal	Longitudinal reduction defect of left radius	0	0	0	1	0	0	1	1
Q7143	Q00-Q99	Congenital malformations, deformations anc	Musculoskeletal	Longitudinal reduction defect of radius, bilateral	0	0	1	0	0	1	0	1
Q715	Q00-Q99	Congenital malformations, deformations anc	Musculoskeletal	Longitudinal reduction defect of ulna	0	0	0	1	0	0	1	1
Q7150	Q00-Q99	Congenital malformations, deformations anc	Musculoskeletal	Longitudinal reduction defect of unspecified ulna	0	0	0	1	0	0	1	1
Q7151	Q00-Q99	Congenital malformations, deformations anc	Musculoskeletal	Longitudinal reduction defect of right ulna	0	0	0	1	0	0	1	1
Q7152	Q00-Q99	Congenital malformations, deformations anc	Musculoskeletal	Longitudinal reduction defect of left ulna	0	0	0	1	0	0	1	1
Q7153	Q00-Q99	Congenital malformations, deformations anc	Musculoskeletal	Longitudinal reduction defect of ulna, bilateral	0	0	1	0	0	1	0	1
Q716	Q00-Q99	Congenital malformations, deformations anc	Musculoskeletal	Lobster-claw hand	0	0	1	0	0	1	0	1
Q7160	Q00-Q99	Congenital malformations, deformations anc	Musculoskeletal	Lobster-claw hand, unspecified hand	0	0	1	0	0	1	0	1
Q7161	Q00-Q99	Congenital malformations, deformations anc	Musculoskeletal	Lobster-claw right hand	0	0	1	0	0	1	0	1
Q7162	Q00-Q99	Congenital malformations, deformations anc	Musculoskeletal	Lobster-claw left hand	0	0	1	0	0	1	0	1
Q7163	Q00-Q99	Congenital malformations, deformations anc	Musculoskeletal	Lobster-claw hand, bilateral	0	0	1	0	0	1	0	1
Q718	Q00-Q99	Congenital malformations, deformations anc	Musculoskeletal	Other reduction defects of upper limb	0	0	0	1	0	0	1	1
Q7181	Q00-Q99	Congenital malformations, deformations anc	Musculoskeletal	Congenital shortening of upper limb	0	0	0	1	0	0	1	1
Q71811	Q00-Q99	Congenital malformations, deformations anc	Musculoskeletal	Congenital shortening of right upper limb	0	0	0	1	0	0	1	1
Q71812	Q00-Q99	Congenital malformations, deformations anc	Musculoskeletal	Congenital shortening of left upper limb	0	0	0	1	0	0	1	1
Q71813	Q00-Q99	Congenital malformations, deformations anc	Musculoskeletal	Congenital shortening of upper limb, bilateral	0	0	0	1	0	0	1	1
Q71819	Q00-Q99	Congenital malformations, deformations anc	Musculoskeletal	Congenital shortening of unspecified upper limb	0	0	0	1	0	0	1	1
Q7189	Q00-Q99	Congenital malformations, deformations anc	Musculoskeletal	Other reduction defects of upper limb	0	0	0	1	0	0	1	1
Q71891	Q00-Q99	Congenital malformations, deformations anc	Musculoskeletal	Other reduction defects of right upper limb	0	0	0	1	0	0	1	1
Q71892	Q00-Q99	Congenital malformations, deformations anc	Musculoskeletal	Other reduction defects of left upper limb	0	0	0	1	0	0	1	1
Q71893	Q00-Q99	Congenital malformations, deformations anc	Musculoskeletal	Other reduction defects of upper limb, bilateral	0	0	0	1	0	0	1	1
Q71899	Q00-Q99	Congenital malformations, deformations anc	Musculoskeletal	Other reduction defects of unspecified upper limb	0	0	0	1	0	0	1	1
Q719	Q00-Q99	Congenital malformations, deformations anc	Musculoskeletal	Unspecified reduction defect of upper limb	0	0	0	1	0	0	1	1
Q7190	Q00-Q99	Congenital malformations, deformations anc	Musculoskeletal	Unspecified reduction defect of unspecified upper limb	0	0	0	1	0	0	1	1
Q7191	Q00-Q99	Congenital malformations, deformations anc	Musculoskeletal	Unspecified reduction defect of right upper limb	0	0	0	1	0	0	1	1
Q7192	Q00-Q99	Congenital malformations, deformations anc	Musculoskeletal	Unspecified reduction defect of left upper limb	0	0	0	1	0	0	1	1
Q7193	Q00-Q99	Congenital malformations, deformations anc	Musculoskeletal	Unspecified reduction defect of upper limb, bilateral	0	0	0	1	0	0	1	1
Q72	Q00-Q99	Congenital malformations, deformations anc	Musculoskeletal	Reduction defects of lower limb	0	0	0	1	0	0	1	1
Q720	Q00-Q99	Congenital malformations, deformations anc	Musculoskeletal	Congenital complete absence of lower limb	0	0	0	1	0	0	1	1
Q7200	Q00-Q99	Congenital malformations, deformations anc	Musculoskeletal	Congenital complete absence of unspecified lower limb	0	0	0	1	0	0	1	1
Q7201	Q00-Q99	Congenital malformations, deformations anc	Musculoskeletal	Congenital complete absence of right lower limb	0	0	0	1	0	0	1	1
Q7202	Q00-Q99	Congenital malformations, deformations anc	Musculoskeletal	Congenital complete absence of left lower limb	0	0	0	1	0	0	1	1
Q7203	Q00-Q99	Congenital malformations, deformations anc	Musculoskeletal	Congenital complete absence of lower limb, bilateral	0	0	0	1	0	0	1	1
Q721	Q00-Q99	Congenital malformations, deformations anc	Musculoskeletal	Congenital absence of thigh and lower leg with foot present	0	0	0	1	0	0	1	1
Q7210	Q00-Q99	Congenital malformations, deformations anc	Musculoskeletal	Congenital absence of unspecifiedthigh and lower leg with foot present	0	0	0	1	0	0	1	1
Q7211	Q00-Q99	Congenital malformations, deformations anc	Musculoskeletal	Congenital absence of right thighand lower leg with foot present	0	0	0	1	0	0	1	1
Q7212	Q00-Q99	Congenital malformations, deformations anc	Musculoskeletal	Congenital absence of left thigh and lower leg with foot present	0	0	0	1	0	0	1	1
Q7213	Q00-Q99	Congenital malformations, deformations anc	Musculoskeletal	Congenital absence of thigh and lower leg with foot present, bilateral	0	0	0	1	0	0	1	1
Q722	Q00-Q99	Congenital malformations, deformations anc	Musculoskeletal	Congenital absence of both lower leg and foot	0	0	0	1	0	0	1	1
Q7220	Q00-Q99	Congenital malformations, deformations anc	Musculoskeletal	Congenital absence of both lower leg and foot, unspecified lower limb	0	0	0	1	0	0	1	1
Q7221	Q00-Q99	Congenital malformations, deformations anc	Musculoskeletal	Congenital absence of both lower leg and foot, right lower limb	0	0	0	1	0	0	1	1
Q7222	Q00-Q99	Congenital malformations, deformations anc	Musculoskeletal	Congenital absence of both lower leg and foot, left lower limb	0	0	0	1	0	0	1	1
Q7223	Q00-Q99	Congenital malformations, deformations anc	Musculoskeletal	Congenital absence of both lower leg and foot, bilateral	0	0	0	1	0	0	1	1
Q723	Q00-Q99	Congenital malformations, deformations anc	Musculoskeletal	Congenital absence of foot and toe(s)	0	0	0	1	0	0	1	1
Q7230	Q00-Q99	Congenital malformations, deformations anc	Musculoskeletal	Congenital absence of unspecifiedfoot and toe(s)	0	0	0	1	0	0	1	1
Q7231	Q00-Q99	Congenital malformations, deformations anc	Musculoskeletal	Congenital absence of right foot and toe(s)	0	0	0	1	0	0	1	1
Q7232	Q00-Q99	Congenital malformations, deformations anc	Musculoskeletal	Congenital absence of left foot and toe(s)	0	0	0	1	0	0	1	1
Q7233	Q00-Q99	Congenital malformations, deformations anc	Musculoskeletal	Congenital absence of foot and toe(s), bilateral	0	0	0	1	0	0	1	1
Q724	Q00-Q99	Congenital malformations, deformations anc	Musculoskeletal	Longitudinal reduction defect of femur	0	0	0	1	0	0	1	1
Q7240	Q00-Q99	Congenital malformations, deformations anc	Musculoskeletal	Longitudinal reduction defect of unspecified femur	0	0	0	1	0	0	1	1
Q7241	Q00-Q99	Congenital malformations, deformations anc	Musculoskeletal	Longitudinal reduction defect of right femur	0	0	0	1	0	0	1	1
Q7242	Q00-Q99	Congenital malformations, deformations anc	Musculoskeletal	Longitudinal reduction defect of left femur	0	0	0	1	0	0	1	1
Q7243	Q00-Q99	Congenital malformations, deformations anc	Musculoskeletal	Longitudinal reduction defect of femur, bilateral	0	0	0	1	0	0	1	1
Q725	Q00-Q99	Congenital malformations, deformations anc	Musculoskeletal	Longitudinal reduction defect of tibia	0	0	0	1	0	0	1	1
Q7250	Q00-Q99	Congenital malformations, deformations anc	Musculoskeletal	Longitudinal reduction defect of unspecified tibia	0	0	0	1	0	0	1	1
Q7251	Q00-Q99	Congenital malformations, deformations anc	Musculoskeletal	Longitudinal reduction defect of right tibia	0	0	0	1	0	0	1	1
Q7252	Q00-Q99	Congenital malformations, deformations anc	Musculoskeletal	Longitudinal reduction defect of left tibia	0	0	0	1	0	0	1	1
Q7253	Q00-Q99	Congenital malformations, deformations anc	Musculoskeletal	Longitudinal reduction defect of tibia, bilateral	0	0	0	1	0	0	1	1
Q726	Q00-Q99	Congenital malformations, deformations anc	Musculoskeletal	Longitudinal reduction defect of fibula	0	0	0	1	0	0	1	1
Q7260	Q00-Q99	Congenital malformations, deformations anc	Musculoskeletal	Longitudinal reduction defect of unspecified fibula	0	0	0	1	0	0	1	1
Q7261	Q00-Q99	Congenital malformations, deformations anc	Musculoskeletal	Longitudinal reduction defect of right fibula	0	0	0	1	0	0	1	1
Q7262	Q00-Q99	Congenital malformations, deformations anc	Musculoskeletal	Longitudinal reduction defect of left fibula	0	0	0	1	0	0	1	1
Q7263	Q00-Q99	Congenital malformations, deformations anc	Musculoskeletal	Longitudinal reduction defect of fibula, bilateral	0	0	0	1	0	0	1	1
Q727	Q00-Q99	Congenital malformations, deformations anc	Musculoskeletal	Split foot	0	0	1	0	0	1	0	1
Q7270	Q00-Q99	Congenital malformations, deformations anc	Musculoskeletal	Split foot, unspecified lower limb	0	0	1	0	0	1	0	1

Q7271	Q00-Q99	Congenital malformations, deformations anc	Musculoskeletal	Split foot, right lower limb	0	0	1	0	0	1	0	1
Q7272	Q00-Q99	Congenital malformations, deformations anc	Musculoskeletal	Split foot, left lower limb	0	0	1	0	0	1	0	1
Q7273	Q00-Q99	Congenital malformations, deformations anc	Musculoskeletal	Split foot, bilateral	0	0	1	0	0	1	0	1
Q728	Q00-Q99	Congenital malformations, deformations anc	Musculoskeletal	Other reduction defects of lower limb	0	0	0	1	0	0	1	1
Q7281	Q00-Q99	Congenital malformations, deformations anc	Musculoskeletal	Congenital shortening of lower limb	0	0	0	1	0	0	1	1
Q72811	Q00-Q99	Congenital malformations, deformations anc	Musculoskeletal	Congenital shortening of right lower limb	0	0	0	1	0	0	1	1
Q72812	Q00-Q99	Congenital malformations, deformations anc	Musculoskeletal	Congenital shortening of left lower limb	0	0	0	1	0	0	1	1
Q72813	Q00-Q99	Congenital malformations, deformations anc	Musculoskeletal	Congenital shortening of lower limb, bilateral	0	0	0	1	0	0	1	1
Q72819	Q00-Q99	Congenital malformations, deformations anc	Musculoskeletal	Congenital shortening of unspecified lower limb	0	0	0	1	0	0	1	1
Q7289	Q00-Q99	Congenital malformations, deformations anc	Musculoskeletal	Other reduction defects of lower limb	0	0	0	1	0	0	1	1
Q72891	Q00-Q99	Congenital malformations, deformations anc	Musculoskeletal	Other reduction defects of right lower limb	0	0	0	1	0	0	1	1
Q72892	Q00-Q99	Congenital malformations, deformations anc	Musculoskeletal	Other reduction defects of left lower limb	0	0	0	1	0	0	1	1
Q72893	Q00-Q99	Congenital malformations, deformations anc	Musculoskeletal	Other reduction defects of lower limb, bilateral	0	0	0	1	0	0	1	1
Q72899	Q00-Q99	Congenital malformations, deformations anc	Musculoskeletal	Other reduction defects of unspecified lower limb	0	0	0	1	0	0	1	1
Q729	Q00-Q99	Congenital malformations, deformations anc	Musculoskeletal	Unspecified reduction defect of lower limb	0	0	0	1	0	0	1	1
Q7290	Q00-Q99	Congenital malformations, deformations anc	Musculoskeletal	Unspecified reduction defect of unspecified lower limb	0	0	0	1	0	0	1	1
Q7291	Q00-Q99	Congenital malformations, deformations anc	Musculoskeletal	Unspecified reduction defect of right lower limb	0	0	0	1	0	0	1	1
Q7292	Q00-Q99	Congenital malformations, deformations anc	Musculoskeletal	Unspecified reduction defect of left lower limb	0	0	0	1	0	0	1	1
Q7293	Q00-Q99	Congenital malformations, deformations anc	Musculoskeletal	Unspecified reduction defect of lower limb, bilateral	0	0	0	1	0	0	1	1
Q73	Q00-Q99	Congenital malformations, deformations anc	Musculoskeletal	Reduction defects of unspecified limb	0	0	0	1	0	0	1	1
Q730	Q00-Q99	Congenital malformations, deformations anc	Musculoskeletal	Congenital absence of unspecifiedlimb(s)	0	0	0	1	0	0	1	1
Q731	Q00-Q99	Congenital malformations, deformations anc	Musculoskeletal	Phocomelia, unspecified limb(s)	0	0	0	1	0	0	1	1
Q738	Q00-Q99	Congenital malformations, deformations anc	Musculoskeletal	Other reduction defects of unspecified limb(s)	0	0	0	1	0	0	1	1
Q74	Q00-Q99	Congenital malformations, deformations anc	Musculoskeletal	Other congenital malformations oflimb(s)	0	0	0	1	0	0	1	1
Q740	Q00-Q99	Congenital malformations, deformations anc	Musculoskeletal	Other congenital malformations ofupper limb(s), including shoulder girdle	0	0	0	1	0	0	1	1
Q741	Q00-Q99	Congenital malformations, deformations anc	Musculoskeletal	Congenital malformation of knee	0	0	0	1	0	0	1	1
Q742	Q00-Q99	Congenital malformations, deformations anc	Musculoskeletal	Other congenital malformations oflower limb(s), including pelvic girdle	0	0	0	1	0	0	1	1
Q743	Q00-Q99	Congenital malformations, deformations anc	Musculoskeletal	Arthrogryposis multiplex congenita	0	0	0	1	0	0	1	1
Q748	Q00-Q99	Congenital malformations, deformations anc	Musculoskeletal	Other specified congenital malformations of limb(s)	0	0	0	1	0	0	1	1
Q749	Q00-Q99	Congenital malformations, deformations anc	Musculoskeletal	Unspecified congenital malformation of limb(s)	0	0	0	1	0	0	1	1
Q75	Q00-Q99	Congenital malformations, deformations anc	Musculoskeletal	Other congenital malformations ofskull and face bones	0	0	0	1	0	0	1	1
Q750	Q00-Q99	Congenital malformations, deformations anc	Musculoskeletal	Craniosynostosis	0	0	1	0	0	1	0	1
Q751	Q00-Q99	Congenital malformations, deformations anc	Musculoskeletal	Craniofacial dysostosis	0	0	1	0	0	1	0	1
Q752	Q00-Q99	Congenital malformations, deformations anc	Musculoskeletal	Hypertelorism	0	0	0	1	0	0	1	1
Q754	Q00-Q99	Congenital malformations, deformations anc	Musculoskeletal	Mandibulofacial dysostosis	0	1	0	0	1	0	0	1
Q755	Q00-Q99	Congenital malformations, deformations anc	Musculoskeletal	Oculomandibular dysostosis	0	1	0	0	1	0	0	1
Q758	Q00-Q99	Congenital malformations, deformations anc	Musculoskeletal	Other specified congenital malformations of skull and face bones	0	0	0	1	0	0	1	1
Q759	Q00-Q99	Congenital malformations, deformations anc	Musculoskeletal	Congenital malformation of skull and face bones, unspecified	0	0	0	1	0	0	1	1
Q76	Q00-Q99	Congenital malformations, deformations anc	Musculoskeletal	Congenital malformations of spineand bony thorax	0	0	0	1	0	0	1	1
Q760	Q00-Q99	Congenital malformations, deformations anc	Musculoskeletal	Spina bifida occulta	0	0	0	1	0	0	1	1
Q761	Q00-Q99	Congenital malformations, deformations anc	Musculoskeletal	Klippel-Feil syndrome	0	0	1	0	0	1	0	1
Q762	Q00-Q99	Congenital malformations, deformations anc	Musculoskeletal	Congenital spondylolisthesis	0	0	0	1	0	0	1	1
Q763	Q00-Q99	Congenital malformations, deformations anc	Musculoskeletal	Congenital scoliosis due to congenital bony malformation	0	0	0	1	0	0	1	1
Q764	Q00-Q99	Congenital malformations, deformations anc	Musculoskeletal	Other congenital malformations ofspine, not associated with scoliosis	0	0	0	1	0	0	1	1
Q7641	Q00-Q99	Congenital malformations, deformations anc	Musculoskeletal	Congenital kyphosis	0	0	0	1	0	0	1	1
Q76411	Q00-Q99	Congenital malformations, deformations anc	Musculoskeletal	Congenital kyphosis, occipito-atlanto-axial region	0	0	0	1	0	0	1	1
Q76412	Q00-Q99	Congenital malformations, deformations anc	Musculoskeletal	Congenital kyphosis, cervical region	0	0	0	1	0	0	1	1
Q76413	Q00-Q99	Congenital malformations, deformations anc	Musculoskeletal	Congenital kyphosis, cervicothoracic region	0	0	0	1	0	0	1	1
Q76414	Q00-Q99	Congenital malformations, deformations anc	Musculoskeletal	Congenital kyphosis, thoracic region	0	0	0	1	0	0	1	1
Q76415	Q00-Q99	Congenital malformations, deformations anc	Musculoskeletal	Congenital kyphosis, thoracolumbarregion	0	0	0	1	0	0	1	1
Q76419	Q00-Q99	Congenital malformations, deformations anc	Musculoskeletal	Congenital kyphosis, unspecified region	0	0	0	1	0	0	1	1
Q7642	Q00-Q99	Congenital malformations, deformations anc	Musculoskeletal	Congenital lordosis	0	0	0	1	0	0	1	1
Q76425	Q00-Q99	Congenital malformations, deformations anc	Musculoskeletal	Congenital lordosis, thoracolumbarregion	0	0	0	1	0	0	1	1
Q76426	Q00-Q99	Congenital malformations, deformations anc	Musculoskeletal	Congenital lordosis, lumbar region	0	0	0	1	0	0	1	1
Q76427	Q00-Q99	Congenital malformations, deformations anc	Musculoskeletal	Congenital lordosis, lumbosacral region	0	0	0	1	0	0	1	1
Q76428	Q00-Q99	Congenital malformations, deformations anc	Musculoskeletal	Congenital lordosis, sacral and sacrococcygeal region	0	0	0	1	0	0	1	1
Q76429	Q00-Q99	Congenital malformations, deformations anc	Musculoskeletal	Congenital lordosis, unspecified region	0	0	0	1	0	0	1	1
Q7649	Q00-Q99	Congenital malformations, deformations anc	Musculoskeletal	Other congenital malformations ofspine, not associated with scoliosis	0	0	0	1	0	0	1	1
Q765	Q00-Q99	Congenital malformations, deformations anc	Musculoskeletal	Cervical rib	0	0	0	1	0	0	1	1
Q766	Q00-Q99	Congenital malformations, deformations anc	Musculoskeletal	Other congenital malformations ofribs	0	0	0	1	0	0	1	1
Q767	Q00-Q99	Congenital malformations, deformations anc	Musculoskeletal	Congenital malformation of sternum	0	0	0	1	0	0	1	1
Q768	Q00-Q99	Congenital malformations, deformations anc	Musculoskeletal	Other congenital malformations ofbony thorax	0	0	0	1	0	0	1	1
Q769	Q00-Q99	Congenital malformations, deformations anc	Musculoskeletal	Congenital malformation of bony thorax, unspecified	0	0	0	1	0	0	1	1
Q77	Q00-Q99	Congenital malformations, deformations anc	Musculoskeletal	Osteochondrodysplasia with defectsof growth of tubular bones and spine	0	0	1	0	0	1	0	1
Q770	Q00-Q99	Congenital malformations, deformations anc	Musculoskeletal	Achondrogenesis	0	1	0	0	1	0	0	1
Q771	Q00-Q99	Congenital malformations, deformations anc	Musculoskeletal	Thanatophoric short stature	1	1	0	0	1	0	0	1
Q772	Q00-Q99	Congenital malformations, deformations anc	Musculoskeletal	Short rib syndrome	0	1	0	0	1	0	0	1
Q773	Q00-Q99	Congenital malformations, deformations anc	Musculoskeletal	Chondrodysplasia punctata	1	1	0	0	1	0	0	1
Q774	Q00-Q99	Congenital malformations, deformations anc	Musculoskeletal	Achondroplasia	1	1	0	0	1	0	0	1
Q775	Q00-Q99	Congenital malformations, deformations anc	Musculoskeletal	Diastrophic dysplasia	0	1	0	0	1	0	0	1
Q776	Q00-Q99	Congenital malformations, deformations anc	Musculoskeletal	Chondroectodermal dysplasia	0	1	0	0	1	0	0	1
Q777	Q00-Q99	Congenital malformations, deformations anc	Musculoskeletal	Spondyloepiphyseal dysplasia	0	1	0	0	1	0	0	1
Q778	Q00-Q99	Congenital malformations, deformations anc	Musculoskeletal	Other osteochondrodysplasia with defects of growth of tubular bones and spine	0	0	1	0	0	1	0	1
Q779	Q00-Q99	Congenital malformations, deformations anc	Musculoskeletal	Osteochondrodysplasia with defectsof growth of tubular bones and spine, unspecified	0	0	1	0	0	1	0	1
Q78	Q00-Q99	Congenital malformations, deformations anc	Musculoskeletal	Other osteochondrodysplasias	0	0	1	0	0	1	0	1
Q780	Q00-Q99	Congenital malformations, deformations anc	Musculoskeletal	Osteogenesis imperfecta	1	1	0	0	1	0	0	1
Q781	Q00-Q99	Congenital malformations, deformations anc	Musculoskeletal	Polyostotic fibrous dysplasia	0	0	1	0	0	1	0	1
Q782	Q00-Q99	Congenital malformations, deformations anc	Musculoskeletal	Osteopetrosis	0	1	0	0	1	0	0	1
Q783	Q00-Q99	Congenital malformations, deformations anc	Musculoskeletal	Progressive diaphyseal dysplasia	0	0	1	0	0	1	0	1
Q784	Q00-Q99	Congenital malformations, deformations anc	Musculoskeletal	Enchondromatosis	0	1	0	0	1	0	0	1
Q785	Q00-Q99	Congenital malformations, deformations anc	Musculoskeletal	Metaphyseal dysplasia	0	1	0	0	1	0	0	1
Q786	Q00-Q99	Congenital malformations, deformations anc	Musculoskeletal	Multiple congenital exostoses	1	1	0	0	1	0	0	1
Q788	Q00-Q99	Congenital malformations, deformations anc	Musculoskeletal	Other specified osteochondrodysplasias	0	0	1	0	0	1	0	1
Q789	Q00-Q99	Congenital malformations, deformations anc	Musculoskeletal	Osteochondrodysplasia, unspecified	0	0	1	0	0	1	0	1
Q79	Q00-Q99	Congenital malformations, deformations anc	Musculoskeletal	Congenital malformations of musculoskeletal system, not elsewhere classified	0	0	0	1	0	0	1	1
Q790	Q00-Q99	Congenital malformations, deformations anc	Musculoskeletal	Congenital diaphragmatic hernia	0	0	0	1	0	0	1	1
Q791	Q00-Q99	Congenital malformations, deformations anc	Musculoskeletal	Other congenital malformations ofdiaphragm	0	0	0	1	0	0	1	1
Q792	Q00-Q99	Congenital malformations, deformations anc	Musculoskeletal	Exomphalos	0	0	0	1	0	0	1	1
Q793	Q00-Q99	Congenital malformations, deformations anc	Musculoskeletal	Gastroschisis	0	0	0	1	0	0	1	1
Q794	Q00-Q99	Congenital malformations, deformations anc	Musculoskeletal	Prune belly syndrome	0	0	0	1	0	0	1	1
Q795	Q00-Q99	Congenital malformations, deformations anc	Musculoskeletal	Other congenital malformations ofabdominal wall	0	0	0	1	0	0	1	1
Q7951	Q00-Q99	Congenital malformations, deformations anc	Musculoskeletal	Congenital hernia of bladder	0	0	0	1	0	0	1	1
Q7959	Q00-Q99	Congenital malformations, deformations anc	Musculoskeletal	Other congenital malformations ofabdominal wall	0	0	0	1	0	0	1	1
Q796	Q00-Q99	Congenital malformations, deformations anc	Musculoskeletal	Ehlers-Danlos syndrome	1	1	0	0	1	0	0	1
Q798	Q00-Q99	Congenital malformations, deformations anc	Musculoskeletal	Other congenital malformations ofmusculoskeletal system	0	0	0	1	0	0	1	1
Q799	Q00-Q99	Congenital malformations, deformations anc	Musculoskeletal	Congenital malformation of musculoskeletal system, unspecified	0	0	0	1	0	0	1	1
Q80	Q00-Q99	Congenital malformations, deformations anc	Other	Congenital ichthyosis	0	1	0	0	1	0	0	1
Q800	Q00-Q99	Congenital malformations, deformations anc	Other	Ichthyosis vulgaris	1	1	0	0	1	0	0	1
Q801	Q00-Q99	Congenital malformations, deformations anc	Other	X-linked ichthyosis	1	1	0	0	1	0	0	1
Q802	Q00-Q99	Congenital malformations, deformations anc	Other	Lamellar ichthyosis	1	1	0	0	1	0	0	1
Q803	Q00-Q99	Congenital malformations, deformations anc	Other	Congenital bullous ichthyosiform erythroderma	0	1	0	0	1	0	0	1
Q804	Q00-Q99	Congenital malformations, deformations anc	Other	Harlequin fetus	1	1	0	0	1	0	0	1
Q808	Q00-Q99	Congenital malformations, deformations anc	Other	Other congenital ichthyosis	0	1	0	0	1	0	0	1
Q809	Q00-Q99	Congenital malformations, deformations anc	Other	Congenital ichthyosis, unspecified	0	1	0	0	1	0	0	1
Q81	Q00-Q99	Congenital malformations, deformations anc	Other	Epidermolysis bullosa	0	1	0	0	1	0	0	1
Q810	Q00-Q99	Congenital malformations, deformations anc	Other	Epidermolysis bullosa simplex	0	1	0	0	1	0	0	1
Q811	Q00-Q99	Congenital malformations, deformations anc	Other	Epidermolysis bullosa letalis	0	1	0	0	1	0	0	1
Q812	Q00-Q99	Congenital malformations, deformations anc	Other	Epidermolysis bullosa dystrophica	0	1	0	0	1	0	0	1
Q818	Q00-Q99	Congenital malformations, deformations anc	Other	Other epidermolysis bullosa	0	1	0	0	1	0	0	1
Q819	Q00-Q99	Congenital malformations, deformations anc	Other	Epidermolysis bullosa, unspecified	0	1	0	0	1	0	0	1
Q82	Q00-Q99	Congenital malformations, deformations anc	Other	Other congenital malformations ofskin	0	0	0	1	0	0	1	1
Q820	Q00-Q99	Congenital malformations, deformations anc	Other	Hereditary lymphedema	0	1	0	0	1	0	0	1
Q821	Q00-Q99	Congenital malformations, deformations anc	Other	Xeroderma pigmentosum	1	1	0	0	1	0	0	1
Q822	Q00-Q99	Congenital malformations, deformations anc	Other	Mastocytosis	0	0	0	1	0	0	1	1
Q823	Q00-Q99	Congenital malformations, deformations anc	Other	Incontinentia pigmenti	1	1	0	0	1	0	0	1
Q824	Q00-Q99	Congenital malformations, deformations anc	Other	Ectodermal dysplasia (anhidrotic)	1	1	0	0	1	0	0	1
Q829	Q00-Q99	Congenital malformations, deformations anc	Other	Congenital malformation of skin, unspecified	0	0	0	1	0	0	1	1
Q83	Q00-Q99	Congenital malformations, deformations anc	Other	Congenital malformations of breast	0	0	0	1	0	0	1	1
Q830	Q00-Q99	Congenital malformations, deformations anc	Other	Congenital absence of breast withabsent nipple	0	0	0	1	0	0	1	1
Q831	Q00-Q99	Congenital malformations, deformations anc	Other	Accessory breast	0	0	0	1	0	0	1	1
Q832	Q00-Q99	Congenital malformations, deformations anc	Other	Absent nipple	0	0	0	1	0	0	1	1
Q838	Q00-Q99	Congenital malformations, deformations anc	Other	Other congenital malformations ofbreast	0	0	0	1	0	0	1	1
Q839	Q00-Q99	Congenital malformations, deformations anc	Other	Congenital malformation of breast,unspecified	0	0	0	1	0	0	1	1
Q84	Q00-Q99	Congenital malformations, deformations anc	Other	Other congenital malformations ofintegument	0	0	0	1	0	0	1	1
Q840	Q00-Q99	Congenital malformations, deformations anc	Other	Congenital alopecia	0	0	1	0	0	1	0	1
Q841	Q00-Q99	Congenital malformations, deformations anc	Other	Congenital morphological disturbances of hair, not elsewhere classified	0	0	1	0	0	1	0	1
Q842	Q00-Q99	Congenital malformations, deformations anc	Other	Other congenital malformations ofhair	0	0	1	0	0	1	0	1
Q843	Q00-Q99	Congenital malformations, deformations anc	Other	Anonychia	0	0	1	0	0	1	0	1
Q844	Q00-Q99	Congenital malformations, deformations anc	Other	Congenital leukonychia	0	0	1	0	0	1	0	1
Q845	Q00-Q99	Congenital malformations, deformations anc	Other	Enlarged and hypertrophic nails	0	0	0	1	0	0	1	1
Q846	Q00-Q99	Congenital malformations, deformations anc	Other	Other congenital malformations ofnails	0	0	0	1	0	0	1	1
Q848	Q00-Q99	Congenital malformations, deformations anc	Other	Other specified congenital malformations of integument	0	0	0	1	0	0	1	1
Q849	Q00-Q99	Congenital malformations, deformations anc	Other	Congenital malformation of integument, unspecified	0	0	0	1	0	0	1	1
Q85	Q00-Q99	Congenital malformations, deformations anc	Other	Phakomatoses, not elsewhere classified	0	0	1	0	0	1	0	1
Q850	Q00-Q99	Congenital malformations, deformations anc	Other	Neurofibromatosis (nonmalignant)	1	1	0	0	1	0	0	1
Q8500	Q00-Q99	Congenital malformations, deformations anc	Other	Neurofibromatosis, unspecified	1	1	0	0	1	0	0	1
Q8501	Q00-Q99	Congenital malformations, deformations anc	Other	Neurofibromatosis, type 1	1	1	0	0	1	0	0	1
Q8502	Q00-Q99	Congenital malformations, deformations anc	Other	Neurofibromatosis, type 2	1	1	0	0	1	0	0	1
Q8503	Q00-Q99	Congenital malformations, deformations anc	Other	Schwannomatosis	0	0	1	0	0	1	0	1
Q8509	Q00-Q99	Congenital malformations, deformations anc	Other	Other neurofibromatosis	1	1	0	0	1	0	0	1
Q851	Q00-Q99	Congenital malformations, deformations anc	Other	Tuberous sclerosis	1	1	0	0	1	0	0	1
Q858	Q00-Q99	Congenital malformations, deformations anc	Other	Other phakomatoses, not elsewhereclassified	0	0	1	0	0	1	0	1
Q859	Q00-Q99	Congenital malformations, deformations anc	Other	Phakomatosis, unspecified	0	0	1	0	0	1	0	1
Q86	Q00-Q99	Congenital malformations, deformations anc	Other	Congenital malformation syndromesdue to known exogenous causes, not elsewhere c	0	0	0	1	0	0	1	1
Q87	Q00-Q99	Congenital malformations, deformations anc	Multiple	Other specified congenital malformation syndromes affecting multiple systems	0	0	0	1	0	0	1	1
Q870	Q00-Q99	Congenital malformations, deformations anc	Multiple	Congenital malformation syndromespredominantly affecting facial appearance	0	0	0	1	0	0	1	1
Q871	Q00-Q99	Congenital malformations, deformations anc	Multiple	Congenital malformation syndromespredominantly associated with short stature	0	0	0	1	0	0	1	1
Q872	Q00-Q99	Congenital malformations, deformations anc	Multiple	Congenital malformation syndromespredominantly involving limbs	0	0	0	1	0	0	1	1
Q873	Q00-Q99	Congenital malformations, deformations anc	Multiple	Congenital malformation syndromesinvolving early overgrowth	0	0	0	1	0	0	1	1
Q874	Q00-Q99	Congenital malformations, deformations anc	Multiple	Marfan's syndrome	1	1	0	0	1	0	0	1
Q8740	Q00-Q99	Congenital malformations, deformations anc	Multiple	Marfan's syndrome, unspecified	1	1	0	0	1	0	0	1
Q8741	Q00-Q99	Congenital malformations, deformations anc	Multiple	Marfan's syndrome with cardiovascular manifestations	1	1	0	0	1	0	0	1
Q87410	Q00-Q99	Congenital malformations, deformations anc	Multiple	Marfan's syndrome with aortic dilation	1	1	0	0	1	0	0	1
Q87418	Q00-Q99	Congenital malformations, deformations anc	Multiple	Marfan's syndrome with other cardiovascular manifestations	1	1	0	0	1	0	0	1
Q8742	Q00-Q99	Congenital malformations, deformations anc	Multiple	Marfan's syndrome with ocular manifestations	1	1	0	0	1	0	0	1
Q8743	Q00-Q99	Congenital malformations, deformations anc	Multiple	Marfan's syndrome with skeletal manifestation	1	1	0	0	1	0	0	1
Q875	Q00-Q99	Congenital malformations, deformations anc	Multiple	Other congenital malformation syndromes with other skeletal changes	0	0	0	1	0	0	1	1
Q878	Q00-Q99	Congenital malformations, deformations anc	Multiple	Other specified congenital malformation syndromes, not elsewhere classified	0	0	0	1	0	0	1	1
Q8781	Q00-Q99	Congenital malformations, deformations anc	Multiple	Alport syndrome	1	1	0	0	1	0	0	1
Q8782	Q00-Q99	Congenital malformations, deformations anc	Multiple	Arterial tortuosity syndrome	1	1	0	0	1	0	0	1
Q8789	Q00-Q99	Congenital malformations, deformations anc	Multiple	Other specified congenital malformation syndromes, not elsewhere classified	0	0	0	1	0	0	1	1
Q89	Q00-Q99	Congenital malformations, deformations anc	Other	Other congenital malformations, not elsewhere classified	0	0	0	1	0	0	1	1
Q890	Q00-Q99	Congenital malformations, deformations anc	Other	Congenital absence and malformations of spleen	0	0	0	1	0	0	1	1
Q8901	Q00-Q99	Congenital malformations, deformations anc	Other	Asplenia (congenital)	0	0	0	1	0	0	1	1
Q8909	Q00-Q99	Congenital malformations, deformations anc	Other	Congenital malformations of spleen	0	0	0	1	0	0	1	1
Q891	Q00-Q99	Congenital malformations, deformations anc	Other	Congenital malformations of adrenal gland	0	0	0	1	0	0	1	1
Q892	Q00-Q99	Congenital malformations, deformations anc	Other	Congenital malformations of otherendocrine glands	0	0	0	1	0	0	1	1
Q893	Q00-Q99	Congenital malformations, deformations anc	Other	Situs inversus	0	0	0	1	0	0	1	1
Q897	Q00-Q99	Congenital malformations, deformations anc	Multiple	Multiple congenital malformations,not elsewhere classified	0	0	0	1	0	0	1	1
Q898	Q00-Q99	Congenital malformations, deformations anc	Other	Other specified congenital malformations	0	0	0	1	0	0	1	1
Q899	Q00-Q99	Congenital malformations, deformations anc	Other	Congenital malformation, unspecified	0	0	0	1	0	0	1	1
Q90	Q00-Q99	Congenital malformations, deformations anc	Chromosomal	Down syndrome	1	1	0	0	1	0	0	
Q900	Q00-Q99	Congenital malformations, deformations anc	Chromosomal	Trisomy 21, nonmosaicism (meioticnondisjunction)	1	1	0	0	1	0	0	
Q901	Q00-Q99	Congenital malformations, deformations anc	Chromosomal	Trisomy 21, mosaicism (mitotic nondisjunction)	1	1	0	0	1	0	0	
Q902	Q00-Q99	Congenital malformations, deformations anc	Chromosomal	Trisomy 21, translocation	1	1	0	0	1	0	0	
Q909	Q00-Q99	Congenital malformations, deformations anc	Chromosomal	Down syndrome, unspecified	1	1	0	0	1	0	0	
Q91	Q00-Q99	Congenital malformations, deformations anc	Chromosomal	Trisomy 18 and Trisomy 13	1	1	0	0	1	0	0	
Q910	Q00-Q99	Congenital malformations, deformations anc	Chromosomal	Trisomy 18, nonmosaicism (meioticnondisjunction)	1	1	0	0	1	0	0	
Q911	Q00-Q99	Congenital malformations, deformations anc	Chromosomal	Trisomy 18, mosaicism (mitotic nondisjunction)	1	1	0	0	1	0	0	
Q912	Q00-Q99	Congenital malformations, deformations anc	Chromosomal	Trisomy 18, translocation	1	1	0	0	1	0	0	
Q913	Q00-Q99	Congenital malformations, deformations anc	Chromosomal	Trisomy 18, unspecified	1	1	0	0	1	0	0	
Q914	Q00-Q99	Congenital malformations, deformations anc	Chromosomal	Trisomy 13, nonmosaicism (meioticnondisjunction)	1	1	0	0	1	0	0	
Q915	Q00-Q99	Congenital malformations, deformations anc	Chromosomal	Trisomy 13, mosaicism (mitotic nondisjunction)	1	1	0	0	1	0	0	
Q916	Q00-Q99	Congenital malformations, deformations anc	Chromosomal	Trisomy 13, translocation	1	1	0	0	1	0	0	
Q917	Q00-Q99	Congenital malformations, deformations anc	Chromosomal	Trisomy 13, unspecified	1	1	0	0	1	0	0	
Q92	Q00-Q99	Congenital malformations, deformations anc	Chromosomal	Other trisomies and partial trisomies of the autosomes, not elsewhere classified	1	1	0	0	1	0	0	
Q920	Q00-Q99	Congenital malformations, deformations anc	Chromosomal	Whole chromosome trisomy, nonmosaicism (meiotic nondisjunction)	1	1	0	0	1	0	0	
Q921	Q00-Q99	Congenital malformations, deformations anc	Chromosomal	Whole chromosome trisomy, mosaicism (mitotic nondisjunction)	1	1	0	0	1	0	0	
Q922	Q00-Q99	Congenital malformations, deformations anc	Chromosomal	Partial trisomy	1	1	0	0	1	0	0	
Q925	Q00-Q99	Congenital malformations, deformations anc	Chromosomal	Duplications with other complex rearrangements	1	1	0	0	1	0	0	
Q926	Q00-Q99	Congenital malformations, deformations anc	Chromosomal	Marker chromosomes	1	1	0	0	1	0	0	
Q9261	Q00-Q99	Congenital malformations, deformations anc	Chromosomal	Marker chromosomes in normal individual	1	1	0	0	1	0	0	
Q9262	Q00-Q99	Congenital malformations, deformations anc	Chromosomal	Marker chromosomes in abnormal individual	1	1	0	0	1	0	0	
Q927	Q00-Q99	Congenital malformations, deformations anc	Chromosomal	Triploidy and polyploidy	1	1	0	0	1	0	0	
Q928	Q00-Q99	Congenital malformations, deformations anc	Chromosomal	Other specified trisomies and partial trisomies of autosomes	1	1	0	0	1	0	0	
Q929	Q00-Q99	Congenital malformations, deformations anc	Chromosomal	Trisomy and partial trisomy of autosomes, unspecified	1	1	0	0	1	0	0	
Q93	Q00-Q99	Congenital malformations, deformations anc	Chromosomal	Monosomies and deletions from theautosomes, not elsewhere classified	1	1	0	0	1	0	0	
Q930	Q00-Q99	Congenital malformations, deformations anc	Chromosomal	Whole chromosome monosomy, nonmosaicism (meiotic nondisjunction)	1	1	0	0	1	0	0	
Q931	Q00-Q99	Congenital malformations, deformations anc	Chromosomal	Whole chromosome monosomy, mosaicism (mitotic nondisjunction)	1	1	0	0	1	0	0	
Q932	Q00-Q99	Congenital malformations, deformations anc	Chromosomal	Chromosome replaced with ring, dicentric or isochromosome	1	1	0	0	1	0	0	
Q933	Q00-Q99	Congenital malformations, deformations anc	Chromosomal	Deletion of short arm of chromosome 4	1	1	0	0	1	0	0	
Q934	Q00-Q99	Congenital malformations, deformations anc	Chromosomal	Deletion of short arm of chromosome 5	1	1	0	0	1	0	0	
Q935	Q00-Q99	Congenital malformations, deformations anc	Chromosomal	Other deletions of part of a chromosome	1	1	0	0	1	0	0	
Q937	Q00-Q99	Congenital malformations, deformations anc	Chromosomal	Deletions with other complex rearrangements	1	1	0	0	1	0	0	
Q938	Q00-Q99	Congenital malformations, deformations anc	Chromosomal	Other deletions from the autosomes	1	1	0	0	1	0	0	
Q9381	Q00-Q99	Congenital malformations, deformations anc	Chromosomal	Velo-cardio-facial syndrome	1	1	0	0	1	0	0	
Q9388	Q00-Q99	Congenital malformations, deformations anc	Chromosomal	Other microdeletions	1	1	0	0	1	0	0	
Q9389	Q00-Q99	Congenital malformations, deformations anc	Chromosomal	Other deletions from the autosomes	1	1	0	0	1	0	0	
Q939	Q00-Q99	Congenital malformations, deformations anc	Chromosomal	Deletion from autosomes, unspecified	1	1	0	0	1	0	0	
Q95	Q00-Q99	Congenital malformations, deformations anc	Chromosomal	Balanced rearrangements and structural markers, not elsewhere classified	1	1	0	0	1	0	0	
Q950	Q00-Q99	Congenital malformations, deformations anc	Chromosomal	Balanced translocation and insertion in normal individual	1	1	0	0	1	0	0	
Q951	Q00-Q99	Congenital malformations, deformations anc	Chromosomal	Chromosome inversion in normal individual	1	1	0	0	1	0	0	
Q952	Q00-Q99	Congenital malformations, deformations anc	Chromosomal	Balanced autosomal rearrangement in abnormal individual	1	1	0	0	1	0	0	
Q953	Q00-Q99	Congenital malformations, deformations anc	Chromosomal	Balanced sex/autosomal rearrangement in abnormal individual	1	1	0	0	1	0	0	
Q955	Q00-Q99	Congenital malformations, deformations anc	Chromosomal	Individual with autosomal fragilesite	1	1	0	0	1	0	0	
Q958	Q00-Q99	Congenital malformations, deformations anc	Chromosomal	Other balanced rearrangements andstructural markers	1	1	0	0	1	0	0	
Q959	Q00-Q99	Congenital malformations, deformations anc	Chromosomal	Balanced rearrangement and structural marker, unspecified	1	1	0	0	1	0	0	
Q96	Q00-Q99	Congenital malformations, deformations anc	Chromosomal	Turner's syndrome	1	1	0	0	1	0	0	
Q960	Q00-Q99	Congenital malformations, deformations anc	Chromosomal	Karyotype 45, X	1	1	0	0	1	0	0	
Q961	Q00-Q99	Congenital malformations, deformations anc	Chromosomal	Karyotype 46, X iso (Xq)	1	1	0	0	1	0	0	
Q962	Q00-Q99	Congenital malformations, deformations anc	Chromosomal	Karyotype 46, X with abnormal sexchromosome, except iso (Xq)	1	1	0	0	1	0	0	
Q963	Q00-Q99	Congenital malformations, deformations anc	Chromosomal	Mosaicism, 45, X/46, XX or XY	1	1	0	0	1	0	0	
Q964	Q00-Q99	Congenital malformations, deformations anc	Chromosomal	Mosaicism, 45, X/other cell line(s) with abnormal sex chromosome	1	1	0	0	1	0	0	
Q968	Q00-Q99	Congenital malformations, deformations anc	Chromosomal	Other variants of Turner's syndrome	1	1	0	0	1	0	0	
Q969	Q00-Q99	Congenital malformations, deformations anc	Chromosomal	Turner's syndrome, unspecified	1	1	0	0	1	0	0	
Q97	Q00-Q99	Congenital malformations, deformations anc	Chromosomal	Other sex chromosome abnormalities, female phenotype, not elsewhere classified	1	1	0	0	1	0	0	
Q970	Q00-Q99	Congenital malformations, deformations anc	Chromosomal	Karyotype 47, XXX	1	1	0	0	1	0	0	
Q971	Q00-Q99	Congenital malformations, deformations anc	Chromosomal	Female with more than three X chromosomes	1	1	0	0	1	0	0	

Q972	Q00-Q99	Congenital malformations, deformations an	Chromosomal	Mosaicism, lines with various numbers of X chromosomes	1	1	0	0	1	0	0
Q973	Q00-Q99	Congenital malformations, deformations an	Chromosomal	Female with 46, XY karyotype	1	1	0	0	1	0	0
Q978	Q00-Q99	Congenital malformations, deformations an	Chromosomal	Other specified sex chromosome abnormalities, female phenotype	1	1	0	0	1	0	0
Q979	Q00-Q99	Congenital malformations, deformations an	Chromosomal	Sex chromosome abnormality, femalephenotype, unspecified	1	1	0	0	1	0	0
Q98	Q00-Q99	Congenital malformations, deformations an	Chromosomal	Other sex chromosome abnormalities, male phenotype, not elsewhere classified	1	1	0	0	1	0	0
Q980	Q00-Q99	Congenital malformations, deformations an	Chromosomal	Klinefelter syndrome karyotype 47,XXY	1	1	0	0	1	0	0
Q981	Q00-Q99	Congenital malformations, deformations an	Chromosomal	Klinefelter syndrome, male with more than two X chromosomes	1	1	0	0	1	0	0
Q983	Q00-Q99	Congenital malformations, deformations an	Chromosomal	Other male with 46, XX karyotype	0	1	0	0	1	0	0
Q984	Q00-Q99	Congenital malformations, deformations an	Chromosomal	Klinefelter syndrome, unspecified	1	1	0	0	1	0	0
Q985	Q00-Q99	Congenital malformations, deformations an	Chromosomal	Karyotype 47, XYY	1	1	0	0	1	0	0
Q986	Q00-Q99	Congenital malformations, deformations an	Chromosomal	Male with structurally abnormal sex chromosome	1	1	0	0	1	0	0
Q987	Q00-Q99	Congenital malformations, deformations an	Chromosomal	Male with sex chromosome mosaicism	1	1	0	0	1	0	0
Q988	Q00-Q99	Congenital malformations, deformations an	Chromosomal	Other specified sex chromosome abnormalities, male phenotype	1	1	0	0	1	0	0
Q989	Q00-Q99	Congenital malformations, deformations an	Chromosomal	Sex chromosome abnormality, male phenotype, unspecified	1	1	0	0	1	0	0
Q99	Q00-Q99	Congenital malformations, deformations an	Chromosomal	Other chromosome abnormalities, not elsewhere classified	1	1	0	0	1	0	0
Q990	Q00-Q99	Congenital malformations, deformations an	Chromosomal	Chimera 46, XX/46, XY	1	1	0	0	1	0	0
Q991	Q00-Q99	Congenital malformations, deformations an	Chromosomal	46, XX true hermaphrodite	0	1	0	0	1	0	0
Q992	Q00-Q99	Congenital malformations, deformations an	Chromosomal	Fragile X chromosome	1	1	0	0	1	0	0
Q998	Q00-Q99	Congenital malformations, deformations an	Chromosomal	Other specified chromosome abnormalities	1	1	0	0	1	0	0
Q999	Q00-Q99	Congenital malformations, deformations an	Chromosomal	Chromosomal abnormality, unspecified	1	1	0	0	1	0	0
R16	R00-R99	Symptoms, signs, and abnormal clinical and	NA	Hepatomegaly and splenomegaly, notelsewhere classified	0	0	0	1	0	0	1
R160	R00-R99	Symptoms, signs, and abnormal clinical and	NA	Hepatomegaly, not elsewhere classified	0	0	0	1	0	0	1
R162	R00-R99	Symptoms, signs, and abnormal clinical and	NA	Hepatomegaly with splenomegaly, not elsewhere classified	0	0	0	1	0	0	1
R25	R00-R99	Symptoms, signs, and abnormal clinical and	NA	Abnormal involuntary movements	0	0	0	1	0	0	1
R260	R00-R99	Symptoms, signs, and abnormal clinical and	NA	Ataxic gait	0	0	0	1	0	0	1
R4183	R00-R99	Symptoms, signs, and abnormal clinical and	NA	Borderline intellectual functioning	0	0	0	1	0	0	1
R430	R00-R99	Symptoms, signs, and abnormal clinical and	NA	Anosmia	0	0	0	1	0	0	1
R441	R00-R99	Symptoms, signs, and abnormal clinical and	NA	Visual hallucinations	0	0	0	1	0	0	1
R442	R00-R99	Symptoms, signs, and abnormal clinical and	NA	Other hallucinations	0	0	0	1	0	0	1
R443	R00-R99	Symptoms, signs, and abnormal clinical and	NA	Hallucinations, unspecified	0	0	0	1	0	0	1
R6250	R00-R99	Symptoms, signs, and abnormal clinical and	NA	UNS LACK EXP NL PHYSIOLOG DEV CHLD	0	0	0	1	0	0	0
R6251	R00-R99	Symptoms, signs, and abnormal clinical and	NA	FAILURE TO THRIVE CHILD	0	0	0	1	0	0	0
R6259	R00-R99	Symptoms, signs, and abnormal clinical and	NA	OTH LACK EXP NL PHYSIOLOG DEV CHLD	0	0	0	1	0	0	0
R94131	R00-R99	Symptoms, signs, and abnormal clinical and	NA	Abnormal electromyogram [EMG]	0	0	0	1	0	0	1
T883	S00-T88	Injury, poisoning, and certain other conseque	NA	Malignant hyperthermia due to anesthesia	1	1	0	0	1	0	0
T883XXA	S00-T88	Injury, poisoning, and certain other conseque	NA	Malignant hyperthermia due to anesthesia, initial encounter	1	1	0	0	1	0	0
T883XXD	S00-T88	Injury, poisoning, and certain other conseque	NA	Malignant hyperthermia due to anesthesia, subsequent encounter	1	1	0	0	1	0	0
T883XXS	S00-T88	Injury, poisoning, and certain other conseque	NA	Malignant hyperthermia due to anesthesia, sequela	1	1	0	0	1	0	0
Z054	Z00-Z99	Factors influencing health status and contac	NA	Observation and evaluation of newborn for suspected genetic, metabolic or immunolo	0	0	1	0	0	1	0
Z0541	Z00-Z99	Factors influencing health status and contac	NA	Observation and evaluation of newborn for suspected genetic condition ruled out	0	0	1	0	0	1	0
Z137	Z00-Z99	Factors influencing health status and contac	NA	Encounter for screening for genetic and chromosomal anomalies	0	0	1	0	0	1	0
Z1379	Z00-Z99	Factors influencing health status and contac	NA	Encounter for other screening forgenetic and chromosomal anomalies	0	0	0	1	0	0	1
Z14	Z00-Z99	Factors influencing health status and contac	NA	Genetic carrier	0	0	1	0	0	1	0
Z148	Z00-Z99	Factors influencing health status and contac	NA	Genetic carrier of other disease	0	0	1	0	0	1	0
Z15	Z00-Z99	Factors influencing health status and contac	NA	Genetic susceptibility to disease	0	0	1	0	0	1	0
Z150	Z00-Z99	Factors influencing health status and contac	NA	Genetic susceptibility to malignant neoplasm	0	0	1	0	0	1	0
Z1501	Z00-Z99	Factors influencing health status and contac	NA	Genetic susceptibility to malignant neoplasm of breast	0	0	0	1	0	0	0
Z1502	Z00-Z99	Factors influencing health status and contac	NA	Genetic susceptibility to malignant neoplasm of ovary	0	0	0	1	0	0	0
Z1503	Z00-Z99	Factors influencing health status and contac	NA	Genetic susceptibility to malignant neoplasm of prostate	0	0	1	0	0	1	0
Z1504	Z00-Z99	Factors influencing health status and contac	NA	Genetic susceptibility to malignant neoplasm of endometrium	0	0	1	0	0	1	0
Z1509	Z00-Z99	Factors influencing health status and contac	NA	Genetic susceptibility to other malignant neoplasm	0	0	1	0	0	1	0
Z158	Z00-Z99	Factors influencing health status and contac	NA	Genetic susceptibility to other disease	0	0	1	0	0	1	0
Z1581	Z00-Z99	Factors influencing health status and contac	NA	Genetic susceptibility to multipleendocrine neoplasia [MEN]	1	1	0	0	1	0	0
Z1589	Z00-Z99	Factors influencing health status and contac	NA	Genetic susceptibility to other disease	0	0	1	0	0	1	0
Z315	Z00-Z99	Factors influencing health status and contac	NA	Encounter for genetic counseling	0	1	0	0	1	0	0
Z800	Z00-Z99	Factors influencing health status and contac	NA	FAM HX MALIG NEOPLASM DIGESTIV ORGN	0	0	0	1	0	0	1
Z801	Z00-Z99	Factors influencing health status and contac	NA	FAM HX MALIG NEOPLSM TRACH BRON LNG	0	0	0	1	0	0	1
Z802	Z00-Z99	Factors influencing health status and contac	NA	FAM HX MAL NEOPLSM OTH RESP&IT ORGN	0	0	0	1	0	0	1
Z803	Z00-Z99	Factors influencing health status and contac	NA	FAMILY HX MALIG NEOPLASM OF BREAST	0	0	0	1	0	0	1
Z8041	Z00-Z99	Factors influencing health status and contac	NA	FAM HX MALIGNANT NEOPLASM OVARY	0	0	0	1	0	0	1
Z8042	Z00-Z99	Factors influencing health status and contac	NA	FAMILY HX MALIG NEOPLASM PROSTATE	0	0	0	1	0	0	1
Z8043	Z00-Z99	Factors influencing health status and contac	NA	FAM HX MALIGNANT NEOPLASM TESTIS	0	0	0	1	0	0	1
Z8049	Z00-Z99	Factors influencing health status and contac	NA	FAM HX MALIG NEOPLSM OTH GENIT ORGN	0	0	0	1	0	0	1
Z8051	Z00-Z99	Factors influencing health status and contac	NA	FAM HX MALIGNANT NEOPLASM KIDNEY	0	0	0	1	0	0	1
Z8052	Z00-Z99	Factors influencing health status and contac	NA	FAM HX MALIGNANT NEOPLASM BLADDER	0	0	0	1	0	0	1
Z8059	Z00-Z99	Factors influencing health status and contac	NA	FAM HX MAL NEO OTH URIN TRACT ORGN	0	0	0	1	0	0	1
Z806	Z00-Z99	Factors influencing health status and contac	NA	FAMILY HISTORY OF LEUKEMIA	0	0	0	1	0	0	1
Z807	Z00-Z99	Factors influencing health status and contac	NA	FAM HX OTH MAL NEO LYMPH HEMATPOETC	0	0	0	1	0	0	1
Z808	Z00-Z99	Factors influencing health status and contac	NA	FAM HX MALIG NEOPLASM OTH ORGN/SYS	0	0	0	1	0	0	1
Z809	Z00-Z99	Factors influencing health status and contac	NA	FAM HX MALIGNANT NEOPLASM UNS	0	0	0	1	0	0	1
Z81	Z00-Z99	Factors influencing health status and contac	NA	Family history of mental and behavioral disorders	0	0	0	1	0	0	1
Z810	Z00-Z99	Factors influencing health status and contac	NA	Family history of intellectual disabilities	0	0	0	1	0	0	1
Z82	Z00-Z99	Factors influencing health status and contac	NA	Family history of certain disabilities and chronic diseases (leading to disablement)	0	0	0	1	0	0	1
Z820	Z00-Z99	Factors influencing health status and contac	NA	Family history of epilepsy and other diseases of the nervous system	0	0	0	1	0	0	1
Z821	Z00-Z99	Factors influencing health status and contac	NA	Family history of blindness and visual loss	0	0	0	1	0	0	1
Z822	Z00-Z99	Factors influencing health status and contac	NA	Family history of deafness and hearing loss	0	0	0	1	0	0	1
Z8241	Z00-Z99	Factors influencing health status and contac	NA	Family history of sudden cardiac death	0	0	0	1	0	0	1
Z8269	Z00-Z99	Factors influencing health status and contac	NA	Family history of other diseases of the musculoskeletal system and connective tissue	0	0	0	1	0	0	1
Z827	Z00-Z99	Factors influencing health status and contac	NA	Family history of congenital malformations, deformations and chromosomal abnorma	0	0	0	1	0	0	1
Z8271	Z00-Z99	Factors influencing health status and contac	NA	Family history of polycystic kidney	0	0	0	1	0	0	1
Z8279	Z00-Z99	Factors influencing health status and contac	NA	Family history of other congenitalmalformations, deformations and chromosomal abn	0	0	0	1	0	0	1
Z828	Z00-Z99	Factors influencing health status and contac	NA	Family history of other disabilities and chronic diseases leading to disablement, not els	0	0	0	1	0	0	1
Z8341	Z00-Z99	Factors influencing health status and contac	NA	Family history of multiple endocrine neoplasia [MEN] syndrome	0	0	0	1	0	0	1
Z8342	Z00-Z99	Factors influencing health status and contac	NA	Family history of familial hypercholesterolemia	0	0	0	1	0	0	1
Z835	Z00-Z99	Factors influencing health status and contac	NA	Family history of eye and ear disorders	0	0	0	1	0	0	1
Z83511	Z00-Z99	Factors influencing health status and contac	NA	Family history of glaucoma	0	0	0	1	0	0	1
Z83518	Z00-Z99	Factors influencing health status and contac	NA	FM HX OTHER SPECIFIED EYE DISORDER	0	0	0	1	0	0	1
Z8352	Z00-Z99	Factors influencing health status and contac	NA	FAMILY HISTORY OF EAR DISORDERS	0	0	0	1	0	0	1
Z8371	Z00-Z99	Factors influencing health status and contac	NA	Family history of colonic polyps	0	0	0	1	0	0	1
Z843	Z00-Z99	Factors influencing health status and contac	NA	Family history of consanguinity	0	0	0	1	0	0	1
Z8481	Z00-Z99	Factors influencing health status and contac	NA	Family history of carrier of genetic disease	0	0	0	1	0	0	1
Z8482	Z00-Z99	Factors influencing health status and contac	NA	Family history of sudden infant death syndrome	0	0	0	1	0	0	1
Z877	Z00-Z99	Factors influencing health status and contac	NA	Personal history of (corrected) congenital malformations	0	0	0	1	0	0	1
Z8771	Z00-Z99	Factors influencing health status and contac	NA	Personal history of (corrected) congenital malformations of genitourinary system	0	0	0	1	0	0	1
Z87718	Z00-Z99	Factors influencing health status and contac	NA	Personal history of other specified (corrected) congenital malformations of genitourina	0	0	0	1	0	0	1
Z8772	Z00-Z99	Factors influencing health status and contac	NA	Personal history of (corrected) congenital malformations of nervous system and sense	0	0	0	1	0	0	1
Z87720	Z00-Z99	Factors influencing health status and contac	NA	Personal history of (corrected) congenital malformations of eye	0	0	0	1	0	0	1
Z87721	Z00-Z99	Factors influencing health status and contac	NA	Personal history of (corrected) congenital malformations of ear	0	0	0	1	0	0	1
Z87728	Z00-Z99	Factors influencing health status and contac	NA	Personal history of other specified (corrected) congenital malformations of nervous sy	0	0	0	1	0	0	1
Z8773	Z00-Z99	Factors influencing health status and contac	NA	Personal history of (corrected) congenital malformations of digestive system	0	0	0	1	0	0	1
Z87738	Z00-Z99	Factors influencing health status and contac	NA	Personal history of other specified (corrected) congenital malformations of digestive s	0	0	0	1	0	0	1
Z8774	Z00-Z99	Factors influencing health status and contac	NA	Personal history of (corrected) congenital malformations of heart and circulatory syste	0	0	0	1	0	0	1
Z8775	Z00-Z99	Factors influencing health status and contac	NA	Personal history of (corrected) congenital malformations of respiratory system	0	0	0	1	0	0	1
Z8776	Z00-Z99	Factors influencing health status and contac	NA	Personal history of (corrected) congenital malformations of integument, limbs and mu	0	0	0	1	0	0	1
Z8779	Z00-Z99	Factors influencing health status and contac	NA	Personal history of other (corrected) congenital malformations	0	0	0	1	0	0	1
Z87790	Z00-Z99	Factors influencing health status and contac	NA	Personal history of (corrected) congenital malformations of face and neck	0	0	0	1	0	0	1
Z87798	Z00-Z99	Factors influencing health status and contac	NA	Personal history of other (corrected) congenital malformations	0	0	0	1	0	0	1

CPT	Clinician Descriptor	Genetic Test	Newborn_Definite	Newborn_Probable	Newborn_Possible	Ped_Definite	Ped_Probable	Ped_Possible
61862	Twist drill, burr hole, craniotomy, or craniectomy for stereotactic implantation of one neurostimulator array	0	0	0	0	0	0	1
61875	Craniectomy for implantation of neurostimulator electrodes, cerebellar; subcortical	0	0	0	0	0	0	1
63660	Revision or removal of spinal neurostimulator electrode percutaneous array(s) or plate/paddle(s)	0	0	0	0	0	0	1
64560	Percutaneous implantation of neurostimulator electrodes; autonomic nerve	0	0	0	0	0	0	1
64565	Percutaneous implantation of neurostimulator electrode array; neuromuscular	0	0	0	0	0	0	1
64573	Incision for implantation of neurostimulator electrodes; cranial nerve	0	0	0	0	0	0	1
64577	Incision for implantation of neurostimulator electrodes; autonomic nerve	0	0	0	0	0	0	1
95973	Electronic analysis of implanted neurostimulator pulse generator system (eg, rate, pulse amplitude, pulse d	0	0	0	0	0	0	1
00102	Anesthesia for plastic repair of cleft lip	0	0	0	1	0	0	1
00103	Anesthesia for reconstruction of eyelid	0	0	0	1	0	0	0
00120	Anesthesia for procedure on external ear	0	0	0	1	0	0	1
0012U	Next-generation sequencing of whole genome for detection of gene rearrangement associated with germl	1	0	0	0	0	0	0
00142	Anesthesia for surgery of lens of eye	0	0	0	1	0	0	1
00172	Anesthesia for repair of cleft palate	0	0	0	1	0	0	1
00215	Anesthesia for cranioplasty	0	0	0	1	0	0	1
00320	Anesthesia for procedure on esophagus, thyroid, larynx, trachea and lymphatic system of neck	0	0	0	1	0	0	0
00326	Anesthesia for procedure on larynx	0	0	0	1	0	0	0
00560	Anesthesia for procedure on heart, pericardial sac, and great vessels of chest	0	0	0	1	0	0	1
00561	Anesthesia for procedure on heart, pericardial sac, and great vessels of chest with pump oxygenator	0	0	0	1	0	0	1
00562	Anesthesia for procedure on heart, pericardial sac, and great vessels of chest with pump oxygenator	0	0	0	1	0	0	1
00563	Anesthesia for procedure on heart, pericardial sac, and great vessels of chest with pump oxygenator and h	0	0	0	1	0	0	1
00580	Anesthesia for heart transplant	0	0	0	1	0	0	0
00702	Anesthesia for percutaneous liver biopsy	0	0	0	1	0	0	0
00754	Anesthesia for repair of omphalocele	0	0	0	1	0	0	0
00756	Anesthesia for transabdominal repair of diaphragmatic hernia	0	0	0	1	0	0	0
00794	Anesthesia for partial pancreatectomy	0	0	0	1	0	0	0
00920	Anesthesia for procedure on male genitalia	0	0	0	1	0	0	0
00924	Anesthesia for procedure on bilateral undescended testis	0	0	0	1	0	0	0
00926	Anesthesia for radical inguinal orchiectomy	0	0	0	1	0	0	0
00928	Anesthesia for radical abdominal orchiectomy	0	0	0	1	0	0	0
0100T	Placement of a subconjunctival retinal prosthesis receiver and pulse generator, and implantation of intra-o	0	0	0	0	0	0	1
0162T	Electronic analysis and programming, reprogramming of gastric neurostimulator (ie, morbid obesity)	0	0	0	0	0	0	1
0282T	Percutaneous or open implantation of neurostimulator electrode array(s), subcutaneous (peripheral subcut	0	0	0	0	0	0	1
0283T	Percutaneous or open implantation of neurostimulator electrode array(s), subcutaneous (peripheral subcut	0	0	0	0	0	0	1
0312T	Vagus nerve blocking therapy (morbid obesity); laparoscopic implantation of neurostimulator electrode ar	0	0	0	0	0	0	1
0313T	Vagus nerve blocking therapy (morbid obesity); laparoscopic revision or replacement of vagal trunk neuro	0	0	0	0	0	0	1
0314T	Vagus nerve blocking therapy (morbid obesity); laparoscopic removal of vagal trunk neurostimulator elect	0	0	0	0	0	0	1
0317T	Vagus nerve blocking therapy (morbid obesity); neurostimulator pulse generator electronic analysis, incluc	0	0	0	0	0	0	1
0424T	Insertion or replacement of neurostimulator system for treatment of central sleep apnea; complete syster	0	0	0	0	0	0	1
0425T	Insertion or replacement of neurostimulator system for treatment of central sleep apnea; sensing lead onl	0	0	0	0	0	0	1
0426T	Insertion or replacement of neurostimulator system for treatment of central sleep apnea; stimulation lead	0	0	0	0	0	0	1
0427T	Insertion or replacement of neurostimulator system for treatment of central sleep apnea; pulse generator	0	0	0	0	0	0	1
0428T	Removal of neurostimulator system for treatment of central sleep apnea; pulse generator only	0	0	0	0	0	0	1
0429T	Removal of neurostimulator system for treatment of central sleep apnea; sensing lead only	0	0	0	0	0	0	1
0430T	Removal of neurostimulator system for treatment of central sleep apnea; stimulation lead only	0	0	0	0	0	0	1
0431T	Removal and replacement of neurostimulator system for treatment of central sleep apnea, pulse generatc	0	0	0	0	0	0	1
0432T	Repositioning of neurostimulator system for treatment of central sleep apnea; stimulation lead only	0	0	0	0	0	0	1
0433T	Repositioning of neurostimulator system for treatment of central sleep apnea; sensing lead only	0	0	0	0	0	0	1
0434T	Interrogation device evaluation implanted neurostimulator pulse generator system for central sleep apnea	0	0	0	0	0	0	1
0435T	Programming device evaluation of implanted neurostimulator pulse generator system for central sleep ap	0	0	0	0	0	0	1
0436T	Programming device evaluation of implanted neurostimulator pulse generator system for central sleep ap	0	0	0	0	0	0	1
13131	Repair of genitalia	0	0	0	1	0	0	0
13132	Repair of genitalia	0	0	0	1	0	0	0
13133	Repair of genitalia	0	0	0	1	0	0	0
13151	Repair of ear	0	0	0	1	0	0	0
13152	Repair of ear	0	0	0	1	0	0	0
13153	Repair of ear	0	0	0	1	0	0	0
20200	Biopsy of muscle	0	0	0	1	0	0	1
20205	Biopsy of muscle	0	0	0	1	0	0	1
20206	Percutaneous needle biopsy of muscle	0	0	0	1	0	0	1
21260	Correction of hypertelorism with periorbital osteotomy and bone graft	0	0	0	0	0	0	1
21261	Correction of hypertelorism with periorbital osteotomy and bone graft	0	0	0	0	0	0	1
21263	Correction of hypertelorism with periorbital osteotomy, bone graft and forehead advancement	0	0	0	0	0	0	1
26560	Repair of syndactyly with skin flap	0	0	0	0	0	0	1
26561	Repair of syndactyly with skin flap and graft	0	0	0	0	0	0	1
26562	Repair of syndactyly	0	0	0	0	0	0	1
28262	Extensive capsulotomy of midfoot with posterior talotibial capsulotomy and lengthening of tendon for exte	0	0	0	1	0	0	0

29450	Application of clubfoot cast with manipulation	0	0	0	1	0	0	0
29750	Wedging of clubfoot cast	0	0	0	1	0	0	0
30460	Rhinoplasty for nasal deformity secondary to congenital cleft lip and palate	0	0	0	1	0	0	1
30462	Rhinoplasty for nasal deformity secondary to congenital cleft lip	0	0	0	1	0	0	1
30540	Repair of choanal atresia by intranasal approach	0	0	0	1	0	0	0
30545	Repair of choanal atresia by transpalatine approach	0	0	0	1	0	0	0
33202	Open insertion of epicardial electrode	0	0	0	1	0	0	1
33203	Endoscopic insertion of epicardial electrode	0	0	0	1	0	0	1
33206	Insertion of permanent atrial pacemaker with transvenous electrode	0	0	0	1	0	0	1
33207	Insertion of permanent ventricular pacemaker with transvenous electrode	0	0	0	1	0	0	1
33208	Insertion of permanent atrial and ventricular pacemaker with transvenous electrode	0	0	0	1	0	0	1
33210	Insertion of temporary transvenous single chamber cardiac electrode	0	0	0	1	0	0	1
33211	Insertion of temporary transvenous dual chamber pacing electrodes	0	0	0	1	0	0	1
33212	Insertion of pacemaker pulse generator	0	0	0	1	0	0	1
33213	Insertion of pacemaker pulse generator	0	0	0	1	0	0	1
33214	Conversion of single chamber implanted pacemaker system to dual chamber system	0	0	0	1	0	0	1
33215	Repositioning of transvenous pacemaker electrode	0	0	0	1	0	0	1
33216	Insertion of transvenous electrode of permanent cardioverter-defibrillator	0	0	0	1	0	0	1
33217	Insertion of transvenous electrode of permanent cardioverter-defibrillator	0	0	0	1	0	0	1
33218	Repair of transvenous electrode of permanent pacemaker	0	0	0	1	0	0	1
33220	Repair of transvenous electrode of permanent pacemaker	0	0	0	1	0	0	1
33221	Insertion of pacemaker pulse generator	0	0	0	1	0	0	1
33222	Relocation of skin pocket for pacemaker	0	0	0	1	0	0	1
33223	Revision of skin pocket for cardioverter-defibrillator	0	0	0	1	0	0	1
33224	Transvenous insertion of pacing electrode for left ventricular pacing	0	0	0	1	0	0	1
33225	Transvenous insertion of pacing electrode for left ventricular pacing and insertion of pacemaker pulse gen	0	0	0	1	0	0	1
33226	Repositioning of left ventricular electrode	0	0	0	1	0	0	1
33227	Removal and replacement of permanent pacemaker pulse generator	0	0	0	1	0	0	1
33228	Removal and replacement of permanent pacemaker pulse generator	0	0	0	1	0	0	1
33229	Removal and replacement of permanent pacemaker pulse generator	0	0	0	1	0	0	1
33230	Insertion of pacing cardioverter-defibrillator pulse generator	0	0	0	1	0	0	1
33231	Insertion of pacing cardioverter-defibrillator pulse generator	0	0	0	1	0	0	1
33233	Removal of permanent pacemaker pulse generator	0	0	0	1	0	0	1
33234	Removal of transvenous pacemaker electrode	0	0	0	1	0	0	1
33235	Removal of transvenous pacemaker electrode	0	0	0	1	0	0	1
33236	Removal of permanent epicardial pacemaker and electrodes via thoracotomy	0	0	0	1	0	0	1
33237	Removal of permanent epicardial pacemaker and electrodes via thoracotomy	0	0	0	1	0	0	1
33238	Removal of permanent transvenous electrode via thoracotomy	0	0	0	1	0	0	1
33240	Insertion of pacing cardioverter-defibrillator pulse generator	0	0	0	1	0	0	1
33241	Removal of pacing cardioverter-defibrillator pulse generator	0	0	0	1	0	0	1
33243	Removal of dual chamber pacing cardioverter-defibrillator electrode via thoracotomy	0	0	0	1	0	0	1
33244	Transvenous removal of single chamber pacing cardioverter-defibrillator electrode	0	0	0	1	0	0	1
33249	Insertion of dual chamber permanent pacing cardioverter-defibrillator system with transvenous lead	0	0	0	1	0	0	1
33250	Surgical ablation of supraventricular arrhythmogenic focus	0	0	0	0	0	0	1
33251	Surgical ablation of supraventricular arrhythmogenic focus with cardiopulmonary bypass	0	0	0	0	0	0	1
33254	Surgical tissue ablation and reconstruction of atrium	0	0	0	0	0	0	1
33255	Surgical tissue ablation and reconstruction of atrium	0	0	0	0	0	0	1
33256	Surgical tissue ablation and reconstruction of atrium with cardiopulmonary bypass	0	0	0	0	0	0	1
33257	Surgical tissue ablation and reconstruction of atrium	0	0	0	0	0	0	1
33258	Surgical tissue ablation and reconstruction of atrium	0	0	0	0	0	0	1
33259	Surgical tissue ablation and reconstruction of atrium with cardiopulmonary bypass	0	0	0	0	0	0	1
33261	Surgical tissue ablation of ventricular arrhythmogenic focus with cardiopulmonary bypass	0	0	0	0	0	0	1
33262	Removal and replacement of pacing cardioverter-defibrillator pulse generator	0	0	0	1	0	0	1
33263	Removal and replacement of pacing cardioverter-defibrillator pulse generator	0	0	0	1	0	0	1
33264	Removal and replacement of pacing cardioverter-defibrillator pulse generator	0	0	0	1	0	0	1
33265	Surgical endoscopy with surgical tissue ablation and reconstruction of atrium	0	0	0	0	0	0	1
33266	Surgical endoscopy with surgical tissue ablation and reconstruction of atrium	0	0	0	0	0	0	1
33270	Insertion of permanent subcutaneous implantable defibrillator system with subcutaneous electrode	0	0	0	1	0	0	1
33271	Insertion of subcutaneous implantable defibrillator electrode	0	0	0	1	0	0	1
33272	Removal of subcutaneous implantable defibrillator electrode	0	0	0	1	0	0	1
33273	Repositioning of subcutaneous implantable defibrillator electrode	0	0	0	1	0	0	1
33390	Open simple commissural resuspension of aortic valve, with cardiopulmonary bypass	0	0	0	1	0	0	1
33391	Complex open valvuloplasty of aortic valve, with cardiopulmonary bypass	0	0	0	1	0	0	1
33404	Construction of apical-aortic conduit	0	0	0	1	0	0	1
33405	Open replacement of aortic valve using prosthetic valve, with cardiopulmonary bypass	0	0	0	1	0	0	1
33406	Open replacement of aortic valve using allograft valve, with cardiopulmonary bypass	0	0	0	1	0	0	1
33410	Open replacement of aortic valve using stentless tissue valve, with cardiopulmonary bypass	0	0	0	1	0	0	1
33411	Replacement of aortic valve, with aortic annulus enlargement using noncoronary sinus	0	0	0	1	0	0	1
33412	Replacement of aortic valve, with transventricular aortic annulus enlargement	0	0	0	1	0	0	1
33413	Replacement of aortic valve by translocation of autologous pulmonary valve with allograft replacement of	0	0	0	1	0	0	1

33414	Repair of left ventricular outflow tract obstruction by patch enlargement of outflow tract	0	0	0	1	0	0	1
33415	Incision of subvalvular tissue for discrete subvalvular aortic stenosis	0	0	0	1	0	0	1
33416	Ventriculomyectomy for idiopathic hypertrophic subaortic stenosis	0	0	0	1	0	0	1
33417	Aortoplasty using gusset for supravalvular stenosis	0	0	0	1	0	0	1
33418	Transcatheter mitral valve repair with prosthetic valve via percutaneous approach	0	0	0	1	0	0	1
33419	Transcatheter mitral valve repair with prosthetic valve via percutaneous approach	0	0	0	1	0	0	1
33420	Closed heart mitral valvotomy	0	0	0	1	0	0	1
33422	Open heart mitral valvotomy with cardiopulmonary bypass	0	0	0	1	0	0	1
33425	Mitral valvuloplasty with cardiopulmonary bypass	0	0	0	1	0	0	1
33426	Mitral valvuloplasty using prosthetic ring, with cardiopulmonary bypass	0	0	0	1	0	0	1
33427	Radical reconstruction of mitral valve using prosthetic ring, with cardiopulmonary bypass	0	0	0	1	0	0	1
33430	Replacement of mitral valve, with cardiopulmonary bypass	0	0	0	1	0	0	1
33460	Tricuspid valvectomy with cardiopulmonary bypass	0	0	0	1	0	0	1
33463	Tricuspid valvuloplasty	0	0	0	1	0	0	1
33464	Tricuspid valvuloplasty using prosthetic ring	0	0	0	1	0	0	1
33465	Replacement of tricuspid valve, with cardiopulmonary bypass	0	0	0	1	0	0	1
33468	Tricuspid valve repositioning and plication for Ebstein anomaly	0	0	0	1	0	0	1
33470	Closed heart pulmonary valvotomy by transventricular approach	0	0	0	1	0	0	1
33471	Closed heart pulmonary valvotomy via pulmonary artery	0	0	0	1	0	0	1
33474	Open heart pulmonary valvotomy with cardiopulmonary bypass	0	0	0	1	0	0	1
33475	Replacement of pulmonary valve	0	0	0	1	0	0	1
33476	Right ventricular resection for infundibular stenosis	0	0	0	1	0	0	1
33477	Percutaneous transcatheter replacement of pulmonary valve with prosthetic valve via transaortic approac	0	0	0	1	0	0	1
33478	Outflow tract augmentation	0	0	0	1	0	0	1
33548	Partial left ventriculectomy	0	0	0	0	0	0	1
33600	Closure of mitral valve using patch	0	0	0	1	0	0	1
33602	Closure of aortic valve using patch	0	0	0	1	0	0	1
33606	Anastomosis of pulmonary artery to aorta	0	0	0	1	0	0	1
33608	Repair of complex cardiac anomaly with ventricular septal defect by construction of conduit from left ventr	0	0	0	1	0	0	1
33610	Repair of complex cardiac anomaly by surgical enlargement of ventricular septal defect	0	0	0	1	0	0	1
33611	Repair of double outlet right ventricle with intraventricular tunnel repair	0	0	0	1	0	0	1
33612	Repair of double outlet right ventricle with intraventricular tunnel repair and repair of right ventricular outfl	0	0	0	1	0	0	1
33615	Repair of complex cardiac anomaly by closure of atrial septal defect and anastomosis of atrium to pulmona	0	0	0	1	0	0	1
33617	Repair of complex cardiac anomalies by modified Fontan procedure	0	0	0	1	0	0	1
33619	Repair of single ventricle heart defect with aortic outflow obstruction and aortic arch hypoplasia	0	0	0	1	0	0	1
33620	Application of right and left pulmonary artery bands	0	0	0	1	0	0	1
33621	Hybrid approach stage 1 procedure for hypoplastic left heart syndrome	0	0	0	1	0	0	1
33622	Reconstruction of complex cardiac anomaly with palliation of single ventricle with aortic outflow obstructio	0	0	0	1	0	0	1
33641	Repair of ostium secundum atrial septal defect using patch, with cardiopulmonary bypass	0	0	0	1	0	0	1
33645	Closure of sinus venosus using patch	0	0	0	1	0	0	1
33647	Closure of atrial septal defect and ventricular septal defect using patch	0	0	0	1	0	0	1
33660	Repair of partial atrioventricular canal	0	0	0	1	0	0	1
33665	Repair of intermediate atrioventricular canal	0	0	0	1	0	0	1
33670	Repair of complete atrioventricular canal	0	0	0	1	0	0	1
33675	Closure of multiple ventricular septal defects	0	0	0	1	0	0	1
33676	Closure of multiple ventricular septal defects with infundibular resection	0	0	0	1	0	0	1
33677	Closure of multiple ventricular septal defects with removal of pulmonary artery band	0	0	0	1	0	0	1
33681	Closure of ventricular septal defect	0	0	0	1	0	0	1
33684	Closure of ventricular septal defect	0	0	0	1	0	0	1
33688	Closure of ventricular septal defect using patch, with removal of pulmonary artery band	0	0	0	1	0	0	1
33690	Banding of pulmonary artery	0	0	0	1	0	0	1
33692	Complete repair of tetralogy of Fallot without pulmonary atresia	0	0	0	1	0	0	1
33694	Complete repair of tetralogy of Fallot without pulmonary atresia using transannular patch	0	0	0	1	0	0	1
33697	Complete repair of tetralogy of Fallot with pulmonary atresia, with construction of conduit from right ventri	0	0	0	1	0	0	1
33702	Repair of sinus of Valsalva fistula, with cardiopulmonary bypass	0	0	0	1	0	0	1
33710	Repair of sinus of Valsalva fistula and ventricular septal defect, with cardiopulmonary bypass	0	0	0	1	0	0	1
33720	Repair of sinus of Valsalva aneurysm with cardiopulmonary bypass	0	0	0	1	0	0	1
33722	Closure of aortico-left ventricular tunnel	0	0	0	1	0	0	1
33724	Repair of isolated partial anomalous pulmonary venous return	0	0	0	1	0	0	1
33726	Repair of pulmonary venous stenosis	0	0	0	1	0	0	1
33730	Complete repair of anomalous pulmonary venous return	0	0	0	1	0	0	1
33732	Repair of cor triatriatum by resection of left atrial membrane	0	0	0	1	0	0	1
33735	Closed heart atrial septectomy	0	0	0	1	0	0	1
33736	Open heart atrial septectomy with cardiopulmonary bypass	0	0	0	1	0	0	1
33737	Open heart atrial septectomy with inflow occlusion	0	0	0	1	0	0	1
33770	Repair of transposition of great arteries with ventricular septal defect and subpulmonary stenosis	0	0	0	1	0	0	1
33771	Repair of transposition of great arteries with ventricular septal defect and subpulmonary stenosis, with sur	0	0	0	1	0	0	1
33774	Repair of transposition of great arteries with atrial baffle procedure and cardiopulmonary bypass	0	0	0	1	0	0	1
33775	Repair of transposition of great arteries with Mustard type atrial baffle procedure and removal of pulmona	0	0	0	1	0	0	1
33776	Repair of transposition of great arteries with Mustard type atrial baffle procedure and closure of ventricula	0	0	0	1	0	0	1

33777	Repair of transposition of great arteries with atrial baffle procedure, repair of subpulmonic obstruction, and	0	0	0	1	0	0	1
33778	Repair of transposition of great arteries with aortic pulmonary artery reconstruction	0	0	0	1	0	0	1
33779	Repair of transposition of great arteries with aortic pulmonary artery reconstruction and removal of pulmo	0	0	0	1	0	0	1
33780	Repair of transposition of great arteries with aortic pulmonary artery reconstruction and closure of ventricu	0	0	0	1	0	0	1
33781	Repair of transposition of great arteries with aortic pulmonary artery reconstruction and repair of subpulmo	0	0	0	1	0	0	1
33782	Aortic root translocation with repair of ventricular septal defect and pulmonary stenosis	0	0	0	1	0	0	1
33783	Aortic root translocation with repair of ventricular septal defect and pulmonary stenosis and with reimplant	0	0	0	1	0	0	1
33786	Complete repair of truncus arteriosus	0	0	0	1	0	0	1
33788	Reimplantation of anomalous pulmonary artery	0	0	0	1	0	0	1
33800	Aortic suspension for tracheal decompression	0	0	0	1	0	0	1
33813	Closure of aortopulmonary septal defect	0	0	0	1	0	0	1
33814	Closure of aortopulmonary septal defect with cardiopulmonary bypass	0	0	0	1	0	0	1
33840	Excision of coarctation of aorta and direct anastomosis of aorta	0	0	0	1	0	0	1
33845	Excision of coarctation of aorta and anastomosis of aorta using graft	0	0	0	1	0	0	1
33851	Excision of coarctation of aorta and repair of aorta using left subclavian artery	0	0	0	1	0	0	1
33852	Repair of hypoplastic aortic arch using autogenous material	0	0	0	1	0	0	1
33853	Repair of hypoplastic aortic arch using autogenous material, with cardiopulmonary bypass	0	0	0	1	0	0	1
33860	Repair of aneurysm of ascending aorta using graft, with cardiopulmonary bypass	0	0	0	0	0	0	1
33863	Repair of aneurysm of ascending aorta using graft,with aortic root replacement using valved conduit and c	0	0	0	0	0	0	1
33864	Repair of aneurysm of ascending aorta with valve suspension, coronary reconstruction and valve-sparing a	0	0	0	0	0	0	1
33870	Repair of aneurysm of transverse aortic arch using graft, with cardiopulmonary bypass	0	0	0	0	0	0	1
33875	Repair of aneurysm descending aorta using graft	0	0	0	0	0	0	1
33877	Repair of aneurysm of thoracoabdominal aortic using graft,	0	0	0	0	0	0	1
33880	Endovascular repair of aneurysm of descending thoracic aorta with coverage of left subclavian artery origi	0	0	0	0	0	0	1
33881	Endovascular repair of descending thoracic aorta	0	0	0	0	0	0	1
33917	Repair of pulmonary artery stenosis by reconstruction using graft	0	0	0	1	0	0	1
33920	Repair of pulmonary atresia with ventricular septal defect, by construction of conduit from left ventricle to p	0	0	0	1	0	0	1
33999	Cardiac surgery procedure	0	0	0	1	0	0	0
38555	Excision of cystic hygroma of axilla with deep neurovascular dissection	0	0	0	1	0	0	1
39503	Repair of hernia of diaphragm	0	0	0	1	0	0	0
39541	Repair of chronic traumatic diaphragmatic hernia	0	0	0	1	0	0	0
39545	Imbrication of diaphragm by transabdominal approach	0	0	0	1	0	0	0
39560	Resection of diaphragm with repair	0	0	0	1	0	0	0
39561	Resection of diaphragm with repair	0	0	0	1	0	0	0
41120	Glossectomy	0	0	0	1	0	0	1
41130	Hemiglossectomy	0	0	0	1	0	0	1
42200	Palatoplasty for cleft hard palate	0	0	0	1	0	0	1
42205	Palatoplasty for cleft palate with closure of alveolar ridge using soft tissue	0	0	0	1	0	0	1
42210	Palatoplasty for cleft palate with closure of alveolar ridge using bone graft	0	0	0	1	0	0	1
42215	Revision of palatoplasty for cleft palate	0	0	0	1	0	0	1
42220	Palatoplasty for cleft palate with secondary lengthening procedure	0	0	0	1	0	0	1
42225	Palatoplasty for cleft palate with attachment of pharyngeal flap	0	0	0	1	0	0	1
42226	Lengthening of palate using pharyngeal flap	0	0	0	1	0	0	1
42299	Procedure on palate	0	0	0	1	0	0	0
42810	Excision of branchial cleft cyst	0	0	0	1	0	0	1
42815	Excision of branchial cleft cyst	0	0	0	1	0	0	1
43647	Laparoscopy, surgical; implantation or replacement of gastric neurostimulator electrodes, antrum	0	0	0	0	0	0	1
43648	Laparoscopy, surgical; revision or removal of gastric neurostimulator electrodes, antrum	0	0	0	0	0	0	1
43881	Implantation or replacement of gastric neurostimulator electrodes, antrum, open	0	0	0	0	0	0	1
43882	Revision or removal of gastric neurostimulator electrodes, antrum, open	0	0	0	0	0	0	1
44050	Laparotomy and reduction of volvulus	0	0	0	1	0	0	1
44055	Correction of malrotation by lysis of duodenal band	0	0	0	1	0	0	1
46715	Repair of low imperforate anus with anoperineal fistula	0	0	0	1	0	0	0
46716	Repair of low imperforate anus with transposition of anoperineal fistula	0	0	0	1	0	0	0
46730	Repair of high imperforate anus by perineal approach	0	0	0	1	0	0	0
46735	Repair of high imperforate anus by combined transabdominal and sacroperineal approaches	0	0	0	1	0	0	0
46740	Repair of high imperforate anus with rectourethral fistula by perineal approach	0	0	0	1	0	0	0
46742	Repair of high imperforate anus with rectourethral fistula by combined transabdominal and sacroperineal a	0	0	0	1	0	0	0
46744	Repair of cloacal anomaly by anorectovaginoplasty and urethroplasty by sacroperineal approach	0	0	0	1	0	0	0
46746	Repair of cloacal anomaly by anorectovaginoplasty and urethroplasty by combined abdominal and sacrop	0	0	0	1	0	0	0
46748	Repair of cloacal anomaly by anorectovaginoplasty and urethroplasty by combined abdominal and sacrop	0	0	0	1	0	0	0
46750	Anal sphincteroplasty	0	0	0	1	0	0	0
46751	Anal sphincteroplasty	0	0	0	1	0	0	0
47000	Percutaneous needle biopsy of liver	0	0	0	1	0	0	0
47001	Needle biopsy of liver	0	0	0	1	0	0	0
47100	Wedge biopsy of liver	0	0	0	1	0	0	0
48140	Distal subtotal pancreatectomy	0	0	0	1	0	0	0
48145	Distal subtotal pancreatectomy with pancreaticojejunostomy	0	0	0	1	0	0	0
48146	Distal near-total pancreatectomy with preservation of duodenum	0	0	0	1	0	0	0
49600	Repair of omphalocele with primary closure	0	0	0	1	0	0	0

49605	Repair of gastroschisis	0	0	0	1	0	0	0
49606	Repair of gastroschisis with removal of prosthesis, final reduction and closure	0	0	0	1	0	0	0
49610	First stage repair of omphalocele	0	0	0	1	0	0	0
49611	Second stage repair of omphalocele	0	0	0	1	0	0	0
54304	Plastic operation on penis for first stage hypospadias repair	0	0	0	1	0	0	1
54308	Urethroplasty for second stage hypospadias repair	0	0	0	1	0	0	1
54312	Urethroplasty for second stage hypospadias repair	0	0	0	1	0	0	1
54316	Urethroplasty for second stage hypospadias repair using free skin graft	0	0	0	1	0	0	1
54318	Urethroplasty for third stage hypospadias repair to release penis from scrotum	0	0	0	1	0	0	1
54322	Repair of distal hypospadias and chordee with meatal advancement	0	0	0	1	0	0	1
54324	Repair of distal hypospadias and chordee with urethroplasty using local skin flap	0	0	0	1	0	0	1
54326	Repair of distal hypospadias and chordee with urethroplasty using local skin flap and mobilization of urethr	0	0	0	1	0	0	1
54328	1-stage repair of distal hypospadias with extensive dissection for chordee and urethroplasty using island fl	0	0	0	1	0	0	1
54332	Repair of penile hypospadias repair	0	0	0	1	0	0	1
54336	Repair of perineal hypospadias with dissection	0	0	0	1	0	0	1
54340	Repair of complication after hypospadias repair	0	0	0	1	0	0	1
54344	Repair of complication after hypospadias repair	0	0	0	1	0	0	1
54352	Repair of hypospadias cripple	0	0	0	1	0	0	1
54380	Plastic operation on penis for epispadias	0	0	0	1	0	0	1
54385	Plastic operation on penis for epispadias with incontinence	0	0	0	1	0	0	1
54390	Plastic operation on penis for epispadias with exstrophy of bladder	0	0	0	1	0	0	1
54550	Exploration of scrotal area for undescended testis	0	0	0	1	0	0	1
54560	Abdominal exploration for undescended testis	0	0	0	1	0	0	1
54640	Orchiopexy by inguinal approach	0	0	0	1	0	0	1
54650	Orchiopexy of intra-abdominal testis by abdominal approach	0	0	0	1	0	0	1
55175	Scrotoplasty	0	0	0	1	0	0	0
55180	Scrotoplasty	0	0	0	1	0	0	0
55970	Male to female intersex surgery	0	0	0	1	0	0	0
55980	Female to male intersex surgery	0	0	0	1	0	0	0
61850	Twist drill or burr hole(s) for implantation of neurostimulator electrodes, cortical	0	0	0	0	0	0	1
61860	Craniectomy or craniotomy for implantation of neurostimulator electrodes, cerebral, cortical	0	0	0	0	0	0	1
61863	Twist drill, burr hole, craniotomy, or craniectomy with stereotactic implantation of neurostimulator electrod	0	0	0	0	0	0	1
61864	Twist drill, burr hole, craniotomy, or craniectomy with stereotactic implantation of neurostimulator electrod	0	0	0	0	0	0	1
61867	Twist drill, burr hole, craniotomy, or craniectomy with stereotactic implantation of neurostimulator electrod	0	0	0	0	0	0	1
61868	Twist drill, burr hole, craniotomy, or craniectomy with stereotactic implantation of neurostimulator electrod	0	0	0	0	0	0	1
61870	Craniectomy for implantation of neurostimulator electrodes, cerebellar, cortical	0	0	0	0	0	0	1
61880	Revision or removal of intracranial neurostimulator electrodes	0	0	0	0	0	0	1
61885	Insertion or replacement of cranial neurostimulator pulse generator or receiver, direct or inductive coupling	0	0	0	0	0	0	1
61886	Insertion or replacement of cranial neurostimulator pulse generator or receiver, direct or inductive coupling	0	0	0	0	0	0	1
61888	Revision or removal of cranial neurostimulator pulse generator or receiver	0	0	0	0	0	0	1
63650	Percutaneous implantation of neurostimulator electrode array, epidural	0	0	0	0	0	0	1
63655	Laminectomy for implantation of neurostimulator electrodes, plate/paddle, epidural	0	0	0	0	0	0	1
63661	Removal of spinal neurostimulator electrode percutaneous array(s), including fluoroscopy, when performe	0	0	0	0	0	0	1
63662	Removal of spinal neurostimulator electrode plate/paddle(s) placed via laminotomy or laminectomy, inclu	0	0	0	0	0	0	1
63663	Revision including replacement, when performed, of spinal neurostimulator electrode percutaneous array(	0	0	0	0	0	0	1
63664	Revision including replacement, when performed, of spinal neurostimulator electrode plate/paddle(s) plac	0	0	0	0	0	0	1
63685	Insertion or replacement of spinal neurostimulator pulse generator or receiver, direct or inductive coupling	0	0	0	0	0	0	1
63688	Revision or removal of implanted spinal neurostimulator pulse generator or receiver	0	0	0	0	0	0	1
64550	Application of surface (transcutaneous) neurostimulator (eg, TENS unit)	0	0	0	0	0	0	1
64553	Percutaneous implantation of neurostimulator electrode array; cranial nerve	0	0	0	0	0	0	1
64555	Percutaneous implantation of neurostimulator electrode array; peripheral nerve (excludes sacral nerve)	0	0	0	0	0	0	1
64561	Percutaneous implantation of neurostimulator electrode array; sacral nerve (transforaminal placement) in	0	0	0	0	0	0	1
64566	Posterior tibial neurostimulation, percutaneous needle electrode, single treatment, includes programming	0	0	0	0	0	0	1
64568	Incision for implantation of cranial nerve (eg, vagus nerve) neurostimulator electrode array and pulse gene	0	0	0	0	0	0	1
64569	Revision or replacement of cranial nerve (eg, vagus nerve) neurostimulator electrode array, including conn	0	0	0	0	0	0	1
64570	Removal of cranial nerve (eg, vagus nerve) neurostimulator electrode array and pulse generator	0	0	0	0	0	0	1
64575	Incision for implantation of neurostimulator electrode array; peripheral nerve (excludes sacral nerve)	0	0	0	0	0	0	1
64580	Incision for implantation of neurostimulator electrode array; neuromuscular	0	0	0	0	0	0	1
64581	Incision for implantation of neurostimulator electrode array; sacral nerve (transforaminal placement)	0	0	0	0	0	0	1
64585	Revision or removal of peripheral neurostimulator electrode array	0	0	0	0	0	0	1
64590	Insertion or replacement of peripheral or gastric neurostimulator pulse generator or receiver, direct or indu	0	0	0	0	0	0	1
64595	Revision or removal of peripheral or gastric neurostimulator pulse generator or receiver	0	0	0	0	0	0	1
64788	Excision of neurofibroma of cutaneous nerve	0	0	0	0	0	0	1
64792	Excision of neurofibroma	0	0	1	0	0	1	0
64795	Biopsy of nerve	0	0	0	0	0	0	1
66825	Repositioning of intraocular lens prosthesis with limbal incision	0	0	0	1	0	0	1
66830	Removal of secondary membranous cataract with corneo-scleral section	0	0	0	1	0	0	1
66840	Removal of lens material by aspiration	0	0	0	1	0	0	1
66850	Removal of lens material by mechanical phacofragmentation and aspiration	0	0	0	1	0	0	1
66852	Removal of lens material by pars plana approach	0	0	0	1	0	0	1

66920	Intracapsular removal of lens material	0	0	0	1	0	0	1
66930	Intracapsular removal of dislocated lens	0	0	1	0	0	1	0
66940	Extracapsular removal of lens material	0	0	1	0	0	0	1
66982	Extracapsular cataract removal by manual technique with insertion of intraocular lens prosthesis	0	0	1	0	0	0	1
66983	Intracapsular cataract extraction with insertion of intraocular lens prosthesis	0	0	1	0	0	0	1
66984	Extracapsular cataract removal by manual technique with insertion of intraocular lens prosthesis	0	0	1	0	0	0	1
66985	Insertion of intraocular lens prosthesis	0	0	1	0	0	0	1
66986	Exchange of intraocular lens	0	0	0	1	0	0	1
75557	Cardiac MRI	0	0	0	1	0	0	0
75561	Cardiac MRI	0	0	0	1	0	0	0
75565	Cardiac MRI	0	0	0	1	0	0	0
81105	Human Platelet Antigen 1 genotyping (HPA-1), ITGB3 (integrin, beta 3 [platelet glycoprotein IIIa], antigen	1	0	0	0	0	0	0
81106	Human Platelet Antigen 2 genotyping (HPA-2), GP1BA (glycoprotein Ib [platelet], alpha polypeptide [GPIba	1	0	0	0	0	0	0
81107	Human Platelet Antigen 3 genotyping (HPA-3), ITGA2B (integrin, alpha 2b [platelet glycoprotein IIb of IIb/	1	0	0	0	0	0	0
81108	Human Platelet Antigen 4 genotyping (HPA-4), ITGB3 (integrin, beta 3 [platelet glycoprotein IIIa], antigen	1	0	0	0	0	0	0
81109	Human Platelet Antigen 5 genotyping (HPA-5), ITGA2 (integrin, alpha 2 [CD49B, alpha 2 subunit of VLA-2 r	1	0	0	0	0	0	0
81110	Human Platelet Antigen 6 genotyping (HPA-6w), ITGB3 (integrin, beta 3 [platelet glycoprotein IIIa, antigen	1	0	0	0	0	0	0
81111	Human Platelet Antigen 9 genotyping (HPA-9w), ITGA2B (integrin, alpha 2b [platelet glycoprotein IIb of IIb	1	0	0	0	0	0	0
81112	Human Platelet Antigen 15 genotyping (HPA-15), CD109 (CD109 molecule) (eg, neonatal alloimmune thro	1	0	0	0	0	0	0
81161	DMD (dystrophin) deletion and duplication analysis	1	0	0	0	0	0	0
81162	Breast cancer 1 and 2 (BRCA1, BRCA2) full sequence gene analysis and analysis of full duplication and delet	1	0	0	0	0	0	0
81175	Additional sex combs like 1, transcriptional regulator (ASXL1) full gene sequence analysis	1	0	0	0	0	0	0
81176	Additional sex combs like 1, transcriptional regulator (ASXL1) targeted sequence analysis	1	0	0	0	0	0	0
81200	Aspartoacylase (ASPA) gene analysis for detection of common variant	1	0	0	0	0	0	0
81201	Adenomatous polyposis coli (APC) full gene sequence analysis	1	0	0	0	0	0	0
81202	Adenomatous polyposis coli (APC) gene analysis for known familial variants	1	0	0	0	0	0	0
81203	Adenomatous polyposis coli (APC) gene analysis for deletion variant	1	0	0	0	0	0	0
81205	Branched-chain keto acid dehydrogenase E1, beta polypeptide (BCKDHB) gene analysis for detection of co	1	0	0	0	0	0	0
81209	Bloom syndrome, RecQ helicase-like (BLM) gene analysis for detection of 2281del6ins7 variant	1	0	0	0	0	0	0
81211	Breast cancer 1 (BRCA1) gene analysis for detection of common deletion variant	1	0	0	0	0	0	0
81212	Breast cancer 1 and 2 (BRCA1, BRCA2) gene analysis for detection of 185delAG variant	1	0	0	0	0	0	0
81213	Breast cancer 1 and 2 (BRCA1, BRCA2) gene analysis for detection of uncommon deletion variant	1	0	0	0	0	0	0
81214	Breast cancer 1 (BRCA1) full sequence analysis gene analysis	1	0	0	0	0	0	0
81215	Breast cancer 1 (BRCA1) gene analysis for detection of known familial variant	1	0	0	0	0	0	0
81216	Breast cancer 2 (BRCA2) full gene sequence analysis	1	0	0	0	0	0	0
81217	Breast cancer 2 (BRCA2) gene analysis for detection of known familial variant	1	0	0	0	0	0	0
81218	CCAAT/enhancer binding protein [C/EBP], alpha (CEBPA) full gene sequence analysis	1	0	0	0	0	0	0
81219	Calreticulin (CALR) gene analysis for detection of common variant on exon 9	1	0	0	0	0	0	0
81220	Cystic fibrosis transmembrane conductance regulator (CFTR) gene analysis for detection of common varia	1	0	0	0	0	0	0
81221	Cystic fibrosis transmembrane conductance regulator (CFTR) gene analysis for detection of known familial	1	0	0	0	0	0	0
81222	Cystic fibrosis transmembrane conductance regulator (CFTR) gene analysis for detection of deletion varian	1	0	0	0	0	0	0
81223	Cystic fibrosis transmembrane conductance regulator (CFTR) full sequence gene analysis	1	0	0	0	0	0	0
81224	Cystic fibrosis transmembrane conductance regulator (CFTR) gene analysis with intron 8 poly-T analysis	1	0	0	0	0	0	0
81228	Cytogenomic constitutional microarray analysis	1	0	0	0	0	0	0
81229	Cytogenomic constitutional microarray analysis	1	0	0	0	0	0	0
81238	F9 (coagulation factor IX) (eg, hemophilia B), full gene sequence	1	0	0	0	0	0	0
81240	F2 (prothrombin, coagulation factor II) (eg, hereditary hypercoagulability) gene analysis, 20210G>A varia	1	0	0	0	0	0	0
81241	F5 (coagulation factor V) (eg, hereditary hypercoagulability) gene analysis, Leiden variant	1	0	0	0	0	0	0
81242	Fanconi anemia, complementation group C (FANCC) gene analysis for detection of common variant	1	0	0	0	0	0	0
81243	Fragile X mental retardation 1 (FMR1) gene analysis for detection of abnormal allele	1	0	0	0	0	0	0
81244	Fragile X mental retardation 1 (FMR1) gene analysis for characterization of allele	1	0	0	0	0	0	0
81247	G6PD (glucose-6-phosphate dehydrogenase) gene analysis for detection of A variant	1	0	0	0	0	0	0
81248	Glucose-6-phosphate dehydrogenase (G6PD) gene analysis for detection of known familial variant	1	0	0	0	0	0	0
81249	Glucose-6-phosphate dehydrogenase (G6PD) full gene sequence analysis	1	0	0	0	0	0	0
81250	Glucose-6-phosphatase, catalytic subunit (G6PC) gene analysis for detection of common variant	1	0	0	0	0	0	0
81251	Glucosidase, beta, acid (GBA) gene analysis for detection of common variant	1	0	0	0	0	0	0
81252	Gap junction protein, beta 2, 26kDa; connexin 26 (GJB6) full gene sequence analysis	1	0	0	0	0	0	0
81253	Gap junction protein, beta 2, 26kDa; connexin 26 (GJB6) for detection of known familial variants	1	0	0	0	0	0	0
81254	Gap junction protein, beta 6, 30kDa, connexin 30 (GJB6) gene analysis for detection of 232kb [del(GJB6-D1	1	0	0	0	0	0	0
81255	Hexosaminidase A (alpha polypeptide) (HEXA) gene analysis for detection of common variant	1	0	0	0	0	0	0
81256	Hemochromatosis (HFE) gene analysis for detection of C282Y and H63D variants	1	0	0	0	0	0	0
81257	Alpha globin 1 and alpha globin 2 (HBA1/HBA2) gene analysis for detection of common deletion	1	0	0	0	0	0	0
81258	Alpha globin 1 and alpha globin 2 (HBA1/HBA2) gene analysis for detection of known familial variant	1	0	0	0	0	0	0
81259	Alpha globin 1 and alpha globin 2 (HBA1/HBA2) gene analysis of full gene sequence	1	0	0	0	0	0	0
81260	Inhibitor of kappa light polypeptide gene enhancer in B-cells, kinase complex-associated protein (IKBKAP)	1	0	0	0	0	0	0
81265	Comparative analysis using Short Tandem Repeat (STR) marker	1	0	0	0	0	0	0
81266	Comparative analysis using Short Tandem Repeat (STR) marker	1	0	0	0	0	0	0
81269	Alpha globin 1 and alpha globin 2 (HBA1/HBA2) gene analysis for detection of duplication and deletion var	1	0	0	0	0	0	0
81290	Mucolipidosis, type IV (MCOLN1) gene analysis for detection of common variant	1	0	0	0	0	0	0
81292	MutL homolog 1, colon cancer, nonpolyposis type 2 (MLH1) full gene sequence analysis	1	0	0	0	0	0	0

81293	MutL homolog 1, colon cancer, nonpolyposis type 2 (MLH1) gene analysis for detection of known familial	1	0	0	0	0	0	0
81294	MutL homolog 1, colon cancer, nonpolyposis type 2) (MLH1) gene analysis for detection of deletion and du	1	0	0	0	0	0	0
81295	MutS homolog 2, colon cancer, nonpolyposis type 1 (MSH2) full sequence gene analysis	1	0	0	0	0	0	0
81296	MutS homolog 2, colon cancer, nonpolyposis type 1 (MSH2) gene analysis for detection of known familial	1	0	0	0	0	0	0
81297	MutS homolog 2, colon cancer, nonpolyposis type 1 (MSH2) gene analysis for detection of deletion variant	1	0	0	0	0	0	0
81298	MutS homolog 6 (E. coli) (MSH6) full gene sequence analysis	1	0	0	0	0	0	0
81299	MutS homolog 6 (E. coli) (MSH6) gene analysis for detection of known familial variant	1	0	0	0	0	0	0
81300	MutS homolog 6 (E. coli) (MSH6) gene analysis for detection of deletion variant	1	0	0	0	0	0	0
81302	Methyl CpG binding protein 2 (MECP2) full gene sequence analysis	1	0	0	0	0	0	0
81303	Methyl CpG binding protein 2 (MECP2) gene analysis for detection of known familial variant	1	0	0	0	0	0	0
81304	Methyl CpG binding protein 2 (MECP2) gene analysis for detection of deletion variant	1	0	0	0	0	0	0
81317	Postmeiotic segregation increased 2 (S. cerevisiae) (PMS2) full gene sequence analysis	1	0	0	0	0	0	0
81318	Postmeiotic segregation increased 2 gene analysis for detection of known familial variant	1	0	0	0	0	0	0
81319	Postmeiotic segregation increased 2 (PMS2) gene analysis for detection of deletion variant	1	0	0	0	0	0	0
81321	Phosphatase and tensin homolog (PTEN) full gene sequence analysis	1	0	0	0	0	0	0
81322	Phosphatase and tensin homolog (PTEN) gene analysis for known familial variant	1	0	0	0	0	0	0
81323	Phosphatase and tensin homolog (PTEN) gene analysis for deletion and duplication variants	1	0	0	0	0	0	0
81324	Peripheral myelin protein 22 (PMP22) gene analysis for deletion and duplication variants	1	0	0	0	0	0	0
81325	Peripheral myelin protein 22 (PMP22) full gene sequence analysis	1	0	0	0	0	0	0
81326	Peripheral myelin protein 22 (PMP22) gene analysis for known familial variant	1	0	0	0	0	0	0
81330	Sphingomyelin phosphodiesterase 1, acid lysosomal (SMPD1) gene analysis for detection of common vari	1	0	0	0	0	0	0
81332	Serpin peptidase inhibitor, clade A, alpha-1 antiproteinase, antitrypsin, member 1 (SERPINA1) gene analys	1	0	0	0	0	0	0
81334	Runt related transcription factor 1 (RUNX1) gene analysis of exons 3-8	1	0	0	0	0	0	0
81361	Hemoglobin, subunit beta (HBB) gene analysis for detection of HbC variant	1	0	0	0	0	0	0
81362	Hemoglobin, subunit beta (HBB) gene analysis for detection of known familial variant	1	0	0	0	0	0	0
81363	Hemoglobin, subunit beta (HBB) gene analysis for detection of deletion and duplication variants	1	0	0	0	0	0	0
81364	HBB (hemoglobin, subunit beta) full gene sequence analysis	1	0	0	0	0	0	0
81400	Level 1 molecular pathology procedure	1	0	0	0	0	0	0
81401	Level 2 molecular pathology procedure	1	0	0	0	0	0	0
81402	Level 3 molecular pathology procedure	1	0	0	0	0	0	0
81403	Level 4 molecular pathology procedure	1	0	0	0	0	0	0
81404	Level 5 molecular pathology procedure	1	0	0	0	0	0	0
81405	Level 6 molecular pathology procedure	1	0	0	0	0	0	0
81406	Level 7 molecular pathology procedure	1	0	0	0	0	0	0
81407	Level 8 molecular pathology procedure	1	0	0	0	0	0	0
81408	Level 9 molecular pathology procedure	1	0	0	0	0	0	0
81410	Aortic dilation gene deletion analysis for detection of FBN1, TGFBR1, TGFBR2, COL3A1, MYH11, ACTA2, SL	1	0	0	0	0	0	0
81411	Aortic dilation gene deletion analysis for detection of TGFBR1, TGFBR2, MYH11, and COL3A1 variants	1	0	0	0	0	0	0
81412	Ashkenazi jewish associated disorders genomic sequence analysis panel	1	0	0	0	0	0	0
81413	Cardiac ion channelopathies duplication/deletion gene analysis panel with sequencing of 10 or more gene	1	0	0	0	0	0	0
81414	Cardiac ion channelopathies duplication/deletion gene analysis panel with analysis of at least genes, inclu	1	0	0	0	0	0	0
81415	Exome sequence analysis	1	0	0	0	0	0	0
81416	Exome sequence analysis	1	0	0	0	0	0	0
81417	Exome sequence analysis	1	0	0	0	0	0	0
81425	Genome sequence analysis	1	0	0	0	0	0	0
81426	Genome sequence analysis	1	0	0	0	0	0	0
81427	Genome sequence analysis	1	0	0	0	0	0	0
81430	Hearing loss genomic sequence analysis panel including CDH23, CLRN1, GJB2, GPR98, MTRNR1, MYO7A,	1	0	0	0	0	0	0
81431	Hearing loss gene analysis for detection of deletion and duplication variants including STRC and DFNB1 del	1	0	0	0	0	0	0
81432	Hereditary breast cancer related disorders genomic sequence analysis	1	0	0	0	0	0	0
81433	Breast cancer (BRCA1, BRCA2, MLH1, MSH2, STK11) gene analysis for detection of duplication and deletio	1	0	0	0	0	0	0
81434	Retinal disorders (ABCA4, CNGA1, CRB1, EYS, PDE6A, PDE6B, PRPF31, PRPH2, RDH12, RHO, RP1) genomic s	1	0	0	0	0	0	0
81435	Hereditary colon cancer syndrome genomic sequence analysis panel including APC, CHEK2, MLH1, MSH2,	1	0	0	0	0	0	0
81436	Hereditary colon cancer syndrome gene analysis for detection of deletion and duplication variants includin	1	0	0	0	0	0	0
81437	Neuroendocrine tumor disorders (MAX, SDHB, SDHC, SDHD, TMEM127) genomic sequence analysis	1	0	0	0	0	0	0
81438	Neuroendocrine tumor disorders (SDHB, SDHC, SDHD, VHL) gene analysis for detection of duplication and	1	0	0	0	0	0	0
81439	Hereditary cardiomyopathy genomic sequence analysis	1	0	0	0	0	0	0
81440	Mitochondrially encoded genomic sequence panel for detection of BCS1L, C10orf2, COQ2, COX10, DGUOK,	1	0	0	0	0	0	0
81442	Noonan spectrum disorders genomic sequence analysis panel including BRAF, CBL, HRAS, KRAS, MAP2K1,	1	0	0	0	0	0	0
81448	Hereditary peripheral neuropathies (eg, Charcot-Marie-Tooth, spastic paraplegia), genomic sequence anal	1	0	0	0	0	0	0
81460	Mitochondrially encoded genomic sequence analysis with detection of heteroplasmy	1	0	0	0	0	0	0
81465	Mitochondrially encoded genomic sequence analysis for detection of deletion and heteroplasmy variants	1	0	0	0	0	0	0
81470	X-linked intellectual disability (XLID) genomic sequence analysis panel including ARX, ATRX, CDKL5, FGD1,	1	0	0	0	0	0	0
81471	X-linked intellectual disability (XLID) gene analysis for detection of deletion and duplication variants includi	1	0	0	0	0	0	0
81479	Molecular pathology procedure	1	0	0	0	0	0	0
82485	Measurement of chondroitin B sulfate	0	0	0	1	0	0	1
82657	Assay of enzyme activity in blood cells	0	0	0	1	0	0	1
82658	Assay of enzyme activity in blood cells with radioactive substrate	0	0	0	1	0	0	1
82963	Measurement of beta glucosidase	0	0	1	0	0	1	0
83080	Measurement of b-hexosaminidase	0	0	1	0	0	1	0

88130	Identification of Barr bodies	0	0	0	1	0	0	1
88245	Chromosome analysis for breakage syndrome	1	0	0	0	0	0	0
88248	Chromosome analysis for breakage syndrome	1	0	0	0	0	0	0
88249	Chromosome analysis for breakage syndrome using clastogen stress	1	0	0	0	0	0	0
88261	Chromosome analysis	1	0	0	0	0	0	0
88262	Chromosome analysis	1	0	0	0	0	0	0
88263	Chromosome analysis for mosaicism	1	0	0	0	0	0	0
88264	Chromosome analysis	1	0	0	0	0	0	0
88271	Molecular cytogenetics using DNA probe	1	0	0	0	0	0	0
88272	Molecular cytogenetics using chromosomal in situ hybridization	1	0	0	0	0	0	0
88273	Molecular cytogenetics using chromosomal in situ hybridization	1	0	0	0	0	0	0
88274	Molecular cytogenetics using interphase in situ hybridization	1	0	0	0	0	0	0
88275	Molecular cytogenetics using interphase in situ hybridization	1	0	0	0	0	0	0
88280	Chromosome analysis	1	0	0	0	0	0	0
88283	Chromosome analysis using specialized banding technique	1	0	0	0	0	0	0
88285	Chromosome analysis	1	0	0	0	0	0	0
88289	High resolution chromosome analysis	1	0	0	0	0	0	0
88291	Interpretation and report of cytogenetic and molecular cytogenetic studies	1	0	0	0	0	0	0
88299	Cytogenetic study	1	0	0	0	0	0	0
88305	Surgical pathology with gross and microscopic examination of specimen from biopsy of muscle	0	0	0	1	0	0	1
88307	Surgical pathology with gross and microscopic examination of specimen from enucleation of eye	0	0	0	1	0	0	0
88355	Morphometric analysis of skeletal muscle	0	0	0	1	0	0	1
89049	Caffeine halothane contracture test (CHCT) for malignant hyperthermia susceptibility	0	0	0	0	0	0	1
93462	Left heart catheterization by transseptal puncture through intact septum	0	0	0	1	0	0	1
93505	Endomyocardial biopsy	0	0	0	1	0	0	1
93530	Right heart catheterization	0	0	0	1	0	0	1
93531	Combined right heart catheterization and retrograde left heart catheterization	0	0	0	1	0	0	1
93532	Combined right heart catheterization and transseptal left heart catheterization through intact septum	0	0	0	1	0	0	1
93533	Combined right heart catheterization and transseptal left heart catheterization through existing septal oper	0	0	0	1	0	0	1
93581	Percutaneous transcatheter closure of a congenital ventricular septal defect with implant	0	0	0	1	0	0	1
93582	Percutaneous transcatheter closure of patent ductus arteriosus	0	0	0	0	0	0	1
93583	Percutaneous transcatheter closure of cardiac septa	0	0	0	1	0	0	1
93600	Bundle of His recording	0	0	0	1	0	0	1
93602	Intra-atrial recording	0	0	0	1	0	0	1
93603	Right ventricular recording	0	0	0	1	0	0	1
93609	Intra-atrial mapping of tachycardia sites with catheter manipulation to record from multiple sites to identify	0	0	0	1	0	0	1
93610	Intra-atrial pacing	0	0	0	1	0	0	1
93612	Intraventricular pacing	0	0	0	1	0	0	1
93613	Intracardiac electrophysiologic 3-dimensional mapping	0	0	0	1	0	0	1
93615	Esophageal recording of atrial electrogram	0	0	0	1	0	0	1
93616	Esophageal recording of atrial electrogram	0	0	0	1	0	0	1
93618	Induction of arrhythmia by electrical pacing	0	0	0	1	0	0	1
93619	Comprehensive electrophysiologic evaluation with right atrial pacing and recording, right ventricular pacing	0	0	0	1	0	0	1
93620	Comprehensive electrophysiologic evaluation including insertion and repositioning of multiple electrode ca	0	0	0	1	0	0	1
93621	Comprehensive electrophysiologic evaluation including insertion and repositioning of multiple electrode ca	0	0	0	1	0	0	1
93622	Comprehensive electrophysiologic evaluation including insertion and repositioning of multiple electrode ca	0	0	0	1	0	0	1
93623	Programmed stimulation and pacing after intravenous drug infusion	0	0	0	1	0	0	1
93624	Electrophysiologic follow-up study with pacing and recording to test effectiveness of therapy, including att	0	0	0	1	0	0	1
93631	Intra-operative epicardial and endocardial pacing and mapping to localize the site of tachycardia or zone o	0	0	0	1	0	0	1
93640	Electrophysiologic evaluation of dual chamber pacing cardioverter-defibrillator leads including defibrillatior	0	0	0	1	0	0	1
93641	Electrophysiologic evaluation of dual chamber pacing cardioverter-defibrillator leads including defibrillatior	0	0	0	1	0	0	1
93642	Electrophysiologic evaluation of dual chamber pacing cardioverter-defibrillator	0	0	0	1	0	0	1
93644	Electrophysiologic evaluation of subcutaneous implantable defibrillator	0	0	0	1	0	0	1
93650	Intracardiac catheter ablation of atrioventricular node function, atrioventricular conduction for creation of c	0	0	0	1	0	0	1
93653	Comprehensive electrophysiologic evaluation including insertion and repositioning of multiple electrode ca	0	0	0	1	0	0	1
93654	Comprehensive electrophysiologic evaluation including insertion and repositioning of multiple electrode ca	0	0	0	1	0	0	1
93655	Intracardiac catheter ablation of arrhythmia	0	0	0	1	0	0	1
93656	Comprehensive electrophysiologic evaluation including transseptal catheterization and repositioning of mu	0	0	0	1	0	0	1
93657	Focal intracardiac catheter ablation of left atrium	0	0	0	1	0	0	1
93660	Evaluation of cardiovascular function with tilt table evaluation, with continuous ECG monitoring and interm	0	0	0	1	0	0	1
93662	Intracardiac echocardiography during diagnostic intervention including imaging supervision and interpreta	0	0	0	1	0	0	1
93668	Peripheral arterial disease rehabilitation	0	0	0	1	0	0	1
93701	Bioimpedance-derived physiologic cardiovascular analysis	0	0	0	1	0	0	1
93702	Bioimpedance-derived spectroscopy for extracellular fluid analysis	0	0	0	1	0	0	1
93724	Electronic analysis of antitachycardia pacemaker system	0	0	0	1	0	0	1
93745	Initial set-up and programming by a physician of wearable cardioverter-defibrillator includes initial prograr	0	0	0	1	0	0	1
93750	Interrogation of ventricular assist device with physician analysis of device parameters and review of devic	0	0	0	1	0	0	1
95812	EEG extended monitoring	0	0	0	1	0	0	1
95813	EEG extended monitoring	0	0	0	1	0	0	1
95816	EEG including recording awake and drowsy	0	0	0	1	0	0	1

95819	EEG including recording awake and asleep	0	0	0	1	0	0	1
95822	EEG recording in coma or sleep only	0	0	0	1	0	0	1
95824	EEG cerebral death evaluation only	0	0	0	1	0	0	1
95827	EEG all night recording	0	0	0	1	0	0	1
95860	Needle electromyography of extremity	0	0	0	1	0	0	1
95861	Needle electromyography of extremities	0	0	0	1	0	0	1
95863	Needle electromyography of extremities	0	0	0	1	0	0	1
95864	Needle electromyography of extremities	0	0	0	1	0	0	1
95872	Needle electromyography using single fiber electrode, with quantitative measurement of jitter and blockin	0	0	0	1	0	0	1
95885	Needle electromyography of extremity with nerve conduction, amplitude and latency study	0	0	0	1	0	0	1
95886	Needle electromyography of extremity with nerve conduction, amplitude and latency study	0	0	0	1	0	0	1
95887	Needle electromyography of non-extremity muscles done with nerve conduction, amplitude and latency st	0	0	0	1	0	0	1
95905	Motor nerve conduction, using preconfigured electrode arrays, amplitude and latency study of each limb w	0	0	0	1	0	0	1
95907	Nerve conduction study	0	0	0	1	0	0	1
95908	Nerve conduction study	0	0	0	1	0	0	1
95909	Nerve conduction study	0	0	0	1	0	0	1
95910	Nerve conduction study	0	0	0	1	0	0	1
95911	Nerve conduction study	0	0	0	1	0	0	1
95912	Nerve conduction study	0	0	0	1	0	0	1
95913	Nerve conduction study	0	0	0	1	0	0	1
95930	Visual evoked potential testing of central nervous system using checkerboard, with interpretation and repo	0	0	0	1	0	0	1
95970	Electronic analysis of implanted neurostimulator pulse generator system	0	0	0	0	0	0	1
95971	Electronic analysis of implanted neurostimulator pulse generator system	0	0	0	0	0	0	1
95972	Electronic analysis of implanted neurostimulator pulse generator system	0	0	0	0	0	0	1
95974	Electronic analysis of implanted neurostimulator pulse generator system	0	0	0	0	0	0	1
95975	Electronic analysis of implanted neurostimulator pulse generator system	0	0	0	0	0	0	1
95978	Electronic analysis of implanted neurostimulator pulse generator system	0	0	0	0	0	0	1
95979	Electronic analysis of implanted neurostimulator pulse generator system	0	0	0	0	0	0	1
95980	Electronic analysis of implanted neurostimulator pulse generator system	0	0	0	0	0	0	1
95981	Electronic analysis of implanted neurostimulator pulse generator system	0	0	0	0	0	0	1
95982	Electronic analysis of implanted neurostimulator pulse generator system	0	0	0	0	0	0	1
95999	Neuromuscular diagnostic procedure	0	0	0	0	0	0	1
96040	Medical genetics and genetic counseling services	0	0	1	0	0	0	1

HCPCS	Description	Genetic Test	Newborn_Definite	Newborn_Probable	Newborn_Possible	Ped_Definite	Ped_Probable	Ped_Possible
B4154	Enteral formula, nutritionally complete, for special metabolic needs, excludes inherited disease of metab	0	0	0	0	0	0	0
B4157	Enteral formula, nutritionally complete, for special metabolic needs for inherited disease of metabolism,	0	1	0	0	1	0	0
B4162	Enteral formula, for pediatrics, special metabolic needs for inherited disease of metabolism, includes pr	0	1	0	0	1	0	0
C1005	Intraocular lens, Sensar Soft Acrylic Ultraviolet Light Absorbing Posterior Chamber Intraocular Lens	0	0	0	1	0	0	1
C1006	Intraocular lens, array multifocal silicone posterior chamber intraocular lens	0	0	0	1	0	0	1
C1102	Generator, pulse, neurostimulator, medtronic synergy neurostimulator generator and extension	0	0	0	1	0	0	1
C1106	Neurostimulator, patient programmer, synergy ez patient programmer	0	0	0	1	0	0	1
C1124	Lead, neurostimulator, kit, interstim test stimulation lead kit	0	0	0	1	0	0	1
C1306	Lead, neurostimulator, cyberonics neurocybernetic prosthesis lead, octad lead 3898-33/389861, on-point model 3987, pisces-quad plus model 3888, resume tl model 3986, pisces-qu	0	0	0	1	0	0	1
C1353	Neurostimulator, implantable, itrel ii/soletra implantable neurostimulator and extension, itrel iii implantable neurostimulator and extension, interstim neurostimulator (implantable) and	0	0	0	1	0	0	1
C1376	Lead, neurostimulator, ans renew spinal cord stimulation system lead (with or without extension)	0	0	0	1	0	0	1
C1377	Lead, neurostimulator, specify 3988 lead	0	0	0	1	0	0	1
C1378	Lead, neurostimulator, inerstim therapy 3080 lead, interstim therapy 3886 lead	0	0	0	1	0	0	1
C1379	Lead, neurostimulator, pisces-quad compact 3887 lead	0	0	0	1	0	0	1
C1767	Generator, neurostimulator (implantable), nonrechargeable	0	0	0	1	0	0	1
C1778	Lead, neurostimulator (implantable)	0	0	0	1	0	0	1
C1780	Lens, intraocular (new technology)	0	0	0	1	0	0	1
C1787	Patient programmer, neurostimulator	0	0	0	1	0	0	1
C1816	Receiver and/or transmitter, neurostimulator (implantable)	0	0	0	1	0	0	1
C1817	Septal defect implant system, intracardiac	0	0	0	1	0	0	1
C1820	Generator, neurostimulator (implantable), with rechargeable battery and charging system	0	0	0	1	0	0	1
C1822	Generator, neurostimulator (implantable), high frequency, with rechargeable battery and charging system	0	0	0	1	0	0	1
C1840	Lens, intraocular (telescopic)	0	0	1	0	0	0	1
C1841	Retinal prosthesis, includes all internal and external components	0	0	0	1	0	0	1
C1842	Retinal prosthesis, includes all internal and external components; add-on to C1841	0	0	0	1	0	0	1
C1883	Adaptor/extension, pacing lead or neurostimulator lead (implantable)	0	0	0	1	0	0	1
C1897	Lead, neurostimulator test kit (implantable)	0	0	0	1	0	0	1
C3851	Intraocular lens, staar elastic ultraviolet-absorbing silicone posterior chamber intraocular lens with toric optic model aa-4203t, model aa-4203tf, model aa-4203tl	0	0	0	1	0	0	1
C8521	Receiver/transmitter, neurostimulator, medtronic mattrix	0	0	0	1	0	0	1
C9132	Prothrombin complex concentrate (human), Kcentra, per IU of Factor IX activity	0	1	0	0	1	0	0
C9133	Factor IX (antihemophilic factor, recombinant), Rixibus, per IU	0	1	0	0	1	0	0
C9134	Factor XIII (antihemophilic factor, recombinant), Tretten, per 10 IU	0	1	0	0	1	0	0
C9135	Factor IX (antihemophilic factor, recombinant), Alprolix, per IU	0	1	0	0	1	0	0
C9136	Injection, factor VIII, Fc fusion protein, (recombinant), per IU	0	1	0	0	1	0	0
C9137	Injection, factor VIII (antihemophilic factor, recombinant) PEGylated, 1 IU	0	1	0	0	1	0	0
C9138	Injection, factor VIII (antihemophilic factor, recombinant) (Nuwiq), 1 IU	0	1	0	0	1	0	0
C9139	Injection, Factor IX, albumin fusion protein (recombinant), Idelvion, 1 IU	0	1	0	0	1	0	0
C9140	Injection, factor VIII (antihemophilic factor, recombinant) (Afstyla), 1 IU	0	1	0	0	1	0	0
C9267	Injection, von Willebrand factor complex (human), Wilate, per 100 IU VWF: RCO	0	1	0	0	1	0	0
E0751	Implantable neurostimulator pulse generator or combination of external transmitter with implantable receiver (includes extension)	0	0	0	1	0	0	1
E0752	Implantable neurostimulator electrode, each	0	0	0	1	0	0	1
E0753	Implantable neurostimulator electrodes, per group of four	0	0	0	1	0	0	1
E0754	Patient programmer (external) for use with implantable programmable neurostimulator pulse generator	0	0	0	1	0	0	1
E0756	Implantable neurostimulator pulse generator	0	0	0	1	0	0	1
E0757	Implantable neurostimulator radiofrequency receiver	0	0	0	1	0	0	1
E0758	Radiofrequency transmitter (external) for use with implantable neurostimulator radiofrequency receiver	0	0	0	1	0	0	1
E0759	Radiofrequency transmitter (external) for use with implantable sacral root neurostimulator receiver for bowel and bladder management, replacement	0	0	0	1	0	0	1
G0337	Hospice evaluation and counseling services, preelection	0	0	0	1	0	0	1
G0452	Molecular pathology procedure; physician interpretation and report	1	0	0	1	0	0	1
G8306	Primary open-angle glaucoma patient with intraocular pressure above the target range goal documented to have received plan of care	0	0	0	1	0	0	1
G8307	Primary open-angle glaucoma patient with intraocular pressure at or below goal, no plan of care necessary	0	0	0	1	0	0	1
G8308	Primary open-angle glaucoma patient with intraocular pressure above the target range goal, and not documented to have received plan of care during the reporting year	0	0	0	1	0	0	1
G9433	Death, permanent nursing home resident or receiving hospice or palliative care any time during the measurement period	0	0	0	1	0	0	1
G9473	Services performed by chaplain in the hospice setting, each 15 minutes	0	0	0	1	0	0	1
G9474	Services performed by dietary counselor in the hospice setting, each 15 minutes	0	0	0	1	0	0	1
G9475	Services performed by other counselor in the hospice setting, each 15 minutes	0	0	0	1	0	0	1
G9476	Services performed by volunteer in the hospice setting, each 15 minutes	0	0	0	1	0	0	1
G9477	Services performed by care coordinator in the hospice setting, each 15 minutes	0	0	0	1	0	0	1
G9478	Services performed by other qualified therapist in the hospice setting, each 15 minutes	0	0	0	1	0	0	1
G9479	Services performed by qualified pharmacist in the hospice setting, each 15 minutes	0	0	0	1	0	0	1
G9598	Aortic aneurysm 5.5 - 5.9 cm maximum diameter on centerline formatted CT or minor diameter on axial forma	0	0	0	0	0	1	0
G9599	Aortic aneurysm 6.0 cm or greater maximum diameter on centerline formatted CT or minor diameter on axial	0	0	0	0	0	1	0
G9662	Previously diagnosed or have an active diagnosis of clinical ASCVD	0	0	0	0	0	1	0
G9674	Patients with clinical ASCVD diagnosis	0	0	0	0	0	0	1
G9718	Hospice services for patient provided any time during the measurement period	0	0	0	1	0	0	1
G9720	Hospice services for patient occurred any time during the measurement period	0	0	0	1	0	0	1
G9723	Hospice services for patient received any time during the measurement period	0	0	0	1	0	0	1
G9740	Hospice services given to patient any time during the measurement period	0	0	0	1	0	0	1
G9785	Pathology report diagnosing cutaneous basal cell carcinoma or squamous cell carcinoma (to include in situ	0	0	0	0	0	0	1
G9786	Pathology report diagnosing cutaneous basal cell carcinoma or squamous cell carcinoma (to include in situ	0	0	0	0	0	0	1
G9843	Ras (KRAS or NRAS) gene mutation	1	0	0	1	0	0	1
G9857	Patient admitted to hospice	0	0	0	1	0	0	1
G9858	Patient enrolled in hospice	0	0	0	1	0	0	1
J2326	Injection, nusinersen, 0.1 mg	0	1	0	0	1	0	0
J7175	Injection, Factor X, (human), 1 I.U.	0	1	0	0	1	0	0
J7179	Injection, von Willebrand factor (recombinant), (VONVENDI), 1 I.U. VWF:RCo	0	1	0	0	1	0	0
J7180	Injection, Factor XIII (antihemophilic factor, human), 1 I.U.	0	1	0	0	1	0	0
J7181	Injection, Factor XIII A-subunit, (recombinant), per IU	0	1	0	0	1	0	0
J7182	Injection, Factor VIII, (antihemophilic factor, recombinant), (NovoEight), per IU	0	1	0	0	1	0	0
J7183	Injection, von Willebrand factor complex (human), Wilate, 1 I.U. VWF:RCo	0	1	0	0	1	0	0
J7185	Injection, Factor VIII (antihemophilic factor, recombinant) (Xyntha), per I.U.	0	1	0	0	1	0	0
J7186	Injection, antihemophilic Factor VIII/von Willebrand factor complex (human), per Factor VIII I.U.	0	1	0	0	1	0	0
J7187	Injection, von Willebrand factor complex (humate-p), per IU VWF:RCo	0	1	0	0	1	0	0
J7188	Injection, Factor VIII (antihemophilic factor, recombinant), (obizur), per I.U.	0	1	0	0	1	0	0
J7189	Factor VIIa (antihemophilic factor, recombinant), per 1 microgram	0	1	0	0	1	0	0
J7190	Factor VIII (antihemophilic factor, human) per I.U.	0	1	0	0	1	0	0
J7191	Factor VIII (antihemophilic factor (porcine)), per I.U.	0	1	0	0	1	0	0
J7192	Factor VIII (antihemophilic factor, recombinant) per I.U., not otherwise specified	0	1	0	0	1	0	0
J7193	Factor IX (antihemophilic factor, purified, non-recombinant) per I.U.	0	1	0	0	1	0	0
J7194	Factor IX, complex, per I.U.	0	1	0	0	1	0	0
J7195	Injection, Factor IX (antihemophilic factor, recombinant) per IU, not otherwise specified	0	1	0	0	1	0	0
J7196	Injection, antithrombin recombinant, 50 I.U.	0	1	0	0	1	0	0
J7197	Antithrombin III (human), per I.U.	0	1	0	0	1	0	0

J7199	Hemophilia clotting factor, not otherwise classified	0	1	0	0	1	0	0
J7200	Injection, Factor IX, (antihemophilic factor, recombinant), Rixubis, per IU	0	1	0	0	1	0	0
J7201	Injection, Factor IX, Fc fusion protein, (recombinant), Alprolix, 1 I.U.	0	1	0	0	1	0	0
J7202	Injection, Factor IX, albumin fusion protein, (recombinant), IDELVION, 1 I.U.	0	1	0	0	1	0	0
J7205	Injection, Factor VIII Fc fusion protein (recombinant), per IU	0	1	0	0	1	0	0
J7207	Injection, Factor VIII, (antihemophilic factor, recombinant), pegylated, 1 I.U.	0	1	0	0	1	0	0
J7209	Injection, Factor VIII, (antihemophilic factor, recombinant), (NUWIQ), 1 I.U.	0	1	0	0	1	0	0
J7210	Injection, Factor VIII, (antihemophilic factor, recombinant), (AFSTYLA), 1 I.U.	0	1	0	0	1	0	0
J7211	Injection, Factor VIII, (antihemophilic factor, recombinant), (KOVALTRY), 1 I.U.	0	1	0	0	1	0	0
L8679	Implantable neurostimulator, pulse generator, any type	0	0	0	1	0	0	1
L8680	Implantable neurostimulator electrode, each	0	0	0	1	0	0	1
L8681	Patient programmer (external) for use with implantable programmable neurostimulator pulse generator, repl	0	0	0	1	0	0	1
L8682	Implantable neurostimulator radiofrequency receiver	0	0	0	1	0	0	1
L8683	Radiofrequency transmitter (external) for use with implantable neurostimulator radiofrequency receiver	0	0	0	1	0	0	1
L8684	Radiofrequency transmitter (external) for use with implantable sacral root neurostimulator receiver for bowel and bladder management, replacement	0	0	0	1	0	0	1
L8685	Implantable neurostimulator pulse generator, single array, rechargeable, includes extension	0	0	0	1	0	0	1
L8686	Implantable neurostimulator pulse generator, single array, nonrechargeable, includes extension	0	0	0	1	0	0	1
L8687	Implantable neurostimulator pulse generator, dual array, rechargeable, includes extension	0	0	0	1	0	0	1
L8688	Implantable neurostimulator pulse generator, dual array, nonrechargeable, includes extension	0	0	0	1	0	0	1
L8689	External recharging system for battery (internal) for use with implantable neurostimulator, replacement only	0	0	0	1	0	0	1
L8695	External recharging system for battery (external) for use with implantable neurostimulator, replacement only	0	0	0	1	0	0	1
Q0160	Factor ix (antihemophilic factor, purified, non-recombinant) per i.u.	0	1	0	0	1	0	0
Q0187	Factor VIIa (coagulation factor, recombinant) per 1.2 mg	0	1	0	0	1	0	0
Q1001	New technology, intraocular lens, category 1 as defined in Federal Register notice, Vol. 65, date May 3, 2000	0	0	0	1	0	0	1
Q1002	New technology, intraocular lens, category 2 as defined in Federal Register notice, Vol. 65, dated May 3, 2000	0	0	0	1	0	0	1
Q1003	New technology, intraocular lens, category 3 (reduced spherical aberration)	0	0	0	1	0	0	1
Q1004	New technology, intraocular lens, category 4 as defined in Federal Register notice	0	0	0	1	0	0	1
Q1005	New technology, intraocular lens, category 5 as defined in Federal Register notice	0	0	0	1	0	0	1
Q2022	Von Willebrand factor complex, human, per IU	0	0	0	1	0	0	1
Q2023	Injection, factor VIII (antihemophilic factor, recombinant) (Xyntha), per IU	0	1	0	0	1	0	0
Q2041	Injection, von Willebrand factor complex (human), Wilate, 1 IU VWF:RCo	0	1	0	0	1	0	0
Q4096	Injection, von Willebrand factor complex human, ristocetin cofactor (not otherwise specified), per I.U. VWF:RCO	0	1	0	0	1	0	0
Q5001	Hospice or home health care provided in patient's home/residence	0	0	0	1	0	0	1
Q5002	Hospice or home health care provided in assisted living facility	0	0	0	1	0	0	1
Q5003	Hospice care provided in nursing long term care facility (LTC) or non-skilled nursing facility (NF)	0	0	0	1	0	0	1
Q5004	Hospice care provided in skilled nursing facility (SNF)	0	0	0	1	0	0	1
Q5005	Hospice care provided in inpatient hospital	0	0	0	1	0	0	1
Q5006	Hospice care provided in inpatient hospice facility	0	0	0	1	0	0	1
Q5007	Hospice care provided in long term care facility	0	0	0	1	0	0	1
Q5008	Hospice care provided in inpatient psychiatric facility	0	0	0	1	0	0	1
Q5009	Hospice or home health care provided in place not otherwise specified (NOS)	0	0	0	1	0	0	1
Q5010	Hospice home care provided in a hospice facility	0	0	0	1	0	0	1
Q9975	Injection, factor VIII, FC fusion protein (recombinant), per IU	0	1	0	0	1	0	0
S0255	Hospice referral visit (advising patient and family of care options) performed by nurse, social worker, or other designated staff	0	0	0	1	0	0	1
S0265	Genetic counseling, under physician supervision, each 15 minutes	0	0	1	0	0	1	0
S0271	Physician management of patient home care, hospice monthly case rate (per 30 days)	0	0	0	1	0	0	1
S0596	Phakic intraocular lens for correction of refractive error	0	0	0	1	0	0	1
S2060	Lobar lung transplantation	0	0	0	0	0	1	0
S2150	Bone marrow or blood-derived stem cells (peripheral or umbilical), allogeneic or autologous, harvesting,	0	0	0	1	0	0	1
S2152	Solid organ(s), complete or segmental, single organ or combination of organs; deceased or living donor(s)	0	0	0	1	0	0	1
S2400	Repair, congenital diaphragmatic hernia in the fetus using temporary tracheal occlusion, procedure perfor	0	0	0	1	0	0	1
S2402	Repair, congenital cystic adenomatoid malformation in the fetus, procedure performed in utero	0	0	0	1	0	0	0
S2409	Repair, congenital malformation of fetus, procedure performed in utero, not otherwise classified	0	0	0	1	0	0	1
S3800	Genetic testing for amyotrophic lateral sclerosis (ALS)	1	0	0	0	0	0	0
S3818	Complete gene sequence analysis; BRCA1 gene	1	0	0	0	0	0	0
S3819	Complete gene sequence analysis; BRCA2 gene	1	0	0	1	0	0	1
S3820	Complete BRCA1 and BRCA2 gene sequence analysis for susceptibility to breast and ovarian cancer	1	0	0	0	0	0	0
S3822	Single mutation analysis (in individual with a known BRCA1 or BRCA2 mutation in the family) for susceptibility to breast and ovarian cancer	1	0	0	0	0	0	0
S3823	Three-mutation BRCA1 and BRCA2 analysis for susceptibility to breast and ovarian cancer in Ashkenazi individuals	1	0	0	0	0	0	0
S3828	Complete gene sequence analysis; MLH1 gene	1	0	0	0	0	0	0
S3829	Complete gene sequence analysis; MSH2 gene	1	0	0	0	0	0	0
S3830	Complete MLH1 and MSH2 gene sequence analysis for hereditary nonpolyposis colorectal cancer (HNPCC) genetic testing	1	0	0	0	0	0	0
S3831	Single-mutation analysis (in individual with a known MLH1 and MSH2 mutation in the family) for hereditary nonpolyposis colorectal cancer (HNPCC) genetic testing	1	0	0	0	0	0	0
S3833	Complete APC gene sequence analysis for susceptibility to familial adenomatous polyposis (FAP) and attenuated fap	1	0	0	0	0	0	0
S3834	Single-mutation analysis (in individual with a known APC mutation in the family) for susceptibility to familial adenomatous polyposis (FAP) and attenuated FAP	1	0	0	0	0	0	0
S3835	Complete gene sequence analysis for cystic fibrosis genetic testing	1	0	1	0	0	1	0
S3837	Complete gene sequence analysis for hemochromatosis genetic testing	1	0	0	1	0	0	0
S3840	DNA analysis for germline mutations of the RET proto-oncogene for susceptibility to multiple endocrine ne	1	0	1	0	0	0	1
S3841	Genetic testing for retinoblastoma	1	0	1	0	0	0	1
S3842	Genetic testing for Von Hippel-Lindau disease	1	0	0	0	0	0	1
S3843	DNA analysis of the F5 gene for susceptibility to factor V Leiden thrombophilia	1	0	0	0	0	0	0
S3844	DNA analysis of the connexin 26 gene (GJB2) for susceptibility to congenital, profound deafness	1	0	0	1	0	0	1
S3845	Genetic testing for alpha-thalassemia	1	0	1	0	0	1	0
S3846	Genetic testing for hemoglobin E beta-thalassemia	1	0	1	0	0	1	0
S3847	Genetic testing for Tay-Sachs disease	1	0	1	0	0	1	0
S3848	Genetic testing for Gaucher disease	1	0	1	0	0	1	0
S3849	Genetic testing for Niemann-Pick disease	1	0	1	0	0	1	0
S3850	Genetic testing for sickle cell anemia	1	0	1	0	0	1	0
S3851	Genetic testing for Canavan disease	1	0	1	0	0	1	0
S3852	DNA analysis for APOE epsilon 4 allele for susceptibility to Alzheimer's disease	1	0	0	0	0	0	0
S3853	Genetic testing for myotonic muscular dystrophy	1	0	0	1	0	0	1
S3855	Genetic testing for detection of mutations in the presenilin - 1 gene	1	0	0	0	0	0	0
S3860	Genetic testing, comprehensive cardiac ion channel analysis, for variants in 5 major cardiac ion channel genes for individuals with high index of suspicion for familial long QT syndrome (	1	0	0	1	0	0	1
S3861	Genetic testing, sodium channel, voltage-gated, type V, alpha subunit (SCN5A) and variants for suspected	1	0	0	1	0	0	1
S3862	Genetic testing, family-specific ion channel analysis, for blood-relatives of individuals (index case) who have previously tested positive for a genetic variant of a cardiac ion channel syndr	1	0	0	1	0	0	1
S3865	Comprehensive gene sequence analysis for hypertrophic cardiomyopathy	1	0	0	1	0	1	0
S3866	Genetic analysis for a specific gene mutation for hypertrophic cardiomyopathy (HCM) in an individual with	1	0	0	1	0	1	0
S3870	Comparative genomic hybridization (CGH) microarray testing for developmental delay, autism spectrum disor	1	0	0	1	0	0	1
T2042	Hospice routine home care; per diem	0	0	0	1	0	0	1
T2043	Hospice continuous home care; per hour	0	0	0	1	0	0	1
T2044	Hospice inpatient respite care; per diem	0	0	0	1	0	0	1
T2045	Hospice general inpatient care; per diem	0	0	0	1	0	0	1
T2046	Hospice long-term care, room and board only; per diem	0	0	0	1	0	0	1
V2630	Anterior chamber intraocular lens	0	0	0	1	0	0	1
V2631	Iris supported intraocular lens	0	0	0	1	0	0	1
V2632	Posterior chamber intraocular lens	0	0	0	1	0	0	1
V2787	Astigmatism correcting function of intraocular lens	0	0	0	1	0	0	1

V2788	Presbyopia correcting function of intraocular lens	0	0	0	1	0	0	1

Critical Care Indicators

Code	Code Type	Description
CCA	AMA	Critical Care Medicine
CCM	AMA	Critical Care Medicine
CCP	AMA	Pediatric Critical Care Medicine
CCS	AMA	Surgical Critical Care
NCC	AMA	Critical Care Medicine (Neurological Surgery)
OCC	AMA	Obstetric Critical Care Medicine
PCC	AMA	Pulmonary Critical Care Medicine
94656	CPT	Ventilation assist and management, initiation of pressure or volume preset ventilators for assisted or controlled breathing; first day
94657	CPT	Ventilation assist and management, initiation of pressure or volume preset ventilators for assisted or controlled breathing; subsequent days
99289	CPT	Critical care services delivered by a physician, face-to-face, during an interfacility transport of critically ill or critically injured pediatric patient, 24 months of age or less; first 30-74 minutes of hands on care during transport
99290	CPT	Critical care services delivered by a physician, face-to-face, during an interfacility transport of critically ill or critically injured pediatric patient, 24 months of age or less; each additional 30 minutes (List separately in addition to code for primary service)
99293	CPT	Initial inpatient pediatric critical care, per day, for the evaluation and management of a critically ill infant or young child, 29 days through 24 months of age
99294	CPT	Subsequent inpatient pediatric critical care, per day, for the evaluation and management of a critically ill infant or young child, 29 days through 24 months of age
99295	CPT	Initial inpatient neonatal critical care, per day, for the evaluation and management of a critically ill neonate, 28 days of age or less
99296	CPT	Subsequent inpatient neonatal critical care, per day, for the evaluation and management of a critically ill neonate, 28 days of age or less
99297	CPT	Subsequent neonatal intensive care, per day, for the evaluation and management of a critically ill though stable neonate or infant Critically ill though stable neonates require cardiac and/or respiratory support (including ventilator and nasal CPAP when indicated), continuous or frequent vital sign monitoring, laboratory and blood gas interpretations, follow-up
99440	CPT	Newborn resuscitation: provision of positive pressure ventilation and/or chest compressions in the presence of acute inadequate ventilation and/or cardiac output
99481	CPT	Total body systemic hypothermia in a critically ill neonate per day (List separately in addition to code for primary procedure)
99482	CPT	Selective head hypothermia in a critically ill neonate per day (List separately in addition to code for primary procedure)
0188T	CPT	Remote real-time interactive video-conferenced critical care, evaluation and management of the critically ill or critically injured patient; first 30-74 minutes
0188T	CPT	Remote real-time interactive video-conferenced critical care, evaluation and management of the critically ill or critically injured patient; first 30-74 minutes
0189T	CPT	Remote real-time interactive video-conferenced critical care, evaluation and management of the critically ill or critically injured patient; each additional 30 minutes (List separately in addition to code for primary service)
0189T	CPT	Remote real-time interactive video-conferenced critical care, evaluation and management of the critically ill or critically injured patient; each additional 30 minutes (List separately in addition to code for primary service)
94002	CPT	Ventilation assist and management, initiation of pressure or volume preset ventilators for assisted or controlled breathing; hospital inpatient/observation, initial day
94003	CPT	Ventilation assist and management, initiation of pressure or volume preset ventilators for assisted or controlled breathing; hospital inpatient/observation, each subsequent day
94660	CPT	Continuous positive airway pressure ventilation (CPAP), initiation and management
94662	CPT	Continuous negative pressure ventilation (CNP), initiation and management
99184	CPT	Initiation of selective head or total body hypothermia in the critically ill neonate, includes appropriate patient selection by review of clinical, imaging and laboratory data, confirmation of esophageal temperature probe location, evaluation of amplitude EEG, supervision of controlled hypothermia, and assessment of patient tolerance of cooling

99291	CPT	Critical care, evaluation and management of the critically ill or critically injured patient; first 30-74 minutes
99291	CPT	Critical care, evaluation and management of the critically ill or critically injured patient; first 30-74 minutes
99292	CPT	Critical care, evaluation and management of the critically ill or critically injured patient; each additional 30 minutes (List separately in addition to code for primary service)
99292	CPT	Critical care, evaluation and management of the critically ill or critically injured patient; each additional 30 minutes (List separately in addition to code for primary service)
99466	CPT	Critical care face-to-face services, during an interfacility transport of critically ill or critically injured pediatric patient, 24 months of age or younger; first 30-74 minutes of hands-on care during transport
99467	CPT	Critical care face-to-face services, during an interfacility transport of critically ill or critically injured pediatric patient, 24 months of age or younger; each additional 30 minutes (List separately in addition to code for primary service)
99468	CPT	Initial inpatient neonatal critical care, per day, for the evaluation and management of a critically ill neonate, 28 days of age or younger
99469	CPT	Subsequent inpatient neonatal critical care, per day, for the evaluation and management of a critically ill neonate, 28 days of age or younger
99471	CPT	Initial inpatient pediatric critical care, per day, for the evaluation and management of a critically ill infant or young child, 29 days through 24 months of age
99472	CPT	Subsequent inpatient pediatric critical care, per day, for the evaluation and management of a critically ill infant or young child, 29 days through 24 months of age
99475	CPT	Initial inpatient pediatric critical care, per day, for the evaluation and management of a critically ill infant or young child, 2 through 5 years of age
99476	CPT	Subsequent inpatient pediatric critical care, per day, for the evaluation and management of a critically ill infant or young child, 2 through 5 years of age
99485	CPT	Supervision by a control physician of interfacility transport care of the critically ill or critically injured pediatric patient, 24 months of age or younger, includes two-way communication with transport team before transport, at the referring facility and during the transport, including data interpretation and report; first 30 minutes
99486	CPT	Supervision by a control physician of interfacility transport care of the critically ill or critically injured pediatric patient, 24 months of age or younger, includes two-way communication with transport team before transport, at the referring facility and during the transport, including data interpretation and report; each additional 30 minutes (List
61	EOR	Critical Care
81	HCFA	Critical care (intensivists) (eff 5/92)
0200	Revenue	Intensive care
0201	Revenue	Intensive care - Surgical
0202	Revenue	Intensive care - Medical
0203	Revenue	Intensive care - Pediatric
0204	Revenue	Intensive care - Psychiatric
0206	Revenue	Intensive care - Intermediate ICU
0207	Revenue	Intensive care - Burn care
0208	Revenue	Intensive care - Trauma
0209	Revenue	Intensive care - Other intensive care
0210	Revenue	Intensive care - Coronary care
0211	Revenue	Intensive care - Myocardial Infarction
0212	Revenue	Intensive care - Pulmonary Care
0213	Revenue	Intensive care - Heart Transplant
0214	Revenue	Intensive care - Intermediate CCU

0219	Revenue	Intensive care - Other Coronary Care
0233	Revenue	Incremental nursing charge rate - ICU
0234	Revenue	Incremental nursing charge rate - CCU
